# Efficacy and safety of Traditional Chinese Medicine in alleviating symptoms associated with myocardial bridge: a systematic review and meta-analysis

**DOI:** 10.3389/fphar.2025.1619617

**Published:** 2025-09-19

**Authors:** Jiaqi Ren, Teng Feng, Xize Wu, Qiuying Wu, Yuxi Huang, Yue Li, Kaifeng Yu, Lihong Gong

**Affiliations:** ^1^ Liaoning University of Traditional Chinese Medicine, Shenyang, Liaoning, China; ^2^ Affiliated Hospital of Liaoning University of Traditional Chinese Medicine, Shenyang, Liaoning, China

**Keywords:** myocardial bridge, randomized controlled trial, meta-analysis, angina, traditional Chinese medicine

## Abstract

**Background:**

With the advancement of technology, the detection rate of myocardial bridge (MB) has gradually increased and attracted attention. However, management options for symptomatic MB are limited, and Traditional Chinese Medicine (TCM) has emerged as a potential complementary approach for managing symptoms in MB patients. This study conducted a meta-analysis by pooling data from clinical randomized controlled trials (RCTs) to assess the efficacy and safety of TCM in alleviating symptoms in patients with MB.

**Methods:**

RCTs of TCM for MB were searched in PubMed, Embase, Web of Science, Cochrane Library, CBM, Wanfang, VIP, and CNKI databases from their inception to 1 April 2025. Patients diagnosed with MB *via* angiography were included in the study. The intervention group received either TCM alone (TCM-alone) or TCM combined with biomedicine (TCM + BM), while the control group received conventional biomedicine alone. Two investigators independently screened the literature according to the inclusion and exclusion criteria. The risk of bias in the included studies was assessed using Stata/MP 18.0 software. A meta-analysis was then conducted using RevMan 5.4.1 software to evaluate outcomes such as angina efficacy, electrocardiogram efficacy, TCM syndrome score efficacy, and the Seattle Angina Scale (SAQ). Subgroup analysis was performed according to the treatment regimen and duration of the intervention group.

**Results:**

A total of 18 publications were included, containing 1,224 participants, with 613 in the intervention group and 611 in the control group. Meta-analysis results showed that TCM significantly improved angina efficacy [RR = 1.30, 95% CI (1.21, 1.40), *P* < 0.00001], reduced angina attack frequency [MD = −0.96 episodes per week, 95% CI (−1.32, −0.59), *P* < 0.00001], improved electrocardiogram efficacy [RR = 1.31, 95% CI (1.20, 1.42), *P* < 0.00001], enhanced TCM syndrome scores [RR = 1.45, 95% CI (1.28, 1.64), *P* < 0.00001], and reduced physical limitation [MD = 5.95, 95% CI (2.25, 9.64), *P =* 0.002], angina stability [MD = 12.10, 95% CI (7.37, 16.83), *P* < 0.00001], angina frequency [MD = 11.29, 95% CI (6.93, 15.64), *P* < 0.00001], treatment satisfaction [MD = 23.44, 95% CI (19.26, 27.61), *P* < 0.00001], and disease perception [MD = 10.69, 95% CI (5.66, 15.72), *P* < 0.0001] scores in the SAQ, as well as Self-rating Anxiety Scale (SAS) [MD = −12.83, 95% CI (−13.95, −11.71), *P* < 0.00001] and Self-rating Depression Scale (SDS) [MD = −6.97, 95% CI (−8.41, −5.52), *P* < 0.00001] scores, and did not increase adverse reactions [RR = 0.82, 95% CI (0.51, 1.34), *P* = 0.43]. Subgroup analysis results indicated that, compared with the control group, both the TCM-alone [RR = 1.22, 95% CI (1.11, 1.34), *P* < 0.0001] and TCM + BM [RR = 1.38, 95% CI (1.24, 1.55), *P* < 0.00001] groups improved angina efficacy; the TCM + BM group improved ECG efficacy [RR = 1.26, 95% CI (1.16, 1.37), *P* < 0.00001] and TCM syndrome scores [RR = 1.54, 95% CI (1.30, 1.81), *P* < 0.00001], while the TCM-alone group did not improve ECG efficacy [RR = 1.53, 95% CI (0.92, 2.53), *P* = 0.10] or TCM syndrome scores [RR = 1.16, 95% CI (0.97, 1.40), *P* = 0.11]. *Salvia miltiorrhiza* Bunge [Lamiaceae; *Salviae miltiorrhizae radix et rhizoma*] and *Ligusticum chuanxiong* Hort. [Apiaceae; *Chuanxiong rhizoma*] are high-frequency medicinal substances for MB symptom management.

**Conclusion:**

TCM combined with biomedicine significantly improves angina symptoms, reduces attack frequency, enhances electrocardiographic parameters, alleviates TCM syndrome scores, and improves quality of life in patients with myocardial bridge, with a favorable safety profile. Crucially, TCM monotherapy showed no significant benefits for objective ischemia markers (ECG) or TCM syndrome scores, underscoring its role as a complementary adjunct rather than an alternative to standard care. However, these findings should be interpreted with caution due to the limited number of included RCTs, poor quality, small sample size, and single-center studies. Future large-scale, high-quality RCTs are warranted to confirm these results and further evaluate the efficacy and safety of TCM for symptom management in MB.

**Systematic Review Registration:**

identifier CRD420251000868.

## 1 Introduction

The coronary arteries, which supply oxygenated blood to the heart, originate from the aortic sinuses at the base of the aorta and branch into the left coronary artery and right coronary artery. The left coronary artery arises from the left coronary sinus of Valsalva and divides into two main branches: the left anterior descending (LAD) artery, which supplies the anterior portion of the heart, and the left circumflex artery, which encircles the lateral side of the heart. Meanwhile, the right coronary artery arises from the right aortic sinus and supplies the right ventricle and the inferior portion of the heart. Normally, these arteries run along the epicardium. However, in some congenital anomalies, a coronary artery segment may tunnel into the myocardium, forming an intramural segment, with the overlying myocardial muscle termed a myocardial bridge (MB)-first documented by Reyman (1737).

Initially considered a rare congenital anomaly, MB is now recognized as a common anatomical variant, with its true prevalence substantially underestimated by clinical imaging. A single-center retrospective study identified LAD myocardial bridge in 510 of 35,813 patients through coronary angiography, yielding a prevalence of 1.42%, which highlights the limitations of imaging detection (Matta et al., 2021). Whereas autopsy is considered the diagnostic “gold standard” for assessing the true prevalence of MB. A meta-analysis has revealed that MB is present in at least 40% of autopsied hearts (Hostiuc et al., 2018). These findings establish MB as a frequently occurring but often subclinical anatomical variant that is routinely underdetected in standard diagnostic practice. This discrepancy in detection rates arises because angiography only reveals compression during cardiac systole, whereas autopsy permits direct visualization of the anatomical structures.

The underlying pathological mechanisms of MB primarily involve the predisposition of atherosclerosis and the induction of myocardial ischemic symptoms. At the MB entrance, proximal segment hemodynamic alterations characterized by low and oscillatory wall shear stress promote endothelial dysfunction through increased vascular cell adhesion molecule-1 expression and excess reactive oxygen species production, contributing to a pro-atherosclerotic microenvironment. Concurrently, impaired systolic-diastolic flow leads to chronic blood supply-demand mismatch, exacerbated by aging, endothelial dysfunction, and plaque progression, ultimately manifesting clinically as ischemia (Corban et al., 2014). This ischemia is evidenced by reversible myocardial perfusion defects and reduced coronary flow velocity reserve in the LAD artery on stress echocardiography, which findings mirror those obstructive in coronary artery disease (CAD) (Guerra et al., 2023). Although MB was previously considered benign, recent evidence suggests otherwise (Moore et al., 2023). MB is significantly associated with reduced quality of life and adverse cardiac events (e.g., nonobstructive coronary myocardial infarction) and serves as an independent risk factor for cardiovascular risk in patients with normal coronary arteries (Ciliberti et al., 2022; Nie et al., 2021; Yang et al., 2024).

With the advancement of technology, the detection rate of MB has gradually increased, and the treatment of MB has attracted attention. Current management strategies include pharmacotherapy, interventional procedures, and surgery. First-line treatment for symptomatic patients involves medications such as beta-blockers and calcium antagonists, which alleviate vascular compression by reducing myocardial contractility and prolonging diastolic perfusion. For refractory cases, invasive options such as myocardial bridge release, coronary artery bypass grafting, or percutaneous stenting may be considered. However, interventional approaches carry the risk of in-stent restenosis and stent perforation, while surgical interventions are limited by higher morbidity and variable efficacy (Sternheim et al., 2021).

Given these constraints, there is a pressing need for safer, more effective therapies aligned with personalized medicine principles. Traditional Chinese medicine (TCM) has emerged as a promising adjunct in cardiovascular disease management. Its components include various bioactive compounds such as polyphenols, terpenoids, saponins, and alkaloids, which exert therapeutic effects through antioxidant, anti-inflammatory, and lipid-regulating properties (Hao et al., 2017). Within the theoretical framework of TCM, while there is no precise anatomical term equivalent to “myocardial bridge,” its clinical manifestations are commonly categorized under the syndrome of “chest bi” (chest obstruction/pain). TCM posits that the core pathogenesis involves “obstruction of the heart vessels and collaterals” (xin mai bi zu), characterized by a complex pattern of underlying deficiency (ben xu) and manifest excess (biao shi). The underlying deficiency entails insufficiency of qi, blood, yin, and yang, leading to pain due to lack of nourishment (bu rong ze tong). The manifest excess arises from pathogenic factors such as qi stagnation, blood stasis, and phlegm turbidity, resulting in pain due to obstruction (bu tong ze tong). Predisposing and exacerbating factors include emotional disharmony, improper diet, imbalance between exertion and rest, and aging accompanied by bodily deficiency. The clinical application of TCM interventions for symptomatic MB has garnered increasing attention. Therapeutic strategies primarily target pathomechanisms identified in TCM theory, including soothing the liver to resolve depression and regulating qi to relieve pain; regulating qi and activating blood circulation to alleviate pain; and tonifying qi to promote blood circulation and regulating qi to relieve pain. Common modalities comprise customized botanical decoctions and standardized proprietary Chinese medicines. Preliminary studies suggest potential benefits of TCM in reducing angina frequency, enhancing exercise tolerance, and improving quality of life in MB patients.

Despite these promising observations, current evidence exhibits critical limitations across the PICOS framework. ① Population: Inconsistent inclusion of symptomatic MB patients without anatomical stratification; ② Intervention: Heterogeneous TCM formulae lacking standardization and syndrome differentiation protocols; ③ Comparison: Non-uniform control treatments (e.g., variable beta-blocker regimens) and absence of placebo controls; ④ Outcomes: Non-validated TCM efficacy criteria and omission of objective hemodynamic metrics (e.g., coronary flow reserve); ⑤ Study design: Underpowered RCTs with high risk of bias due to inadequate blinding and allocation concealment. Crucially, no meta-analysis has addressed these gaps. Therefore, this study conducts a systematic review and meta-analysis of published RCTs using PICOS-defined criteria to evaluate TCM’s efficacy and safety *versus* conventional biomedicine in improving angina symptoms, electrocardiographic parameters, and TCM syndrome scores in MB patients. Results will provide evidence for optimizing integrative management of symptomatic MB.

## 2 Materials and methods

### 2.1 Protocol and registration

This study protocol adheres to the Preferred Reporting Items for Systematic Reviews and Meta-Analyses (PRISMA) guidelines (Page et al., 2021) and has been registered on the International Prospective Register of Systematic Reviews (PROSPERO) platform under the number CRD420251000868.

### 2.2 Information sources and search strategy

A comprehensive search was conducted across eight databases, namely, PubMed, Embase, Web of Science (WOS), Cochrane Library, China Biology Medicine Database (CBM), Wanfang Database, China Science and Technology Journal Database (VIP), and China National Knowledge Infrastructure (CNKI) Database, to identify all relevant studies on TCM interventions for MB published before 1 April 2025. The search employed both Medical Subject Headings (MeSH) terms and free-text keywords, with search strategies customized for each database’s unique characteristics. The complete search syntax for each database, including all MeSH terms and keyword variations, is documented in Supplementary Material S1. No language restrictions were applied to ensure maximal study inclusion.

### 2.3 Inclusion and exclusion criteria

The present systematic review encompasses an analysis of randomized controlled trials assessing the efficacy of TCM in alleviating symptoms associated with angiographically verified MB. The intervention group received either TCM alone or TCM combined with conventional biomedicine (TCM + BM), while the control group received conventional biomedicine alone. If the intervention group is also combined with conventional treatment, the conventional treatment regimen for the intervention group should be the same as that of the control group.

Primary outcome indicators included angina relief efficacy, electrocardiogram (ECG) improvement efficacy, and TCM symptom score reduction efficacy. Secondary outcomes included quality of life, as assessed by the Seattle Angina Questionnaire (SAQ), and psychological status, as assessed by the Self-Rating Anxiety Scale (SAS) and Self-Rating Depression Scale (SDS).

To ensure methodological rigor, studies with sample sizes of fewer than 15 participants per group were excluded from this study. Studies using multiple adjunctive non-pharmacological TCM therapies (acupuncture, moxibustion, massage, and acupoint injections) at the same time will be excluded. Master’s and doctoral theses are also excluded.

### 2.4 Data collection and extraction

Two independent researchers with methodological training conducted literature screening and data extraction using standardized forms, with cross-verification to ensure accuracy. Any discrepancies were resolved through discussion or consultation with a third party. The extraction protocol captured the following information: (1) study characteristics (first author, publication year, sample size, location); (2) participant details (Age, sex, disease duration); (3) Intervention protocols (TCM formulation name and composition, dosage regimen, treatment duration, conventional biomedicine regimen); (4) methodological quality indicators (randomization method, blinding, dropout rates); and (5) all predefined primary and secondary outcome indicators.

All formulae included in the studies were classified and assessed for compliance using the Consensus on Phytochemical Methods for Medicinal Plants (ConPhyMP) guidelines (Heinrich et al., 2022). Each botanical drug was taxonomically validated through the Medicinal Plant Names Services (MPNS, https://mpns.science.kew.org/mpns-portal) and Plants of the World Online (POWO, https://powo.science.kew.org/). For medicinal substances not resolved by these databases, verification was performed against regional pharmacopeia standards (e.g., Chinese Pharmacopoeia, Provincial Materia Medica Standards).

### 2.5 Risk of bias assessment

Two independent researchers conducted duplicate risk of bias evaluations using the Cochrane Risk of Bias Tool 1.0 (Cochrane ROB 1.0) and Cochrane Risk of Bias Tool 2.0 (Cochrane ROB 2.0), with subsequent cross-verification of the results. Cochrane ROB 1.0 assessment covered the following critical domains: (1) random sequence generation; (2) allocation concealment; (3) blinding of participants and personnel; (4) blinding of outcome assessments; (5) incomplete outcome data; (6) selective reporting; and (7) other biases. Each domain was rated as “low risk,” “high risk,” or “unclear” according to the ROB 1.0 criteria.

Cochrane ROB 2.0 including: (1) randomization process; (2) deviations from intended interventions; (3) missing outcome data; (4) measurement of the outcome; (5) selection of the reported result; and (6) overall bias. Each domain was rated as “low risk,” “high risk,” or “some concerns” according to the Cochrane ROB 2.0 criteria. Any disagreements were resolved through discussion or consultation with a senior reviewer.

### 2.6 Data analysis

For single-study comparisons, results were summarized descriptively. Meta-analyses were performed using RevMan 5.4.1 software when multiple studies were available. Continuous variables were analyzed using mean difference (MD) or standardized mean difference (SMD), while dichotomous variables were assessed using relative risk (RR). All effect sizes were reported with 95% confidence intervals (CI). Heterogeneity among study outcomes was evaluated using the *I* ^2^ statistic. A fixed-effects model was applied for low heterogeneity (*I* ^2^ < 50%), while a random-effects model was used for substantial heterogeneity (*I* ^2^ ≥ 50%), with potential sources of heterogeneity explored. Publication bias was assessed using funnel plots for all analyses. For analyses including eight or more studies (*n* ≥ 8), the Begg test was used; otherwise, the Egger test was employed. Association rule analysis based on the *Apriori* algorithm using SPSS Modeler. Cluster analysis was performed using SPSS Statistics 25.0.

## 3 Results

### 3.1 Search results

**FIGURE 1:**
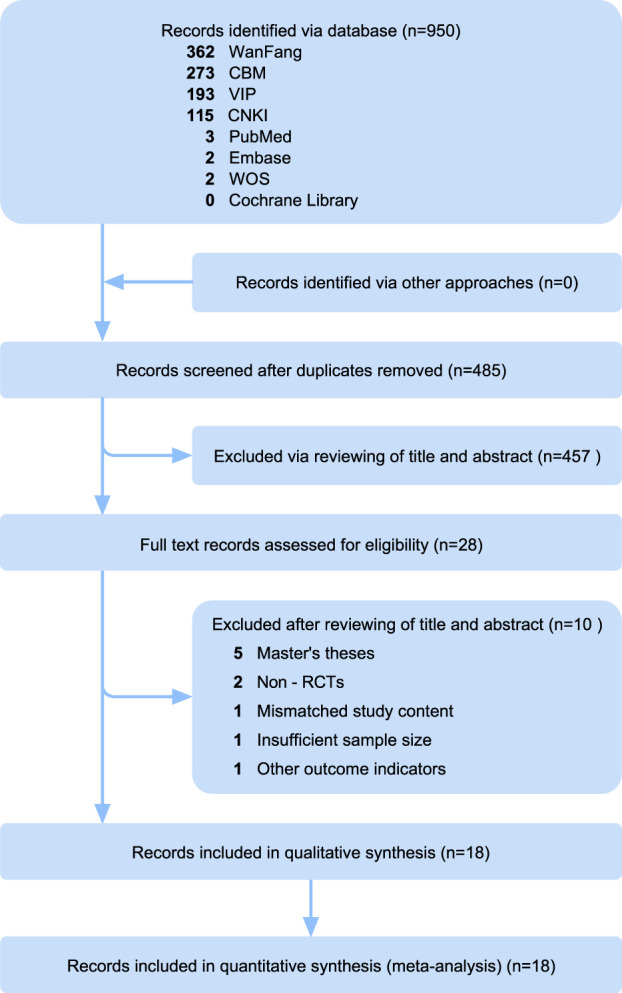
Literature screening flowchart.

The preliminary search yielded a total of 950 records, including 7 English-language articles and 943 Chinese-language publications. After removing duplicates, 485 articles were retained for further screening. Initial screening of titles and abstracts excluded 453 records, leaving 28 articles for full-text evaluation (Supplementary Material S1). Upon applying the predefined eligibility criteria, 10 articles were excluded for the following reasons: master’s theses (*n* = 5), non-RCTs (*n* = 2), insufficient sample size (<15 participants per group, *n* = 1), mismatched study content (*n* = 1), and other outcome indicators (*n* = 1). This process resulted in the final inclusion of 18 RCTs that met all the inclusion criteria. The specific process of literature screening is illustrated in Figure 1, in accordance with PRISMA guidelines.

### 3.2 Risk of bias evaluation

**FIGURE 2:**
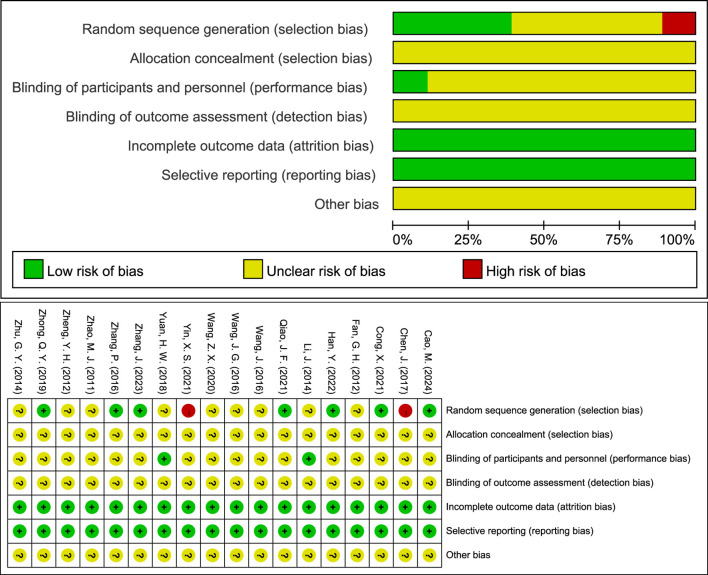
Risk of bias evaluation through Cochrane ROB 1.0.

The methodological quality of the 18 included RCTs was evaluated using the Cochrane ROB 1.0, with the following findings: 1) Random Sequence Generation: 7 RCTs used randomized numeric tables and were rated as “low risk”; 2 RCTs used the order of consultation and were rated as “high risk”; the remaining 9 RCTs mentioned randomization but did not describe the specific method and were rated as “unclear.” 2) Allocation Concealment: All studies lacked a description of concealment methods and were rated as “unclear.” 3) Blinding of Participants and Personnel: 2 RCTs were single-blinded and rated as “low risk”; the remaining RCTs did not report blinding procedures and were rated as “unclear.” 4) Blinding of Outcome Assessment: All included studies did not indicate whether the outcome assessors were blinded and were rated as “unclear.” 5) Incomplete Outcome Data: All studies demonstrated that patients completed the treatment with no loss to follow-up, rated as “low risk.” 6) Selective Reporting: All studies provided sufficient information to demonstrate all of the predefined primary and secondary outcomes and were therefore rated as “low risk.” 7) Other Bias: None of the included studies received funding from pharmaceutical companies, and no other factors that could have contributed to bias were identified. However, due to limited information, this domain was rated as “unclear.” Additionally, funnel plots for each outcome indicator were visualized to determine the presence of publication bias (Figure 2).

**FIGURE 3:**
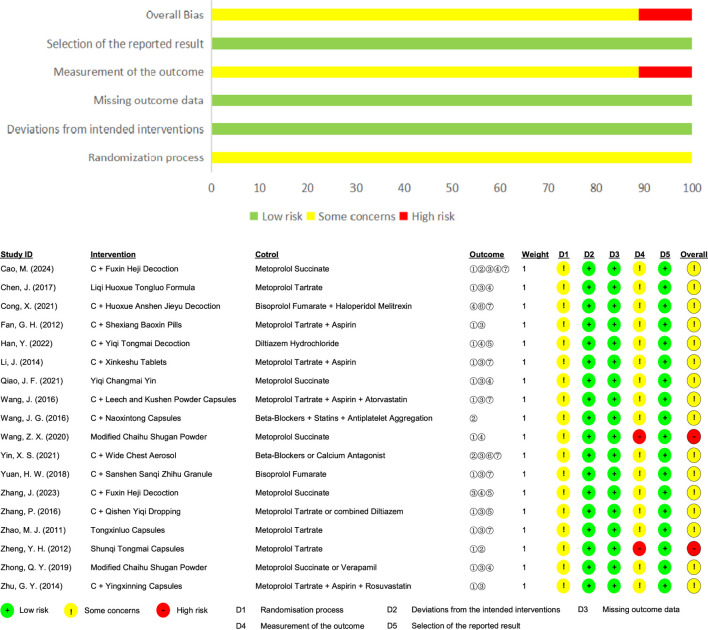
Risk of bias evaluation through Cochrane ROB 2.0.

Additionally, Cochrane ROB 2.0 was used to further assess the quality of the included RCTs, with the following findings: 1) Randomization Process: None of the studies specified allocation concealment, but the baseline characteristics were consistent across groups and were rated as “some concerns.” 2) Deviations from Intended Interventions: 2 RCTs were described as single-blinded, featuring no unplanned interventions or evenly distributed interventions among groups, and all participants finished the entire study, so they were rated as “low risk.” 3) Missing Outcome Data: All studies provided complete outcome data for all subjects and were rated as “low risk.” 4) Measurement of the Outcome: All studies employed appropriate measurement methods with no differences between groups, but the outcome assessors were aware of the interventions, and the outcome criteria were subjective in 2 studies (Wang and Han, 2020; Zheng et al., 2012), which led to a “high risk” rating; 3 studies utilized objective measures not included in this analysis (Cong et al., 2021; Wang J. G. et al., 2016; Han et al., 2022), such as electrocardiographic stress tests and carotid intima-media thickness, and were therefore rated as “some concerns”; the remaining studies used objective measures like electrocardiography and were also rated as “some concerns.” 5) Selection of the Reported Result: All studies did not selectively report outcome indicators and were rated as “low risk”; 6) Overall Bias: 2 RCTs were rated as “low risk,” and the remaining RCTs were rated as “some concerns.” (Figure 3).

### 3.3 Characteristics of included studies

The included 18 RCTs were conducted in China and published in Chinese-language journals (Wang and Han, 2020; Zheng et al., 2012; Cong et al., 2021; Wang J. G. et al., 2016; Han et al., 2022; Cao et al., 2024; Chen et al., 2017; Fan et al., 2012; Li, 2014; Qiao et al., 2021; Wang J. et al., 2016; Yin et al., 2021; Yuan et al., 2018; Zhang et al., 2023; Zhang et al., 2016; Zhao et al., 2011; Zhong and Guo, 2019; Zhu, 2014). These studies enrolled a total of 1,224 participants, with 613 in the intervention group and 611 in the control group. Individual study sample sizes ranged from 30 to 120 patients. The mean age across studies varied between 46.5 and 65.6 years in the intervention groups and 48.3 and 65.52 years in the control groups. Treatment durations ranged from 4 weeks to 12 months. In the control group, all 18 RCTs used conventional biomedicine alone (BM-alone); for the intervention group, 12 RCTs used a combination of TCM and biomedicine medicine (TCM + BM), and 6 RCTs used TCM alone (TCM-alone). All investigated formulae, including decoctions (e.g., Fuxin Heji Decoction), pills (e.g., Shexiang Baoxin Pills), capsules (e.g., Naoxintong Capsules), granules, and aerosols (e.g., Wide Chest Aerosol), were orally administered according to their original trial protocols. The basic characteristics of all RCTs included in the analysis are presented in Table 1.

**TABLE 1:** Basic characteristics of the included studies.

References	Sample Size		Age		Treatment		Treatment Duration	Outcome
	I	C	I	C	I	C		
Cao et al. (2024)	35	35	58.61 ± 3.46	58.25 ± 3.24	C + Fuxin Heji Decoction	Metoprolol Succinate	2 months	①②③④⑦
Chen et al. (2017)	60	60	53.48 ± 1.54	52.31 ± 3.55	Liqi Huoxue Tongluo Formula	Metoprolol Tartrate	4 weeks	①③④
Cong et al. (2021)	45	45	62.5 ± 7.2	61.7 ± 7.2	C + Huoxue Anshen Jieyu Decoction	Bisoprolol Fumarate + Haloperidol Melitrexin	3 months	④⑥⑦
Fan et al. (2012)	36	36	47.7 ± 6.4	49.4 ± 6.8	C + Shexiang Baoxin Pills	Metoprolol Tartrate + Aspirin	4 weeks	①③
Han et al. (2022)	32	32	53.3 ± 4.2	55.1 ± 3.2	C + Yiqi Tongmai Decoction	Diltiazem Hydrochloride	8 weeks	①④⑤
Li (2014)	30	30	64.7±*	62.2 ±*	C + Xinkeshu Tablets	Metoprolol Tartrate + Aspirin	6 months	①③⑦
Qiao et al. (2021)	30	30	62.77 ± 11.46	61.33 ± 12.74	Yiqi Changmai Yin	Metoprolol Succinate	8 weeks	①③④
Wang J. et al. (2016)	30	30	54.11±*	53.22 ±*	C + Leech and Kushen Powder Capsules	Metoprolol Tartrate + Aspirin + Atorvastatin	4 weeks	①③⑦
Wang J. G. et al. (2016)	40	40	62.5 ± 6.6	64.1 ± 7.1	C + Naoxintong Capsules	Beta-Blockers + Statins + Antiplatelet Aggregation	1 year	②
Wang and Han (2020)	25	25	64.15 ± 2.25	63.35 ± 2.27	Modified Chaihu Shugan Powder	Metoprolol Succinate	4 weeks	①④
Yin et al. (2021)	35	35	65.12 ± 2.34	65.52 ± 2.21	C + Wide Chest Aerosol	Beta-Blockers or Calcium Antagonist	10 months	②③⑥⑦
Yuan et al. (2018)	34	34	65.6±*	63.2 ±*	C + Sanshen Sanqi Zhihu Granule	Bisoprolol Fumarate	6 months	①③⑦
Zhang et al. (2023)	46	46	62.24 ± 4.67	63.39 ± 5.35	C + Fuxin Heji Decoction	Metoprolol Succinate	2 months	③④⑤
Zhang et al. (2016)	30	30	50.83 ± 4.01	51.74 ± 3.95	C + Qishen Yiqi Dropping	Metoprolol Tartrate or combined Diltiazem	8 weeks	①③⑤
Zhao et al. (2011)	20	18	49.7 ± 7.3	50.2 ± 4.2	Tongxinluo Capsules	Metoprolol Tartrate	6 weeks	①③⑦
Zheng et al. (2012)	40	40	61.8±*	60.5 ±*	Shunqi Tongmai Capsules	Metoprolol Tartrate	6 months	①②
Zhong and Guo (2019)	30	30	58.67 ± 9.15	60.77 ± 7.68	Modified Chaihu Shugan Powder	Metoprolol Succinate or Verapamil	6 weeks	①③④
Zhu (2014)	15	15	46.5 ± 5.4	48.3 ± 6.1	C + Yingxinning Capsules	Metoprolol Tartrate + Aspirin + Rosuvastatin	4 weeks	①③

① Angina Efficacy; ② Angina Frequency; ③ ECG Efficacy; ④ TCM Syndrome Score Efficacy; ⑤ SAQ; ⑥ SAS/SDS; ⑦ Adverse Reaction; “C” represents the control group, “I” represents the intervention group, * represents the SD not reported.

#### 3.3.1 Baseline characteristics of age

**FIGURE 4:**
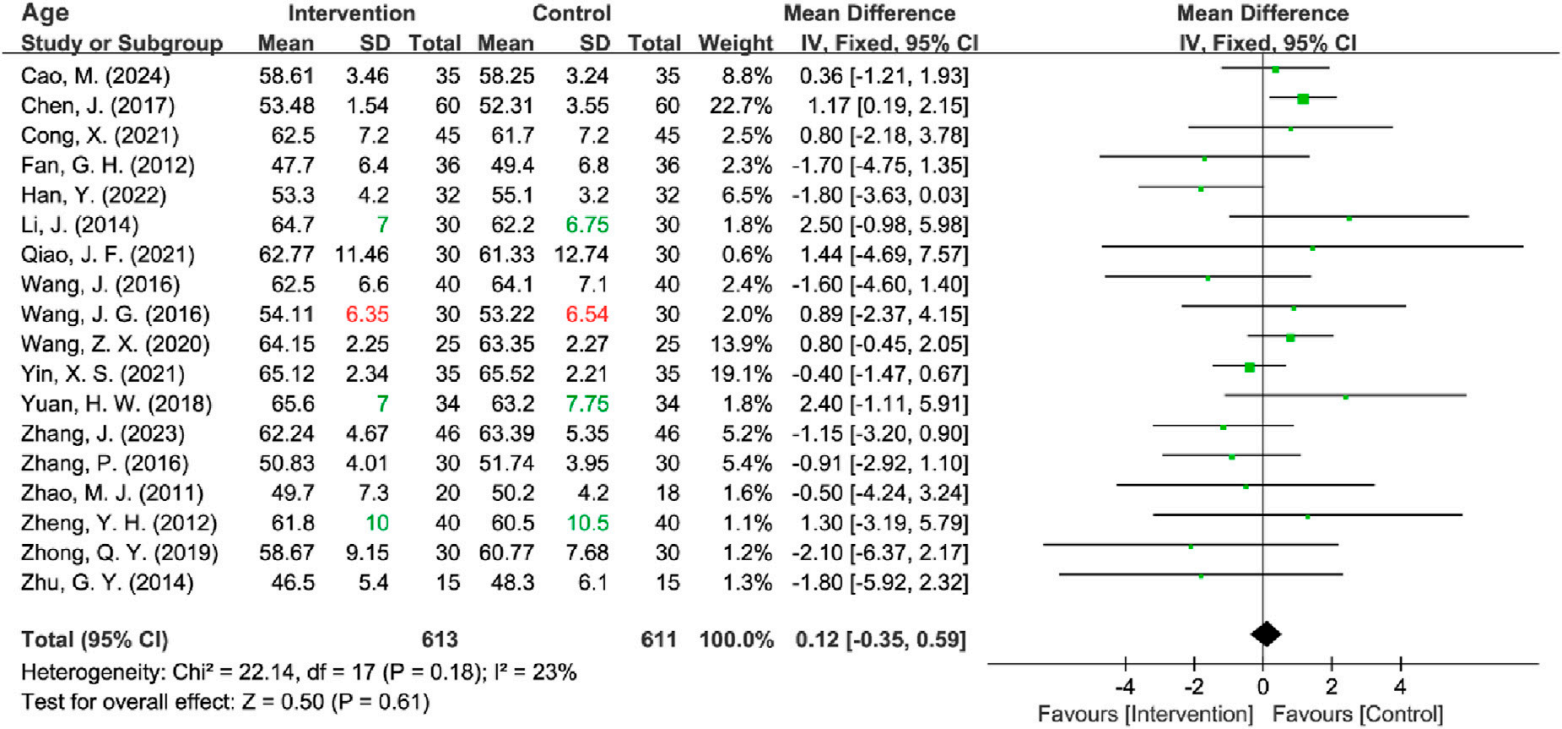
Forest plot of age.

All included RCTs reported age data, but the standard deviation (SD) was missing for four trials. Among these, three studies provided mean, maximum, and minimum values for age (Zheng et al., 2012; Li, 2014; Yuan et al., 2018), while one study provided only the mean values (Wang J. G. et al., 2016). Firstly, the missing SD for all four studies was interpolated using three methods: mean imputation, sample size-weighted mean imputation, and median imputation. Subsequently, to estimate the missing SDs, Hozo’s Range Rule (Hozo et al., 2005), applicable for sample sizes between 15 and 70, was first utilized for the three studies with partial data. For the study that provided only mean values, the above three methods were also used to estimate the missing SD. Regardless of the method used to fill in the missing values, all approaches demonstrated lower heterogeneity (all *I* ^2^ ≤ 35%). Consequently, the range rule + sample size-weighted mean imputation method was ultimately employed for the analysis, and a fixed-effects model was utilized for subsequent analyses. The results indicated no statistically significant difference in age between the intervention and control groups [MD = 0.12, 95% CI (−0.35, 0.59), Z = 0.50, *P* = 0.61] (Figure 4). After sensitivity analyses excluding 4 RCTs [*I* ^2^ = 28%; Fixed-effects model: MD = 0.00, 95% CI (−0.48, 0.48), Z = 0.00, *P* = 1.00] and 1 [*I* ^2^ = 27%; Fixed-effects model: MD = 0.10, 95% CI (−0.37, 0.57), Z = 0.43, *P* = 0.67] with imputed SD, the pooled MD remained nonsignificant (Table 2) (Supplementary Material S2).

**TABLE 2:** Results of sensitivity analysis, heterogeneity test, and meta-analysis of baseline characteristics.

Baseline	Method	Heterogeneity test		Effect model	Meta-analysis		
		*I* ^2^	*P*		MD (95% CI)	Z	*P*
Age	Mean Imputation	33%	0.09	Fixed	0.21 (−0.24, 0.67)	0.93	0.35
	Size-Weighted Mean Imputation	30%	0.11	Fixed	0.18 (−0.27, 0.64)	0.78	0.43
	Median Imputation	36%	0.06	Fixed	0.25 (−0.19, 0.70)	1.11	0.27
	Range +Mean Imputation	23%	0.18	Fixed	0.12 (−0.34, 0.59)	0.52	0.61
	Range + Size-Weighted Mean Imputation	23%	0.18	Fixed	0.12 (−0.35, 0.59)	0.50	0.61
	Range + Median Imputation	23%	0.18	Fixed	0.12 (−0.35, 0.59)	0.51	0.61
	Remaining 14 RCTs	28%	0.16	Fixed	0.00 (−0.48, 0.48)	0.00	1.00
	Remaining 17 RCTs	27%	0.15	Fixed	0.10 (−0.37, 0.57)	0.43	0.67
Disease Duration	Mean Imputation	61%	0.04	Radom	0.03 (−0.15, 0.22)	0.36	0.72
	Size-Weighted Mean Imputation	55%	0.06	Radom	0.02 (−0.16, 0.19)	0.17	0.87
	Median Imputation	72%	0.007	Radom	0.06 (−0.14, 0.26)	0.59	0.56
	Remaining 4 RCTs	63%	0.04	Radom	0.00 (−0.19, 0.19)	0.03	0.98

#### 3.3.2 Baseline characteristics of disease duration

**FIGURE 5:**
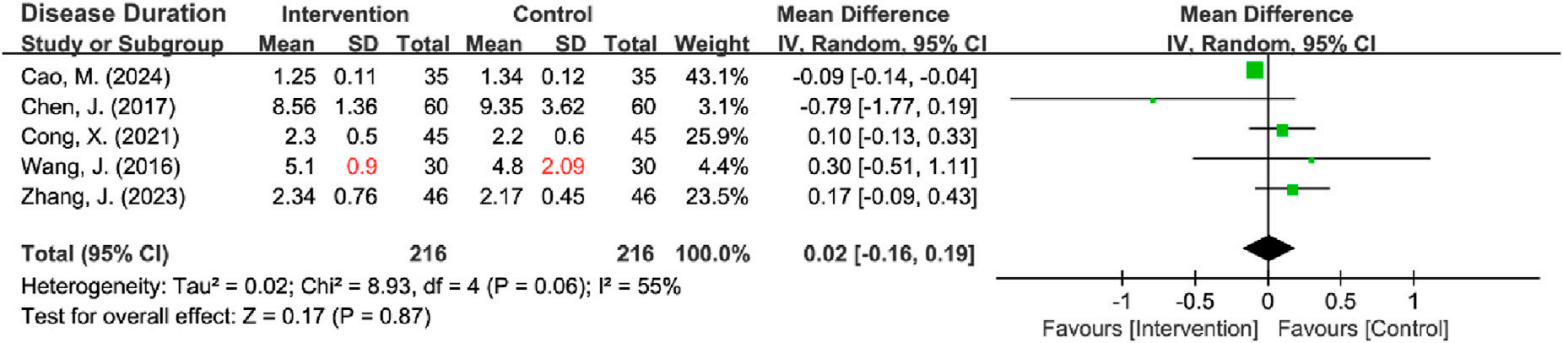
Forest plot of disease duration.

Five RCTs reported on disease duration (Cong et al., 2021; Cao et al., 2024; Chen et al., 2017; Wang J. et al., 2016; Zhang et al., 2023), with 1 missing the SD (Wang J. et al., 2016). This missing SD was estimated using three methods: mean imputation (*I* ^2^ = 61%, *P* = 0.04), sample size-weighted mean imputation (*I* ^2^ = 55%, *P* = 0.06), and median imputation (*I* ^2^ = 72%, *P* = 0.007), and all methods revealed significant heterogeneity. Given that the heterogeneity stemmed from natural differences in the characteristics of the included populations rather than flawed study design, the missing SD was imputed using the sample size-weighted mean imputation method, and a random-effects model was subsequently employed for the analysis. The results indicated no statistically significant difference in the disease duration between the intervention and control groups [MD = 0.02, 95% CI (−0.16, 0.19), Z = 0.17, *P* = 0.87] (Figure 5). Sensitivity analyses, excluding the RCT with missing SD, yielded identical results [*I* ^2^ = 63%; Radom-effects model: MD = 0.00, 95% CI (−0.19, 0.19)] (Table 2) (Supplementary Material S2).

### 3.4 Efficacy analysis

#### 3.4.1 Angina efficacy analysis

**FIGURE 6:**
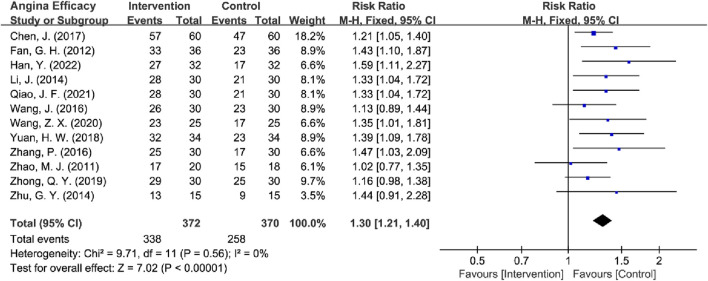
Forest plot of angina efficacy.

Among the 14 RCTs evaluating angina efficacy (Wang and Han, 2020; Zheng et al., 2012; Han et al., 2022; Cao et al., 2024; Chen et al., 2017; Fan et al., 2012; Li, 2014; Qiao et al., 2021; Wang J. et al., 2016; Yuan et al., 2018; Zhang et al., 2016; Zhao et al., 2011; Zhong and Guo, 2019; Zhu, 2014), 1 study by Cao et al. (2024) was excluded because it did not report angina efficacy rates, and 1 study by Zheng et al. (2012) was excluded due to the use of independent efficacy ratings for chest tightness, chest pain, palpitations, and dyspnea that were inconsistent with other studies. Therefore, the remaining 12 RCTs were analyzed for angina efficacy. The absence of heterogeneity (*I* ^2^ = 0%, *P* = 0.56) justified the use of a fixed-effect model for analysis. Pooled analysis of the remaining 12 RCTs demonstrated that TCM provided significantly improved angina relief compared to BM-alone in MB patients [RR = 1.30, 95% CI (1.21, 1.40), Z = 7.02, *P* < 0.00001] (Figure 6). Subsequently, high-risk studies by Wang and Han (2020) were removed for sensitivity analyses [*I* ^2^ = 0%, RR = 1.30, 95% CI (1.20, 1.40), Z = 6.72, *P* < 0.00001], and the results were consistent with previous ones.

**FIGURE 7:**
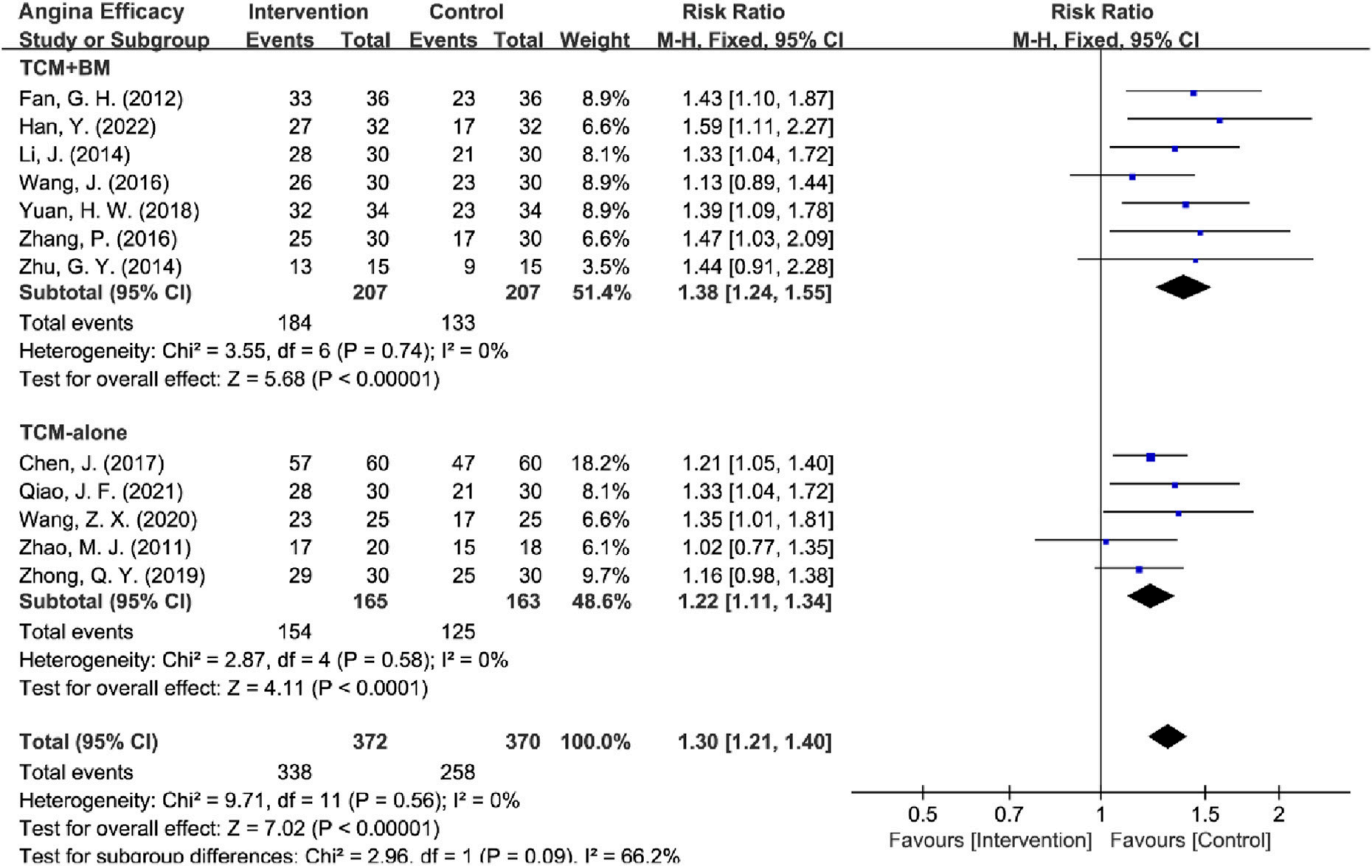
Forest plot of angina efficacy based on subgroups of interventions.

The interventions were categorized into two subgroups: TCM-alone (*n* = 5) and TCM + BM (*n* = 7). The heterogeneity test indicated low heterogeneity within both subgroups (TCM-alone: I = 0%, *P* = 0.58; TCM + BM: *I* ^2^ = 0%, *P* = 0.74), which justified the application of a fixed-effects model for the analysis. The results demonstrated that both subgroups significantly improved angina efficacy compared to the control group [TCM-alone: RR = 1.22, 95% CI (1.11, 1.34), Z = 4.11, *P* < 0.0001; TCM + BM: RR = 1.38, 95% CI (1.24, 1.55), Z = 5.68, *P* < 0.00001]. Additionally, the efficacy of TCM + BM was slightly higher than that of TCM-alone, although there was no statistically significant difference between the two subgroups (*P* = 0.09). Sensitivity analyses in removing high-risk articles yielded the same conclusions [TCM-alone: RR = 1.20, 95% CI (1.08, 1.32), Z = 3.59, *P =* 0.0003; TCM + BM: RR = 1.38, 95% CI (1.24, 1.55), Z = 5.68, *P* < 0.00001], these findings suggest that both TCM-alone and TCM + BM are significantly more effective than BM-alone in improving angina efficacy, with low heterogeneity and high reliability of the results (Figure 7).

Subsequently, the studies were categorized into two distinct groups based on treatment durations: one group included studies with treatment durations of 30 days or less (≤30 days, *n* = 5), and the other included studies with durations exceeding 30 days (>30 days, *n* = 7). Similarly, for the 45-day and 60-day treatment durations, studies were divided into those with durations of 45 days or less (≤45 days, *n* = 7) and those longer than 45 days (>45 days, *n* = 5), and ≤60 days (*n* = 10) and >60 days (*n* = 2), respectively. Heterogeneity tests across all subgroups indicated low heterogeneity (all *I* ^2^ < 25%), which justified the use of a fixed-effects model for analysis. The results demonstrated that, compared to the control group, treatments of varying lengths (30, 45, and 60 days) significantly improved angina efficacy. Sensitivity analyses for deleting high-risk articles yielded consistent findings.

Furthermore, the studies were divided into three distinct subgroups based on treatment durations: less than 30 days (≤30 days, *n* = 5) (*I* ^2^ = 0%, *P* = 0.62), 30–60 days (30–60 days, *n* = 5) (*I* ^2^ = 38%, *P* = 0.17), and more than 60 days (>60 days, *n* = 2) (*I* ^2^ = 0%, *P* = 0.81). Low heterogeneity suggests the use of a fixed-effects model analysis. The results indicated significant improvement in angina efficacy across different intervention durations compared to the control group. Sensitivity analysis using random-effects models and deletion of high-risk articles yielded the same conclusion.

Moreover, the 30–60 days were divided into two distinct subgroups: 30–45 days (*n* = 2) and 45–60 days (*n* = 3). The low heterogeneity in both the 30–45 days (*I* ^2^ = 0%) and 45–60 days groups (*I* ^2^ = 0%), thus warranting the use of a fixed-effects model for analysis. The results showed improved angina efficacy in the 45–60 days [RR = 1.45, 95% CI (1.21, 1.75), Z = 3.99, *P* < 0.0001] group compared to the control group, while there was no statistically significant difference in the 30–45 days [RR = 1.11, 95% CI (0.95, 1.28), Z = 1.32, *P* = 0.19] group, which may have been caused by the small sample size (Supplementary Material S3). Similarly, sensitivity analyses in removing high-risk articles yielded consistent findings. Sensitivity analysis for deleting high-risk articles yielded the same conclusion.

This analysis demonstrated that, compared to the control group, both TCM + BM and TCM-alone treatments, regardless of shorter or longer treatment durations, significantly improved angina symptoms in patients with MB. Additionally, the effect size of TCM + BM was greater than that of TCM-alone, and the effect size of long-term treatment (>45 days) was greater than that of short-term treatment (<45 days), although these differences were not statistically significant (Table 3). Therefore, further RCTs with larger samples are needed for validation.

**TABLE 3:** Subgroup analysis of angina efficacy.

Difference	Subgroup	Heterogeneity test		Effect model	Meta analysis		
		*I* ^2^	*P*		RR (95% CI)	Z/*x* ^2^	*P*
Total	Intervention	0%	0.56	Fixed	1.30 (1.21, 1.40)	7.02	<0.00001
Sensitivity Analysis	Deletion of high-risk	0%	0.48	Fixed	1.30 (1.20, 1.40)	6.72	<0.00001
Interventions	TCM + BM	0%	0.74	Fixed	1.38 (1.24, 1.55)	5.68	<0.00001
	TCM-alone	0%	0.58	Fixed	1.22 (1.11, 1.34)	4.11	<0.0001
	Inter-subgroup	66.2%				2.96	0.09
Sensitivity Analysis	TCM + BM	0%	0.74	Fixed	1.38 (1.24, 1.55)	5.68	<0.00001
Deletion of high-risk	TCM-alone	0%	0.54	Fixed	1.20 (1.08, 1.32)	3.59	0.0003
	Inter-subgroup	72.9%				3.69	0.05
30-day Treatment	≤30 days	0%	0.62	Fixed	1.28 (1.15, 1.42)	4.53	<0.00001
	>30 days	16%	0.31	Fixed	1.32 (1.19, 1.47)	5.36	<0.00001
	Inter-subgroup	0%				0.23	0.63
45-day Treatment	≤45 days	0%	0.55	Fixed	1.23 (1.13, 1.35)	4.67	<0.00001
	>45 days	0%	0.93	Fixed	1.41 (1.24, 1.61)	5.25	<0.00001
	Inter-subgroup	65.9%				2.93	0.09
60-day Treatment	≤60 days	0%	0.45	Fixed	1.29 (1.19, 1.40)	6.14	<0.00001
	>60 days	0%	0.81	Fixed	1.36 (1.14, 1.63)	3.44	0.0006
	Inter-subgroup	0%				0.31	0.58
Treatment Duration	≤30 days	0%	0.62	Fixed	1.28 (1.15, 1.42)	4.53	<0.00001
	30–60 days	38%	0.17	Fixed	1.31 (1.15, 1.48)	4.15	<0.0001
	>60 days	0%	0.81	Fixed	1.36 (1.14, 1.63)	3.44	0.0006
	Inter-subgroup	0%				0.39	0.82
Sensitivity Analysis	≤30 days	0%	0.62	Random	1.26 (1.13, 1.39)	4.35	<0.0001
	30–60 days	38%	0.17	Random	1.26 (1.08, 1.46)	2.94	0.003
	>60 days	0%	0.81	Random	1.36 (1.14, 1.63)	3.43	0.0006
	Inter-subgroup	0%				0.66	0.72
Treatment Duration	≤30 days	0%	0.62	Fixed	1.28 (1.15, 1.42)	4.53	<0.0001
	30–45 days	0%	0.43	Fixed	1.11 (0.95, 1.28)	1.32	0.19
	45–60 days	0%	0.71	Fixed	1.45 (1.21, 1.75)	3.99	<0.0001
	>60 days	0%	0.81	Fixed	1.36 (1.14, 1.63)	3.44	0.0006
	Inter-subgroup	50%				6.00	0.11
Sensitivity Analysis	≤30 days	0%	0.50	Fixed	1.26 (1.13, 1.42)	4.06	<0.0001
Deletion of high-risk	30–45 days	0%	0.43	Fixed	1.11 (0.95, 1.28)	1.32	0.19
	45–60 days	0%	0.71	Fixed	1.45 (1.21, 1.75)	3.99	<0.0001
	>60 days	0%	0.81	Fixed	1.36 (1.14, 1.63)	3.44	0.0006
	Inter-subgroup	50%				6.00	0.11

#### 3.4.2 Angina attack frequency efficacy analysis

**FIGURE 8:**
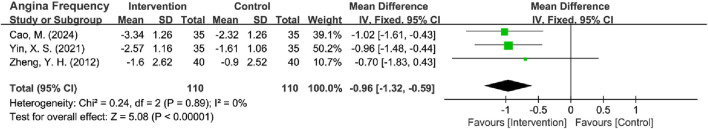
Forest plot of angina attack frequency after excluding heterogeneous sources.

Four RCTs reported the frequency of angina attacks (Zheng et al., 2012; Wang J. G. et al., 2016; Cao et al., 2024; Yin et al., 2021), with th studies (Zheng et al., 2012; Cao et al., 2024; Yin et al., 2021) using ‘episodes/week’ as the measurement unit and 1 Wang J. G. et al. (2016) study employing ‘episodes/year.’ To ensure data comparability, annual frequency measurements were converted to weekly frequency by dividing by 52 weeks. The treatment effect was evaluated by calculating the MD in attack frequency between pre- and post-treatment assessments, with corresponding SDs derived using established Cochrane-recommended methods for change-from-baseline data. The initial pooled analysis showed substantial heterogeneity (*I* ^2^ = 88%, *P* < 0.0001). Sensitivity analysis identified Wang, J. G. (2016) as the primary contributor to this heterogeneity. The exclusion of this particular trial eliminated heterogeneity (*I* ^2^ = 0%, *P* = 0.89), warranting the application of a fixed-effects model for final analysis. The results revealed a statistically significant reduction in angina attack frequency in the intervention group over the control group [MD = −0.96 episodes per week, 95% CI (−1.32, −0.59), Z = 5.08, *P* < 0.00001] (Figure 8). This finding remained directionally consistent in sensitivity analyses including all 4 RCTs [Random-effects model: MD = −0.64 episodes per week, 95% CI (−1.32, 0.05), Z = 1.83, *P* = 0.07], though the effect size was attenuated and lost statistical significance (Supplementary Material S3).

**FIGURE 9:**
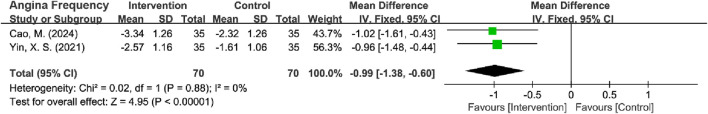
Forest plot of angina attack frequency after excluding high-risk articles and heterogeneous sources.

In addition, sensitivity analyses were performed by deleting high-risk articles from Zheng et al. (2012) and deleting heterogeneous sources, Wang J. G. et al. (2016), which yielded consistent findings [MD = −0.99 episodes per week, 95% CI (−1.38, −0.60), Z = 4.95, *P* < 0.00001] (Figure 9) (Supplementary Material S3).

#### 3.4.3 ECG efficacy analysis

**FIGURE 10:**
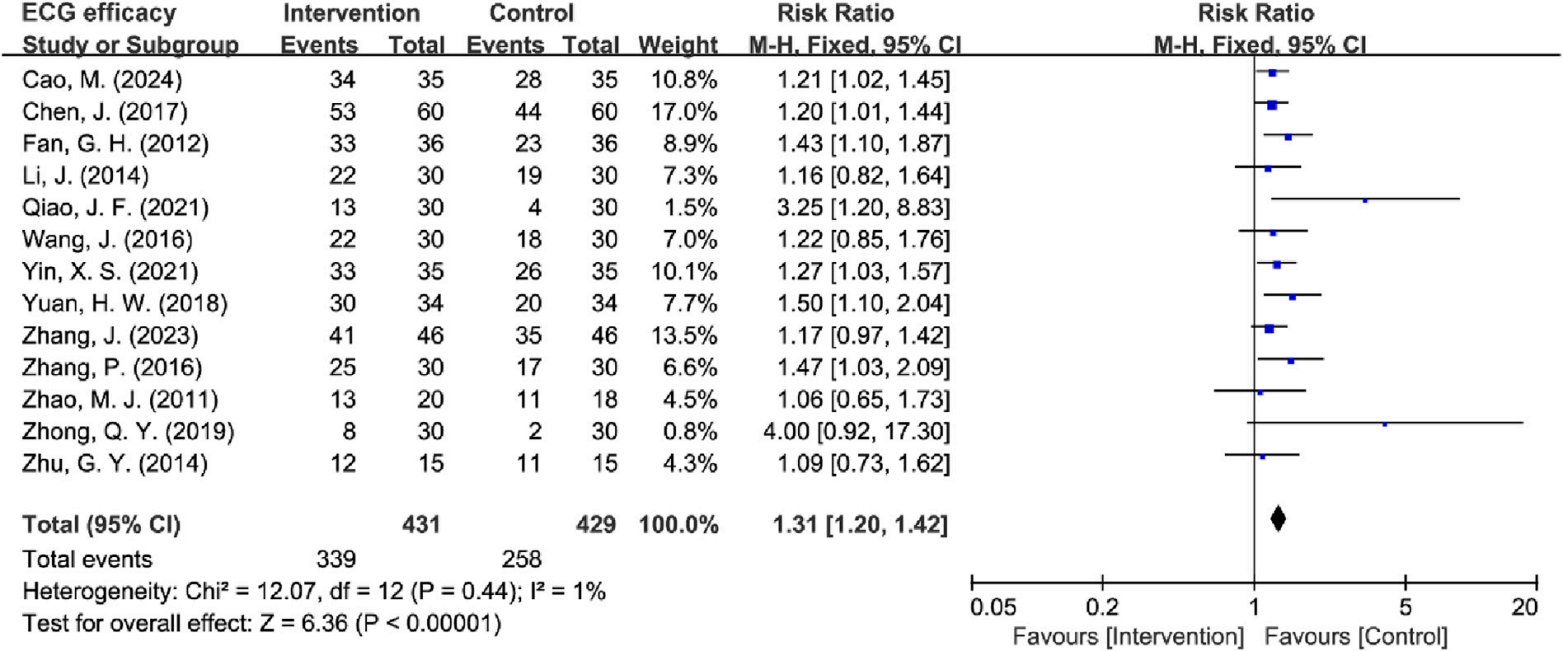
Forest plot of ECG efficacy.

13 RCTs evaluated ECG efficacy (Cao et al., 2024; Chen et al., 2017; Fan et al., 2012; Li, 2014; Qiao et al., 2021; Wang J. et al., 2016; Yin et al., 2021; Yuan et al., 2018; Zhang et al., 2023; Zhang et al., 2016; Zhao et al., 2011; Zhong and Guo, 2019; Zhu, 2014), the results demonstrated low between-study heterogeneity (*I* ^2^ = 1%, *P* = 0.44), supporting the use of a fixed-effects model. The pooled results revealed that TCM treatment produced significantly superior ECG improvement compared to the control group in MB patients [RR = 1.31, 95% CI (1.20, 1.42), Z = 6.36, *P* < 0.00001] (Figure 10).

**FIGURE 11:**
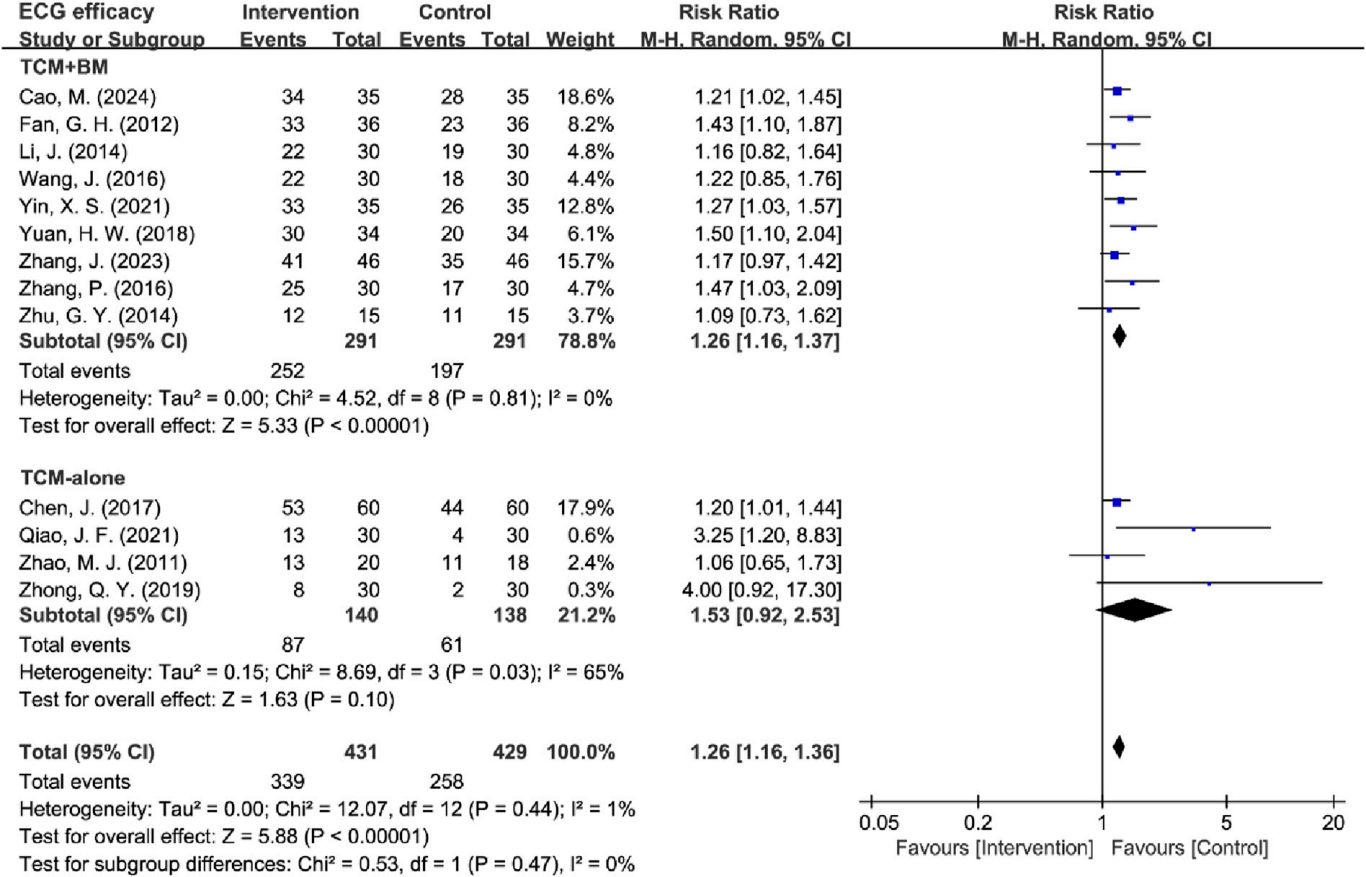
Forest plot of ECG efficacy based on subgroups of interventions.

The included studies were stratified into two intervention subgroups: TCM-alone (*n* = 4) and TCM + BM (*n* = 9). Heterogeneity assessment demonstrated low between-study variability in the TCM + BM subgroup (*I* ^2^ = 0%, *P* = 0.81), while the TCM-alone group exhibited moderate heterogeneity (*I* ^2^ = 65%, *P* = 0.03), so a random-effects model was used for subsequent analysis. The meta-analysis revealed that TCM + BM intervention [RR = 1.26, 95% CI (1.16, 1.37), Z = 5.33, *P* < 0.00001] significantly improved ECG efficacy compared to the control group, whereas TCM-alone [RR = 1.53, 95% CI (0.92, 2.53), Z = 1.63, *P* = 0.10] did not (Figure 11). Sensitivity analysis pointed out that Qiao (2021) and Zhong (2021) were the principal contributors to heterogeneity in the TCM-alone group, which was significantly reduced after exclusion (*I* ^2^ = 0%, *P* = 0.82) and yielded the same results (Table 4).

**TABLE 4:** Subgroup analysis of ECG efficacy.

Difference	Subgroup	Heterogeneity test		Effect model	Meta analysis		
		*I* ^2^	*P*		RR (95% CI)	Z/*x* ^2^	*P*
Total	Intervention	1%	0.44	Fixed	1.31 (1.20, 1.42)	6.36	<0.00001
Interventions	TCM + BM	0%	0.81	Random	1.26 (1.16, 1.37)	5.33	<0.00001
	TCM-alone	65%	0.03	Random	1.53 (0.92, 2.53)	1.63	0.10
	Inter-subgroup	0%				0.53	0.47
Sensitivity Analysis	TCM + BM	0%	0.81	Fixed	1.28 (1.17, 1.40)	5.34	<0.00001
	TCM-alone	0%	0.63	Fixed	1.18 (0.99, 1.40)	1.83	0.07
	Inter-subgroup	0%				0.73	0.39
30-day Treatment	≤30 days	0%	0.64	Fixed	1.25 (1.10, 1.43)	3.32	0.0009
	>30 days	27%	0.20	Fixed	1.34 (1.21, 1.49)	5.44	<0.00001
	Inter-subgroup	0%				0.69	0.41
45-day Treatment	≤45 days	0%	0.45	Fixed	1.28 (1.12, 1.46)	3.67	0.0002
	>45 days	20%	0.28	Fixed	1.33 (1.20, 1.48)	5.26	<0.00001
	Inter-subgroup	0%				0.18	0.67
60-day Treatment	≤60 days	16%	0.29	Fixed	1.31 (1.19, 1.44)	5.48	<0.00001
	>60 days	0%	0.52	Fixed	1.31 (1.11, 1.54)	3.24	0.001
	Inter-subgroup	0%				0.00	0.99
Treatment Duration	≤30 days	0%	0.64	Random	1.25 (1.09, 1.42)	3.32	0.0009
	30–60 days	52%	0.07	Random	1.31 (1.06, 1.61)	2.46	0.01
	>60 days	0%	0.52	Random	1.30 (1.11, 1.52)	3.31	0.0009
	Inter-subgroup	0%				0.24	0.89
Treatment Duration	≤30 days	0%	0.64	Random	1.25 (1.09, 1.42)	3.32	0.0009
	30–45 days	72%	0.06	Random	1.78 (0.43, 7.43)	0.79	0.43
	45–60 days	54%	0.09	Random	1.30 (1.05, 1.60)	2.44	0.01
	>60 days	0%	0.52	Random	1.30 (1.11, 1.52)	3.31	0.0009
	Inter-subgroup	0%				0.43	0.93

Subtypes were categorized based on treatment duration into two distinct groups: ≤30 days (n = 4) vs >30 days (n = 9), ≤45 days (n = 6) vs >45 days (n = 7), and ≤60 days (n = 10) vs >60 days (n = 3). All subgroups exhibited low heterogeneity (I2 > 50%, P > 0.05), supporting the use of fixed-effects models for analysis. The results demonstrated that TCM interventions with durations of 30, 45, and 60 days significantly improved ECG efficacy compared to the control group.

Subsequently, studies were further divided into three duration-based groups: ≤30 days (*n* = 4), 30–60 days (*n* = 6), and >60 days (*n* = 3). Heterogeneity analysis revealed that the 30–60 days group showed moderate heterogeneity (*I* ^2^ = 52%, *P* = 0.07), while the ≤30 days (*I* ^2^ = 0%, *P* = 0.64) and >60 days (*I* ^2^ = 0%, *P* = 0.52) groups had low heterogeneity. Accordingly, a random-effects model was applied. The analysis indicated that TCM interventions significantly improved ECG efficacy across all duration groups: ≤30 days [RR = 1.25, 95% CI (1.09, 1.42), *P* = 0.0009], 30–60 days [RR = 1.31, 95% CI (1.06, 1.61), *P* = 0.01], and >60 days [RR = 1.30, 95% CI (1.11, 1.52), *P* = 0.0009]. To explore heterogeneity within the 30–60 days group, a subgroup analysis was performed, dividing it into 30–45 days (*n* = 2) (*I* ^2^ = 72%, *P* = 0.06) and 45–60 days (*n* = 4) (*I* ^2^ = 54%, *P* = 0.09). The random-effects model showed no significant improvement in ECG efficacy for the 30–45 days subgroup [RR = 1.78, 95% CI (0.43, 7.43), *P* = 0.43] (Table 4) (Supplementary Material S3).

These results suggest that TCM + BM intervention significantly improved ECG efficacy, whereas no significant difference was observed with TCM-alone. Shorter (≤30 days) and longer (>60 days) regimens show the most consistent benefits, whereas intermediate durations (30–45 days) require careful interpretation and further studies to elucidate their role.

#### 3.4.4 TCM syndrome score efficacy analysis

**FIGURE 12:**
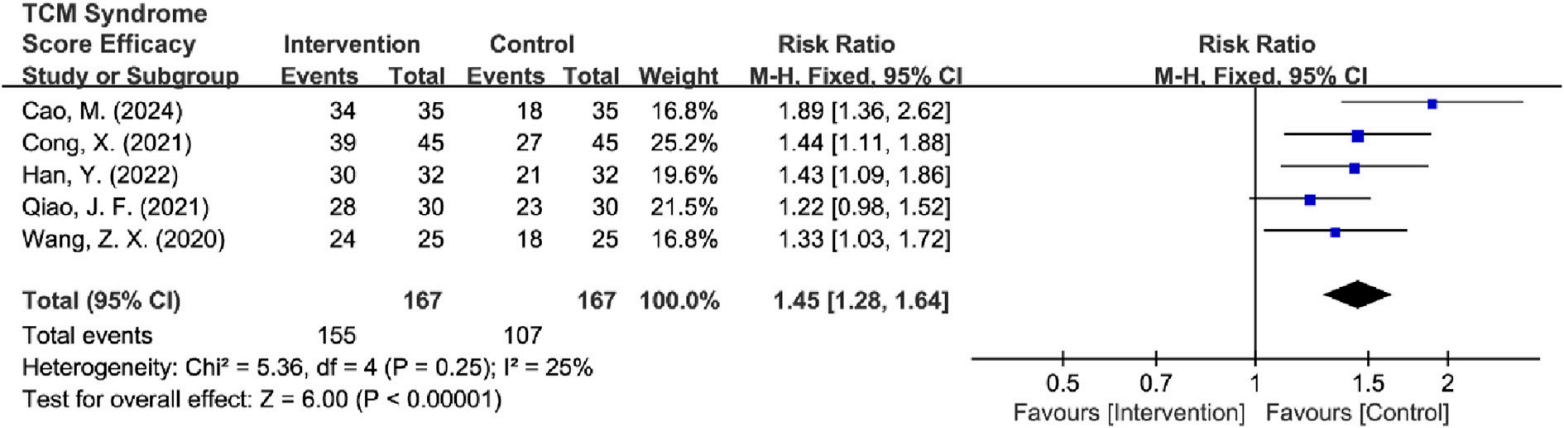
Forest plot of TCM syndrome score efficacy after excluding heterogeneous sources.

Among the 8 RCTs evaluating TCM syndrome scores (Wang and Han, 2020; Cong et al., 2021; Han et al., 2022; Cao et al., 2024; Chen et al., 2017; Qiao et al., 2021; Zhang et al., 2023; Zhong and Guo, 2019), 5 studies reported only effectiveness rates (Wang and Han, 2020; Cong et al., 2021; Cao et al., 2024; Qiao et al., 2021; Zhong and Guo, 2019), two studies provided only detailed score data (Chen et al., 2017; Zhang et al., 2023), and 1 study reported both metrics (Han et al., 2022). Due to the incompatible outcome reporting formats, these datasets were analyzed separately. The primary analysis focused on the 6 RCTs (Wang and Han, 2020; Cong et al., 2021; Han et al., 2022; Cao et al., 2024; Qiao et al., 2021; Zhong and Guo, 2019) that reported effectiveness rates. The initial assessment revealed substantial heterogeneity among studies [*I* ^2^ = 81%, *P* < 0.0001], so a random-effects model was used for subsequent analysis. The results showed significant improvement in TCM syndrome scores in the intervention group compared to the control group [RR = 1.34, 95% CI (1.09, 1.66), Z = 2.72, *P* = 0.006]. Sensitivity analysis identified Zhong and Guo (2019) as the primary source of heterogeneity as determined by leave-one-out analysis. The exclusion of this study resulted in a significant reduction in heterogeneity (*I* ^2^ = 25%, *P* = 0.25) and increased the precision of the effect estimate [RR = 1.45, 95% CI (1.28, 1.64), Z = 6.00, *P* < 0.00001] (Figure 12). The final analysis using a fixed-effects model demonstrated that TCM intervention significantly improved TCM syndrome scores in MB patients.

**FIGURE 13:**
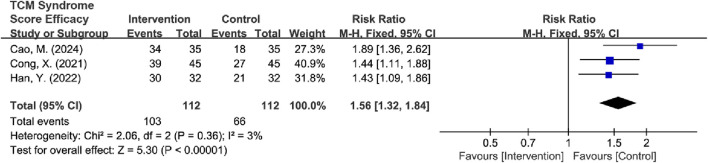
Forest plot of TCM syndrome score efficacy after excluding heterogeneous sources and high-risk articles.

Subsequently, high-risk studies by Wang and Han (2020) were removed for sensitivity analyses [*I* ^2^ = 85%, RR = 1.35, 95% CI (1.04, 1.75), Z = 6.72, *P* < 0.00001], and the results were consistent with previous ones. Sensitivity analysis revealed that Qiao et al. (2021) and Zhong and Guo (2019) were the main sources of heterogeneity. After removing high-risk articles and sources of heterogeneity, the results remained consistent with the previous findings [*I* ^2^ = 3%, RR = 1.56, 95% CI (1.32, 1.84), Z = 5.30, *P* < 0.00001] (Figure 13).

**FIGURE 14:**
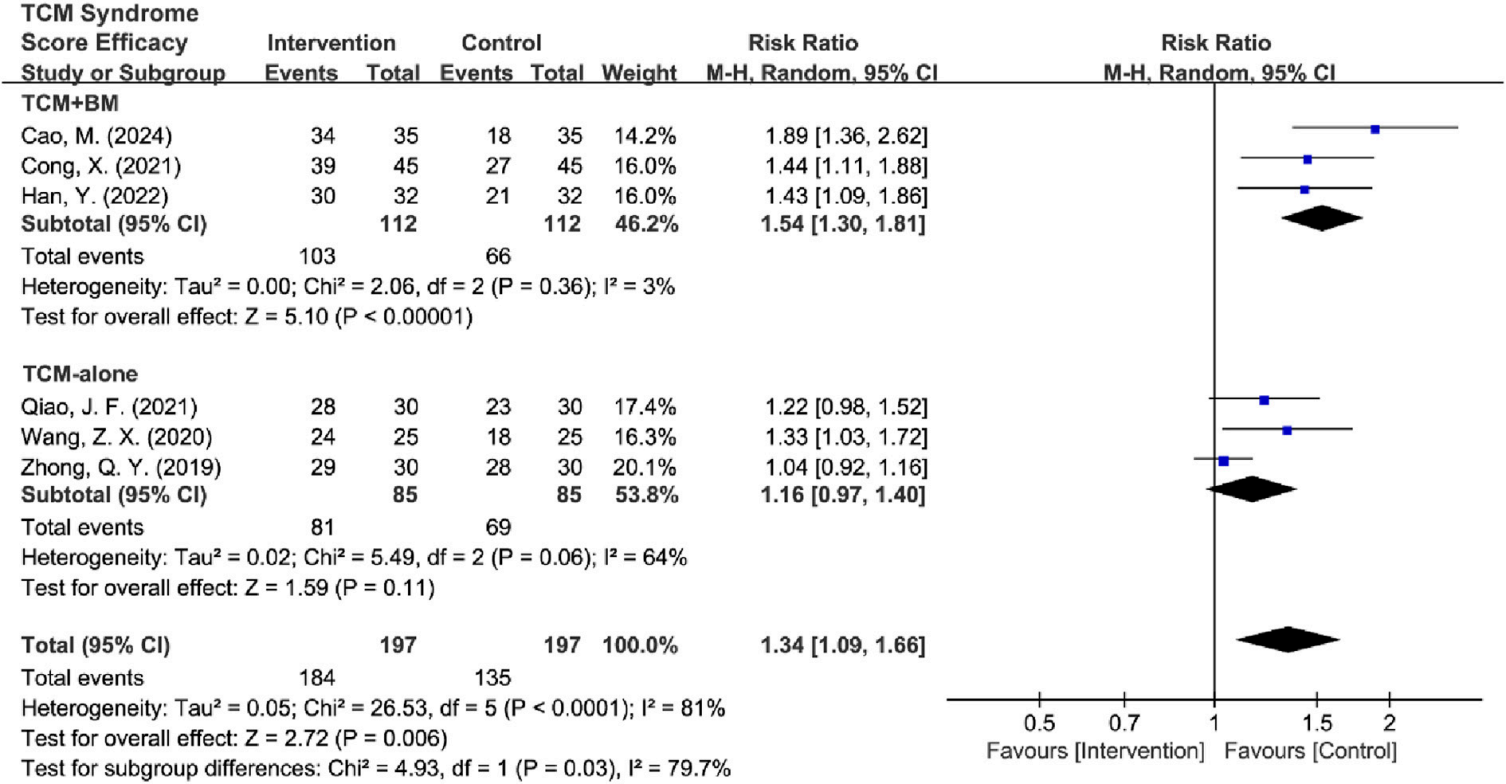
Forest plot of TCM syndrome score efficacy based on subgroups of interventions.

Subtypes were grouped into two intervention groups: TCM-alone (*n* = 3) and TCM + BM groups (*n* = 3). Heterogeneity was low in the TCM + BM group (*I* ^2^ = 3%, *P* = 0.36), whereas it was high in the TCM-alone group (*I* ^2^ = 64%, *P* = 0.06). Therefore, a random-effects model was used for analysis, and the results showed that compared to the control group, TCM + BM treatment significantly improved the TCM syndrome score [RR = 1.54, 95% CI (1.30, 1.81), Z = 5.10, *P* < 0.00001], whereas the improvement with TCM-alone [RR = 1.16, 95% CI (0.97, 1.40), Z = 1.59, *P* = 0.11] was not significant, with a significant difference between the two subgroups (*P* = 0.03) (Figure 14). Sensitivity analyses for removing high-risk articles reached consistent conclusions [TCM-alone: RR = 1.10, 95% CI (0.92, 1.32), Z = 1.04, *P =* 0.30; TCM + BM: RR = 1.54, 95% CI (1.30, 1.81), Z = 5.10, *P* < 0.00001].

**FIGURE 15:**
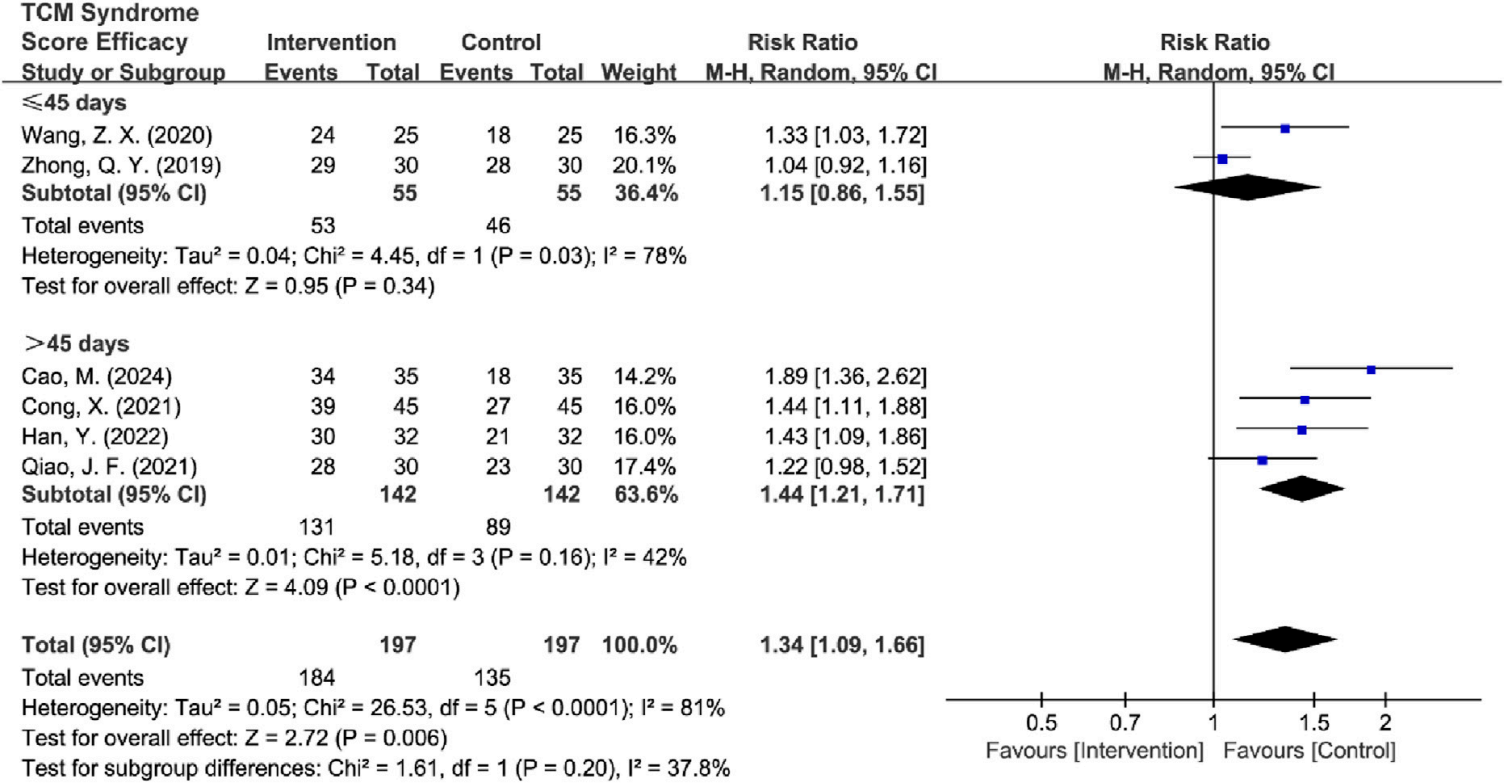
Forest plot of TCM syndrome score efficacy based on subgroups of treatment duration.

Subsequently, the studies were categorized into two groups based on treatment duration: ≤45 days (*n* = 2) and >45 days (*n* = 4). Both groups exhibited significant heterogeneity (for ≤45 days: *I* ^2^ = 78%, *P* = 0.03; for >45 days: *I* ^2^ = 42%, *P* = 0.16), which justified the use of the random-effects model. The analysis revealed that treatment durations exceeding 45 days significantly improved the TCM syndrome scores compared to the control group [RR = 1.44, 95% CI (1.21, 1.71), Z = 4.09, *P* < 0.0001], while no significant difference was observed for treatments lasting ≤45 days [RR = 1.15, 95% CI (0.86, 1.55), Z = 0.95, *P* = 0.34] (Figure 15; Table 5). After the removal of high-risk articles, there is only one RCT remaining in the group of ≤45 days, making it impossible to conduct a comparison; therefore, no exclusion will be made (Supplementary Material S3).

**TABLE 5:** Subgroup analysis of TCM syndrome score efficacy.

Difference	Subgroup	Heterogeneity test		Effect model	Meta analysis		
		*I* ^2^	*P*		RR (95% CI)	Z/*x* ^2^	*P*
Total	Intervention	81%	0.66	Random	1.34 (1.09, 1.66)	2.72	0.006
Sensitivity Analysis	Intervention	25%	0.25	Fixed	1.45 (1.28, 1.64)	6.00	<0.00001
Deletion of high-risk	Intervention	85%	<0.0001	Random	1.35 (1.04, 1.75)	2.28	0.02
Sensitivity Analysis	Intervention	3%	0.36	Fixed	1.56 (1.32, 1.84)	5.30	<0.00001
Interventions	TCM + BM	3%	0.36	Random	1.54 (1.30, 1.81)	5.10	<0.00001
	TCM-alone	64%	0.06	Random	1.16 (0.97, 1.40)	1.59	0.11
	Inter-subgroup	79.7%				4.93	0.03
Sensitivity Analysis	TCM + BM	3%	0.36	Random	1.54 (1.30, 1.81)	5.10	<0.00001
Deletion of high-risk	TCM-alone	55%	0.14	Random	1.10 (0.92, 1.32)	1.04	0.30
	Inter-subgroup	86.2%				7.25	0.007
45-day Treatment	≤45 days	78%	0.03	Random	1.15 (0.86, 1.55)	0.95	0.34
	>45 days	42%	0.16	Random	1.44 (1.21, 1.71)	4.09	<0.0001
	Inter-subgroup	37.8%				1.61	0.20

#### 3.4.5 SAQ efficacy analysis

**FIGURE 16:**
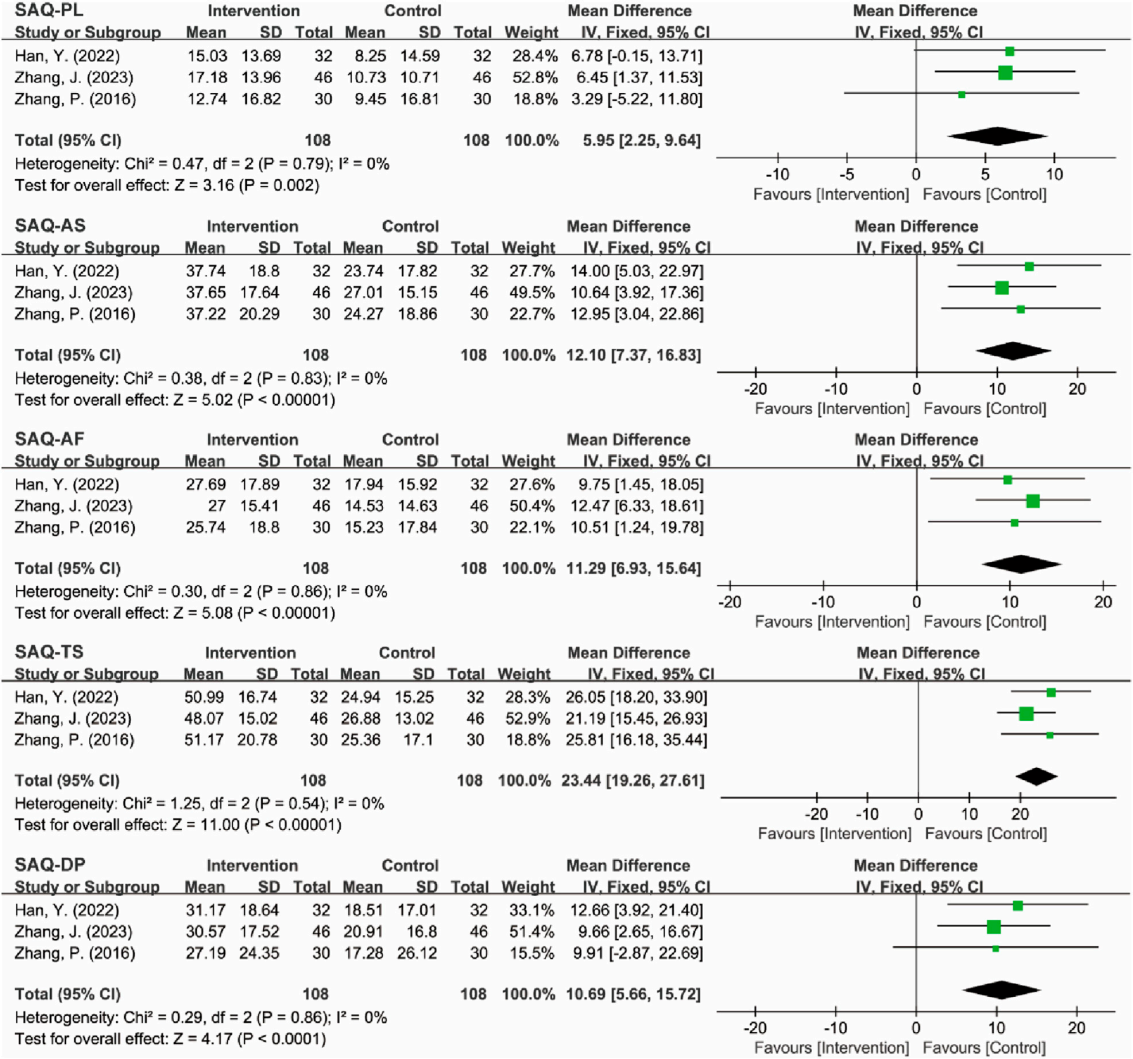
Forest plot of PL, AS, AF, TS, and DP in SAQ.

Three RCTs assessed the quality of life in MB patients using the SAQ, which evaluates five key dimensions: physical limitation (PL), angina stability (AS), angina frequency (AF), treatment satisfaction (TS), and disease perception (DP) (Wang J. G. et al., 2016; Yuan et al., 2018; Zhang et al., 2023). The analysis revealed low heterogeneity across all dimensions (*I* ^2^ = 0%), which justified the application of a fixed-effects model. The meta-analysis results demonstrated that TCM significantly outperformed the control group in enhancing all aspects of quality of life for patients with MB (Figure 16; Table 6).

**TABLE 6:** Results of heterogeneity tests and meta-analyses for SAQ, SAS, and SDS indicators.

Indicators	Heterogeneity test		Effect model	Meta analysis		
	*I* ^2^	*P*		MD (95% CI)	Z	*P*
Physical Limitation (PL)	0%	0.79	Fixed	5.95 (2.25, 9.64)	3.16	0.002
Angina Stability (AS)	0%	0.83	Fixed	12.10 (7.37, 16.83)	5.02	<0.00001
Angina Frequency (AF)	0%	0.86	Fixed	11.29 (6.93, 15.64)	5.08	<0.00001
Treatment Satisfaction (TS)	0%	0.54	Fixed	23.44 (19.26, 27.61)	11.00	<0.00001
Disease Perception (DP)	0%	0.86	Fixed	10.69 (5.66, 15.72)	4.17	<0.0001
SAS	0%	0.69	Fixed	−12.83 (−13.95, −11.71)	22.44	<0.00001
SDS	0%	0.88	Fixed	−6.97 (−8.41, −5.52)	9.45	<0.00001

#### 3.4.6 Psychological status assessment (SAS/SDS)

**FIGURE 17:**
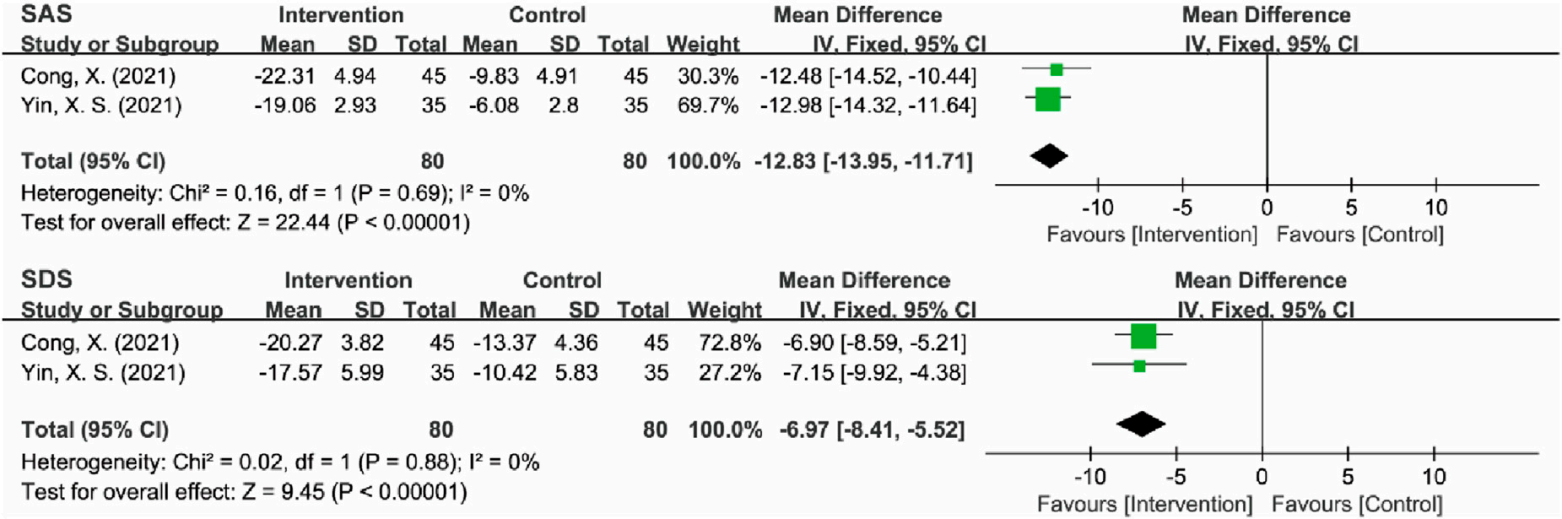
Forest plot of SAS and SDS.

Two RCTs assessed anxiety and depression status in MB patients using the SAS and SDS (Cong et al., 2021; Yin et al., 2021). Heterogeneity tests indicated low heterogeneity for both SAS (*I* ^2^ = 0%, P = 0.69) and SDS (*I* ^2^ = 0%, P = 0.88), supporting the use of a fixed-effects model for the analysis. The analysis revealed that the intervention group exhibited significantly lower SAS [MD = −12.83, 95% CI (−13.95, −11.71), Z = 22.44, *P* < 0.00001] and SDS [MD = −6.97, 95% CI (−8.41, −5.52), Z = 9.45, *P* < 0.00001] scores compared to the control group, effectively improving the anxiety and depression status of MB patients. Despite the statistically significant results, interpretation should be cautious due to the small number of included studies (*n* = 2), which limits the robustness of the conclusions. While the point estimates suggest strong effects, the precision is constrained by the sample size. Therefore, the results should be interpreted as preliminary and await replication in larger trials to confirm the findings (Figure 17; Table 6).

### 3.5 Adverse reactions

**FIGURE 18:**
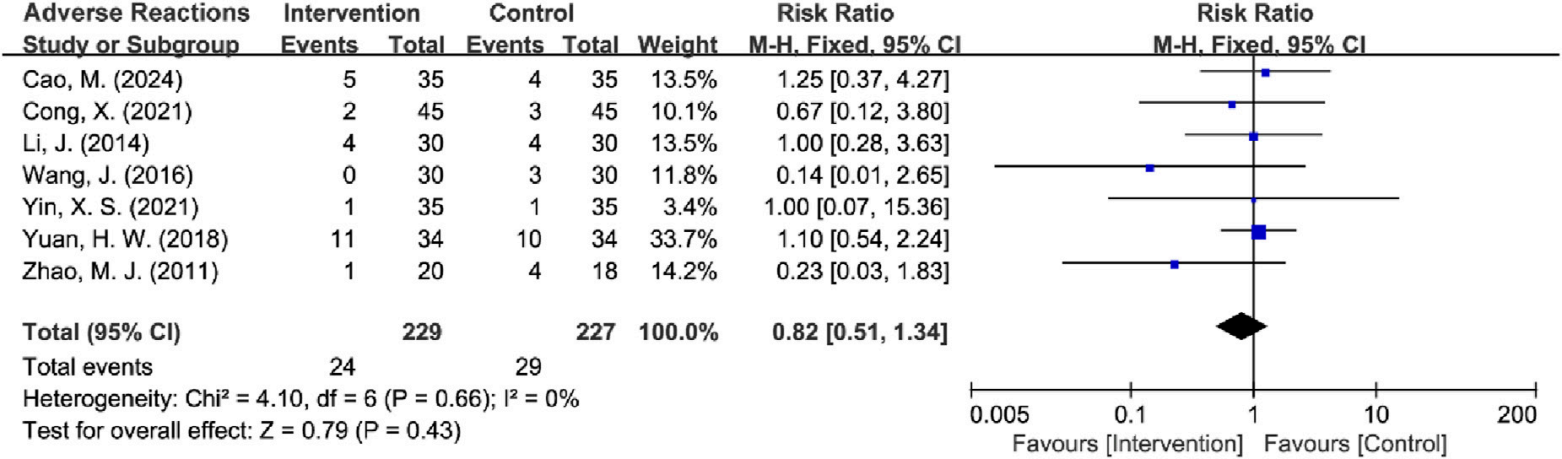
Forest plot of adverse reactions.

Seven RCTs compared adverse reactions between the two groups (Cong et al., 2021; Wang J. G. et al., 2016; Cao et al., 2024; Li, 2014; Yin et al., 2021; Yuan et al., 2018; Zhao et al., 2011). The heterogeneity test showed low between-study heterogeneity (*I* ^2^ = 0%, *P* = 0.66), and a fixed-effects model was used for analysis. The results indicated that the difference in adverse reactions between the two groups was not statistically significant [RR = 0.82, 95% CI (0.51, 1.34), Z = 0.79, *P* = 0.43], suggesting that TCM treatment for myocardial bridges may be safer relative to BM-alone. However, since the confidence interval crossed 1 and the difference was not significant, more studies are needed to verify the safety of TCM treatment for MB (Figure 18).

Additionally, subgroup analyses were conducted based on the interventions. However, due to the availability of only 1 RCT in the TCM-alone group, this subgroup was not performed. The studies were categorized into two groups based on treatment duration: ≤60 days (*n* = 3) and >60 days (*n* = 4). The >60 days group exhibited low heterogeneity (*I* ^2^ = 0%, *P* = 0.96), while the ≤60 days group demonstrated moderate heterogeneity (*I* ^2^ = 38%, *P* = 0.20). Both fixed-effects and random-effects models were applied, and the results indicated that neither the longer (>60 days) nor the shorter (≤60 days) treatment durations significantly increased adverse effects compared to the control group. Subsequent sensitivity analyses identified a study by Cao et al. (2024) as a significant source of heterogeneity within the ≤60 days group. After excluding this RCT, the heterogeneity was significantly reduced (*I* ^2^ = 0%, *P* = 0.80), and the analysis yielded consistent results but with a notably lower effect size in the ≤60 days group. This suggests that short-term use of TCM interventions may reduce the incidence of adverse reactions (Table 7) (Supplementary Material S3).

**TABLE 7:** Subgroup analysis of adverse reactions.

Difference	Subgroup	Heterogeneity test		Effect model	Meta analysis		
		*I* ^2^	*P*		RR (95% CI)	Z/*x* ^2^	*P*
Total	Intervention	0%	0.66	Fixed	0.82 (0.51, 1.34)	0.79	0.43
60-day Treatment	≤60 days	38%	0.20	Fixed	0.55 (0.22, 1.37)	1.28	0.20
	>60 days	0%	0.96	Fixed	1.00 (0.56, 1.78)	0.00	1.00
	Inter-subgroup	15%				1.18	0.28
60-day Treatment	≤60 days	38%	0.20	Random	0.50 (0.12, 2.09)	0.95	0.34
	>60 days	0%	0.96	Random	1.02 (0.57, 1.81)	0.06	0.95
	Inter-subgroup	0%				0.82	0.37
Sensitivity Analysis	≤60 days	0%	0.80	Random	0.19 (0.04, 1.06)	1.89	0.06
	>60 days	0%	0.96	Random	1.02 (0.57, 1.81)	0.06	0.95
	Inter-subgroup	69.6%				3.29	0.07

### 3.6 Descriptive analysis

Several outcome indicators, such as angina duration (Cao et al., 2024), Nobel classification (Wang J. G. et al., 2016), ischemic changes in ambulatory electrocardiogram (Zhang et al., 2016), exercise tolerance (Yin et al., 2021), plate exercise test indicator (Cong et al., 2021; Han et al., 2022; Zhang et al., 2023), carotid artery ultrasound indicator (Wang J. G. et al., 2016), blood lipid levels (Wang J. G. et al., 2016), and vascular endothelial function (Cao et al., 2024; Zhang et al., 2023), were reported in the literature but were not combined for a comprehensive analysis due to insufficient data or inconsistent reporting standards.

### 3.7 Publication bias analysis

**FIGURE 19:**
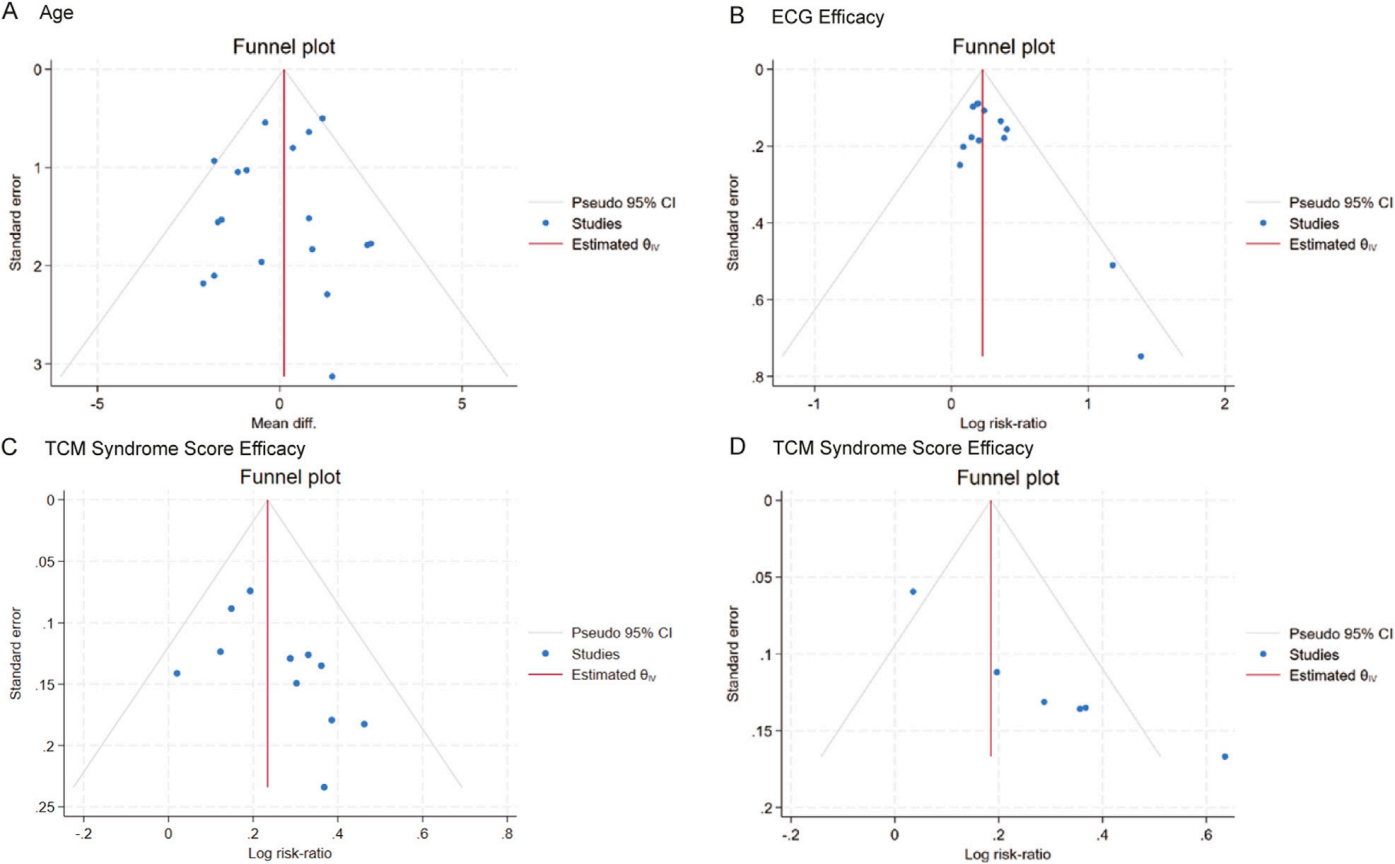
Funnel plot of publication bias. **(A–D)**: Funnel plots of **(A)** Age, **(B)** ECG efficacy, **(C)** angina efficacy, **(D)** TCM syndrome score.

Publication bias was assessed for indicators that contained 6 or more included studies, namely, age (18 RCTs), angina efficacy (14 RCTs), ECG efficacy (13 RCTs), and TCM syndrome score efficacy (6 RCTs). Funnel plots were constructed to visually inspect the presence of publication bias. The plots were tested for symmetry, and the Begg test was applied for age, angina efficacy, and ECG efficacy, while the Egger test was used for TCM symptom score efficacy. Comprehensive analysis of the funnel plots combined with the *P*-values of the tests indicated no publication bias for age (*P* = 0.8796 > 0.05) (Figure 19A), ECG efficacy (*P* = 0.2997 > 0.05) (Figure 19B), and angina efficacy (*P* = 0.0537 > 0.05) (Figure 19C). However, potential publication bias was detected for TCM syndrome score efficacy (*P* < 0.00001) (Figure 19D). Additionally, although angina efficacy (*P* = 0.0537) was greater than 0.05, there was still potential for publication bias.

**FIGURE 20:**
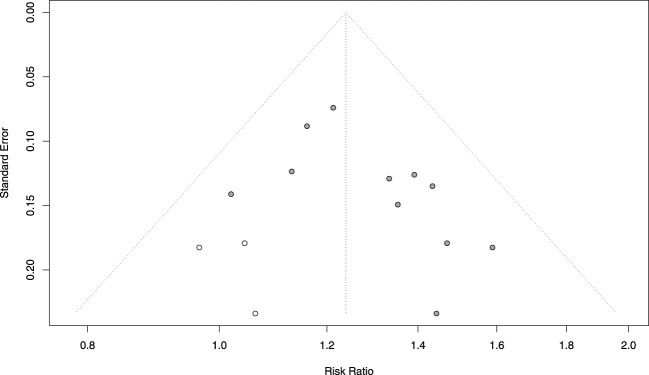
Funnel chart after Trim-and-fill of angina efficacy.

To address the potential publication bias issue regarding the angina efficacy and TCM syndrome score efficacy. Trim-and-fill analysis estimated three missing studies for angina efficacy (Figure 20). The adjusted effect size remained significant [RR = 1.24, 95% CI (1.16, 1.32) vs. original RR = 1.30], suggesting that the efficacy was overestimated by approximately 4.88% in the original analysis. For TCM syndrome score efficacy, the Bayesian publication bias correction model was applied, the improvement rate in the intervention group was significantly higher than that in the control group [RR = 1.26, 95% CI (1.07, 1.46) vs. original RR = 1.34], indicating that the efficacy was overestimated by approximately 6.35% in the original analysis. Despite these adjustments, the overall conclusion remains unchanged: TCM significantly improves angina efficacy and TCM syndrome score efficacy in patients with MB. The intervention group showed a significantly higher improvement rate compared to the control group, even after accounting for potential publication bias.

### 3.8 Medicinal substance properties

To delve deeper into the specific prescription patterns of medicinal substances for MB symptom management, this study analyzed 18 RCTs encompassing 18 distinct formulae. Two RCTs utilized Fuxin Heji Decoction employing differential dosages, and two RCTs used Modified Chaihu Shugan Powder with modified drug compositions. In alignment with the ConPhyMP guidelines, the formulae under investigation were classified accordingly: Six were designated as Class A (Shexiang Baoxin Pills, Xinkeshu Tablets, Naoxintong Capsules, Wide Chest Aerosol, Qishen Yiqi Dropping, Tongxinluo Capsules), defined as botanical drugs/extracts included in national pharmacopoeias; Nine were classified as Class B (Fuxin Heji Decoction, Liqi Huoxue Tongluo Formula, Huoxue Anshen Jieyu Decoction, Yiqi Tongmai Decoction, Yiqi Changmai Yin, Sanshen Sanqi Zhihu Granule, Shunqi Tongmai Capsules, Modified Chaihu Shugan Powder, Yingxinning Capsules), representing commercially available botanical drugs/extracts not listed in pharmacopoeias; and one was identified as a Class C (Hirudo and Kushen Powder Capsules), referring to preparations derived from understudied species without pharmacopoeial inclusion (Supplementary Material S4).

The frequency analysis indicated that across the 18 formulae, a total of 72 distinct medicinal substances were utilized, comprising 61 botanical drugs (84.72%), 9 zoological drugs (12.50%), and 2 mineral drugs (2.78%) (Supplementary Materials S5 and S6). Among botanical drugs, the most frequently used were *Salvia miltiorrhiza* Bunge [Lamiaceae; *Salviae miltiorrhizae radix et rhizoma*] present in 12 formulae (66.67%), *Ligusticum chuanxiong* Hort. [Apiaceae; *Chuanxiong rhizoma*] present in 11 formulae (61.11%), *Glycyrrhiza glabra* L. [Fabaceae; *Glycyrrhizae radix et rhizoma*] and *Angelica sinensis* (Oliv.) Diels [Apiaceae; *Angelicae sinensis radix*] each present in 7 formulae (38.89%), *Bupleurum chinense* DC. [Apiaceae; *Bupleuri radix*] present in 6 formulae (33.33%), *Astragalus mongholicus* Bunge [Fabaceae; *Astragali radix*], *Paeonia lactiflora* Pall. [Paeoniaceae; *Paeoniae radix alba*], and *Carthamus tinctorius* L. [Asteraceae; *Carthami flos*] each present in 5 formulae (27.78%), and *Santalum album* L. [Santalaceae; *Santali albi lignum*], *Panax notoginseng* (Burkill) F.H.Chen [Araliaceae; *Notoginseng radix et rhizoma*], and *P. lactiflora* Pall. [Paeoniaceae; *P. radix rubra*] each present in 4 formulae (22.22%). Among zoological drugs, *Hirudo nipponica* Whitman [Hirudinidae; *Hirudo*] appears in 4 formulae (22.22%), and *Mesobuthus martensii* Karsch [Buthidae; *Scorpio*] appears in 2 formulae. Mineral drugs included *Dens Draconis* (Fossilized Mammal Tooth, *Longchi*) and *Succinum* (Fossilized Resin, *Hupo*) each appearing in 1 formula.

Analysis of medicinal properties showed that 37 of the 72 medicinal substances were classified as having Warm/Heat in nature, 23 as Cold/Cool in nature, and 12 as neutral in nature. Primary therapeutic effects included 15 kinds of tonic medicines, 14 kinds of blood-activating and stasis-resolving medicines, and 13 kinds of Qi-regulating medicines (Table 8).

**TABLE 8:** Characteristics of high-frequency medicinal substances (≥4 times).

Efficacy	NO.	Medicinal substances	Natures	Frequency
Promoting Blood Circulation and Removing Blood Stasis Medicines	1	*Salvia miltiorrhiza* Bunge [Lamiaceae; *Salviae miltiorrhizae radix et rhizoma*]	Cold	12 (66.671%)
	2	*Ligusticum chuanxiong* Hort. [Apiaceae; *Chuanxiong rhizoma*]	Warm	11 (61.11%)
	3	*Carthamus tinctorius* L. [Asteraceae; *Carthami flos*]	Warm	5 (27.78%)
	4	*Paeonia lactiflora* Pall. [Paeoniaceae; *Paeoniae radix alba*]	Cold	5 (27.78%)
	5	*Panax notoginseng* (Burkill) F.H.Chen [Araliaceae; *Notoginseng radix et rhizoma*]	Warm	4 (22.22%)
	6	*Paeonia lactiflora* Pall. [Paeoniaceae; *Paeoniae radix rubra*]	Cold	4 (22.22%)
	7	*Hirudo nipponica* Whitman [Hirudinidae; *Hirudo*]	Neutral	4 (22.22%)
Tonic Medicines	8	*Glycyrrhiza glabra* L. [Fabaceae; *Glycyrrhizae radix et rhizoma*]	Neutral	7 (38.89%)
	9	*Angelica sinensis* (Oliv.) Diels [Apiaceae; *Angelicae sinensis radix*]	Warm	7 (38.89%)
	10	*Astragalus mongholicus* Bunge [Fabaceae; *Astragali radix*]	Warm	5 (27.78%)
Qi Regulating Medicines	11	*Bupleurum chinense* DC. [Apiaceae; *Bupleuri radix*]	Cold	6 (33.33%)
	12	*Santalum album* L. [Santalaceae; *Santali albi lignum*]	Warm	4 (22.22%)

Subsequently, an association rule analysis was performed on high-frequency medicinal substances with the objective of identifying medicinal substances combination rules. This analysis focused on two critical metrics: support and confidence. Support denotes the proportion of occurrences where both the antecedent and consequent items appear together within all possible item combinations. Confidence, on the other hand, indicates the proportion of transactions containing the antecedent item that also include the consequent item. The analysis revealed that *Salvia miltiorrhiza* Bunge [Lamiaceae; *Salviae miltiorrhizae radix et rhizoma*] and *Ligusticum chuanxiong* Hort. [Apiaceae; *Chuanxiong rhizoma*] exhibited high levels of support (61.111%) and confidence (81.818%). Specifically, the likelihood of a prescription containing *S. miltiorrhiza* Bunge [Lamiaceae; *Salviae miltiorrhizae radix et rhizoma*] given that it includes *L. chuanxiong* Hort. [Apiaceae; *Chuanxiong rhizoma*] was found to be 81.818% (Table 9).

**TABLE 9:** Association rule analysis of high-frequency medicinal substances (≥4 times).

Consequent	Antecedent	Support	Confidence
Salvia miltiorrhiza Bunge [Lamiaceae; Salviae miltiorrhizae radix et rhizoma]	Ligusticum chuanxiong Hort. [Apiaceae; Chuanxiong rhizoma]	61.111%	81.818%
Ligusticum chuanxiong Hort. [Apiaceae; Chuanxiong rhizoma]	Angelica sinensis (Oliv.) Diels [Apiaceae; Angelicae sinensis radix]	38.889%	100%
Ligusticum chuanxiong Hort. [Apiaceae; Chuanxiong rhizoma]	Glycyrrhiza glabra L. [Fabaceae; Glycyrrhizae radix et rhizoma]	38.889%	100%
Bupleurum chinense DC. [Apiaceae; Bupleuri radix]	Glycyrrhiza glabra L. [Fabaceae; Glycyrrhizae radix et rhizoma]	38.889%	85.714%
Salvia miltiorrhiza Bunge [Lamiaceae; Salviae miltiorrhizae radix et rhizoma]	Angelica sinensis (Oliv.) Diels [Apiaceae; Angelicae sinensis radix]	38.889%	85.714%
Salvia miltiorrhiza Bunge [Lamiaceae; Salviae miltiorrhizae radix et rhizoma]	Glycyrrhiza glabra L. [Fabaceae; Glycyrrhizae radix et rhizoma]	38.889%	85.714%
Bupleurum chinense DC. [Apiaceae; Bupleuri radix]	Glycyrrhiza glabra L. [Fabaceae; Glycyrrhizae radix et rhizoma] and Ligusticum chuanxiong Hort. [Apiaceae; Chuanxiong rhizoma]	38.889%	85.714%
Salvia miltiorrhiza Bunge [Lamiaceae; Salviae miltiorrhizae radix et rhizoma]	Angelica sinensis (Oliv.) Diels [Apiaceae; Angelicae sinensis radix] and Ligusticum chuanxiong Hort. [Apiaceae; Chuanxiong rhizoma]	38.889%	85.714%
Salvia miltiorrhiza Bunge [Lamiaceae; Salviae miltiorrhizae radix et rhizoma]	Glycyrrhiza glabra L. [Fabaceae; Glycyrrhizae radix et rhizoma] and Ligusticum chuanxiong Hort. [Apiaceae; Chuanxiong rhizoma]	38.889%	85.714%
Glycyrrhiza glabra L. [Fabaceae; Glycyrrhizae radix et rhizoma]	Bupleurum chinense DC. [Apiaceae; Bupleuri radix]	33.333%	100%
Ligusticum chuanxiong Hort. [Apiaceae; Chuanxiong rhizoma]	Bupleurum chinense DC. [Apiaceae; Bupleuri radix]	33.333%	100%
Paeonia lactiflora Pall. [Paeoniaceae; Paeoniae radix alba]	Bupleurum chinense DC. [Apiaceae; Bupleuri radix]	33.333%	83.333%
Ligusticum chuanxiong Hort. [Apiaceae; Chuanxiong rhizoma]	Bupleurum chinense DC. [Apiaceae; Bupleuri radix] and Glycyrrhiza glabra L. [Fabaceae; Glycyrrhizae radix et rhizoma]	33.333%	100%
Glycyrrhiza glabra L. [Fabaceae; Glycyrrhizae radix et rhizoma]	Bupleurum chinense DC. [Apiaceae; Bupleuri radix] and Ligusticum chuanxiong Hort. [Apiaceae; Chuanxiong rhizoma]	33.333%	100%
Ligusticum chuanxiong Hort. [Apiaceae; Chuanxiong rhizoma]	Angelica sinensis (Oliv.) Diels [Apiaceae; Angelicae sinensis radix] and Salvia miltiorrhiza Bunge [Lamiaceae; Salviae miltiorrhizae radix et rhizoma]	33.333%	100%
Ligusticum chuanxiong Hort. [Apiaceae; Chuanxiong rhizoma]	Glycyrrhiza glabra L. [Fabaceae; Glycyrrhizae radix et rhizoma] and Salvia miltiorrhiza Bunge [Lamiaceae; Salviae miltiorrhizae radix et rhizoma]	33.333%	100%
Salvia miltiorrhiza Bunge [Lamiaceae; Salviae miltiorrhizae radix et rhizoma]	Bupleurum chinense DC. [Apiaceae; Bupleuri radix]	33.333%	83.333%
Paeonia lactiflora Pall. [Paeoniaceae; Paeoniae radix alba]	Bupleurum chinense DC. [Apiaceae; Bupleuri radix] and Glycyrrhiza glabra L. [Fabaceae; Glycyrrhizae radix et rhizoma]	33.333%	83.333%
Paeonia lactiflora Pall. [Paeoniaceae; Paeoniae radix alba]	Bupleurum chinense DC. [Apiaceae; Bupleuri radix] and Ligusticum chuanxiong Hort. [Apiaceae; Chuanxiong rhizoma]	33.333%	83.333%
Paeonia lactiflora Pall. [Paeoniaceae; Paeoniae radix alba]	Glycyrrhiza glabra L. [Fabaceae; Glycyrrhizae radix et rhizoma] and Salvia miltiorrhiza Bunge [Lamiaceae; Salviae miltiorrhizae radix et rhizoma]	33.333%	83.333%
Salvia miltiorrhiza Bunge [Lamiaceae; Salviae miltiorrhizae radix et rhizoma]	Bupleurum chinense DC. [Apiaceae; Bupleuri radix] and Glycyrrhiza glabra L. [Fabaceae; Glycyrrhizae radix et rhizoma]	33.333%	83.333%
Bupleurum chinense DC. [Apiaceae; Bupleuri radix]	Glycyrrhiza glabra L. [Fabaceae; Glycyrrhizae radix et rhizoma] and Salvia miltiorrhiza Bunge [Lamiaceae; Salviae miltiorrhizae radix et rhizoma]	33.333%	83.333%
Salvia miltiorrhiza Bunge [Lamiaceae; Salviae miltiorrhizae radix et rhizoma]	Bupleurum chinense DC. [Apiaceae; Bupleuri radix] and Ligusticum chuanxiong Hort. [Apiaceae; Chuanxiong rhizoma]	33.333%	83.333%
Paeonia lactiflora Pall. [Paeoniaceae; Paeoniae radix alba]	Bupleurum chinense DC. [Apiaceae; Bupleuri radix] and Glycyrrhiza glabra L. [Fabaceae; Glycyrrhizae radix et rhizoma] and Ligusticum chuanxiong Hort. [Apiaceae; Chuanxiong rhizoma]	33.333%	83.333%
Paeonia lactiflora Pall. [Paeoniaceae; Paeoniae radix alba]	Glycyrrhiza glabra L. [Fabaceae; Glycyrrhizae radix et rhizoma] and Ligusticum chuanxiong Hort. [Apiaceae; Chuanxiong rhizoma] and Salvia miltiorrhiza Bunge [Lamiaceae; Salviae miltiorrhizae radix et rhizoma]	33.333%	83.333%
Salvia miltiorrhiza Bunge [Lamiaceae; Salviae miltiorrhizae radix et rhizoma]	Bupleurum chinense DC. [Apiaceae; Bupleuri radix] and Glycyrrhiza glabra L. [Fabaceae; Glycyrrhizae radix et rhizoma] and Ligusticum chuanxiong Hort. [Apiaceae; Chuanxiong rhizoma]	33.333%	83.333%
Bupleurum chinense DC. [Apiaceae; Bupleuri radix]	Glycyrrhiza glabra L. [Fabaceae; Glycyrrhizae radix et rhizoma] and Ligusticum chuanxiong Hort. [Apiaceae; Chuanxiong rhizoma] and Salvia miltiorrhiza Bunge [Lamiaceae; Salviae miltiorrhizae radix et rhizoma]	33.333%	83.333%

**FIGURE 21:**
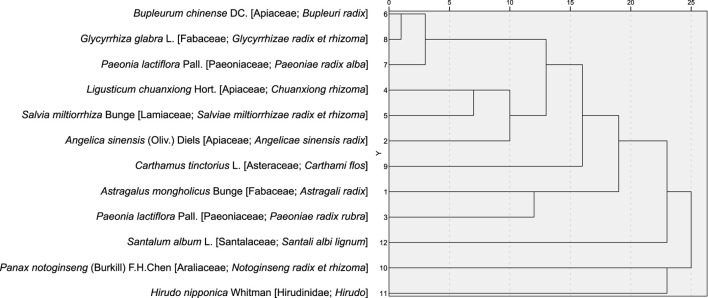
Cluster analysis of high frequency medicinal substances.

Cluster analysis was performed on the 12 medicinal substances occurring in ≥4 formulae, with results visualized through a dendrogram. Applying the elbow method, two discrete clusters were identified at a threshold of 11 linkage distance units. Cluster 1 comprised *Glycyrrhiza glabra* L. [Fabaceae; *Glycyrrhizae radix et rhizoma*], *Bupleurum chinense* DC. [Apiaceae; *Bupleuri radix*], and *Paeonia lactiflora* Pall. [Paeoniaceae; *Paeoniae radix alba*] (appearing in 27.78%–38.89% of formulae), while Cluster 2 contained *Salvia miltiorrhiza* Bunge [Lamiaceae; *Salviae miltiorrhizae radix et rhizoma*], *Ligusticum chuanxiong* Hort. [Apiaceae; *Chuanxiong rhizoma*], and *Angelica sinensis* (Oliv.) Diels [Apiaceae; *Angelicae sinensis radix*] (appearing in 61.11%–66.67% of formulae) (Figure 21).

The results demonstrate that *Salvia miltiorrhiza* Bunge [Lamiaceae; *Salviae miltiorrhizae radix et rhizoma*] and *Ligusticum chuanxiong* Hort. [Apiaceae; *Chuanxiong rhizoma*] are the primary medicinal substances for MB symptom management; Medicinal substances with Warm/Heat properties dominate the prescription profile; Core therapeutic actions focus on tonifying deficiencies and activating blood circulation to resolve stasis; and the combination of Salviae-Ligustici-Angelicae, comprising *S. miltiorrhiza* Bunge, *L. chuanxiong* Hort., and *Angelica sinensis* (Oliv.) Diels, and *Bupleurum-Paeonia-Glycyrrhiza,* comprising *Bupleurum chinense* DC. [Apiaceae; *Bupleuri radix*], *Paeonia lactiflora* Pall. [Paeoniaceae; *Paeoniae radix alba*], and *Glycyrrhiza glabra* L. [Fabaceae; *Glycyrrhizae radix et rhizoma*], is identified as the core therapeutic protocol for MB symptom management.

## 4 Discussion

MB are frequently overlooked, yet they represent a significant potential cause of angina symptoms in patients with nonobstructive CAD. Importantly, myocardial bridges are often undetectable by routine invasive coronary angiography, which may contribute to their underdiagnosis (Pargaonkar et al., 2021). Recent studies have elucidated the role of myocardial bridge anatomy in cardiovascular pathology. Specifically, the thickness, length, and location of myocardial bridges can significantly influence the risk of myocardial infarction. These anatomical characteristics promote the natural progression of atherosclerosis and exacerbate its aggregation at specific sites in the LAD artery segments proximal to the myocardial bridge, not only accelerating atherosclerotic changes but also increasing the likelihood of plaque rupture and thrombosis (Ishikawa et al., 2009). Clinically, myocardial bridges have been implicated in the induction of acute coronary syndrome (ACS), further highlighting their potential to trigger adverse cardiovascular events (Kwan and Singh, 2022; Bian et al., 2024). Moreover, emerging evidence suggests a strong association between myocardial bridges and endothelial dysfunction, particularly in elderly individuals with atherosclerotic risk factors. In these patients, endothelial dysfunction is frequently observed in the coronary artery segments where myocardial bridges are present (Ajmal et al., 2025). Additionally, myocardial bridges are strongly correlated with epicardial and microvascular endothelial dysfunction in patients presenting with chest pain and nonobstructive CAD. This correlation is believed to further elevate the risk of adverse cardiovascular events (Sara et al., 2020). Given the significant clinical implications of myocardial bridges, the development of effective therapeutic strategies to manage this condition is of paramount importance. Future research should focus on elucidating the precise mechanisms underlying myocardial bridge-associated pathology and identifying targeted interventions to mitigate the associated cardiovascular risks.

### 4.1 Limitations and prospects of research

TCM is increasingly recognized as an adjunctive, complementary, or alternative treatment for cardiovascular diseases. To evaluate the potential of TCM in alleviating symptoms associated with MB, this study conducted a meta-analysis summarizing 18 included RCTs. While this meta-analysis suggests TCM’s promise in alleviating MB symptoms, several limitations demand cautious interpretation.

Methodological constraints threaten validity. All included RCTs exhibited high risk or unclear risk in allocation concealment and blinding of outcome assessors. Only two studies implemented single-blinding. These deficiencies may inflate effect estimates for subjective outcomes like TCM syndrome scores and angina efficacy, aligning with Cochrane evidence that unblinded studies overestimate effects by 29%–36% (Salazar et al., 2025; Hróbjartsson et al., 2012). While objective measures (e.g., ECG) are less susceptible to detection bias, the overall reliability is compromised. This study then utilized the Cochrane ROB 2.0 tool to conduct a more thorough assessment of the risk of bias. The findings indicated that two studies were rated as “high risk,” while the remaining 16 studies were rated as having “some concerns.” When comparing the ROB and ROB 2.0 tools in dental trials, approximately 33.3% of the studies were rated as “low risk” by both instruments. However, 37% of the studies that were rated as “low risk” by ROB were subsequently rated as “high risk” by ROB 2.0, and 14.6% of the studies initially rated as “high risk” by ROB were upgraded to “low risk” by ROB 2.0. This discrepancy arises because ROB 2.0 employs stricter criteria for evaluating the randomization process, the implementation of blinding, and the handling of dropouts (Viana et al., 2025). Consequently, this study performed a sensitivity analysis after excluding high-risk RCTs, and the results were consistent with the previous conclusions. Nevertheless, the overall quality of the RCTs included in this study was suboptimal, with the majority being categorized as having “some concerns.” Future trials must adhere to CONSORT guidelines with centralized randomization and blinded endpoint adjudication.

Heterogeneity patterns reveal critical context. TCM monotherapy showed no significant benefit for ECG (*P* = 0.10) or TCM syndrome scores (*P* = 0.11), with substantial heterogeneity (*I* ^2^ > 60%). This stems from Uncontrolled prescription diversity and Methodological flaws. 0/7 reported allocation concealment or blinding (vs 2/11 in TCM + BM group), which affected the reliability of the results. In addition, TCM treatment is based on syndrome differentiation and the concept of holism, which involves adjusting prescriptions according to individual patient characteristics, including constitution, climate, geography, and specific symptoms. These individualized adjustments may have led to high heterogeneity due to the diverse TCM prescriptions used. By contrast, TCM + BM demonstrated consistent efficacy, when combined with biomedicine, TCM significantly improved angina symptoms, ECG parameters, and TCM syndrome scores, suggesting TCM’s efficacy for MB symptoms depends on synergistic use with standard biomedicine, rather than as standalone therapy.

Publication bias compromises angina and TCM syndrome score findings. Publication bias test indicated bias for angina efficacy (*P* = 0.0537) and TCM scores (*P* < 0.00001). To mitigate overestimation, the Trim-and-fill analysis estimated 3 missing studies for angina outcomes; adjusted RR remained significant [RR = 1.24, 95% CI (1.15, 1.32)]. For TCM syndrome scores, only six RCTs were available, which precluded the application of Trim-and-fill or deletion of small samples. Consequently, a Bayesian model was utilized to address the bias, yielding results consistent with previous findings [RR = 1.26, 95% CI (1.07,1.46)]. This suggests observed biases likely overestimate true effects by 5%–10%, possibly due to Underpowered studies with extreme effects in small samples. Despite these adjustments, the overall conclusion remains that TCM significantly improves angina efficacy and TCM syndrome scores in patients with MB. Therefore, more well-designed RCTs with larger sample sizes are needed in future research to further validate these findings, future RCTs should register protocols (e.g., WHO ICTRP) to minimize publication bias.

Sparse outcome reporting limits clinical insights. Angina pectoris is a common symptom of MB, and the SAQ is a specific and important scale for assessing the functional status of angina. Additionally, somatic symptom disorders such as anxiety and depression are also common in MB patients (Tao et al., 2025), with the SAS/SDS being commonly used to evaluate these symptoms. Although angina frequency reduction (MD = −0.96/week) demonstrates symptom control, the scarcity of SAQ data (3/18 studies) precludes assessment of functional recovery-a core treatment goal for symptomatic MB. Concurrently, the neglect of psychological metrics (SAS/SDS in only two studies) obscures TCM’s potential role in mitigating MB-related anxiety. Therefore, it is strongly recommended that core outcome sets (e.g., SAQ, SAS/SDS) be adopted in future MB trials to standardize efficacy evaluation criteria and psychological assessment of severely injured patients.

Subgroup analyses were underpowered. Non-significant results in the 30–45-day subgroup likely reflect type II error rather than true inefficacy, compared to robust effects in 45–60 days and ≥60-day groups.

### 4.2 Potential medicinal substances for TCM intervention in MB

Upon further analysis of the prescriptions used to alleviate MB symptoms, *S. miltiorrhiza* Bunge [Lamiaceae; *Salviae miltiorrhizae radix et rhizoma*] and *L. chuanxiong* Hort. [Apiaceae; *Chuanxiong rhizoma*] have emerged as particularly significant medicinal substances. *Salvia miltiorrhiza* Bunge [Lamiaceae; *Salviae miltiorrhizae radix et rhizoma*], often referred to as the “golden herb” in cardiovascular treatment, exerts its therapeutic effects through multiple mechanisms (Li et al., 2018). It targets various tissues and signaling pathways to inhibit endothelial inflammation and atherosclerotic plaque formation, modulate immune responses, regulate lipid metabolism, and adjust gut microbiota (Li et al., 2018; Ma et al., 2023; Tang and Zhao, 2024). Currently, several *S. miltiorrhiza* Bunge [Lamiaceae; *Salviae miltiorrhizae radix et rhizoma*]-based injections, including Danhong Injection, Danshen Injection, Danshen Ligustrazine Injection, Shenxiong Glucose Injection, and Danshen-Chuanxiongqin Injection, are widely used in the clinical treatment of cardiovascular and cerebrovascular diseases (Huajuan et al., 2023). A recent meta-analysis has further confirmed that Danshen class injections significantly improve inflammation and oxidative stress, demonstrating notable efficacy in the treatment of coronary heart disease (Dai et al., 2024).

*Ligusticum chuanxiong* Hort. [Apiaceae; *Chuanxiong rhizoma*], another botanical drug known for its qi-regulating and blood-activating properties, has also been extensively studied. Research indicates that *L. chuanxiong* Hort. [Apiaceae; *Chuanxiong rhizoma*] exerts significant therapeutic effects in cardiovascular diseases through its antiplatelet activity, endothelial cell protection, anti-inflammatory, antioxidant, and anti-apoptotic properties (Wang et al., 2025). Additionally, it has been shown to promote angiogenesis both *in vivo* and *in vitro* by modulating the PI3K/AKT/Ras/MAPK signaling pathway (Cheng et al., 2023).

The combination of *S. miltiorrhiza* Bunge [Lamiaceae; *Salviae miltiorrhizae radix et rhizoma*] and *L. chuanxiong* Hort. [Apiaceae; *Chuanxiong rhizoma*] is a commonly used medicinal substance pair (Chen et al., 2025). Studies have shown that this combination reduces the protein expression of TNF-α, IL-1β, and Cleaved-CASP3, while increasing the levels of p-AKT and Bcl2, thereby inhibiting apoptosis and significantly improving ischemic stroke (Pang et al., 2024). Furthermore, the trio of *S. miltiorrhiza* Bunge [Lamiaceae; *Salviae miltiorrhizae radix et rhizoma*], *L. chuanxiong* Hort. [Apiaceae; *Chuanxiong rhizoma*], and *Carthamus tinctorius* L. [Asteraceae; *Carthami flos*] has been shown to inhibit the expression of IL-6, IL-1β, and TNF-α. It also regulates hormones related to the hypothalamic-pituitary-adrenal axis, such as dopamine, norepinephrine, and 5-hydroxytryptamine, as well as the secretion of ghrelin and brain-derived neurotrophic factor. This combination significantly improves neurofunction and inflammatory responses in rats with traumatic brain injury (Zhang et al., 2025).

Based on the pairing of *S. miltiorrhiza* Bunge [Lamiaceae; *Salviae miltiorrhizae radix et rhizoma*] and *L. chuanxiong* Hort. [Apiaceae; *Chuanxiong rhizoma*], Danshen-Chuanxiong Injection and Shenxiong Glucose Injection have been recommended by Chinese expert consensus for the treatment of cardiovascular and cerebrovascular diseases (Gao et al., 2019). Research has shown that Shenxiong Glucose Injection inhibits oxidative stress and apoptosis, significantly improving isoproterenol-induced myocardial ischemia in rats and enhancing the function of HUVECs exposed to CoCl_2_ (Wu et al., 2022). Additionally, Danshen-Chuanxiong Injection has been shown to inhibit neuroinflammation by suppressing the TLR2/TLR4-MyD88-NF-κB signaling pathway, demonstrating significant therapeutic effects in tMCAO mice with ischemic stroke (Xu et al., 2021).

In addition, this study identified two key medicinal substance combinations, Salviae-Ligustici-Angelicae and *Bupleurum-Paeonia-Glycyrrhiza*, through cluster analysis. The combination of Salviae-Ligustici-Angelicae is a classic botanical drug pair known for promoting the circulation of Qi and blood. Research indicates that this botanical drug pair can stimulate the proliferation of microvascular endothelial cells derived from rats and increase vascularization to facilitate angiogenesis (Meng et al., 2006). Bupleurum-Paeonia-Glycyrrhiza is a typical classic botanical pair used for soothing the liver and relieving depression, frequently found in many classical formulas, especially SiNi Powder and ChaiHuShuGan Powder. Studies have shown that the botanical drug pair of *B. chinense* DC. [Apiaceae; *Bupleuri radix*] and *P. lactiflora* Pall. [Paeoniaceae; *P. radix alba*] is widely used to improve psychological disorders, particularly depression (Lv et al., 2022), possibly through the regulation of purine metabolism, inhibition of oxidative stress, and anti-depression effects on inflammatory responses in the cortex (Chen et al., 2022).

This study demonstrates that TCM holds significant potential in improving MB symptoms and quality of life. Specifically, the combination of TCM can significantly improve angina symptoms in MB patients, reduce the frequency of angina attacks, optimize ECG profiles, improve TCM syndrome scores, and effectively alleviate anxiety and depression, while also enhancing quality of life with a favorable safety profile. Subgroup analyses further reveal that the combination of TCM and conventional biomedicine shows remarkable efficacy in improving the aforementioned prognostic indicators. In contrast, TCM alone improves angina efficacy but has limited effects on ECG profiles and TCM syndrome scores. Additionally, the study finds that longer treatment durations (≥45 days) yield more significant therapeutic effects compared to shorter durations (<30 days). Given these findings, TCM’s efficacy for MB symptoms appears contingent on 1) combination with biomedicine; 2) standardized protocols minimizing prescription heterogeneity; and 3) adequate trial duration (≥45 days).

Despite these encouraging results, the study is not without its limitations: ① Methodological Quality Concerns: The primary concern is the suboptimal quality of the included RCTs, largely attributable to inadequate blinding procedures, lack of reported allocation concealment, and vague randomization methods (e.g., simply stated as ‘randomized’ without detailing the techniques used). ② Publication Bias in Subjective Outcomes: Although Bayesian models for publication bias correction were applied to TCM syndrome scores, the inherent subjectivity of these outcome measures remains a significant confounding factor. ③ Substantial Heterogeneity: Marked heterogeneity across studies undermined the stability of the results. While comprehensive sensitivity analyses verified the robustness of the efficacy findings, the conclusions are still influenced by the inclusion of lower-quality RCTs. ④ Control Group Limitations: All control groups utilized β-blockers or calcium channel blockers. Future research should conduct drug-class subgroup analyses (e.g., comparing β-blockers with calcium antagonists) and evaluate dose-response relationships. ⑤ Inconclusive Subgroup Analysis for TCM Protocols: Despite efforts to analyze TCM formulas through herb-function clusters and combination patterns, no statistically significant subgroups were identified. This reflects the methodological challenges associated with standardizing syndrome differentiation-based treatments for MB.

To address the aforementioned limitations, future research should: ① Enhance methodological rigor by implementing triple-blinding, explicit allocation concealment, and detailed randomization in RCTs; ② Reduce subjectivity bias through objective tools (e.g., AI-assisted TCM syndrome evaluation) replacing traditional scores; ③ Mitigate heterogeneity via strict participant stratification (e.g., by MB Nobel classification) and standardized interventions; ④ Optimize control groups with active comparators and drug-class/dose-response subgroup analyses; ⑤ Standardize TCM protocols by developing consensus-driven treatment algorithms balancing syndrome differentiation and reproducibility; ⑥Incorporate objective biomarkers such as coronary flow reserve and serum metabolomics profiles; ⑦Conduct large multicenter trials to ensure generalizability; and ⑧Extend follow-up durations (>24 months) to evaluate long-term efficacy/safety. Collectively, these advances will generate robust evidence for botanical drugs in MB management.

## Data Availability

The original contributions presented in the study are included in the article/Supplementary Material, further inquiries can be directed to the corresponding authors.

## Abbreviations

AS, Angina Stability; AF, Angina Frequency; CAD, Coronary Artery Disease; CI, Confidence Intervals (CI), DP, Disease Perception; ECG, Electrocardiogram; LAD, Left Anterior Descending; MB, Myocardial Bridge; MD, Mean Difference; MeSH, Medical Subject Headings; PL, Physical Limitation; RCTs, Randomized Controlled Trials; RR, Relative Risk; SAQ, Seattle Angina Questionnaire; SAS, Self-rating Anxiety Scale; SDS, Self-rating Depression Scale; TCM, Traditional Chinese Medicine; TS, Treatment Satisfaction.

## References

[B1] Ajmal M. Javed B. Kubba S. Singh K. Samady H. Lerman A. (2025). Contemporary review of myocardial bridging for internists. Am. J. Med. 138 (7), 1068–1073. 10.1016/j.amjmed.2025.02.025 40043868

[B2] Bian W. Wu Y. Wang B. (2024). Myocardial infarction associated with myocardial bridging: a case report. Asian J. Surg. S1015-9584 (24), 02847–02841. 10.1016/j.asjsur.2024.11.198 39668044

[B3] Cao M. Huang F. Yu Y. P. (2024). The effect of metoprolol succinate sustained-release tablets combined with fuxin mixture in the treatment of coronary artery myocardial bridging angina and its impact on vascular endothelial function. Med. Innov. China 21 (14), 48–52. 10.3969/j.issn.1674-4985.2024.14.012

[B4] Chen J. Chen L. F. Xu N. (2017). Clinical observation of self-prepared Liqi Huoxue Tongluo formula in the treatment of Qi stagnation and blood stasis type coronary artery myocardial bridge. Prev. Treat. Cardiovasc Dis. (8), 50–52. 10.3969/j.issn.1672-3015(x).2017.08.019

[B5] Chen J. Li T. Qin X. Du G. Zhou Y. (2022). Integration of non-targeted metabolomics and targeted quantitative analysis to elucidate the synergistic antidepressant effect of Bupleurum Chinense DC-Paeonia lactiflora pall herb pair by regulating purine metabolism. Front. Pharmacol. 13, 900459. 10.3389/fphar.2022.900459 35847012 PMC9280301

[B6] Chen Y. Lai F. Xu H. He Y. (2025). Chinese herb pairs for cardiovascular and cerebrovascular diseases: compatibility effects, pharmacological potential, clinical efficacy, and molecular mechanisms. J. Ethnopharmacol. 347, 119516. 10.1016/j.jep.2025.119516 39978448

[B7] Cheng X. H. Yang X. X. Cui H. R. Zhang B. B. Chen K. D. Yang X. Y. (2023). Chuanxiong improves angiogenesis *via* the PI3K/AKT/Ras/MAPK pathway based on network pharmacology and DESI-MSI metabolomics. Front. Pharmacol. 14, 1135264. 10.3389/fphar.2023.1135264 37214436 PMC10196038

[B8] Ciliberti G. Laborante R. Di Francesco M. Restivo A. Rizzo G. Galli M. (2022). Comprehensive functional and anatomic assessment of myocardial bridging: unlocking the Gordian Knot. Front. Cardiovasc Med. 9, 970422. 10.3389/fcvm.2022.970422 36426224 PMC9678929

[B9] Cong X. Wang L. Su Q. Q. Mu R. N. Li L. (2021). Effect of bisoprolol combined with Huoxue Anshen Jieyu decoction on treadmill exercise test and unhealthy emotion in patients with left anterior descending coronary artery myocardial bridge combined with anxiety and depression. Mod. J. Integr. Tradit. West Med. 30 (17), 1867–1870+1919. 10.3969/j.issn.1008-8849.2021.17.010

[B10] Corban M. T. Hung O. Y. Eshtehardi P. Rasoul-Arzrumly E. McDaniel M. Mekonnen G. (2014). Myocardial bridging: contemporary understanding of pathophysiology with implications for diagnostic and therapeutic strategies. J. Am. Coll. Cardiol. 63 (22), 2346–2355. 10.1016/j.jacc.2014.01.049 24583304 PMC4065198

[B11] Dai S. Ding Y. Guo J. Wang X. (2024). Efficacy and safety of danshen class injections in the treatment of coronary heart disease: a network meta-analysis. Front. Pharmacol. 15, 1487119. 10.3389/fphar.2024.1487119 39726778 PMC11669530

[B12] Fan G. H. He Y. X. Zou J. N. Chen X. J. (2012). Clinical observation of Shexiang Baoxin pill and metoprolol for treatment of coronary myocardial bridge. Chin. J. Integr. Med. Cardio Cerebrovasc. Dis. 10 (1), 3–4. 10.3969/j.issn.1672-1349.2012.01.002

[B13] Gao Y. Wang G. Q. Wang J. Zhang M. X. Xie Y. M. Liu H. (2019). Expert consensus statement on Danshen Chuanxiongqin injection in clinical practice. Zhongguo Zhong Yao Za Zhi 44 (14), 2937–2942. 10.19540/j.cnki.cjcmm.20190509.503 31602836

[B14] Guerra E. Bergamaschi L. Tuttolomondo D. Pizzi C. Sartorio D. Gaibazzi N. (2023). Contrast stress echocardiography findings in myocardial bridging compared to normal coronary course, with and without coronary artery disease. J. Am. Soc. Echocardiogr. 36 (10), 1092–1099. 10.1016/j.echo.2023.06.008 37356674

[B15] Han Y. Dai M. Liu H. X. Zhang D. W. Wei Z. Z. (2022). Clinical study of Yiqi-Tongmai Decoction on qi deficiency and blood stasis syndrome of isolated coronary artery muscle bridge angina pectoris. Int. J. Tradit. Chin. Med. 44 (1), 22–27. 10.3760/cma.j.cn115398-20210825-00299

[B16] Hao P. Jiang F. Cheng J. Ma L. Zhang Y. Zhao Y. (2017). Traditional Chinese medicine for cardiovascular disease: evidence and potential mechanisms. J. Am. Coll. Cardiol. 69 (24), 2952–2966. 10.1016/j.jacc.2017.04.041 28619197

[B17] Heinrich M. Jalil B. Abdel-Tawab M. Echeverria J. Kulić Ž. McGaw L. J. (2022). Best practice in the chemical characterisation of extracts used in pharmacological and toxicological research-The ConPhyMP-guidelines. Front. Pharmacol. 13, 953205. 10.3389/fphar.2022.953205 36176427 PMC9514875

[B18] Hostiuc S. Negoi I. Rusu M. C. Hostiuc M. (2018). Myocardial bridging: a meta-analysis of prevalence. J. Forensic Sci. 63 (4), 1176–1185. 10.1111/1556-4029.13665 29044562

[B19] Hozo S. P. Djulbegovic B. Hozo I. (2005). Estimating the mean and variance from the median, range, and the size of a sample. BMC Med. Res. Methodol. 5, 13. 10.1186/1471-2288-5-13 15840177 PMC1097734

[B20] Hróbjartsson A. Thomsen A. S. Emanuelsson F. Tendal B. Hilden J. Boutron I. (2012). Observer bias in randomised clinical trials with binary outcomes: systematic review of trials with both blinded and non-blinded outcome assessors. Bmj 344, e1119. 10.1136/bmj.e1119 22371859

[B21] Huajuan J. Xulong H. Bin X. Yue W. Yongfeng Z. Chaoxiang R. (2023). Chinese herbal injection for cardio-cerebrovascular disease: overview and challenges. Front. Pharmacol. 14, 1038906. 10.3389/fphar.2023.1038906 36909150 PMC9998719

[B22] Ishikawa Y. Akasaka Y. Suzuki K. Fujiwara M. Ogawa T. Yamazaki K. (2009). Anatomic properties of myocardial bridge predisposing to myocardial infarction. Circulation 120 (5), 376–383. 10.1161/circulationaha.108.820720 19620504

[B23] Kwan B. Singh A. (2022). Acute coronary syndrome caused by myocardial bridging. Am. J. Emerg. Med. 52, 272.e271–272.e273. 10.1016/j.ajem.2021.08.080 34629225

[B24] Li J. (2014). Clinical observation of xinkeshu tablets and metoprolol in the treatment of coronary artery myocardial bridge. Chin. J. Integr. Med. Cardio Cerebrovasc. Dis. 12 (10), 1185–1186. 10.3969/j.issn.16721349.2014.10.010

[B25] Li Z. M. Xu S. W. Liu P. Q. (2018). Salvia miltiorrhizaBurge (Danshen): a golden herbal medicine in cardiovascular therapeutics. Acta Pharmacol. Sin. 39 (5), 802–824. 10.1038/aps.2017.193 29698387 PMC5943903

[B26] Lv S. Zhao Y. Wang L. Yu Y. Li J. Huang Y. (2022). Antidepressant active components of Bupleurum chinense DC-Paeonia lactiflora pall herb pair: pharmacological mechanisms. Biomed. Res. Int. 2022, 1024693. 10.1155/2022/1024693 36408279 PMC9668458

[B27] Ma X. Zhang L. Gao F. Jia W. Li C. (2023). Salvia miltiorrhiza and Tanshinone IIA reduce endothelial inflammation and atherosclerotic plaque formation through inhibiting COX-2. Biomed. Pharmacother. 167, 115501. 10.1016/j.biopha.2023.115501 37713995

[B28] Matta A. Canitrot R. Nader V. Blanco S. Campelo-Parada F. Bouisset F. (2021). Left anterior descending myocardial bridge: angiographic prevalence and its association to atherosclerosis. Indian Heart J. 73 (4), 429–433. 10.1016/j.ihj.2021.01.018 34474753 PMC8424261

[B29] Meng H. Zhu M. Z. Guo J. Sun J. Y. Pei J. M. Huang C. (2006). The study on angiogenesis activity of Danggui, Chuanxiong and Danshen. Zhong Yao Cai 29 (6), 574–576. 10.13863/j.issn1001-4454.2006.06.022 17039881

[B30] Moore P. Murdock P. Ramanathan A. Sathyamoorthy M. (2023). A contemporary review of the genomic associations of coronary artery myocardial bridging. Genes (Basel) 14 (12), 2175. 10.3390/genes14122175 38136997 PMC10871102

[B31] Nie C. Zhu C. Yang Q. Xiao M. Meng Y. Wang S. (2021). Myocardial bridging of the left anterior descending coronary artery as a risk factor for atrial fibrillation in patients with hypertrophic obstructive cardiomyopathy: a matched case-control study. BMC Cardiovasc Disord. 21 (1), 382. 10.1186/s12872-021-02185-1 34362314 PMC8348797

[B32] Page M. J. McKenzie J. E. Bossuyt P. M. Boutron I. Hoffmann T. C. Mulrow C. D. (2021). The PRISMA 2020 statement: an updated guideline for reporting systematic reviews. BMJ 372, n71. 10.1136/bmj.n71 33782057 PMC8005924

[B33] Pang H. Q. Guo J. X. Yang Y. Xu L. Wang J. Yang F. (2024). Elucidating the chemical interaction effects of herb pair Danshen-Chuanxiong and its anti-ischemic stroke activities evaluation. J. Ethnopharmacol. 318 (Pt B), 117058. 10.1016/j.jep.2023.117058 37597675

[B34] Pargaonkar V. S. Kimura T. Kameda R. Tanaka S. Yamada R. Schwartz J. G. (2021). Invasive assessment of myocardial bridging in patients with angina and no obstructive coronary artery disease. EuroIntervention 16 (13), 1070–1078. 10.4244/eij-d-20-00779 33074153 PMC9725037

[B35] Qiao J. F. Zheng F. Li C. Y. Lin C. Wu X. (2021). Clinical observation of modified Yiqi changmai Yin in the treatment of deficiency of Qi and blood stasis type coronary artery myocardial Bridge. Prev. Treat. Cardiovasc Dis. 11 (26), 16–18. 10.3969/j.issn.1672-3015(x).2021.26.005

[B36] Reyman H. C. (1737). Dissertatio de vasis cordis propriis. Bibl. Anat. 2, 359–378.

[B37] Salazar J. Moustgaard H. Bracchiglione J. Hróbjartsson A. (2025). Empirical evidence of observer bias in randomized clinical trials: updated and expanded analysis of trials with both blinded and non-blinded outcome assessors. J. Clin. Epidemiol. 183, 111787. 10.1016/j.jclinepi.2025.111787 40258524

[B38] Sara J. D. S. Corban M. T. Prasad M. Prasad A. Gulati R. Lerman L. O. (2020). Prevalence of myocardial bridging associated with coronary endothelial dysfunction in patients with chest pain and non-obstructive coronary artery disease. EuroIntervention 15 (14), 1262–1268. 10.4244/eij-d-18-00920 30636680

[B39] Sternheim D. Power D. A. Samtani R. Kini A. Fuster V. Sharma S. (2021). Myocardial bridging: diagnosis, functional assessment, and management: JACC state-of-the-art review. J. Am. Coll. Cardiol. 78 (22), 2196–2212. 10.1016/j.jacc.2021.09.859 34823663

[B40] Tang J. Zhao X. (2024). Research progress on regulation of immune response by tanshinones and salvianolic acids of Danshen (*Salvia miltiorrhiza* bunge). Molecules 29 (6), 1201. 10.3390/molecules29061201 38542838 PMC10975292

[B41] Tao Z. Wu Y. Qiao Y. Wang Z. Chai Y. Wu Q. (2025). Somatic symptom disorder in patients with myocardial bridge: cross-sectional study in China. BJPsych Open 11 (2), e67. 10.1192/bjo.2024.851 40123454 PMC12001947

[B42] Viana J. Machado V. Proença L. Chambrone L. Mendes J. J. Botelho J. (2025). Comparative assessment of cochrane’s ROB and ROB2 in dentistry trials: a meta-research study. Syst. Rev. 14 (1), 154. 10.1186/s13643-025-02901-4 40722191 PMC12302898

[B43] Wang Z. X. Han Y. (2020). Evaluation of the effect of modified chaihu Shugan powder in the treatment of Qi stagnation and blood stasis type coronary artery myocardial bridge. Healthmust-Read Mag. (26), 68.

[B44] Wang J. G. Xi J. T. Zhao X. K. Zou Y. Zhang H. He J. (2016). Efficacy observation and prognostic analysis of Naoxintong capsules in the treatment of symptomatic coronary artery myocardial bridges combined with carotid atherosclerosis. Electron J. Clin. Med. Lit. 3 (21), 4302–4304. 10.16281/j.cnki.jocml.2016.21.123

[B45] Wang J. Xue J. M. Lv X. W. Zhang X. L. Wang L. R. Chen J. S. (2016). Clinical observation of leech and Kushen Powder capsules in the treatment of 60 cases of patients with Angina Pectoris due to coronary artery myocardial Bridge. Chin. J. Tradit. Med. Sci. Technol. 23 (4), 492–493.

[B46] Wang Y. Wu L. Wang H. Jiang M. Chen Y. Zheng X. (2025). Ligusticum chuanxiong: a chemical, pharmacological and clinical review. Front. Pharmacol. 16, 1523176. 10.3389/fphar.2025.1523176 40235541 PMC11996930

[B47] Wu Z. X. Chen S. S. Lu D. Y. Xue W. N. Sun J. Zheng L. (2022). Shenxiong glucose injection inhibits oxidative stress and apoptosis to ameliorate isoproterenol-induced myocardial ischemia in rats and improve the function of HUVECs exposed to CoCl(2). Front. Pharmacol. 13, 931811. 10.3389/fphar.2022.931811 36686658 PMC9849394

[B48] Xu X. J. Long J. B. Jin K. Y. Chen L. B. Lu X. Y. Fan X. H. (2021). Danshen-Chuanxiongqin Injection attenuates cerebral ischemic stroke by inhibiting neuroinflammation *via* the TLR2/TLR4-MyD88-NF-κB Pathway in tMCAO mice. Chin. J. Nat. Med. 19 (10), 772–783. 10.1016/s1875-5364(21)60083-3 34688467

[B49] Yang T. L. Hao W. R. Chen C. C. Fang Y. A. Leu H. B. Liu J. C. (2024). Myocardial bridging increases the risk of adverse cardiovascular events in patients without coronary atherosclerosis. Life (Basel) 14 (7), 811. 10.3390/life14070811 39063566 PMC11278439

[B50] Yin X. S. Zheng J. W. Zhang Y. H. Zhong L. L. Liu L. Y. Wang X. G. (2021). Clinical study of wide chest aerosol in the treatment of patients with coronary artery muscle bridge. Jilin Med. J. 42 (7), 1569–1571. 10.3969/j.issn.1004-0412.2021.07.009

[B51] Yuan H. W. Wang X. L. Li Z. Yang J. Y. Yuan J. Q. (2018). Clinical observation on Sanshen Sanqi Zhihu Granule and bisoprolol in the treatment of myocardial Bridge. Chin. J. Integr. Med. Cardio Cerebrovasc. Dis. 16 (6), 684–686. 10.3969/j.issn.1672-1349.2018.06.003

[B52] Zhang P. Zhang G. J. Zhang H. (2016). Efficacy study for Qishen Yiqi dropping pills in patients with myocardial bridge and angina pectoris. Tianjin J. Tradit. Chin. Med. 33 (4), 208–212. 10.11656/j.issn.1672-1519.2016.04.05

[B53] Zhang J. Wu J. Wei X. J. Yang S. W. (2023). Clinical observation of Fuxin Heji Decoction combined with metoprolol succinate sustained-release tablets in the treatment of coronary artery myobridge angina. Chin. J. Diffic. Complic. Cases. 22 (3), 253–257+271. 10.3969/j.issn.1671-6450.2023.03.006

[B54] Zhang X. Cai Y. Chen M. Chen L. Mao Y. He R. (2025). Danshen-Chuanxiong-Honghua ameliorates neurological function and inflammation in traumatic brain injury in rats *via* modulating Ghrelin/GHSR. J. Ethnopharmacol. 345, 119625. 10.1016/j.jep.2025.119625 40074098

[B55] Zhao M. J. Yan W. L. Zhao Y. (2011). Coronary myocardial bridge massage treated by tongxinluo capsule. Shaanxi J. Tradit. Chin. Med. 32 (6), 645+658. 10.3969/j.issn.1000-7369.2011.06.002

[B56] Zheng Y. H. Xie J. N. Lin S. Zhang W. Chen Z. G. Zhou J. R. (2012). Clinical observation of “Shunqi Tongxin Capsule” in the treatment of 40 cases of patients with Angina Pectoris due to coronary artery myocardial bridge. Jiangsu J. Tradit. Chin. Med. 44 (9), 13–14. 10.3969/j.issn.1672-397X.2012.09.008

[B57] Zhong Q. Y. Guo J. J. (2019). Clinical observation on treatment of Qi stagnation and blood stasis type myocardial Bridge from liver. Guangming J. Chin. Med. 34 (13), 1967–1970. 10.3969/j.issn.1003-8914.2019.13.012

[B58] Zhu G. Y. (2014). Clinical observation of yingxinning capsule and metoprolol in treatment of coronary myocardial bridge. Hubei J. Tradit. Chin. Med. 36 (5), 3–4.

