# Supplementary material for: Efficacy and safety of Traditional Chinese Medicine in alleviating symptoms associated with myocardial bridge: a systematic review and meta-analysis
Source: Front Pharmacol. 2025 Sep 19;16:1619617. doi: 10.3389/fphar.2025.1619617 (PMC12492955; doi:10.3389/fphar.2025.1619617)

Detailed Filling Strategy of Missing Value

S1. For Age

S1.1 Mean Imputation (4 RCTs)

$$SD_{mean} = \frac{1}{n}\sum_{i=1}^{n} SD_i$$

Study or Subgroup	Intervention Mean	Intervention SD	Intervention Total	Control Mean	Control SD	Control Total	Weight	Mean Difference IV, Fixed, 95% CI
Cao, M. (2024)	58.61	3.46	35	58.25	3.24	35	8.3%	0.36 [-1.21, 1.93]
Chen, J. (2017)	53.48	1.54	60	52.31	3.55	60	21.4%	1.17 [0.19, 2.15]
Cong, X. (2021)	62.5	7.2	45	61.7	7.2	45	2.3%	0.80 [-2.18, 3.78]
Fan, G. H. (2012)	47.7	6.4	36	49.4	6.8	36	2.2%	-1.70 [-4.75, 1.35]
Han, Y. (2022)	53.3	4.2	32	55.1	3.2	32	6.1%	-1.80 [-3.63, 0.03]
Li, J. (2014)	64.7	5.43	30	62.2	5.4	30	2.7%	2.50 [-0.24, 5.24]
Qiao, J. F. (2021)	62.77	11.46	30	61.33	12.74	30	0.5%	1.44 [-4.69, 7.57]
Wang, J. (2016)	62.5	6.6	40	64.1	7.1	40	2.3%	-1.60 [-4.60, 1.40]
Wang, J. G. (2016)	54.11	5.43	30	53.22	5.4	30	2.7%	0.89 [-1.85, 3.63]
Wang, Z. X. (2020)	64.15	2.25	25	63.35	2.27	25	13.0%	0.80 [-0.45, 2.05]
Yin, X. S. (2021)	65.12	2.34	35	65.52	2.21	35	18.0%	-0.40 [-1.47, 0.67]
Yuan, H. W. (2018)	65.6	5.43	34	63.2	5.4	34	3.1%	2.40 [-0.17, 4.97]
Zhang, J. (2023)	62.24	4.67	46	63.39	5.35	46	4.9%	-1.15 [-3.20, 0.90]
Zhang, P. (2016)	50.83	4.01	30	51.74	3.95	30	5.0%	-0.91 [-2.92, 1.10]
Zhao, M. J. (2011)	49.7	7.3	20	50.2	4.2	18	1.5%	-0.50 [-4.24, 3.24]
Zheng, Y. H. (2012)	61.8	5.43	40	60.5	5.4	40	3.6%	1.30 [-1.07, 3.67]
Zhong, Q. Y. (2019)	58.67	9.15	30	60.77	7.68	30	1.1%	-2.10 [-6.37, 2.17]
Zhu, G. Y. (2014)	46.5	5.4	15	48.3	6.1	15	1.2%	-1.80 [-5.92, 2.32]
**Total (95% CI)**			**613**			**611**	**100.0%**	**0.21 [-0.24, 0.67]**

Heterogeneity: Chi² = 25.24, df = 17 (P = 0.09); I² = 33%
Test for overall effect: Z = 0.93 (P = 0.35)

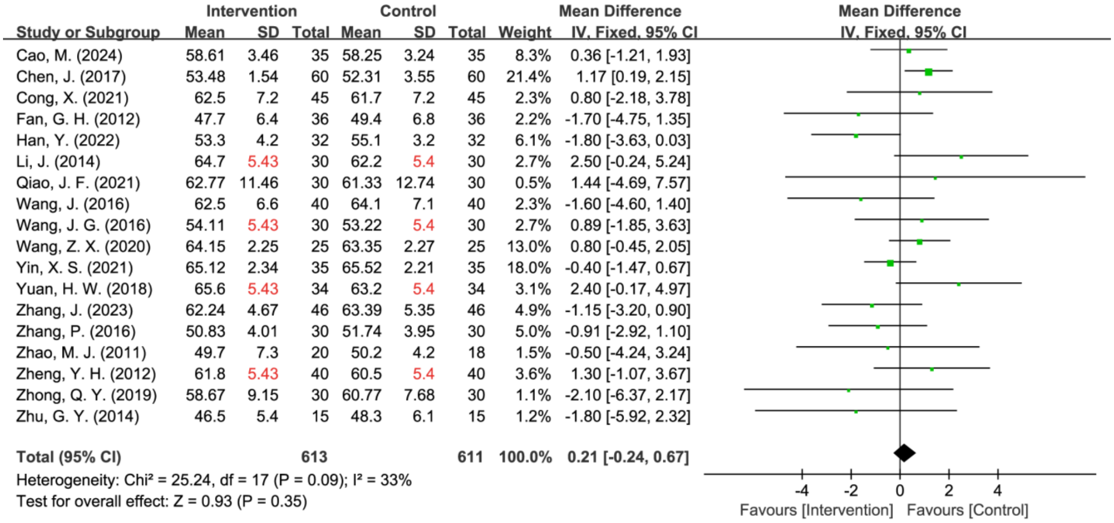

S1.2 Sample Size-Weighted Mean Imputation (4 RCTs)

$$SD_{pooled} = \sqrt{\frac{\sum(n_i - 1)\times SD_i^2}{\sum(n_i - 1)}}$$

Study or Subgroup	Intervention Mean	Intervention SD	Intervention Total	Control Mean	Control SD	Control Total	Weight	Mean Difference IV, Fixed, 95% CI
Cao, M. (2024)	58.61	3.46	35	58.25	3.24	35	8.5%	0.36 [-1.21, 1.93]
Chen, J. (2017)	53.48	1.54	60	52.31	3.55	60	21.8%	1.17 [0.19, 2.15]
Cong, X. (2021)	62.5	7.2	45	61.7	7.2	45	2.4%	0.80 [-2.18, 3.78]
Fan, G. H. (2012)	47.7	6.4	36	49.4	6.8	36	2.2%	-1.70 [-4.75, 1.35]
Han, Y. (2022)	53.3	4.2	32	55.1	3.2	32	6.2%	-1.80 [-3.63, 0.03]
Li, J. (2014)	64.7	5.85	30	62.2	5.98	30	2.3%	2.50 [-0.49, 5.49]
Qiao, J. F. (2021)	62.77	11.46	30	61.33	12.74	30	0.6%	1.44 [-4.69, 7.57]
Wang, J. (2016)	62.5	6.6	40	64.1	7.1	40	2.3%	-1.60 [-4.60, 1.40]
Wang, J. G. (2016)	54.11	5.85	30	53.22	5.98	30	2.3%	0.89 [-2.10, 3.88]
Wang, Z. X. (2020)	64.15	2.25	25	63.35	2.27	25	13.3%	0.80 [-0.45, 2.05]
Yin, X. S. (2021)	65.12	2.34	35	65.52	2.21	35	18.4%	-0.40 [-1.47, 0.67]
Yuan, H. W. (2018)	65.6	5.85	34	63.2	5.98	34	2.6%	2.40 [-0.41, 5.21]
Zhang, J. (2023)	62.24	4.67	46	63.39	5.35	46	5.0%	-1.15 [-3.20, 0.90]
Zhang, P. (2016)	50.83	4.01	30	51.74	3.95	30	5.1%	-0.91 [-2.92, 1.10]
Zhao, M. J. (2011)	49.7	7.3	20	50.2	4.2	18	1.5%	-0.50 [-4.24, 3.24]
Zheng, Y. H. (2012)	61.8	5.85	40	60.5	5.98	40	3.1%	1.30 [-1.29, 3.89]
Zhong, Q. Y. (2019)	58.67	9.15	30	60.77	7.68	30	1.1%	-2.10 [-6.37, 2.17]
Zhu, G. Y. (2014)	46.5	5.4	15	48.3	6.1	15	1.2%	-1.80 [-5.92, 2.32]
**Total (95% CI)**			**613**			**611**	**100.0%**	**0.18 [-0.27, 0.64]**

Heterogeneity: Chi² = 24.17, df = 17 (P = 0.11); I² = 30%
Test for overall effect: Z = 0.78 (P = 0.43)

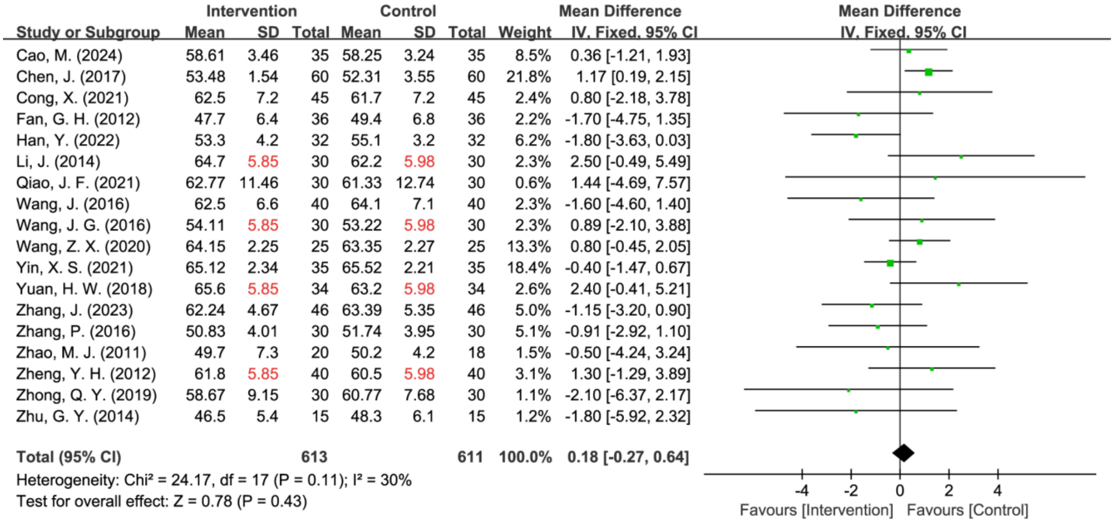

S1.3 Median Imputation (4 RCTs)

$$Median = \frac{SD_{\frac{n}{2}} + SD_{\frac{n}{2}+1}}{2}$$

Study or Subgroup	Intervention Mean	Intervention SD	Intervention Total	Control Mean	Control SD	Control Total	Weight	Mean Difference IV, Fixed, 95% CI
Cao, M. (2024)	58.61	3.46	35	58.25	3.24	35	8.1%	0.36 [-1.21, 1.93]
Chen, J. (2017)	53.48	1.54	60	52.31	3.55	60	20.8%	1.17 [0.19, 2.15]
Cong, X. (2021)	62.5	7.2	45	61.7	7.2	45	2.3%	0.80 [-2.18, 3.78]
Fan, G. H. (2012)	47.7	6.4	36	49.4	6.8	36	2.1%	-1.70 [-4.75, 1.35]
Han, Y. (2022)	53.3	4.2	32	55.1	3.2	32	6.0%	-1.80 [-3.63, 0.03]
Li, J. (2014)	64.7	5.04	30	62.2	4.78	30	3.2%	2.50 [0.01, 4.99]
Qiao, J. F. (2021)	62.77	11.46	30	61.33	12.74	30	0.5%	1.44 [-4.69, 7.57]
Wang, J. (2016)	62.5	6.6	40	64.1	7.1	40	2.2%	-1.60 [-4.60, 1.40]
Wang, J. G. (2016)	54.11	5.04	30	53.22	4.78	30	3.2%	0.89 [-1.60, 3.38]
Wang, Z. X. (2020)	64.15	2.25	25	63.35	2.27	25	12.7%	0.80 [-0.45, 2.05]
Yin, X. S. (2021)	65.12	2.34	35	65.52	2.21	35	17.5%	-0.40 [-1.47, 0.67]
Yuan, H. W. (2018)	65.6	5.04	34	63.2	4.78	34	3.7%	2.40 [0.07, 4.73]
Zhang, J. (2023)	62.24	4.67	46	63.39	5.35	46	4.7%	-1.15 [-3.20, 0.90]
Zhang, P. (2016)	50.83	4.01	30	51.74	3.95	30	4.9%	-0.91 [-2.92, 1.10]
Zhao, M. J. (2011)	49.7	7.3	20	50.2	4.2	18	1.4%	-0.50 [-4.24, 3.24]
Zheng, Y. H. (2012)	61.8	5.04	40	60.5	4.78	40	4.3%	1.30 [-0.85, 3.45]
Zhong, Q. Y. (2019)	58.67	9.15	30	60.77	7.68	30	1.1%	-2.10 [-6.37, 2.17]
Zhu, G. Y. (2014)	46.5	5.4	15	48.3	6.1	15	1.2%	-1.80 [-5.92, 2.32]
**Total (95% CI)**			**613**			**611**	**100.0%**	**0.25 [-0.19, 0.70]**

Heterogeneity: Chi² = 26.60, df = 17 (P = 0.06); I² = 36%
Test for overall effect: Z = 1.11 (P = 0.27)

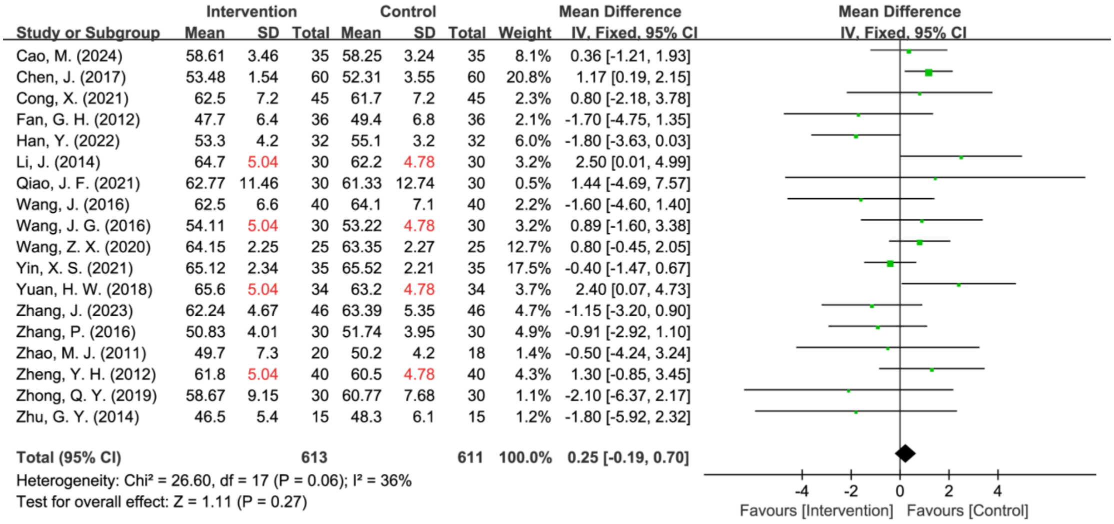

S1.4 Hozo’s Range Rule (3 RCTs) + Mean Imputation (1 RCT)

$$\mathrm{SD} = \frac{\mathrm{Age_{max}} - \mathrm{Age_{min}}}{4} \quad (15 < n \leq 70) \quad \text{(Hozo's Range Rule)}$$

Study or Subgroup	Intervention Mean	Intervention SD	Intervention Total	Control Mean	Control SD	Control Total	Weight	Mean Difference IV, Fixed, 95% CI
Cao, M. (2024)	58.61	3.46	35	58.25	3.24	35	8.8%	0.36 [-1.21, 1.93]
Chen, J. (2017)	53.48	1.54	60	52.31	3.55	60	22.6%	1.17 [0.19, 2.15]
Cong, X. (2021)	62.5	7.2	45	61.7	7.2	45	2.4%	0.80 [-2.18, 3.78]
Fan, G. H. (2012)	47.7	6.4	36	49.4	6.8	36	2.3%	-1.70 [-4.75, 1.35]
Han, Y. (2022)	53.3	4.2	32	55.1	3.2	32	6.5%	-1.80 [-3.63, 0.03]
Li, J. (2014)	64.7	7	30	62.2	6.75	30	1.8%	2.50 [-0.98, 5.98]
Qiao, J. F. (2021)	62.77	11.46	30	61.33	12.74	30	0.6%	1.44 [-4.69, 7.57]
Wang, J. (2016)	62.5	6.6	40	64.1	7.1	40	2.4%	-1.60 [-4.60, 1.40]
Wang, J. G. (2016)	54.11	5.88	30	53.22	5.92	30	2.4%	0.89 [-2.10, 3.88]
Wang, Z. X. (2020)	64.15	2.25	25	63.35	2.27	25	13.8%	0.80 [-0.45, 2.05]
Yin, X. S. (2021)	65.12	2.34	35	65.52	2.21	35	19.1%	-0.40 [-1.47, 0.67]
Yuan, H. W. (2018)	65.6	7	34	63.2	7.75	34	1.8%	2.40 [-1.11, 5.91]
Zhang, J. (2023)	62.24	4.67	46	63.39	5.35	46	5.1%	-1.15 [-3.20, 0.90]
Zhang, P. (2016)	50.83	4.01	30	51.74	3.95	30	5.3%	-0.91 [-2.92, 1.10]
Zhao, M. J. (2011)	49.7	7.3	20	50.2	4.2	18	1.5%	-0.50 [-4.24, 3.24]
Zheng, Y. H. (2012)	61.8	10	40	60.5	10.5	40	1.1%	1.30 [-3.19, 5.79]
Zhong, Q. Y. (2019)	58.67	9.15	30	60.77	7.68	30	1.2%	-2.10 [-6.37, 2.17]
Zhu, G. Y. (2014)	46.5	5.4	15	48.3	6.1	15	1.3%	-1.80 [-5.92, 2.32]
**Total (95% CI)**			**613**			**611**	**100.0%**	**0.12 [-0.34, 0.59]**

Heterogeneity: Chi² = 22.18, df = 17 (P = 0.18); I² = 23%
Test for overall effect: Z = 0.52 (P = 0.61)

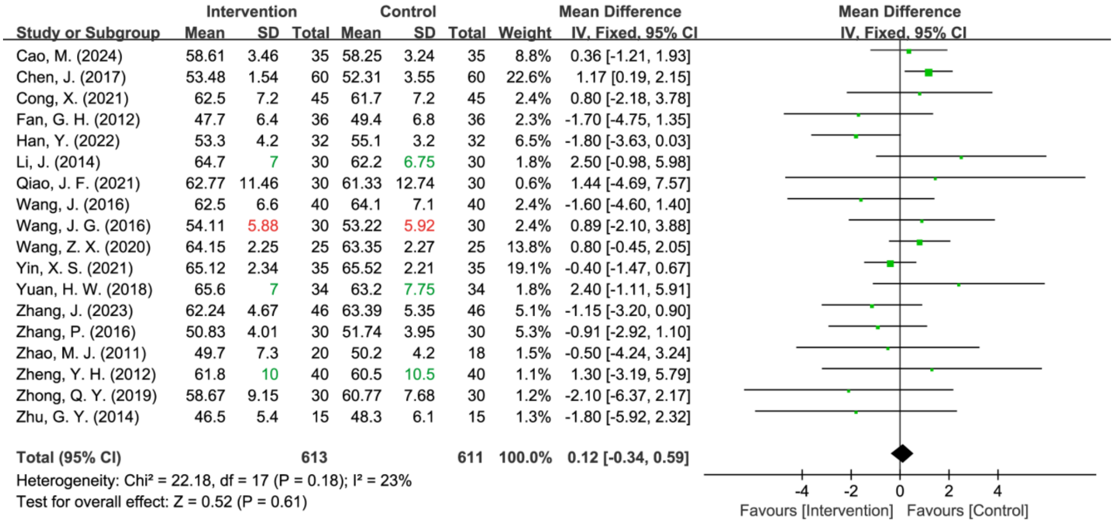

S1.5 Hozo’s Range Rule (3 RCTs) + Sample Size-Weighted Mean Imputation (1 RCT)

$$\mathrm{SD_{pooled}} = \sqrt{\frac{\sum (n_i - 1) \times \mathrm{SD}_i^2}{\sum (n_i - 1)}}$$

Study or Subgroup	Intervention Mean	Intervention SD	Intervention Total	Control Mean	Control SD	Control Total	Weight	Mean Difference IV, Fixed, 95% CI
Cao, M. (2024)	58.61	3.46	35	58.25	3.24	35	8.8%	0.36 [-1.21, 1.93]
Chen, J. (2017)	53.48	1.54	60	52.31	3.55	60	22.7%	1.17 [0.19, 2.15]
Cong, X. (2021)	62.5	7.2	45	61.7	7.2	45	2.5%	0.80 [-2.18, 3.78]
Fan, G. H. (2012)	47.7	6.4	36	49.4	6.8	36	2.3%	-1.70 [-4.75, 1.35]
Han, Y. (2022)	53.3	4.2	32	55.1	3.2	32	6.5%	-1.80 [-3.63, 0.03]
Li, J. (2014)	64.7	7	30	62.2	6.75	30	1.8%	2.50 [-0.98, 5.98]
Qiao, J. F. (2021)	62.77	11.46	30	61.33	12.74	30	0.6%	1.44 [-4.69, 7.57]
Wang, J. (2016)	62.5	6.6	40	64.1	7.1	40	2.4%	-1.60 [-4.60, 1.40]
Wang, J. G. (2016)	54.11	6.35	30	53.22	6.54	30	2.0%	0.89 [-2.37, 4.15]
Wang, Z. X. (2020)	64.15	2.25	25	63.35	2.27	25	13.9%	0.80 [-0.45, 2.05]
Yin, X. S. (2021)	65.12	2.34	35	65.52	2.21	35	19.1%	-0.40 [-1.47, 0.67]
Yuan, H. W. (2018)	65.6	7	34	63.2	7.75	34	1.8%	2.40 [-1.11, 5.91]
Zhang, J. (2023)	62.24	4.67	46	63.39	5.35	46	5.2%	-1.15 [-3.20, 0.90]
Zhang, P. (2016)	50.83	4.01	30	51.74	3.95	30	5.4%	-0.91 [-2.92, 1.10]
Zhao, M. J. (2011)	49.7	7.3	20	50.2	4.2	18	1.6%	-0.50 [-4.24, 3.24]
Zheng, Y. H. (2012)	61.8	10	40	60.5	10.5	40	1.1%	1.30 [-3.19, 5.79]
Zhong, Q. Y. (2019)	58.67	9.15	30	60.77	7.68	30	1.2%	-2.10 [-6.37, 2.17]
Zhu, G. Y. (2014)	46.5	5.4	15	48.3	6.1	15	1.3%	-1.80 [-5.92, 2.32]
**Total (95% CI)**			**613**			**611**	**100.0%**	**0.12 [-0.35, 0.59]**

Heterogeneity: Chi² = 22.14, df = 17 (P = 0.18); I² = 23%
Test for overall effect: Z = 0.50 (P = 0.61)

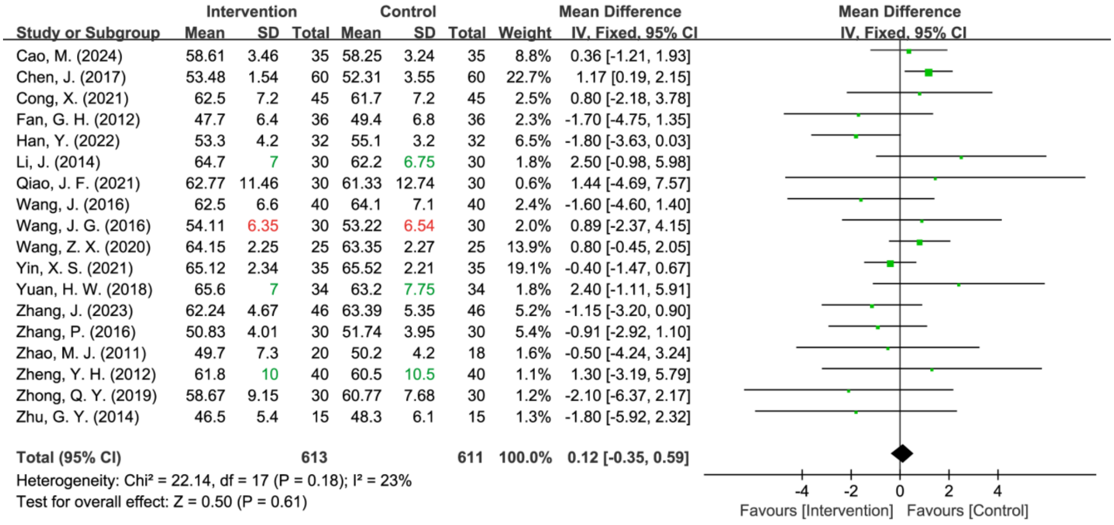

S1.6 Hozo's Range Rule (3 RCTs) + Median Imputation (1 RCT)

$$\text{Median} = \text{SD}_{\frac{n+1}{2}}$$

Study or Subgroup	Intervention Mean	Intervention SD	Intervention Total	Control Mean	Control SD	Control Total	Weight	Mean Difference IV, Fixed, 95% CI
Cao, M. (2024)	58.61	3.46	35	58.25	3.24	35	8.8%	0.36 [-1.21, 1.93]
Chen, J. (2017)	53.48	1.54	60	52.31	3.55	60	22.7%	1.17 [0.19, 2.15]
Cong, X. (2021)	62.5	7.2	45	61.7	7.2	45	2.5%	0.80 [-2.18, 3.78]
Fan, G. H. (2012)	47.7	6.4	36	49.4	6.8	36	2.3%	-1.70 [-4.75, 1.35]
Han, Y. (2022)	53.3	4.2	32	55.1	3.2	32	6.5%	-1.80 [-3.63, 0.03]
Li, J. (2014)	64.7	7	30	62.2	6.75	30	1.8%	2.50 [-0.98, 5.98]
Qiao, J. F. (2021)	62.77	11.46	30	61.33	12.74	30	0.6%	1.44 [-4.69, 7.57]
Wang, J. (2016)	62.5	6.6	40	64.1	7.1	40	2.4%	-1.60 [-4.60, 1.40]
Wang, J. G. (2016)	54.11	6.4	30	53.22	6.1	30	2.2%	0.89 [-2.27, 4.05]
Wang, Z. X. (2020)	64.15	2.25	25	63.35	2.27	25	13.8%	0.80 [-0.45, 2.05]
Yin, X. S. (2021)	65.12	2.34	35	65.52	2.21	35	19.1%	-0.40 [-1.47, 0.67]
Yuan, H. W. (2018)	65.6	7	34	63.2	7.75	34	1.8%	2.40 [-1.11, 5.91]
Zhang, J. (2023)	62.24	4.67	46	63.39	5.35	46	5.2%	-1.15 [-3.20, 0.90]
Zhang, P. (2016)	50.83	4.01	30	51.74	3.95	30	5.4%	-0.91 [-2.92, 1.10]
Zhao, M. J. (2011)	49.7	7.3	20	50.2	4.2	18	1.6%	-0.50 [-4.24, 3.24]
Zheng, Y. H. (2012)	61.8	10	40	60.5	10.5	40	1.1%	1.30 [-3.19, 5.79]
Zhong, Q. Y. (2019)	58.67	9.15	30	60.77	7.68	30	1.2%	-2.10 [-6.37, 2.17]
Zhu, G. Y. (2014)	46.5	5.4	15	48.3	6.1	15	1.3%	-1.80 [-5.92, 2.32]
**Total (95% CI)**			**613**			**611**	**100.0%**	**0.12 [-0.35, 0.59]**

Heterogeneity: Chi² = 22.15, df = 17 (P = 0.18); I² = 23%
Test for overall effect: Z = 0.51 (P = 0.61)

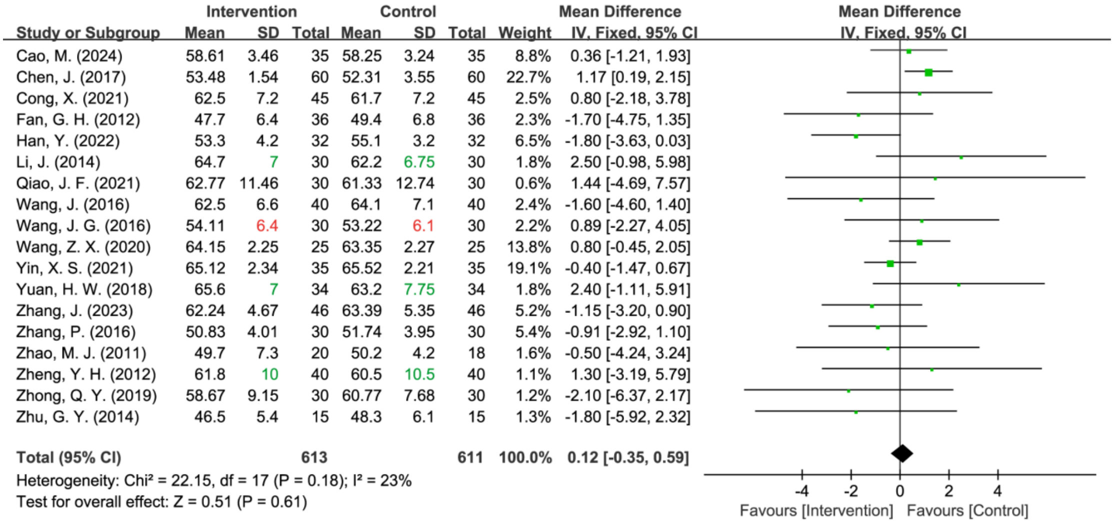

S1.7 Remaining 14 RCTs (Exclude 4 SD Missing RCTs)

Study or Subgroup	Intervention Mean	Intervention SD	Intervention Total	Control Mean	Control SD	Control Total	Weight	Mean Difference IV, Fixed, 95% CI
Cao, M. (2024)	58.61	3.46	35	58.25	3.24	35	9.5%	0.36 [-1.21, 1.93]
Chen, J. (2017)	53.48	1.54	60	52.31	3.55	60	24.3%	1.17 [0.19, 2.15]
Cong, X. (2021)	62.5	7.2	45	61.7	7.2	45	2.6%	0.80 [-2.18, 3.78]
Fan, G. H. (2012)	47.7	6.4	36	49.4	6.8	36	2.5%	-1.70 [-4.75, 1.35]
Han, Y. (2022)	53.3	4.2	32	55.1	3.2	32	7.0%	-1.80 [-3.63, 0.03]
Qiao, J. F. (2021)	62.77	11.46	30	61.33	12.74	30	0.6%	1.44 [-4.69, 7.57]
Wang, J. G. (2016)	62.5	6.6	40	64.1	7.1	40	2.6%	-1.60 [-4.60, 1.40]
Wang, Z. X. (2020)	64.15	2.25	25	63.35	2.27	25	14.8%	0.80 [-0.45, 2.05]
Yin, X. S. (2021)	65.12	2.34	35	65.52	2.21	35	20.5%	-0.40 [-1.47, 0.67]
Zhang, J. (2023)	62.24	4.67	46	63.39	5.35	46	5.5%	-1.15 [-3.20, 0.90]
Zhang, P. (2016)	50.83	4.01	30	51.74	3.95	30	5.7%	-0.91 [-2.92, 1.10]
Zhao, M. J. (2011)	49.7	7.3	20	50.2	4.2	18	1.7%	-0.50 [-4.24, 3.24]
Zhong, Q. Y. (2019)	58.67	9.15	30	60.77	7.68	30	1.3%	-2.10 [-6.37, 2.17]
Zhu, G. Y. (2014)	46.5	5.4	15	48.3	6.1	15	1.4%	-1.80 [-5.92, 2.32]
**Total (95% CI)**			**479**			**477**	**100.0%**	**0.00 [-0.48, 0.48]**

Heterogeneity: Chi² = 18.00, df = 13 (P = 0.16); I² = 28%
Test for overall effect: Z = 0.00 (P = 1.00)

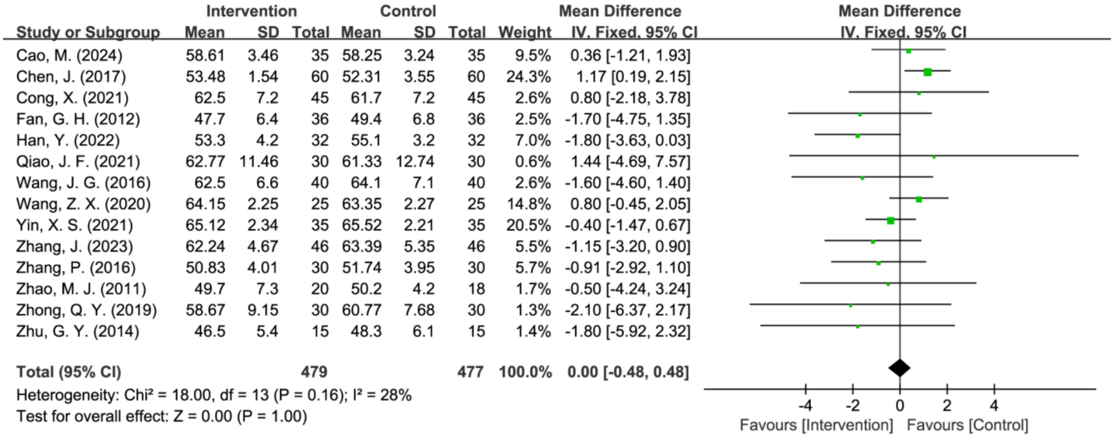

S1.8 Remaining 17 RCTs (Exclude 1 SD Missing RCT)

Study or Subgroup	Intervention Mean	Intervention SD	Intervention Total	Control Mean	Control SD	Control Total	Weight	Mean Difference IV, Fixed, 95% CI
Cao, M. (2024)	58.61	3.46	35	58.25	3.24	35	9.0%	0.36 [-1.21, 1.93]
Chen, J. (2017)	53.48	1.54	60	52.31	3.55	60	23.2%	1.17 [0.19, 2.15]
Cong, X. (2021)	62.5	7.2	45	61.7	7.2	45	2.5%	0.80 [-2.18, 3.78]
Fan, G. H. (2012)	47.7	6.4	36	49.4	6.8	36	2.4%	-1.70 [-4.75, 1.35]
Han, Y. (2022)	53.3	4.2	32	55.1	3.2	32	6.6%	-1.80 [-3.63, 0.03]
Li, J. (2014)	64.7	7	30	62.2	6.75	30	1.8%	2.50 [-0.98, 5.98]
Qiao, J. F. (2021)	62.77	11.46	30	61.33	12.74	30	0.6%	1.44 [-4.69, 7.57]
Wang, J. G. (2016)	62.5	6.6	40	64.1	7.1	40	2.5%	-1.60 [-4.60, 1.40]
Wang, Z. X. (2020)	64.15	2.25	25	63.35	2.27	25	14.1%	0.80 [-0.45, 2.05]
Yin, X. S. (2021)	65.12	2.34	35	65.52	2.21	35	19.5%	-0.40 [-1.47, 0.67]
Yuan, H. W. (2018)	65.6	7	34	63.2	7.75	34	1.8%	2.40 [-1.11, 5.91]
Zhang, J. (2023)	62.24	4.67	46	63.39	5.35	46	5.3%	-1.15 [-3.20, 0.90]
Zhang, P. (2016)	50.83	4.01	30	51.74	3.95	30	5.5%	-0.91 [-2.92, 1.10]
Zhao, M. J. (2011)	49.7	7.3	20	50.2	4.2	18	1.6%	-0.50 [-4.24, 3.24]
Zheng, Y. H. (2012)	61.8	10	40	60.5	10.5	40	1.1%	1.30 [-3.19, 5.79]
Zhong, Q. Y. (2019)	58.67	9.15	30	60.77	7.68	30	1.2%	-2.10 [-6.37, 2.17]
Zhu, G. Y. (2014)	46.5	5.4	15	48.3	6.1	15	1.3%	-1.80 [-5.92, 2.32]
**Total (95% CI)**			**583**			**581**	**100.0%**	**0.10 [-0.37, 0.57]**

Heterogeneity: Chi² = 21.92, df = 16 (P = 0.15); I² = 27%

Test for overall effect: Z = 0.43 (P = 0.67)

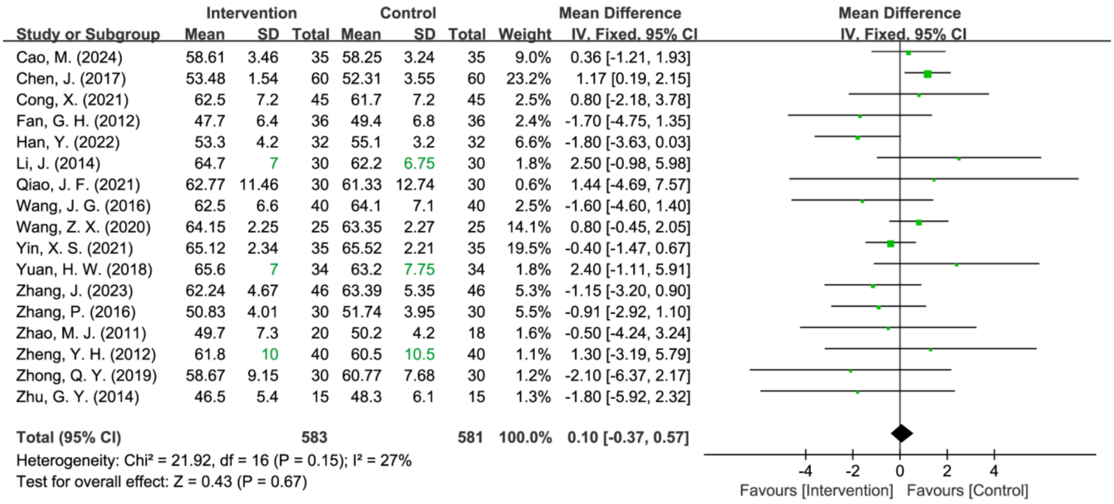

S2 For Disease Duration

S2.1 Mean Imputation (1 RCT)

Study or Subgroup	Intervention Mean	Intervention SD	Intervention Total	Control Mean	Control SD	Control Total	Weight	Mean Difference IV, Random, 95% CI
Cao, M. (2024)	1.25	0.11	35	1.34	0.12	35	38.7%	-0.09 [-0.14, -0.04]
Chen, J. (2017)	8.56	1.36	60	9.35	3.62	60	3.3%	-0.79 [-1.77, 0.19]
Cong, X. (2021)	2.3	0.5	45	2.2	0.6	45	24.9%	0.10 [-0.13, 0.33]
Wang, J. (2016)	5.1	0.68	30	4.8	1.2	30	10.4%	0.30 [-0.19, 0.79]
Zhang, J. (2023)	2.34	0.76	46	2.17	0.45	46	22.7%	0.17 [-0.09, 0.43]
**Total (95% CI)**			**216**			**216**	**100.0%**	**0.03 [-0.15, 0.22]**

Heterogeneity: Tau² = 0.02; Chi² = 10.29, df = 4 (P = 0.04); I² = 61%
Test for overall effect: Z = 0.36 (P = 0.72)

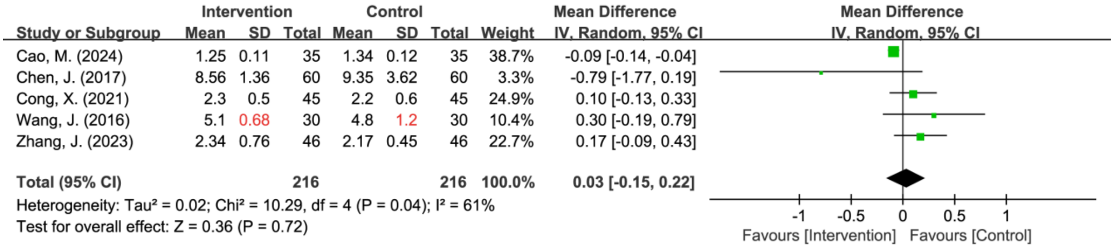

S2.2 Sample Size-Weighted Mean Imputation (1 RCT)

Study or Subgroup	Intervention Mean	Intervention SD	Intervention Total	Control Mean	Control SD	Control Total	Weight	Mean Difference IV, Random, 95% CI
Cao, M. (2024)	1.25	0.11	35	1.34	0.12	35	43.1%	-0.09 [-0.14, -0.04]
Chen, J. (2017)	8.56	1.36	60	9.35	3.62	60	3.1%	-0.79 [-1.77, 0.19]
Cong, X. (2021)	2.3	0.5	45	2.2	0.6	45	25.9%	0.10 [-0.13, 0.33]
Wang, J. (2016)	5.1	0.9	30	4.8	2.09	30	4.4%	0.30 [-0.51, 1.11]
Zhang, J. (2023)	2.34	0.76	46	2.17	0.45	46	23.5%	0.17 [-0.09, 0.43]
**Total (95% CI)**			**216**			**216**	**100.0%**	**0.02 [-0.16, 0.19]**

Heterogeneity: Tau² = 0.02; Chi² = 8.93, df = 4 (P = 0.06); I² = 55%
Test for overall effect: Z = 0.17 (P = 0.87)

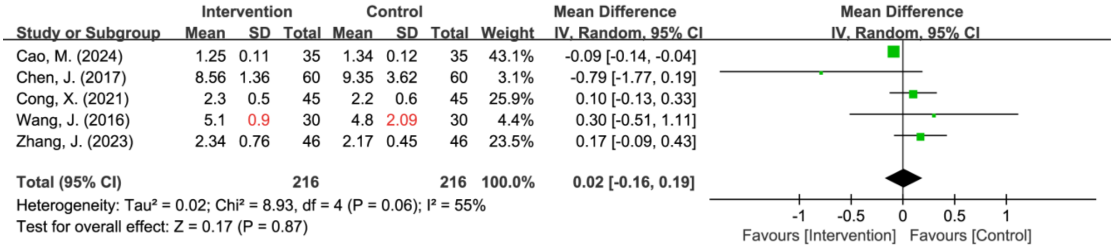

S2.3 Median Imputation (1 RCT)

Study or Subgroup	Intervention Mean	Intervention SD	Intervention Total	Control Mean	Control SD	Control Total	Weight	Mean Difference IV, Random, 95% CI
Cao, M. (2024)	1.25	0.11	35	1.34	0.12	35	32.6%	-0.09 [-0.14, -0.04]
Chen, J. (2017)	8.56	1.36	60	9.35	3.62	60	3.6%	-0.79 [-1.77, 0.19]
Cong, X. (2021)	2.3	0.5	45	2.2	0.6	45	23.1%	0.10 [-0.13, 0.33]
Wang, J. (2016)	5.1	0.63	30	4.8	0.525	30	19.2%	0.30 [0.01, 0.59]
Zhang, J. (2023)	2.34	0.76	46	2.17	0.45	46	21.4%	0.17 [-0.09, 0.43]
**Total (95% CI)**			**216**			**216**	**100.0%**	**0.06 [-0.14, 0.26]**

Heterogeneity: Tau² = 0.03; Chi² = 14.12, df = 4 (P = 0.007); I² = 72%
Test for overall effect: Z = 0.59 (P = 0.56)

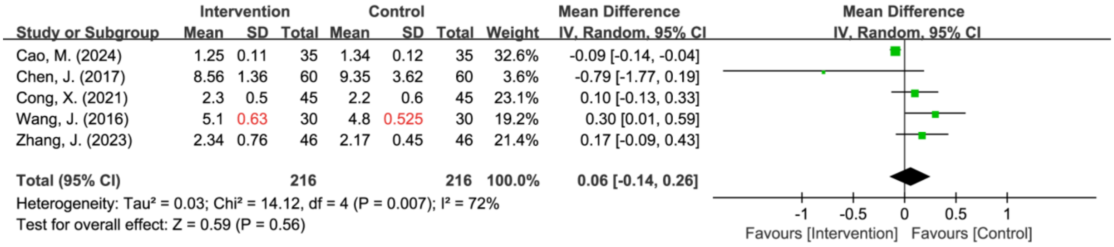

S2.4 Remaining 4 RCTs (Exclude 1 SD Missing RCT)

Study or Subgroup	Intervention Mean	Intervention SD	Intervention Total	Control Mean	Control SD	Control Total	Weight	Mean Difference IV, Random, 95% CI
Cao, M. (2024)	1.25	0.11	35	1.34	0.12	35	44.2%	-0.09 [-0.14, -0.04]
Chen, J. (2017)	8.56	1.36	60	9.35	3.62	60	3.4%	-0.79 [-1.77, 0.19]
Cong, X. (2021)	2.3	0.5	45	2.2	0.6	45	27.4%	0.10 [-0.13, 0.33]
Zhang, J. (2023)	2.34	0.76	46	2.17	0.45	46	24.9%	0.17 [-0.09, 0.43]
**Total (95% CI)**			**186**			**186**	**100.0%**	**0.00 [-0.19, 0.19]**

Heterogeneity: Tau² = 0.02; Chi² = 8.13, df = 3 (P = 0.04); I² = 63%
Test for overall effect: Z = 0.03 (P = 0.98)

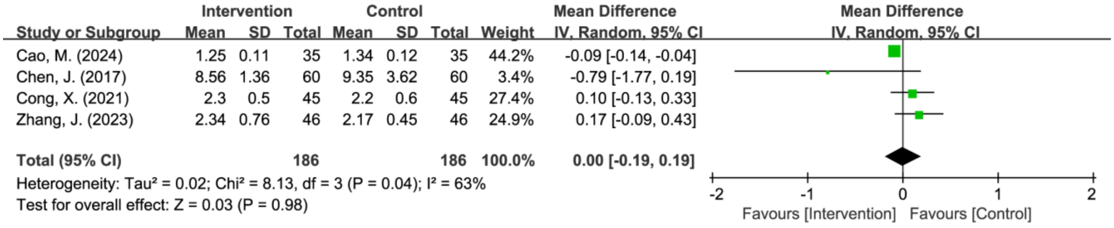

Supplement: Supplementary file 1 [file DataSheet2.pdf]
