# Supplementary material for: Efficacy and safety of Traditional Chinese Medicine in alleviating symptoms associated with myocardial bridge: a systematic review and meta-analysis
Source: Front Pharmacol. 2025 Sep 19;16:1619617. doi: 10.3389/fphar.2025.1619617 (PMC12492955; doi:10.3389/fphar.2025.1619617)
Supplement: Supplementary file 2 [file DataSheet4.pdf]

NO.	Author	Formulas and components	Usage	ConPhyMP Type
1	Cao, M. (2024)	复心合剂（Fuxin Heji Decoction）：<br>炙黄芪（zhi huangqi）：*Astragalus mongholicus* Bunge [Fabaceae; *Astragali radix praeparata cum melle*] 30g；<br>白术（baizhu）：*Atractylodes macrocephala* Koidz [Asteraceae; *Atractylodis macrocephalae rhizoma*] 15g；<br>附子（fuzi）：*Aconitum carmichaelii* Debeaux [Ranunculaceae; *Aconiti lateralis radix praeparata*] 15g；<br>当归（danggui）：*Angelica sinensis* (Oliv.) Diels [Apiaceae; *Angelicae sinensis radix*] 15g；<br>泽泻（zexie）：*Alisma orientale* (Sam.) Juz. [Alismataceae; *Alismatis rhizoma*] 15g；<br>党参（dangshen）：*Codonopsis pilosula* (Franch.) Nannf. [Campanulaceae; *Codonopsis radix*] 15g；<br>赤芍（chishao）：*Paeonia lactiflora* Pall. [Paeoniaceae; *Paeoniae radix rubra*] 15g；<br>川芎（chuanxiong）：*Ligusticum chuanxiong* Hort. [Apiaceae; *Chuanxiong rhizoma*] 15g；<br>丹参（danshen）：*Salvia miltiorrhiza* Bunge [Lamiaceae; *Salviae miltiorrhizae radix et rhizoma*] 10g；<br>柴胡（chaihu）：*Bupleurum chinense* DC. [Apiaceae; *Bupleuri radix*] 10g；<br>白芍（baishao）：*Paeonia lactiflora* Pall. [Paeoniaceae; *Paeoniae radix alba*] 10g；<br>木香（muxiang）：*Aucklandia costus* (Falc.) Kasana & A.K.Pandey [Asteraceae; *Aucklandiae radix*] 10g；<br>升麻（shengma）：*Actaea cimicifuga* L. [Ranunculaceae; *Cimicifugae rhizoma*] 10g；<br>麦冬（maidong）：*Ophiopogon japonicus* (Thunb.) Ker-Gawl. [Asparagaceae; *Ophiopogonis radix*] 10g；<br>熟地黄（shu dihuang）：*Rehmannia glutinosa* (Gaertn.) Libosch. ex DC. [Orobanchaceae; *Rehmanniae radix praeparata*] 10g；<br>陈皮（chenpi）：*Citrus reticulata* Blanco [Rutaceae; *Citri reticulatae pericarpium*] 10g；<br>炙甘草（zhi gancao）：*Glycyrrhiza glabra* L. [Fabaceae; *Glycyrrhizae radix et rhizoma praeparata cum melle*] 5g.	Prepare by water decoction to yield 400 mL, taken orally in two equal doses (morning and evening), with a daily dosage of one preparation.	B

NO.	Author	Formulas and components	Usage	ConPhyMP Type
2	Chen, J. (2017)	理气活血通络汤（Liqi Huoxue Tongluo Formula）：<br>柴胡（chaihu）：*Bupleurum chinense* DC. [Apiaceae; *Bupleuri radix*] 10g；<br>白芍（baishao）：*Paeonia lactiflora* Pall. [Paeoniaceae; *Paeoniae radix alba*] 15g；<br>枳实（zhishi）：*Citrus* × *aurantium* f. *aurantium* [Rutaceae; *Aurantii fructus immaturus*] 10g；<br>瓜蒌（gualou）：*Trichosanthes kirilowii* Maxim. [Cucurbitaceae; *Trichosanthis fructus*] 15g；<br>薤白（xiebai）：*Allium macrostemon* Bunge [Alliaceae; *Allii macrostemonis bulbus*] 10g；<br>法半夏（fa banxia）：*Pinellia ternata* (Thunb.) Makino [Araceae; *Pinelliae rhizoma praeparatum*] 10g；<br>川芎（chuanxiong）：*Ligusticum chuanxiong* Hort. [Apiaceae; *Chuanxiong rhizoma*] 15g；<br>丹参（danshen）：*Salvia miltiorrhiza* Bunge [Lamiaceae; *Salviae miltiorrhizae radix et rhizoma*] 20g；<br>当归（danggui）：*Angelica sinensis* (Oliv.) Diels [Apiaceae; *Angelicae sinensis radix*] 10g；<br>降香（jiangxiang）：*Dalbergia odorifera* T.C.Chen [Fabaceae; *Dalbergiae odoriferae lignum*] 6g；<br>延胡索（yanhusuo）：*Corydalis yanhusuo* (Y.H.Chou & Chun C.Hsu) W.T.Wang ex Z.Y.Su & C.Y.Wu [Papaveraceae; *Corydalis rhizoma*] 15g；<br>甘草（gancao）：*Glycyrrhiza glabra* L. [Fabaceae; *Glycyrrhizae radix et rhizoma*] 5g.	One dose per day, administered as two equal divided doses taken orally on an empty stomach–morning and evening.	B

NO.	Author	Formulas and components	Usage	ConPhyMP Type
3	Cong, X. (2021)	活血安神解郁方（Huoxue Anshen Jieyu Decoction）：<br>柴胡（chaihu）：*Bupleurum chinense* DC. [Apiaceae; *Bupleuri radix*] 15g；<br>大枣（dazao）：*Ziziphus jujuba* Mill. [Rhamnaceae; *Jujubae fructus*] 6 pieces；<br>炒白术（chao baizhu）：*Atractylodes macrocephala* Koidz [Asteraceae; *Atractylodis macrocephalae rhizoma stir-fry*] 15g；<br>炙甘草(zhi gancao)：*Glycyrrhiza glabra* L. [Fabaceae; *Glycyrrhizae radix et rhizoma praeparata cum melle*] 9g；<br>炒栀子（chao zhizi）：*Gardenia jasminoides* J.Ellis [Rubiaceae; *Gardeniae fructus stir-fry*] 12g；<br>百合（baihe）：*Lilium lancifolium* Thunb. [Liliaceae; *Lilii bulbus*] 15g；<br>龙齿（longchi）：*Dens Draconis* (Fossilized Mammal Tooth, *Longchi*) 15g；<br>炒酸枣仁(chao suanzaoren)：*Ziziphus jujuba* Mill. [Paeoniaceae; *Ziziphi spinosae semen stir-fry*] 30g；<br>当归（danggui）：*Angelica sinensis* (Oliv.) Diels [Apiaceae; *Angelicae sinensis radix*] 15g；<br>川芎（chuanxiong）：*Ligusticum chuanxiong* Hort. [Apiaceae*; Chuanxiong rhizoma*] 15g；<br>桃仁（taoren）：*Prunus persica* (L.) Batsch [Rosaceae; *Persicae semen*] 9g；<br>红花（honghua）：*Carthamus tinctorius* L. [Asteraceae; *Carthami flos*] 6g；<br>佛手（foshou）：*Citrus medica* L. var. *sarcodactylis* Swingle [Rutaceae; *Citri sarcodactylis fructus*] 12g；<br>玫瑰花（meiguihua）：*Rosa rugosa Thunb.* [Rosaceae; *Rosae rugosae flos*] 12g	One dose daily, administered twice per day (morning and evening).	B
4	Fan, G. H. (2012)	麝香保心丸（Shexiang Baoxin Pills）：<br>人工麝香（rengong shexiang）：*Moschus Artificialis* (Synthetic Musk Compound, *Rengong Shexiang*)；<br>人参（renshen）：*Panax ginseng* C.A.Mey. [Araliaceae; *Ginseng radix et rhizoma*]；<br>人工牛黄（rengong niuhuang）：*Bovis Calculus Artifactus* (Artificial Bile Extract, *Rengong Shexiang*)；<br>肉桂（rougui）：*Cinnamomum cassia* (L.) Kosterm. [Lauraceae; *Cinnamomi cortex*]；<br>苏合香（suhexiang）：*Liquidambar orientalis* Mill. [Altingiaceae; *Styrax*]；<br>蟾酥（chansu）：*Venenum Bufonis* (Toad-cake; *Chansu*)；<br>冰片（bingpian）：*Dryobalanops aromatica* C.F.Gaertn. [Dipterocarpaceae; *Borneolum syntheticum*].	Take 2 tablets orally, three times daily.	A

NO.	Author	Formulas and components	Usage	ConPhyMP Type
5	Han, Y. (2022)	益气通脉汤（Yiqi Tongmai Decoction）：<br>太子参（taizishen）：*Pseudostellaria heterophylla* (Miq.) Pax [Caryophyllaceae; *Pseudostellariae radix*] 30g；<br>麦冬（maidong）：*Ophiopogon japonicus* (Thunb.) Ker-Gawl. [Asparagaceae; *Ophiopogonis radix*] 15g；<br>五味子（wuweizi）：*Schisandra chinensis* (Turcz.) Baill. [Schisandraceae; *Schisandrae chinensis fructus*] 10g；<br>川芎（chuanxiong）：*Ligusticum chuanxiong* Hort. [Apiaceae*; Chuanxiong rhizoma*] 15g；<br>丹参（danshen）：*Salvia miltiorrhiza* Bunge [Lamiaceae; *Salviae miltiorrhizae radix et rhizoma*] 30g；<br>香附（xiangfu）：*Cyperus rotundus* L. [Cyperaceae; *Cyperi rhizoma*] 10g；<br>乌药（wuyao）：*Lindera aggregata* (Sims) Kosterm. [Lauraceae; *Linderae radix*] 10g；<br>香橼（xiangyuan）：*Citrus medica* L. [Rutaceae; *Citri fructus*] 10g；<br>佛手（foshou）：*Citrus medica* L. var. *sarcodactylis* Swingle [Rutaceae; *Citri sarcodactylis fructus*] 10g	Take one dose daily (200 mL per dose), administered twice per day.	B
6	Li, J. (2014)	心可舒片（Xinkeshu Tablets）：<br>丹参（danshen）：*Salvia miltiorrhiza* Bunge [Lamiaceae; *Salviae miltiorrhizae radix et rhizoma*]；<br>葛根（gegen）：*Pueraria montana var. lobata* (Willd.) Maesen & S.M.Almeida ex Sanjappa & Predeep [Fabaceae; *Puerariae lobatae radix*]；<br>三七（sanqi）：*Panax notoginseng* (Burkill) F.H.Chen [Araliaceae; *Notoginseng radix et rhizoma*]；<br>山楂（shanzha）：*Crataegus pinnatifida Bunge* [Rosaceae; *Crataegi fructus*]；<br>木香（muxiang）：*Aucklandia costus* (Falc.) Kasana & A.K.Pandey [Asteraceae; *Aucklandiae radix*].	Take 4 tablets orally, three times daily.	A

NO.	Author	Formulas and components	Usage	ConPhyMP Type
7	Qiao, J. F. (2021)	益气畅脉饮（Yiqi Changmai Yin）：<br>党参（dangshen）：*Codonopsis pilosula* (Franch.) Nannf. [Campanulaceae; *Codonopsis radix*] 15g；<br>黄芪（huangqi）：*Astragalus mongholicus* Bunge [Fabaceae; *Astragali radix*] 30g；<br>当归（danggui）：*Angelica sinensis* (Oliv.) Diels [Apiaceae; *Angelicae sinensis radix*] 9g；<br>丹参（danshen）：*Salvia miltiorrhiza* Bunge [Lamiaceae; *Salviae miltiorrhizae radix et rhizoma*] 15g；<br>桃仁（taoren）：*Prunus persica* (L.) Batsch [Rosaceae; *Persicae semen*] 9g；<br>红花（honghua）：*Carthamus tinctorius* L. [Asteraceae; *Carthami flos*] 6g；<br>川芎（chuanxiong）：*Ligusticum chuanxiong* Hort. [Apiaceae; *Chuanxiong rhizoma*] 9g；<br>炙甘草(zhi gancao):*Glycyrrhiza glabra* L. [Fabaceae; *Glycyrrhizae radix et rhizoma praeparata cum melle*] 5g	Take one sachet twice daily (bid), dissolved in warm water 30 minutes after morning and evening meals.	B
8	Wang, J. G. (2016)	脑心通胶囊（Naoxintong Capsules）：<br>黄芪（huangqi）：*Astragalus mongholicus* Bunge [Fabaceae; *Astragali radix*]；<br>赤芍（chishao）：*Paeonia lactiflora* Pall. [Paeoniaceae; *Paeoniae radix rubra*]；<br>丹参（danshen）：*Salvia miltiorrhiza* Bunge [Lamiaceae; *Salviae miltiorrhizae radix et rhizoma*]；<br>当归（danggui）：*Angelica sinensis* (Oliv.) Diels [Apiaceae; *Angelicae sinensis radix*]；<br>川芎（chuanxiong）：*Ligusticum chuanxiong* Hort. [Apiaceae; *Chuanxiong rhizoma*]；<br>桃仁（taoren）：*Prunus persica* (L.) Batsch [Rosaceae; *Persicae semen*]；<br>红花（honghua）：*Carthamus tinctorius* L. [Asteraceae; *Carthami flos*]；<br>醋乳香（cu ruxiang）：*Boswellia sacra* Flück. [Burseraceae; *Olibanum acetatum*]；<br>醋没药（cu moyao）：*Commiphora myrrha* (Nees) Engl. [Burseraceae; *Myrrha acetatum*]；<br>鸡血藤（jixuetang）：*Spatholobus suberectus* Dunn [Leguminosae; *Spatholobi caulis*]；<br>牛膝（niuxi）：*Achyranthes bidentata* Blume [Amaranthaceae; *Achyranthis bidentatae radix*]；<br>桂枝（guizhi）：*Neolitsea cassia* (L.) Kosterm. [Lauraceae; *Cinnamomi ramulus*]；<br>桑枝（sangzhi）：*Morus alba* L. [Moraceae; *Mori ramulus*]；<br>地龙（dilong）：*Pheretima aspergillum* (Earthworm; *Dilong*)；<br>全蝎（quanxie）：*Mesobuthus martensii* (Scorpion; *Quanxie*)；<br>水蛭（shuizhi）：*Whitmania pigra* (Medicinal Leech; *Shuizhi*)	Take 3 capsules each time, three times daily.	A
9	Wang, J. (2016)	水蛭、苦参粉胶囊（Hirudo and Kushen Powder Capsules）（Ratio 1：10）：<br>水蛭（shuizhi）：*Whitmania pigra* (Medicinal Leech; *Shuizhi*)<br>苦参（kushen）：*Sophora flavescens* Aiton [Fabaceae; *Sophorae flavescentis radix*]	Take 3 to 5 capsules per dose, 2 to 3 times daily.	C

NO.	Author	Formulas and components	Usage	ConPhyMP Type
10	Wang, Z. X. (2020)	柴胡疏肝散（Modified Chaihu Shugan Powder）：<br>柴胡（chaihu）：*Bupleurum chinense* DC. [Apiaceae; *Bupleuri radix*] 12g；<br>丹参（danshen）：*Salvia miltiorrhiza* Bunge [Lamiaceae; *Salviae miltiorrhizae radix et rhizoma*] 15g；<br>川芎（chuanxiong）：*Ligusticum chuanxiong* Hort. [Apiaceae*; Chuanxiong rhizoma*] 9g；<br>香附（xiangfu）：*Cyperus rotundus* L. [Cyperaceae; *Cyperi rhizoma*] 9g；<br>白芍（baishao）：*Paeonia lactiflora* Pall. [Paeoniaceae; *Paeoniae radix alba*] 9g；<br>枳壳（zhiqiao）：*Citrus × aurantium* L. [Rutaceae; *Aurantii fructus*] 9g；<br>檀香（tanxiang）：*Santalum album* L. [Santalaceae; *Santali albi lignum*] 6g；<br>红花（honghua）：*Carthamus tinctorius* L. [Asteraceae; *Carthami flos*] 6g；<br>炙甘草(zhi gancao):*Glycyrrhiza glabra* L. [Fabaceae; *Glycyrrhizae radix et rhizoma praeparata cum melle*] 6g	Take twice daily, dissolved in warm water 30 minutes after breakfast and dinner.	B
11	Yin, X. S. (2021)	宽胸气雾剂（Wide Chest Aerosol）：<br>檀香（tanxiang）：*Santalum album* L. [Santalaceae; *Santali albi lignum*]；<br>荜茇（bibo）：*Piper longum* L. [Piperaceae; *Piperis longi fructus*]；<br>高良姜（gaoliangjiang）：*Alpinia officinarum* Hance [Zingiberaceae; *Alpiniae officinarum rhizoma*]；<br>细辛（xixin）：*Asarum heterotropoides* F.Schmidt [Aristolochiaceae; *Asari radix et rhizoma*]；<br>冰片（bingpian）：*Dryobalanops aromatica* C.F.Gaertn. [Dipterocarpaceae; *Borneolum syntheticum*].	2 sprays, twice daily.	A
12	Yuan, H. W. (2018)	三参三七蛭琥颗粒（Sanshen Sanqi Zhihu Granule）：<br>红参（hongshen）：*Panax ginseng* C.A.Mey. [Araliaceae; *Ginseng radix et rhizoma rubra*] 10g；<br>丹参（danshen）：*Salvia miltiorrhiza* Bunge [Lamiaceae; *Salviae miltiorrhizae radix et rhizoma*] 10g；<br>玄参（xuanshen）：*Scrophularia ningpoensis* Hemsl. [Scrophulariaceae; *Scrophulariae radix*] 10g；<br>三七（sanqi）：*Panax notoginseng* (Burkill) F.H.Chen [Araliaceae; *Notoginseng radix et rhizoma*] 3g；<br>水蛭（shuizhi）：*Whitmania pigra* (Medicinal Leech; *Shuizhi*) 3g；<br>琥珀（hupo）：*Succinum* (Fossilized Resin, *Hupo*) 3g；<br>绞股蓝（jiaogulan）：*Gynostemma pentaphyllum* (Thunb.) Makino [Cucurbitaceae; *Herba Gynostemmatis Pentaphylli*] 10g	Dissolve in boiling water and take in two divided doses.	B

NO.	Author	Formulas and components	Usage	ConPhyMP Type
13	Zhang, J. (2023)	复心合剂（Fuxin Heji Decoction）：<br>炙黄芪（zhi huangqi）：*Astragalus mongholicus* Bunge [Fabaceae; *Astragali radix praeparata cum melle*] 30g；<br>白术（baizhu）：*Atractylodes macrocephala* Koidz [Asteraceae; *Atractylodis macrocephalae rhizoma*] 15g；<br>附子（fuzi）：*Aconitum carmichaelii* Debeaux [Ranunculaceae; *Aconiti lateralis radix praeparata*] 15g；<br>当归（danggui）：*Angelica sinensis* (Oliv.) Diels [Apiaceae; *Angelicae sinensis radix*]12g；<br>泽泻（zexie）：*Alisma orientale* (Sam.) Juz. [Alismataceae; *Alismatis rhizoma*] 12g；<br>党参（dangshen）：*Codonopsis pilosula* (Franch.) Nannf. [Campanulaceae; *Codonopsis radix*] 12g；<br>赤芍（chishao）：*Paeonia lactiflora* Pall. [Paeoniaceae; *Paeoniae radix rubra*] 12g；<br>川芎（chuanxiong）：*Ligusticum chuanxiong* Hort. [Apiaceae; *Chuanxiong rhizoma*] 12g；<br>丹参（danshen）：*Salvia miltiorrhiza* Bunge [Lamiaceae; *Salviae miltiorrhizae radix et rhizoma*] 9g；<br>柴胡（chaihu）：*Bupleurum chinense* DC. [Apiaceae; *Bupleuri radix*] 9g；<br>白芍（baishao）：*Paeonia lactiflora* Pall. [Paeoniaceae; *Paeoniae radix alba*] 9g；<br>木香（muxiang）：*Aucklandia costus* (Falc.) Kasana & A.K.Pandey [Asteraceae; *Aucklandiae radix*] 9g；<br>升麻（shengma）：*Actaea cimicifuga* L. [Ranunculaceae; *Cimicifugae rhizoma*] 9g；<br>麦冬（maidong）：*Ophiopogon japonicus* (Thunb.) Ker-Gawl. [Asparagaceae; *Ophiopogonis radix*] 9g；<br>地黄（shu dihuang）：*Rehmannia glutinosa* (Gaertn.) Libosch. ex DC. [Orobanchaceae; *Rehmanniae radix praeparata*]9g；<br>陈皮（chenpi）：*Citrus reticulata* Blanco [Rutaceae; *Citri reticulatae pericarpium*]9g；<br>炙甘草（zhi gancao）：*Glycyrrhiza glabra* L. [Fabaceae; *Glycyrrhizae radix et rhizoma praeparata cum melle*] 6g	400 mL total, divided into two 200 mL doses taken warm after morning and evening meals.	B
14	Zhang, P. (2016)	芪参益气滴丸（Qishen Yiqi Dropping）：<br>黄芪（huangqi）：*Astragalus mongholicus* Bunge [Fabaceae; *Astragali radix*]；<br>丹参（danshen）：*Salvia miltiorrhiza* Bunge [Lamiaceae; *Salviae miltiorrhizae radix et rhizoma*]；<br>三七（sanqi）：*Panax notoginseng* (Burkill) F.H.Chen [Araliaceae; *Notoginseng radix et rhizoma*]；<br>降香（jiangxiang）：*Dalbergia odorifera* T.C.Chen [Fabaceae; *Dalbergiae odoriferae lignum*].	Take one 0.5g sachet per dose, three times daily.	A

NO.	Author	Formulas and components	Usage	ConPhyMP Type
15	Zhao, M. J. (2011)	通心络胶囊（Tongxinluo Capsules）：<br>人参（renshen）：*Panax ginseng* C.A.Mey. [Araliaceae; *Ginseng radix et rhizoma*];<br>水蛭（shuizhi）：*Whitmania pigra* (Medicinal Leech; *Shuizhi*);<br>全蝎（quanxie）：*Mesobuthus martensii* (Scorpion; *Quanxie*);<br>赤芍（chishao）：*Paeonia lactiflora* Pall. [Paeoniaceae; *Paeoniae radix rubra*];<br>蝉蜕（chantui）：*Periostracum Cicadae* (Cicada Slough; *Chantui*);<br>土鳖虫（tubiechong）：*Eupolyphaga sinensis* (Ground Beetle; *Tubiechong*);<br>蜈蚣（wugong）：*Scolopendra subspinipes mutilans* (Centipede; *Wugong*);<br>檀香（tanxiang）：*Santalum album* L. [Santalaceae; *Santali albi lignum*];<br>降香（jiangxiang）：*Dalbergia odorifera* T.C.Chen [Fabaceae; *Dalbergiae odoriferae lignum*];<br>制乳香（zhi ruxiang）：*Boswellia sacra* Flück. [Burseraceae; *Olibanum praeparatum*];<br>炒酸枣仁（chao suanzaoren）：*Ziziphus jujuba* Mill. [Paeoniaceae; *Ziziphi spinosae semen stir-fry*];<br>冰片（bingpian）：*Dryobalanops aromatica* C.F.Gaertn. [Dipterocarpaceae; *Borneolum syntheticum*].	Take 4 tablets orally, three times daily.	A
16	Zheng, Y. H. (2012)	顺气通脉胶囊（Shunqi Tongmai Capsules）：<br>当归（danggui）：*Angelica sinensis* (Oliv.) Diels [Apiaceae; *Angelicae sinensis radix*]15g;<br>川芎（chuanxiong）：*Ligusticum chuanxiong* Hort. [Apiaceae; *Chuanxiong rhizoma*] 10g;<br>丹参（danshen）：*Salvia miltiorrhiza* Bunge [Lamiaceae; *Salviae miltiorrhizae radix et rhizoma*] 15g;<br>莪术（ezhu）：*Curcuma phaeocaulis* Valeton [Zingiberaceae; *Curcumae rhizoma*] 10g;<br>延胡索（yanhusuo）：*Corydalis yanhusuo* (Y.H.Chou & Chun C.Hsu) W.T.Wang ex Z.Y.Su & C.Y.Wu [Papaveraceae; *Corydalis rhizoma*] 15g;<br>瓜蒌（gualou）：*Trichosanthes kirilowii* Maxim. [Cucurbitaceae; *Trichosanthis fructus*] 30g;<br>枳壳（zhiqiao）：*Citrus × aurantium* L. [Rutaceae; *Aurantii fructus*] 10g	Take 3 tablets orally, three times daily.	B

NO.	Author	Formulas and components	Usage	ConPhyMP Type
17	Zhong, Q. Y. (2019)	柴胡疏肝散（Modified Chaihu Shugan Powder）：<br>柴胡（chaihu）：*Bupleurum chinense* DC. [Apiaceae; *Bupleuri radix*] 12g；<br>丹参（danshen）：*Salvia miltiorrhiza* Bunge [Lamiaceae; *Salviae miltiorrhizae radix et rhizoma*] 15g；<br>川芎（chuanxiong）：*Ligusticum chuanxiong* Hort. [Apiaceae; *Chuanxiong rhizoma*] 9g；<br>香附（xiangfu）：*Cyperus rotundus* L. [Cyperaceae; *Cyperi rhizoma*] 9g；<br>枳壳（zhiqiao）：*Citrus × aurantium* L. [Rutaceae; *Aurantii fructus*] 9g；<br>白芍（baishao）：*Paeonia lactiflora* Pall. [Paeoniaceae; *Paeoniae radix alba*] 9g；<br>红花（honghua）：*Carthamus tinctorius* L. [Asteraceae; *Carthami flos*] 6g；<br>檀香（tanxiang）：*Santalum album* L. [Santalaceae; *Santali albi lignum*] 6g；<br>炙甘草(zhi gancao)：*Glycyrrhiza glabra* L. [Fabaceae; *Glycyrrhizae radix et rhizoma praeparata cum melle*] 6g	Take one dose daily (2 sachets total), divided into two administrations (1 sachet per dose) dissolved in warm water 30 minutes after breakfast and dinner.	B
18	Zhu, G. Y. (2014)	营心宁胶囊（Yingxinning Capsules）：<br>藿香（huoxiang）：*Pogostemon cablin* (Blanco) Benth. [Lamiaceae; *Pogostemonis herba*]；<br>枳实（zhishi）：*Citrus × aurantium* f. *aurantium* [Rutaceae; *Aurantii fructus immaturus*]；<br>三七(sanqi)：*Panax notoginseng* (Burkill) F.H.Chen [Araliaceae; *Notoginseng radix et rhizoma*；<br>薄荷（bohe）：*Mentha canadensis* L. [Lamiaceae; *Menthae haplocalycis herba*]；<br>红参（hongshen）：*Panax ginseng* C.A.Mey. [Araliaceae; *Ginseng radix et rhizoma rubra*]；<br>川芎（chuanxiong）：*Ligusticum chuanxiong* Hort. [Apiaceae; *Chuanxiong rhizoma*].	Take 4 tablets orally, three times daily.	B
