# Supplementary material for: Efficacy and safety of Traditional Chinese Medicine in alleviating symptoms associated with myocardial bridge: a systematic review and meta-analysis
Source: Front Pharmacol. 2025 Sep 19;16:1619617. doi: 10.3389/fphar.2025.1619617 (PMC12492955; doi:10.3389/fphar.2025.1619617)

Sensitivity and Subgroup Analysis

S1. Angina Efficacy Analysis based on Subgroups of Treatment Duration

S1.1 30 days

Study or Subgroup	Intervention Events	Intervention Total	Control Events	Control Total	Weight	Risk Ratio M-H, Fixed, 95% CI
**≤30 days**						
Chen, J. (2017)	57	60	47	60	18.2%	1.21 [1.05, 1.40]
Fan, G. H. (2012)	33	36	23	36	8.9%	1.43 [1.10, 1.87]
Wang, J. (2016)	26	30	23	30	8.9%	1.13 [0.89, 1.44]
Wang, Z. X. (2020)	23	25	17	25	6.6%	1.35 [1.01, 1.81]
Zhu, G. Y. (2014)	13	15	9	15	3.5%	1.44 [0.91, 2.28]
**Subtotal (95% CI)**		**166**		**166**	**46.0%**	**1.28 [1.15, 1.42]**
Total events	152		119			
Heterogeneity: Chi² = 2.63, df = 4 (P = 0.62); I² = 0%						
Test for overall effect: Z = 4.53 (P < 0.00001)						
**>30 days**						
Han, Y. (2022)	27	32	17	32	6.6%	1.59 [1.11, 2.27]
Li, J. (2014)	28	30	21	30	8.1%	1.33 [1.04, 1.72]
Qiao, J. F. (2021)	28	30	21	30	8.1%	1.33 [1.04, 1.72]
Yuan, H. W. (2018)	32	34	23	34	8.9%	1.39 [1.09, 1.78]
Zhang, P. (2016)	25	30	17	30	6.6%	1.47 [1.03, 2.09]
Zhao, M. J. (2011)	17	20	15	18	6.1%	1.02 [0.77, 1.35]
Zhong, Q. Y. (2019)	29	30	25	30	9.7%	1.16 [0.98, 1.38]
**Subtotal (95% CI)**		**206**		**204**	**54.0%**	**1.32 [1.19, 1.47]**
Total events	186		139			
Heterogeneity: Chi² = 7.15, df = 6 (P = 0.31); I² = 16%						
Test for overall effect: Z = 5.36 (P < 0.00001)						
**Total (95% CI)**		**372**		**370**	**100.0%**	**1.30 [1.21, 1.40]**
Total events	338		258			
Heterogeneity: Chi² = 9.71, df = 11 (P = 0.56); I² = 0%						
Test for overall effect: Z = 7.02 (P < 0.00001)						
Test for subgroup differences: Chi² = 0.23, df = 1 (P = 0.63), I² = 0%						

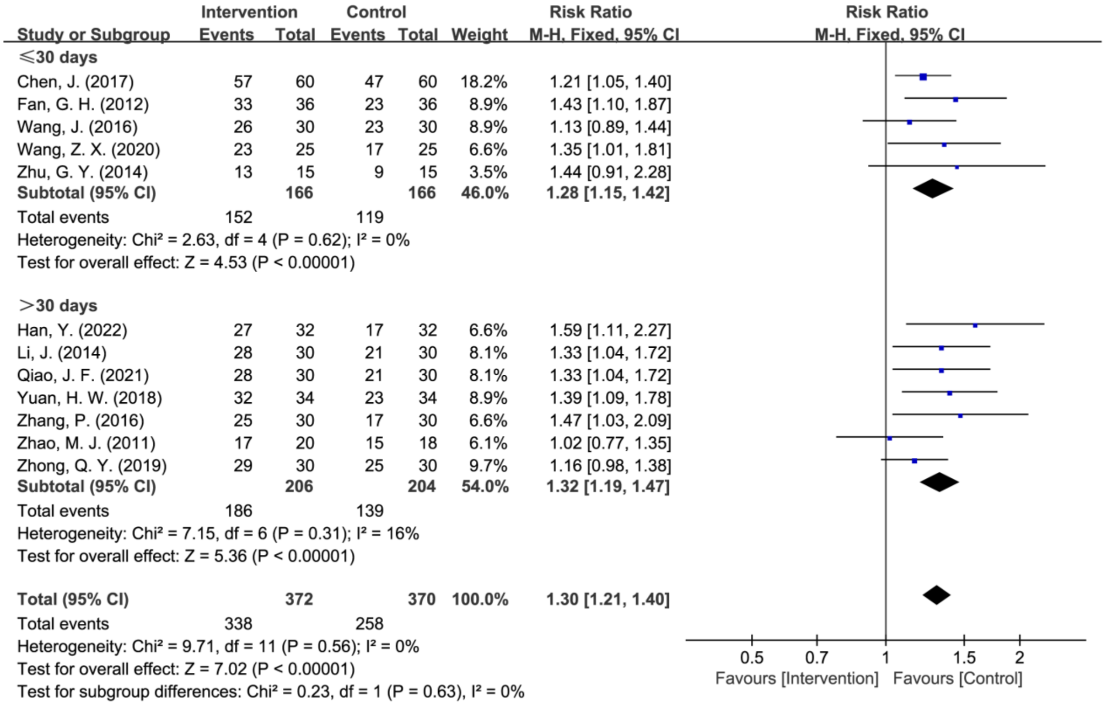

S1.2 45 days

Study or Subgroup	Intervention Events	Intervention Total	Control Events	Control Total	Weight	Risk Ratio M-H, Fixed, 95% CI
**≤45 days**						
Chen, J. (2017)	57	60	47	60	18.2%	1.21 [1.05, 1.40]
Fan, G. H. (2012)	33	36	23	36	8.9%	1.43 [1.10, 1.87]
Wang, J. (2016)	26	30	23	30	8.9%	1.13 [0.89, 1.44]
Wang, Z. X. (2020)	23	25	17	25	6.6%	1.35 [1.01, 1.81]
Zhao, M. J. (2011)	17	20	15	18	6.1%	1.02 [0.77, 1.35]
Zhong, Q. Y. (2019)	29	30	25	30	9.7%	1.16 [0.98, 1.38]
Zhu, G. Y. (2014)	13	15	9	15	3.5%	1.44 [0.91, 2.28]
**Subtotal (95% CI)**		**216**		**214**	**61.7%**	**1.23 [1.13, 1.35]**
Total events	198		159			
Heterogeneity: Chi² = 4.94, df = 6 (P = 0.55); I² = 0%						
Test for overall effect: Z = 4.67 (P < 0.00001)						
**>45 days**						
Han, Y. (2022)	27	32	17	32	6.6%	1.59 [1.11, 2.27]
Li, J. (2014)	28	30	21	30	8.1%	1.33 [1.04, 1.72]
Qiao, J. F. (2021)	28	30	21	30	8.1%	1.33 [1.04, 1.72]
Yuan, H. W. (2018)	32	34	23	34	8.9%	1.39 [1.09, 1.78]
Zhang, P. (2016)	25	30	17	30	6.6%	1.47 [1.03, 2.09]
**Subtotal (95% CI)**		**156**		**156**	**38.3%**	**1.41 [1.24, 1.61]**
Total events	140		99			
Heterogeneity: Chi² = 0.88, df = 4 (P = 0.93); I² = 0%						
Test for overall effect: Z = 5.25 (P < 0.00001)						
**Total (95% CI)**		**372**		**370**	**100.0%**	**1.30 [1.21, 1.40]**
Total events	338		258			
Heterogeneity: Chi² = 9.71, df = 11 (P = 0.56); I² = 0%						
Test for overall effect: Z = 7.02 (P < 0.00001)						
Test for subgroup differences: Chi² = 2.93, df = 1 (P = 0.09), I² = 65.9%						

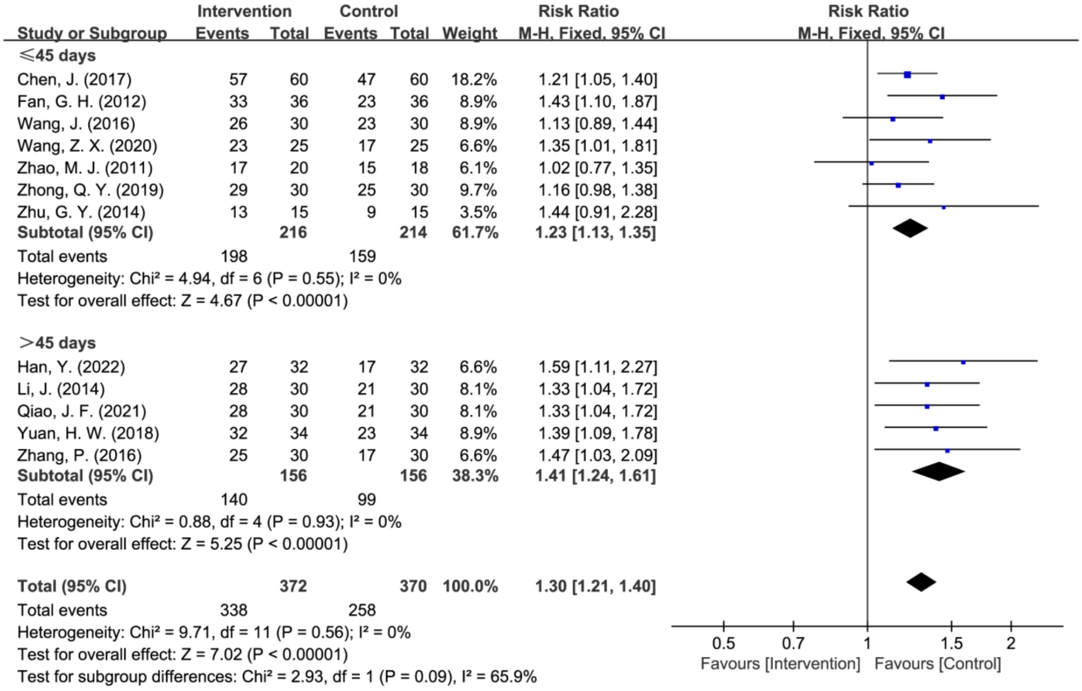

S1.3 60 days

Study or Subgroup	Intervention Events	Intervention Total	Control Events	Control Total	Weight	Risk Ratio M-H, Fixed, 95% CI
**≤60 days**						
Chen, J. (2017)	57	60	47	60	18.2%	1.21 [1.05, 1.40]
Fan, G. H. (2012)	33	36	23	36	8.9%	1.43 [1.10, 1.87]
Han, Y. (2022)	27	32	17	32	6.6%	1.59 [1.11, 2.27]
Qiao, J. F. (2021)	28	30	21	30	8.1%	1.33 [1.04, 1.72]
Wang, J. (2016)	26	30	23	30	8.9%	1.13 [0.89, 1.44]
Wang, Z. X. (2020)	23	25	17	25	6.6%	1.35 [1.01, 1.81]
Zhang, P. (2016)	25	30	17	30	6.6%	1.47 [1.03, 2.09]
Zhao, M. J. (2011)	17	20	15	18	6.1%	1.02 [0.77, 1.35]
Zhong, Q. Y. (2019)	29	30	25	30	9.7%	1.16 [0.98, 1.38]
Zhu, G. Y. (2014)	13	15	9	15	3.5%	1.44 [0.91, 2.28]
**Subtotal (95% CI)**		**308**		**306**	**83.0%**	**1.29 [1.19, 1.40]**
Total events	278		214			
Heterogeneity: Chi² = 8.90, df = 9 (P = 0.45); I² = 0%						
Test for overall effect: Z = 6.14 (P < 0.00001)						
**>60 days**						
Li, J. (2014)	28	30	21	30	8.1%	1.33 [1.04, 1.72]
Yuan, H. W. (2018)	32	34	23	34	8.9%	1.39 [1.09, 1.78]
**Subtotal (95% CI)**		**64**		**64**	**17.0%**	**1.36 [1.14, 1.63]**
Total events	60		44			
Heterogeneity: Chi² = 0.06, df = 1 (P = 0.81); I² = 0%						
Test for overall effect: Z = 3.44 (P = 0.0006)						
**Total (95% CI)**		**372**		**370**	**100.0%**	**1.30 [1.21, 1.40]**
Total events	338		258			
Heterogeneity: Chi² = 9.71, df = 11 (P = 0.56); I² = 0%						
Test for overall effect: Z = 7.02 (P < 0.00001)						
Test for subgroup differences: Chi² = 0.31, df = 1 (P = 0.58), I² = 0%						

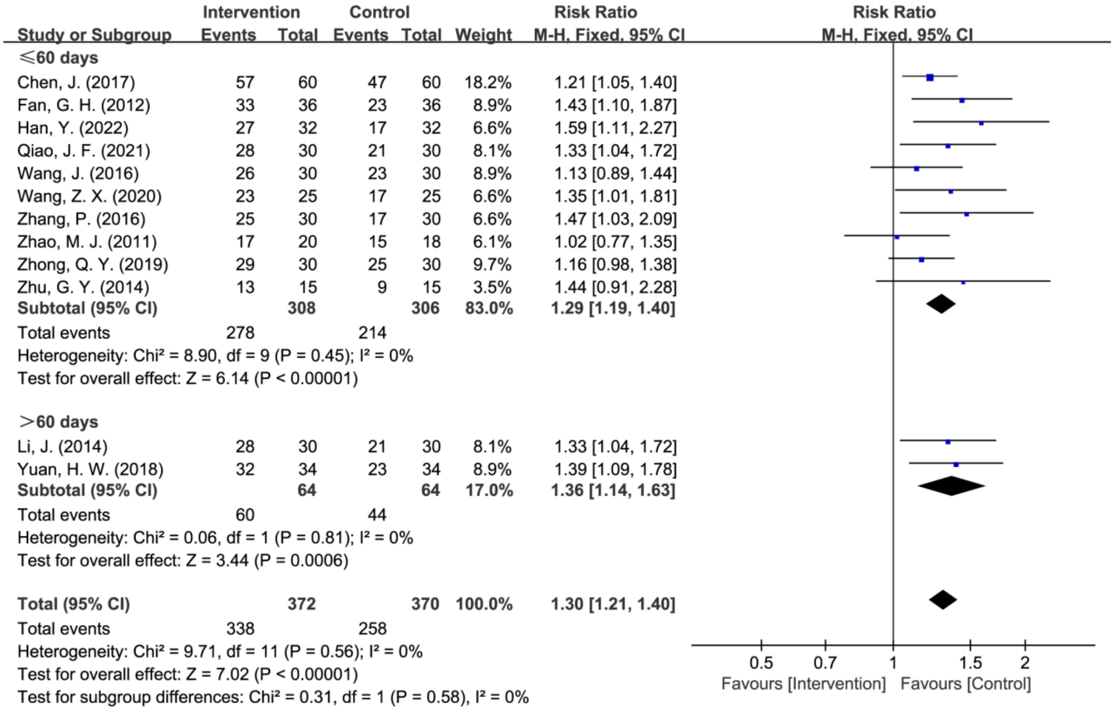

S1.4 30, 30-60, 60 days

Study or Subgroup	Intervention Events	Intervention Total	Control Events	Control Total	Weight	Risk Ratio M-H, Fixed, 95% CI
**≤30 days**						
Chen, J. (2017)	57	60	47	60	18.2%	1.21 [1.05, 1.40]
Fan, G. H. (2012)	33	36	23	36	8.9%	1.43 [1.10, 1.87]
Wang, J. (2016)	26	30	23	30	8.9%	1.13 [0.89, 1.44]
Wang, Z. X. (2020)	23	25	17	25	6.6%	1.35 [1.01, 1.81]
Zhu, G. Y. (2014)	13	15	9	15	3.5%	1.44 [0.91, 2.28]
**Subtotal (95% CI)**		**166**		**166**	**46.0%**	**1.28 [1.15, 1.42]**
Total events	152		119			
Heterogeneity: Chi² = 2.63, df = 4 (P = 0.62); I² = 0%						
Test for overall effect: Z = 4.53 (P < 0.00001)						
**30< days ≤60**						
Han, Y. (2022)	27	32	17	32	6.6%	1.59 [1.11, 2.27]
Qiao, J. F. (2021)	28	30	21	30	8.1%	1.33 [1.04, 1.72]
Zhang, P. (2016)	25	30	17	30	6.6%	1.47 [1.03, 2.09]
Zhao, M. J. (2011)	17	20	15	18	6.1%	1.02 [0.77, 1.35]
Zhong, Q. Y. (2019)	29	30	25	30	9.7%	1.16 [0.98, 1.38]
**Subtotal (95% CI)**		**142**		**140**	**37.0%**	**1.31 [1.15, 1.48]**
Total events	126		95			
Heterogeneity: Chi² = 6.48, df = 4 (P = 0.17); I² = 38%						
Test for overall effect: Z = 4.15 (P < 0.0001)						
**3.10.3 >60 days**						
Li, J. (2014)	28	30	21	30	8.1%	1.33 [1.04, 1.72]
Yuan, H. W. (2018)	32	34	23	34	8.9%	1.39 [1.09, 1.78]
**Subtotal (95% CI)**		**64**		**64**	**17.0%**	**1.36 [1.14, 1.63]**
Total events	60		44			
Heterogeneity: Chi² = 0.06, df = 1 (P = 0.81); I² = 0%						
Test for overall effect: Z = 3.44 (P = 0.0006)						
**Total (95% CI)**		**372**		**370**	**100.0%**	**1.30 [1.21, 1.40]**
Total events	338		258			
Heterogeneity: Chi² = 9.71, df = 11 (P = 0.56); I² = 0%						
Test for overall effect: Z = 7.02 (P < 0.00001)						
Test for subgroup differences: Chi² = 0.39, df = 2 (P = 0.82), I² = 0%						

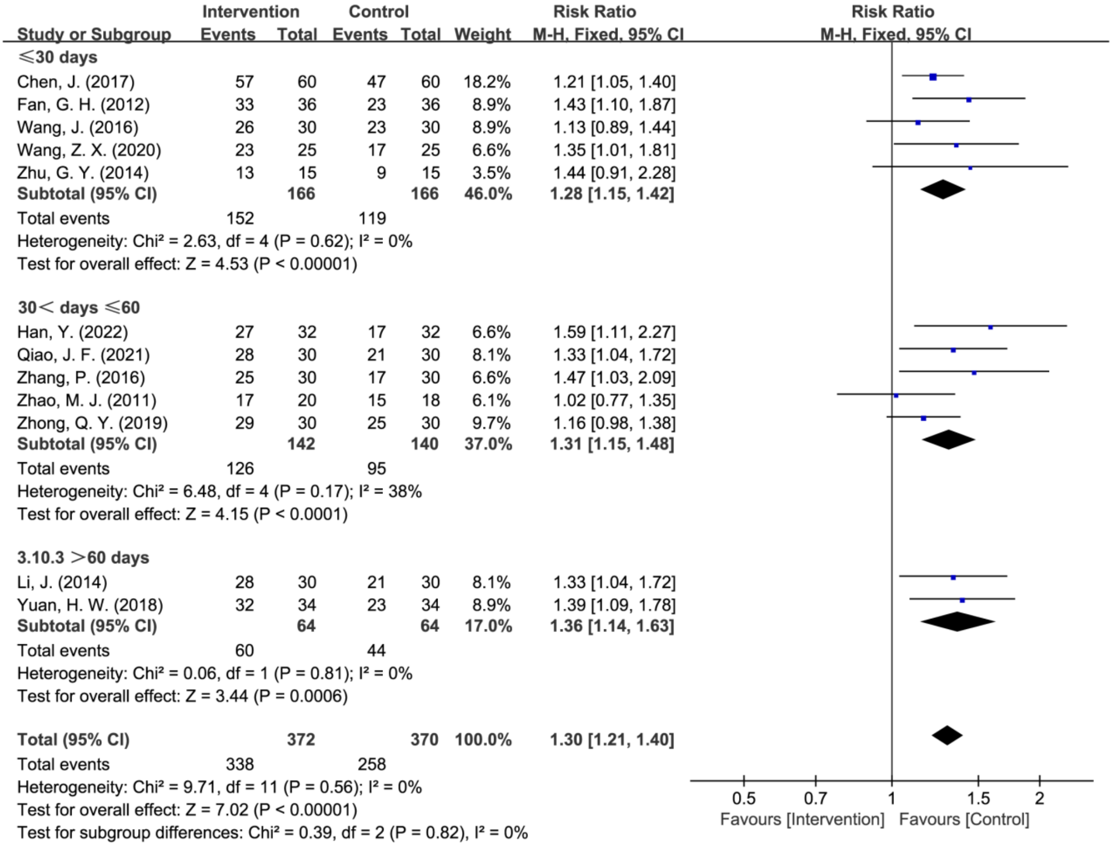

Sensitivity Analysis (Random-Effects Model)

Study or Subgroup	Intervention Events	Intervention Total	Control Events	Control Total	Weight	Risk Ratio M-H, Random, 95% CI
**≤30 days**						
Chen, J. (2017)	57	60	47	60	23.1%	1.21 [1.05, 1.40]
Fan, G. H. (2012)	33	36	23	36	7.0%	1.43 [1.10, 1.87]
Wang, J. (2016)	26	30	23	30	8.3%	1.13 [0.89, 1.44]
Wang, Z. X. (2020)	23	25	17	25	5.7%	1.35 [1.01, 1.81]
Zhu, G. Y. (2014)	13	15	9	15	2.3%	1.44 [0.91, 2.28]
**Subtotal (95% CI)**		**166**		**166**	**46.4%**	**1.26 [1.13, 1.39]**
Total events	152		119			
Heterogeneity: Tau^2 = 0.00; Chi^2 = 2.63, df = 4 (P = 0.62); I^2 = 0%						
Test for overall effect: Z = 4.35 (P < 0.0001)						
**30＜ days ≤60**						
Han, Y. (2022)	27	32	17	32	3.8%	1.59 [1.11, 2.27]
Qiao, J. F. (2021)	28	30	21	30	7.6%	1.33 [1.04, 1.72]
Zhang, P. (2016)	25	30	17	30	3.9%	1.47 [1.03, 2.09]
Zhao, M. J. (2011)	17	20	15	18	6.4%	1.02 [0.77, 1.35]
Zhong, Q. Y. (2019)	29	30	25	30	16.2%	1.16 [0.98, 1.38]
**Subtotal (95% CI)**		**142**		**140**	**38.0%**	**1.26 [1.08, 1.46]**
Total events	126		95			
Heterogeneity: Tau^2 = 0.01; Chi^2 = 6.48, df = 4 (P = 0.17); I^2 = 38%						
Test for overall effect: Z = 2.94 (P = 0.003)						
**＞60 days**						
Li, J. (2014)	28	30	21	30	7.6%	1.33 [1.04, 1.72]
Yuan, H. W. (2018)	32	34	23	34	8.0%	1.39 [1.09, 1.78]
**Subtotal (95% CI)**		**64**		**64**	**15.6%**	**1.36 [1.14, 1.63]**
Total events	60		44			
Heterogeneity: Tau^2 = 0.00; Chi^2 = 0.06, df = 1 (P = 0.81); I^2 = 0%						
Test for overall effect: Z = 3.43 (P = 0.0006)						
**Total (95% CI)**		**372**		**370**	**100.0%**	**1.26 [1.18, 1.36]**
Total events	338		258			
Heterogeneity: Tau^2 = 0.00; Chi^2 = 9.71, df = 11 (P = 0.56); I^2 = 0%						
Test for overall effect: Z = 6.57 (P < 0.00001)						
Test for subgroup differences: Chi^2 = 0.66, df = 2 (P = 0.72), I^2 = 0%						

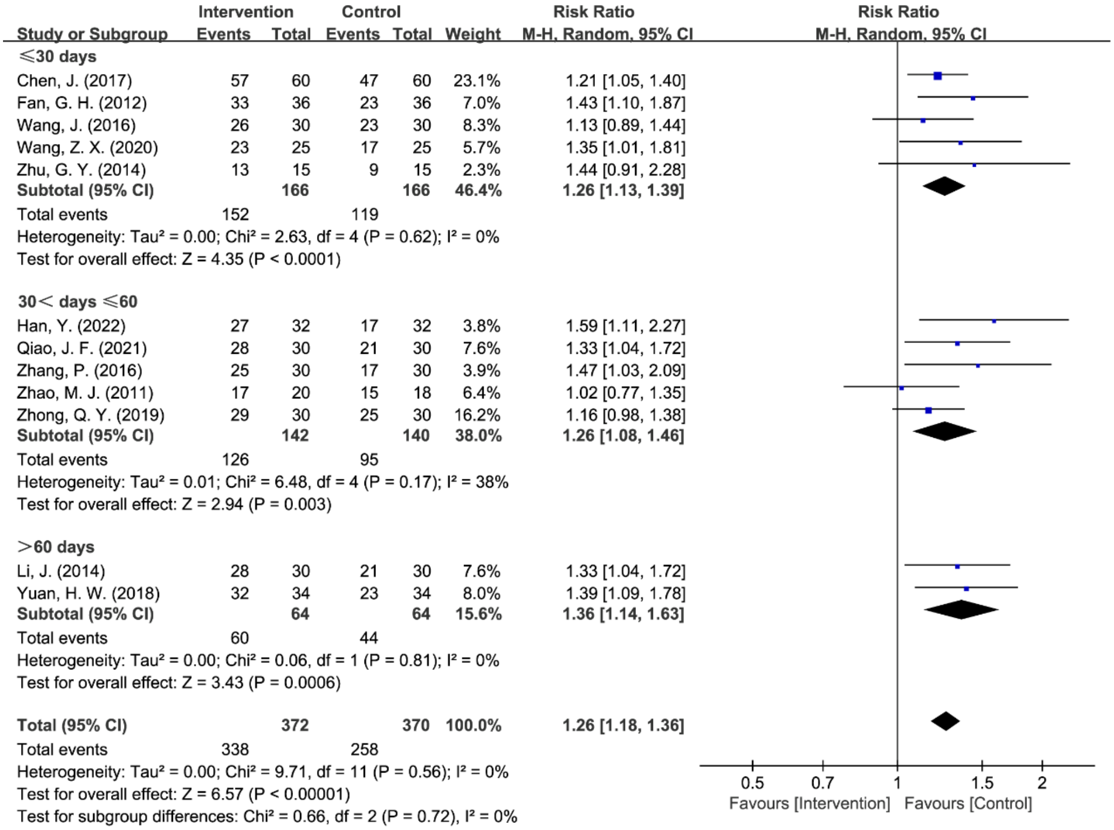

S1.5 30, 30-45, 45-60, 60 days

Study or Subgroup	Intervention Events	Intervention Total	Control Events	Control Total	Weight	Risk Ratio M-H, Fixed, 95% CI
**≤30 days**						
Chen, J. (2017)	57	60	47	60	18.2%	1.21 [1.05, 1.40]
Fan, G. H. (2012)	33	36	23	36	8.9%	1.43 [1.10, 1.87]
Wang, J. (2016)	26	30	23	30	8.9%	1.13 [0.89, 1.44]
Wang, Z. X. (2020)	23	25	17	25	6.6%	1.35 [1.01, 1.81]
Zhu, G. Y. (2014)	13	15	9	15	3.5%	1.44 [0.91, 2.28]
**Subtotal (95% CI)**		**166**		**166**	**46.0%**	**1.28 [1.15, 1.42]**
Total events	152		119			
Heterogeneity: Chi² = 2.63, df = 4 (P = 0.62); I² = 0%						
Test for overall effect: Z = 4.53 (P < 0.00001)						
**30< days ≤45**						
Zhao, M. J. (2011)	17	20	15	18	6.1%	1.02 [0.77, 1.35]
Zhong, Q. Y. (2019)	29	30	25	30	9.7%	1.16 [0.98, 1.38]
**Subtotal (95% CI)**		**50**		**48**	**15.8%**	**1.11 [0.95, 1.28]**
Total events	46		40			
Heterogeneity: Chi² = 0.62, df = 1 (P = 0.43); I² = 0%						
Test for overall effect: Z = 1.32 (P = 0.19)						
**45< days ≤60**						
Han, Y. (2022)	27	32	17	32	6.6%	1.59 [1.11, 2.27]
Qiao, J. F. (2021)	28	30	21	30	8.1%	1.33 [1.04, 1.72]
Zhang, P. (2016)	25	30	17	30	6.6%	1.47 [1.03, 2.09]
**Subtotal (95% CI)**		**92**		**92**	**21.3%**	**1.45 [1.21, 1.75]**
Total events	80		55			
Heterogeneity: Chi² = 0.69, df = 2 (P = 0.71); I² = 0%						
Test for overall effect: Z = 3.99 (P < 0.0001)						
**>60 days**						
Li, J. (2014)	28	30	21	30	8.1%	1.33 [1.04, 1.72]
Yuan, H. W. (2018)	32	34	23	34	8.9%	1.39 [1.09, 1.78]
**Subtotal (95% CI)**		**64**		**64**	**17.0%**	**1.36 [1.14, 1.63]**
Total events	60		44			
Heterogeneity: Chi² = 0.06, df = 1 (P = 0.81); I² = 0%						
Test for overall effect: Z = 3.44 (P = 0.0006)						
**Total (95% CI)**		**372**		**370**	**100.0%**	**1.30 [1.21, 1.40]**
Total events	338		258			
Heterogeneity: Chi² = 9.71, df = 11 (P = 0.56); I² = 0%						
Test for overall effect: Z = 7.02 (P < 0.00001)						
Test for subgroup differences: Chi² = 6.00, df = 3 (P = 0.11), I² = 50.0%						

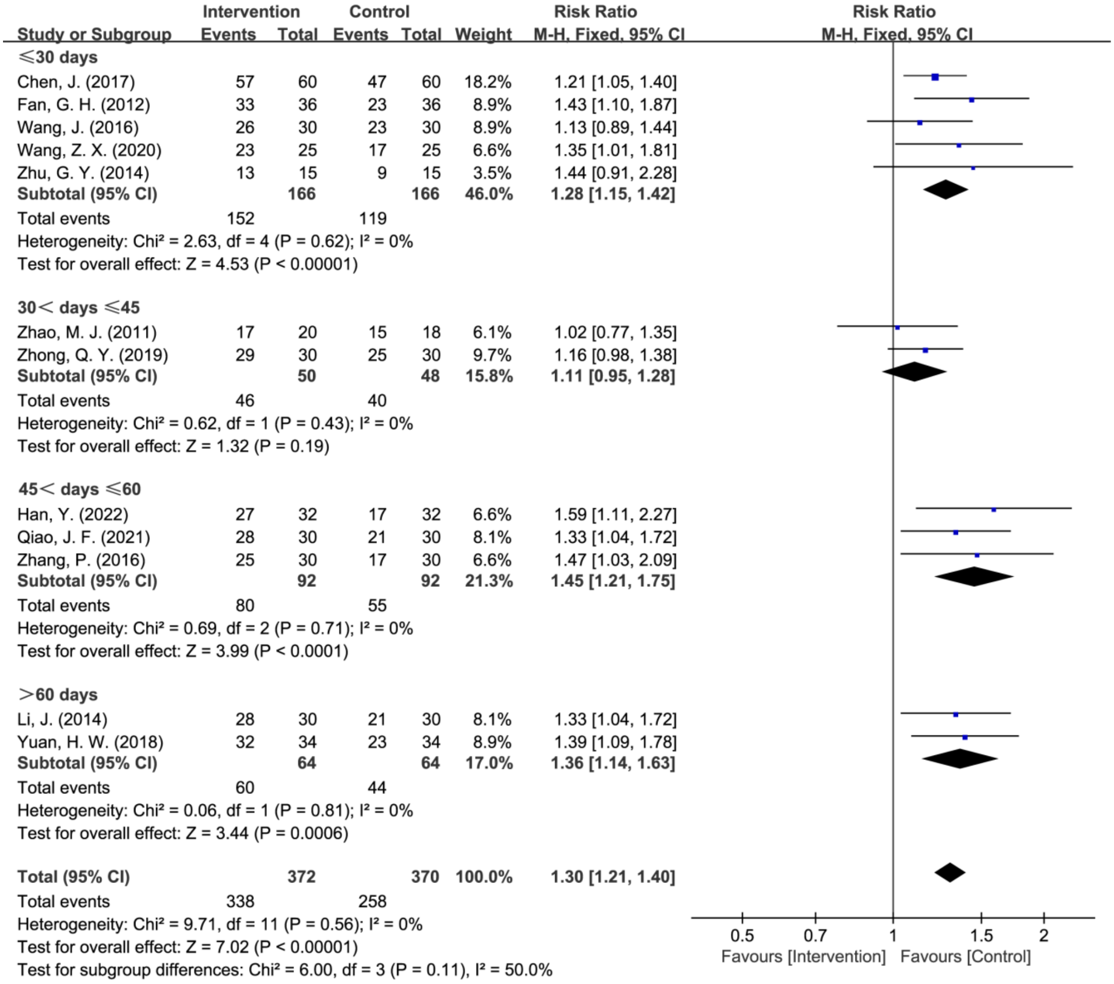

S2. Sensitivity Analysis for Deleting Wang, Z. X. (2020)' High-risk Articles

S2.1 Angina Efficacy Analysis

Study or Subgroup	Intervention Events	Intervention Total	Control Events	Control Total	Weight	Risk Ratio M-H, Fixed, 95% CI
Chen, J. (2017)	57	60	47	60	19.4%	1.21 [1.05, 1.40]
Fan, G. H. (2012)	33	36	23	36	9.5%	1.43 [1.10, 1.87]
Han, Y. (2022)	27	32	17	32	7.0%	1.59 [1.11, 2.27]
Li, J. (2014)	28	30	21	30	8.7%	1.33 [1.04, 1.72]
Qiao, J. F. (2021)	28	30	21	30	8.7%	1.33 [1.04, 1.72]
Wang, J. (2016)	26	30	23	30	9.5%	1.13 [0.89, 1.44]
Yuan, H. W. (2018)	32	34	23	34	9.5%	1.39 [1.09, 1.78]
Zhang, P. (2016)	25	30	17	30	7.0%	1.47 [1.03, 2.09]
Zhao, M. J. (2011)	17	20	15	18	6.5%	1.02 [0.77, 1.35]
Zhong, Q. Y. (2019)	29	30	25	30	10.3%	1.16 [0.98, 1.38]
Zhu, G. Y. (2014)	13	15	9	15	3.7%	1.44 [0.91, 2.28]
**Total (95% CI)**		**347**		**345**	**100.0%**	**1.30 [1.20, 1.40]**
Total events	315		241			

Heterogeneity: Chi² = 9.52, df = 10 (P = 0.48); I² = 0%
Test for overall effect: Z = 6.72 (P < 0.00001)

Risk Ratio M-H, Fixed, 95% CI

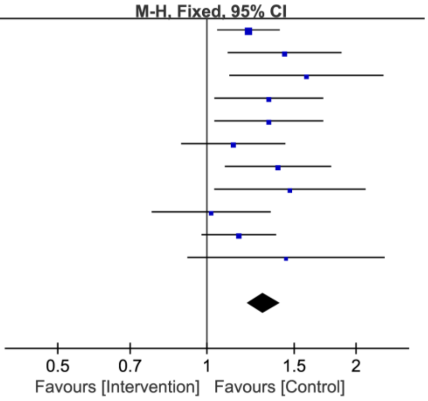

S2.2 Angina Efficacy Analysis based on Subgroup Analysis of Intervention Strategies

Angina Efficacy Study or Subgroup	Intervention Events	Intervention Total	Control Events	Control Total	Weight	Risk Ratio M-H, Fixed, 95% CI
**TCM+BM**						
Fan, G. H. (2012)	33	36	23	36	9.5%	1.43 [1.10, 1.87]
Han, Y. (2022)	27	32	17	32	7.0%	1.59 [1.11, 2.27]
Li, J. (2014)	28	30	21	30	8.7%	1.33 [1.04, 1.72]
Wang, J. (2016)	26	30	23	30	9.5%	1.13 [0.89, 1.44]
Yuan, H. W. (2018)	32	34	23	34	9.5%	1.39 [1.09, 1.78]
Zhang, P. (2016)	25	30	17	30	7.0%	1.47 [1.03, 2.09]
Zhu, G. Y. (2014)	13	15	9	15	3.7%	1.44 [0.91, 2.28]
**Subtotal (95% CI)**		**207**		**207**	**55.0%**	**1.38 [1.24, 1.55]**
Total events	184		133			
Heterogeneity: Chi² = 3.55, df = 6 (P = 0.74); I² = 0%						
Test for overall effect: Z = 5.68 (P < 0.00001)						
**TCM-alone**						
Chen, J. (2017)	57	60	47	60	19.4%	1.21 [1.05, 1.40]
Qiao, J. F. (2021)	28	30	21	30	8.7%	1.33 [1.04, 1.72]
Zhao, M. J. (2011)	17	20	15	18	6.5%	1.02 [0.77, 1.35]
Zhong, Q. Y. (2019)	29	30	25	30	10.3%	1.16 [0.98, 1.38]
**Subtotal (95% CI)**		**140**		**138**	**45.0%**	**1.20 [1.08, 1.32]**
Total events	131		108			
Heterogeneity: Chi² = 2.13, df = 3 (P = 0.54); I² = 0%						
Test for overall effect: Z = 3.59 (P = 0.0003)						
**Total (95% CI)**		**347**		**345**	**100.0%**	**1.30 [1.20, 1.40]**
Total events	315		241			

Heterogeneity: Chi² = 9.52, df = 10 (P = 0.48); I² = 0%
Test for overall effect: Z = 6.72 (P < 0.00001)
Test for subgroup differences: Chi² = 3.69, df = 1 (P = 0.05), I² = 72.9%

Risk Ratio M-H, Fixed, 95% CI

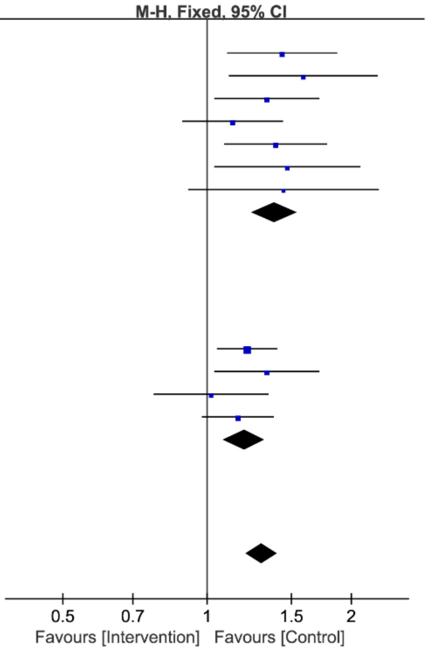

S2.3 30 days

Angina Efficacy<br>Study or Subgroup	Intervention<br>Events	 Total	Control<br>Events	 Total	 Weight	Risk Ratio<br>M-H, Fixed, 95% CI
**≤30 days**						
Chen, J. (2017)	57	60	47	60	19.4%	1.21 [1.05, 1.40]
Fan, G. H. (2012)	33	36	23	36	9.5%	1.43 [1.10, 1.87]
Wang, J. (2016)	26	30	23	30	9.5%	1.13 [0.89, 1.44]
Zhu, G. Y. (2014)	13	15	9	15	3.7%	1.44 [0.91, 2.28]
**Subtotal (95% CI)**		**141**		**141**	**42.2%**	**1.26 [1.13, 1.42]**
Total events	129		102			
Heterogeneity: $Chi^2 = 2.34$, df = 3 (P = 0.50); $I^2 = 0\%$						
Test for overall effect: Z = 4.06 (P < 0.0001)						
**>30 days**						
Han, Y. (2022)	27	32	17	32	7.0%	1.59 [1.11, 2.27]
Li, J. (2014)	28	30	21	30	8.7%	1.33 [1.04, 1.72]
Qiao, J. F. (2021)	28	30	21	30	8.7%	1.33 [1.04, 1.72]
Yuan, H. W. (2018)	32	34	23	34	9.5%	1.39 [1.09, 1.78]
Zhang, P. (2016)	25	30	17	30	7.0%	1.47 [1.03, 2.09]
Zhao, M. J. (2011)	17	20	15	18	6.5%	1.02 [0.77, 1.35]
Zhong, Q. Y. (2019)	29	30	25	30	10.3%	1.16 [0.98, 1.38]
**Subtotal (95% CI)**		**206**		**204**	**57.8%**	**1.32 [1.19, 1.47]**
Total events	186		139			
Heterogeneity: $Chi^2 = 7.15$, df = 6 (P = 0.31); $I^2 = 16\%$						
Test for overall effect: Z = 5.36 (P < 0.00001)						
**Total (95% CI)**		**347**		**345**	**100.0%**	**1.30 [1.20, 1.40]**
Total events	315		241			
Heterogeneity: $Chi^2 = 9.52$, df = 10 (P = 0.48); $I^2 = 0\%$						
Test for overall effect: Z = 6.72 (P < 0.00001)						
Test for subgroup differences: $Chi^2 = 0.35$, df = 1 (P = 0.56), $I^2 = 0\%$						

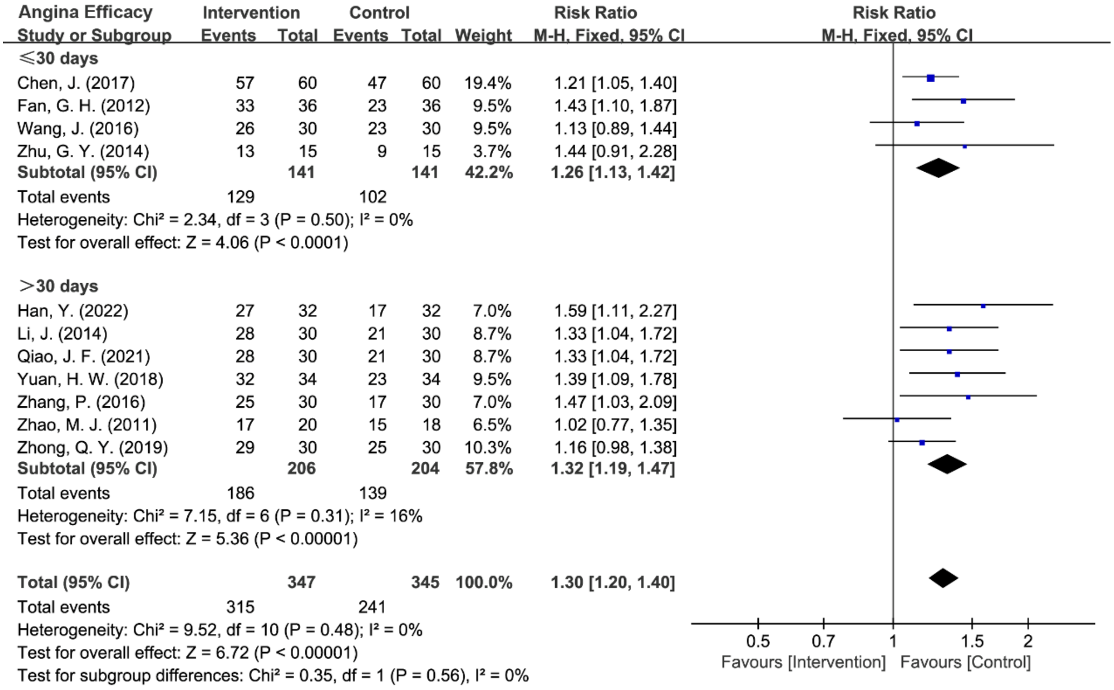

S2.4 45 days

Angina Efficacy<br>Study or Subgroup	Intervention<br>Events	 Total	Control<br>Events	 Total	 Weight	Risk Ratio<br>M-H, Fixed, 95% CI
**≤45 days**						
Chen, J. (2017)	57	60	47	60	19.4%	1.21 [1.05, 1.40]
Fan, G. H. (2012)	33	36	23	36	9.5%	1.43 [1.10, 1.87]
Wang, J. (2016)	26	30	23	30	9.5%	1.13 [0.89, 1.44]
Zhao, M. J. (2011)	17	20	15	18	6.5%	1.02 [0.77, 1.35]
Zhong, Q. Y. (2019)	29	30	25	30	10.3%	1.16 [0.98, 1.38]
Zhu, G. Y. (2014)	13	15	9	15	3.7%	1.44 [0.91, 2.28]
**Subtotal (95% CI)**		**191**		**189**	**59.1%**	**1.22 [1.11, 1.34]**
Total events	175		142			
Heterogeneity: $Chi^2 = 4.27$, df = 5 (P = 0.51); $I^2 = 0\%$						
Test for overall effect: Z = 4.22 (P < 0.0001)						
**>45 days**						
Han, Y. (2022)	27	32	17	32	7.0%	1.59 [1.11, 2.27]
Li, J. (2014)	28	30	21	30	8.7%	1.33 [1.04, 1.72]
Qiao, J. F. (2021)	28	30	21	30	8.7%	1.33 [1.04, 1.72]
Yuan, H. W. (2018)	32	34	23	34	9.5%	1.39 [1.09, 1.78]
Zhang, P. (2016)	25	30	17	30	7.0%	1.47 [1.03, 2.09]
**Subtotal (95% CI)**		**156**		**156**	**40.9%**	**1.41 [1.24, 1.61]**
Total events	140		99			
Heterogeneity: $Chi^2 = 0.88$, df = 4 (P = 0.93); $I^2 = 0\%$						
Test for overall effect: Z = 5.25 (P < 0.00001)						
**Total (95% CI)**		**347**		**345**	**100.0%**	**1.30 [1.20, 1.40]**
Total events	315		241			
Heterogeneity: $Chi^2 = 9.52$, df = 10 (P = 0.48); $I^2 = 0\%$						
Test for overall effect: Z = 6.72 (P < 0.00001)						
Test for subgroup differences: $Chi^2 = 3.35$, df = 1 (P = 0.07), $I^2 = 70.2\%$						

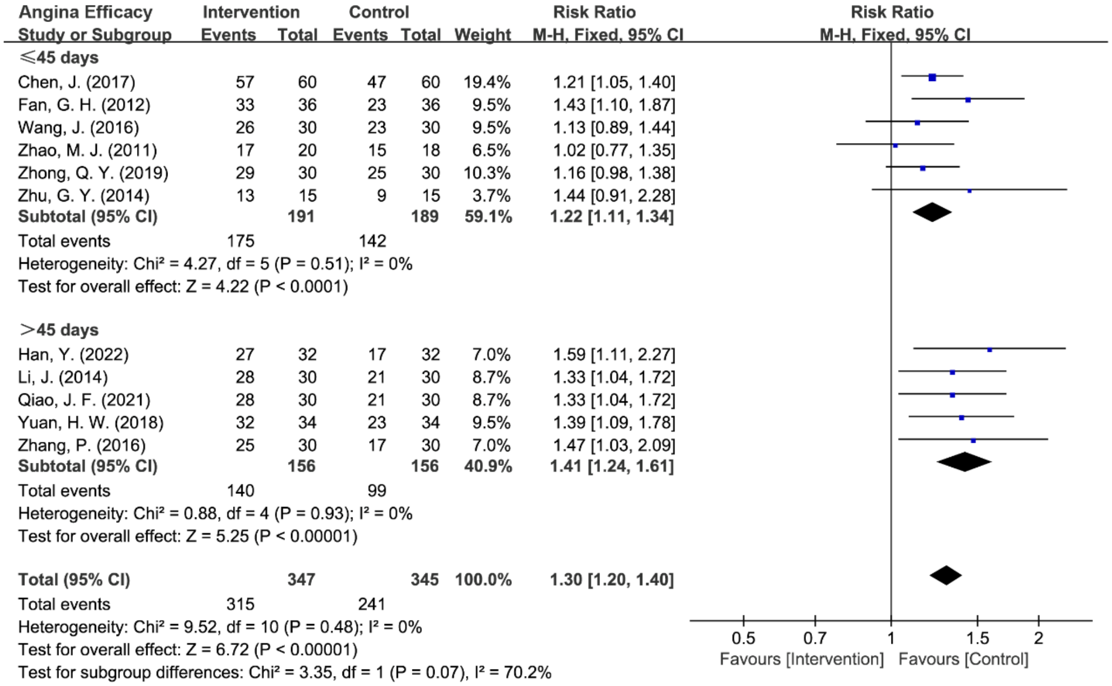

S2.5 60 days

Angina Efficacy<br>Study or Subgroup	Intervention<br>Events	Total	Control<br>Events	Total	Weight	Risk Ratio<br>M-H, Fixed, 95% CI
**≤60 days**						
Chen, J. (2017)	57	60	47	60	19.4%	1.21 [1.05, 1.40]
Fan, G. H. (2012)	33	36	23	36	9.5%	1.43 [1.10, 1.87]
Han, Y. (2022)	27	32	17	32	7.0%	1.59 [1.11, 2.27]
Qiao, J. F. (2021)	28	30	21	30	8.7%	1.33 [1.04, 1.72]
Wang, J. (2016)	26	30	23	30	9.5%	1.13 [0.89, 1.44]
Zhang, P. (2016)	25	30	17	30	7.0%	1.47 [1.03, 2.09]
Zhao, M. J. (2011)	17	20	15	18	6.5%	1.02 [0.77, 1.35]
Zhong, Q. Y. (2019)	29	30	25	30	10.3%	1.16 [0.98, 1.38]
Zhu, G. Y. (2014)	13	15	9	15	3.7%	1.44 [0.91, 2.28]
**Subtotal (95% CI)**		**283**		**281**	**81.8%**	**1.28 [1.18, 1.40]**
Total events	255		197			
Heterogeneity: Chi² = 8.60, df = 8 (P = 0.38); I² = 7%						
Test for overall effect: Z = 5.81 (P < 0.00001)						
**>60 days**						
Li, J. (2014)	28	30	21	30	8.7%	1.33 [1.04, 1.72]
Yuan, H. W. (2018)	32	34	23	34	9.5%	1.39 [1.09, 1.78]
**Subtotal (95% CI)**		**64**		**64**	**18.2%**	**1.36 [1.14, 1.63]**
Total events	60		44			
Heterogeneity: Chi² = 0.06, df = 1 (P = 0.81); I² = 0%						
Test for overall effect: Z = 3.44 (P = 0.0006)						
**Total (95% CI)**		**347**		**345**	**100.0%**	**1.30 [1.20, 1.40]**
Total events	315		241			
Heterogeneity: Chi² = 9.52, df = 10 (P = 0.48); I² = 0%						
Test for overall effect: Z = 6.72 (P < 0.00001)						
Test for subgroup differences: Chi² = 0.36, df = 1 (P = 0.55), I² = 0%						

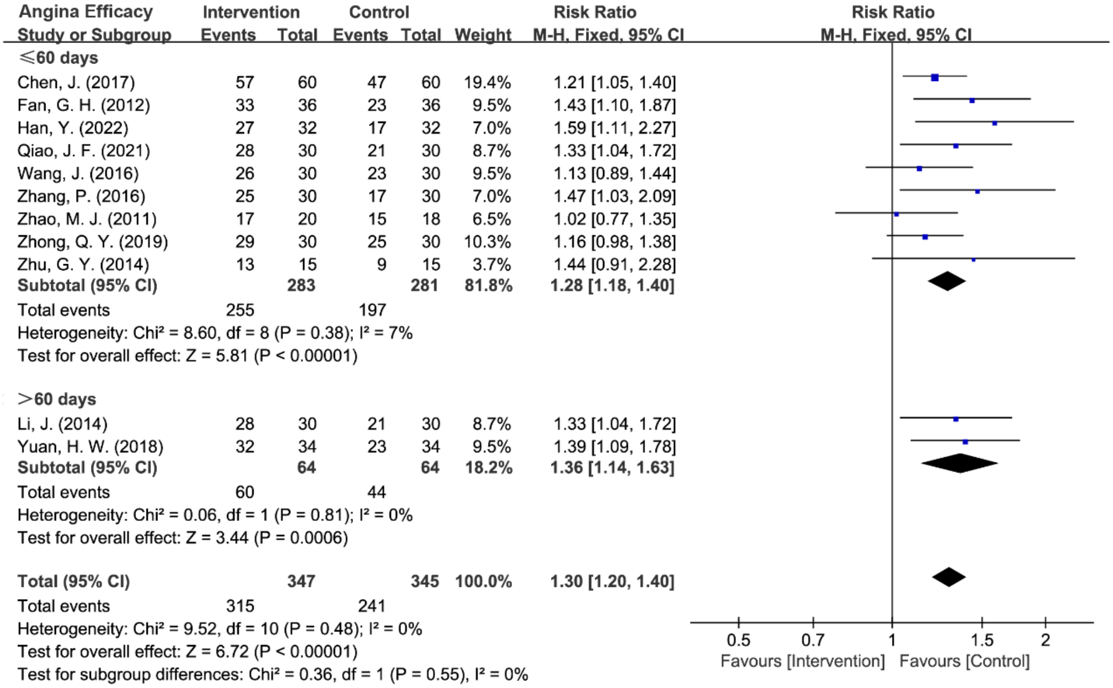

S2.6 30, 30-60, 60 days (Fixed-Effects Model)

Angina Efficacy<br>Study or Subgroup	Intervention<br>Events	Total	Control<br>Events	Total	Weight	Risk Ratio<br>M-H, Fixed, 95% CI
**≤30 days**						
Chen, J. (2017)	57	60	47	60	19.4%	1.21 [1.05, 1.40]
Fan, G. H. (2012)	33	36	23	36	9.5%	1.43 [1.10, 1.87]
Wang, J. (2016)	26	30	23	30	9.5%	1.13 [0.89, 1.44]
Zhu, G. Y. (2014)	13	15	9	15	3.7%	1.44 [0.91, 2.28]
**Subtotal (95% CI)**		**141**		**141**	**42.2%**	**1.26 [1.13, 1.42]**
Total events	129		102			
Heterogeneity: Chi² = 2.34, df = 3 (P = 0.50); I² = 0%						
Test for overall effect: Z = 4.06 (P < 0.0001)						
**30< days ≤60**						
Han, Y. (2022)	27	32	17	32	7.0%	1.59 [1.11, 2.27]
Qiao, J. F. (2021)	28	30	21	30	8.7%	1.33 [1.04, 1.72]
Zhang, P. (2016)	25	30	17	30	7.0%	1.47 [1.03, 2.09]
Zhao, M. J. (2011)	17	20	15	18	6.5%	1.02 [0.77, 1.35]
Zhong, Q. Y. (2019)	29	30	25	30	10.3%	1.16 [0.98, 1.38]
**Subtotal (95% CI)**		**142**		**140**	**39.6%**	**1.31 [1.15, 1.48]**
Total events	126		95			
Heterogeneity: Chi² = 6.48, df = 4 (P = 0.17); I² = 38%						
Test for overall effect: Z = 4.15 (P < 0.0001)						
**>60 days**						
Li, J. (2014)	28	30	21	30	8.7%	1.33 [1.04, 1.72]
Yuan, H. W. (2018)	32	34	23	34	9.5%	1.39 [1.09, 1.78]
**Subtotal (95% CI)**		**64**		**64**	**18.2%**	**1.36 [1.14, 1.63]**
Total events	60		44			
Heterogeneity: Chi² = 0.06, df = 1 (P = 0.81); I² = 0%						
Test for overall effect: Z = 3.44 (P = 0.0006)						
**Total (95% CI)**		**347**		**345**	**100.0%**	**1.30 [1.20, 1.40]**
Total events	315		241			
Heterogeneity: Chi² = 9.52, df = 10 (P = 0.48); I² = 0%						
Test for overall effect: Z = 6.72 (P < 0.00001)						
Test for subgroup differences: Chi² = 0.51, df = 2 (P = 0.78), I² = 0%						

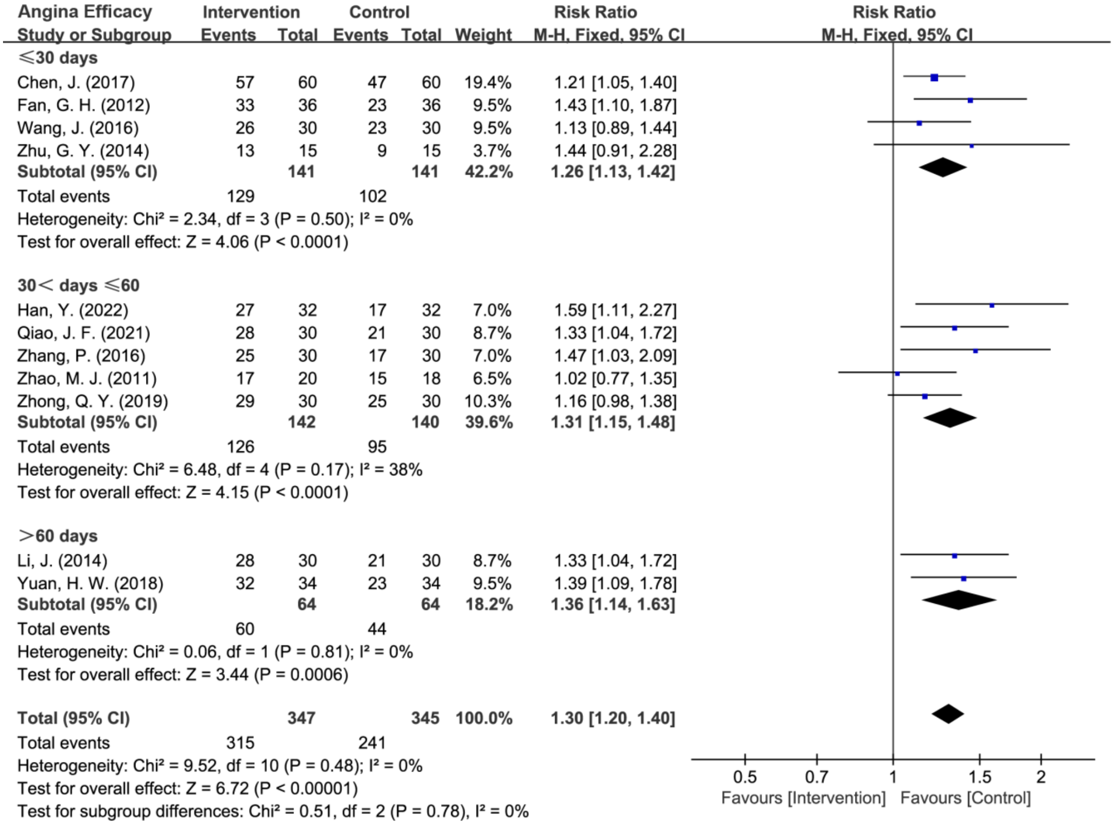

S2.7 30, 30-60, 60 days (Random-Effects Model)

Angina Efficacy<br>Study or Subgroup	Intervention<br>Events	 Total	Control<br>Events	 Total	 Weight	Risk Ratio<br>M-H, Random, 95% CI
**≤30 days**						
Chen, J. (2017)	57	60	47	60	24.5%	1.21 [1.05, 1.40]
Fan, G. H. (2012)	33	36	23	36	7.4%	1.43 [1.10, 1.87]
Wang, J. (2016)	26	30	23	30	8.8%	1.13 [0.89, 1.44]
Zhu, G. Y. (2014)	13	15	9	15	2.5%	1.44 [0.91, 2.28]
**Subtotal (95% CI)**		**141**		**141**	**43.2%**	**1.24 [1.11, 1.39]**
Total events	129		102			
Heterogeneity: Tau² = 0.00; Chi² = 2.34, df = 3 (P = 0.50); I² = 0%						
Test for overall effect: Z = 3.89 (P < 0.0001)						
**30< days ≤60**						
Han, Y. (2022)	27	32	17	32	4.0%	1.59 [1.11, 2.27]
Qiao, J. F. (2021)	28	30	21	30	8.1%	1.33 [1.04, 1.72]
Zhang, P. (2016)	25	30	17	30	4.2%	1.47 [1.03, 2.09]
Zhao, M. J. (2011)	17	20	15	18	6.8%	1.02 [0.77, 1.35]
Zhong, Q. Y. (2019)	29	30	25	30	17.2%	1.16 [0.98, 1.38]
**Subtotal (95% CI)**		**142**		**140**	**40.3%**	**1.26 [1.08, 1.46]**
Total events	126		95			
Heterogeneity: Tau² = 0.01; Chi² = 6.48, df = 4 (P = 0.17); I² = 38%						
Test for overall effect: Z = 2.94 (P = 0.003)						
**>60 days**						
Li, J. (2014)	28	30	21	30	8.1%	1.33 [1.04, 1.72]
Yuan, H. W. (2018)	32	34	23	34	8.5%	1.39 [1.09, 1.78]
**Subtotal (95% CI)**		**64**		**64**	**16.5%**	**1.36 [1.14, 1.63]**
Total events	60		44			
Heterogeneity: Tau² = 0.00; Chi² = 0.06, df = 1 (P = 0.81); I² = 0%						
Test for overall effect: Z = 3.43 (P = 0.0006)						
**Total (95% CI)**		**347**		**345**	**100.0%**	**1.26 [1.17, 1.35]**
Total events	315		241			
Heterogeneity: Tau² = 0.00; Chi² = 9.52, df = 10 (P = 0.48); I² = 0%						
Test for overall effect: Z = 6.27 (P < 0.00001)						
Test for subgroup differences: Chi² = 0.78, df = 2 (P = 0.68), I² = 0%						

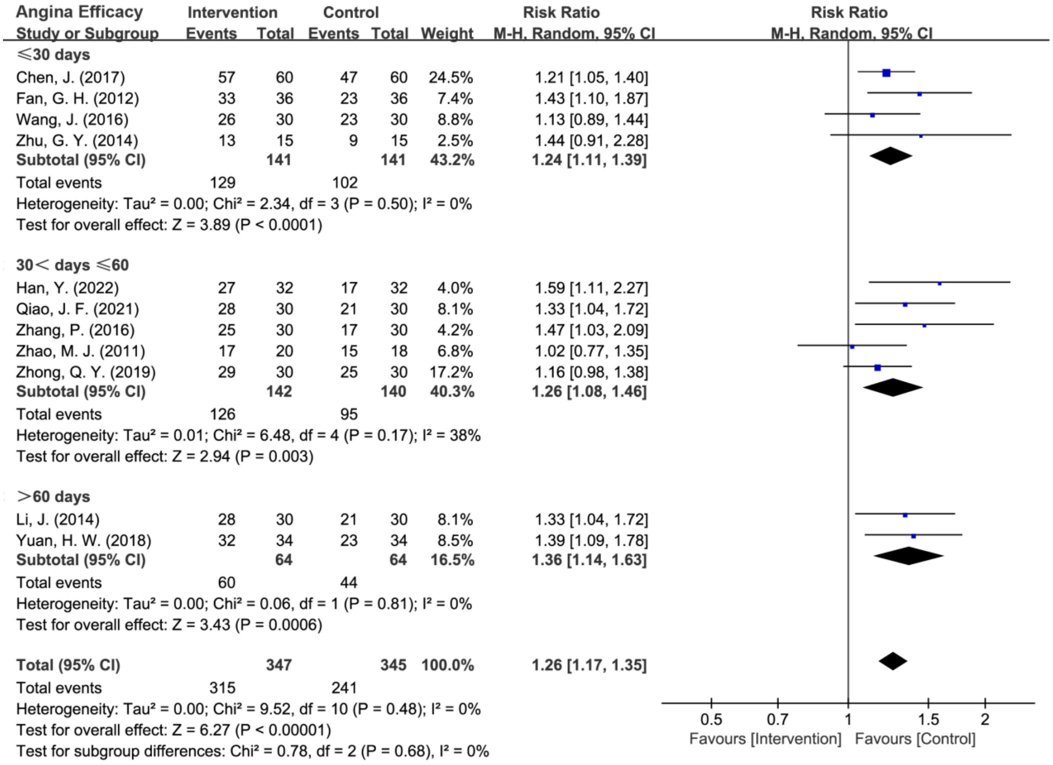

S2.8 30, 30-45, 45-60, 60 days (Random-Effects Model)

Angina Efficacy<br>Study or Subgroup	Intervention<br>Events	 Total	Control<br>Events	 Total	 Weight	Risk Ratio<br>M-H, Random, 95% CI
**≤30 days**						
Chen, J. (2017)	57	60	47	60	24.5%	1.21 [1.05, 1.40]
Fan, G. H. (2012)	33	36	23	36	7.4%	1.43 [1.10, 1.87]
Wang, J. (2016)	26	30	23	30	8.8%	1.13 [0.89, 1.44]
Zhu, G. Y. (2014)	13	15	9	15	2.5%	1.44 [0.91, 2.28]
**Subtotal (95% CI)**		**141**		**141**	**43.2%**	**1.24 [1.11, 1.39]**
Total events	129		102			
Heterogeneity: Tau² = 0.00; Chi² = 2.34, df = 3 (P = 0.50); I² = 0%						
Test for overall effect: Z = 3.89 (P < 0.0001)						
**30< days ≤45**						
Zhao, M. J. (2011)	17	20	15	18	6.8%	1.02 [0.77, 1.35]
Zhong, Q. Y. (2019)	29	30	25	30	17.2%	1.16 [0.98, 1.38]
**Subtotal (95% CI)**		**50**		**48**	**24.0%**	**1.12 [0.97, 1.30]**
Total events	46		40			
Heterogeneity: Tau² = 0.00; Chi² = 0.62, df = 1 (P = 0.43); I² = 0%						
Test for overall effect: Z = 1.50 (P = 0.13)						
**45< days ≤60**						
Han, Y. (2022)	27	32	17	32	4.0%	1.59 [1.11, 2.27]
Qiao, J. F. (2021)	28	30	21	30	8.1%	1.33 [1.04, 1.72]
Zhang, P. (2016)	25	30	17	30	4.2%	1.47 [1.03, 2.09]
**Subtotal (95% CI)**		**92**		**92**	**16.3%**	**1.43 [1.19, 1.71]**
Total events	80		55			
Heterogeneity: Tau² = 0.00; Chi² = 0.69, df = 2 (P = 0.71); I² = 0%						
Test for overall effect: Z = 3.92 (P < 0.0001)						
**>60 days**						
Li, J. (2014)	28	30	21	30	8.1%	1.33 [1.04, 1.72]
Yuan, H. W. (2018)	32	34	23	34	8.5%	1.39 [1.09, 1.78]
**Subtotal (95% CI)**		**64**		**64**	**16.5%**	**1.36 [1.14, 1.63]**
Total events	60		44			
Heterogeneity: Tau² = 0.00; Chi² = 0.06, df = 1 (P = 0.81); I² = 0%						
Test for overall effect: Z = 3.43 (P = 0.0006)						
**Total (95% CI)**		**347**		**345**	**100.0%**	**1.26 [1.17, 1.35]**
Total events	315		241			
Heterogeneity: Tau² = 0.00; Chi² = 9.52, df = 10 (P = 0.48); I² = 0%						
Test for overall effect: Z = 6.27 (P < 0.00001)						
Test for subgroup differences: Chi² = 5.23, df = 3 (P = 0.16), I² = 42.6%						

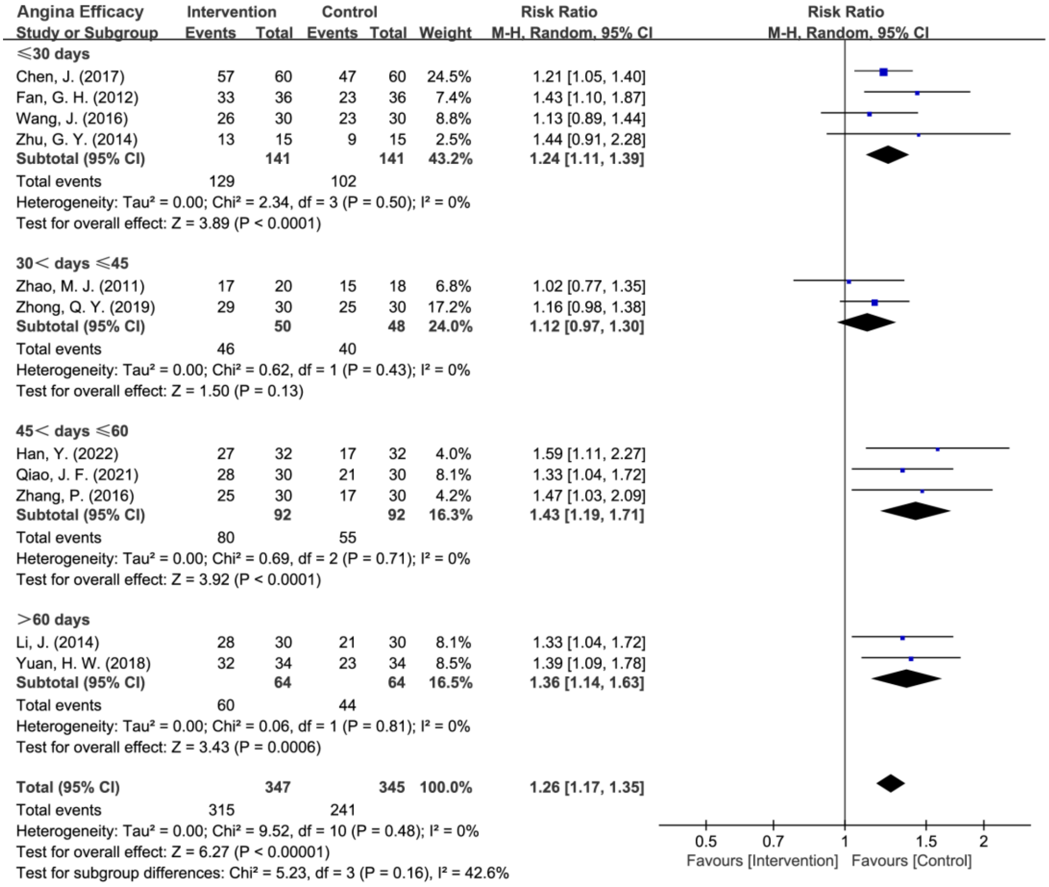

S2.9 30, 30-45, 45-60, 60 days (Fixed-Effects Model)

Angina Efficacy	Intervention		Control			Risk Ratio
Study or Subgroup	Events	Total	Events	Total	Weight	M-H, Fixed, 95% CI
**≤30 days**						
Chen, J. (2017)	57	60	47	60	19.4%	1.21 [1.05, 1.40]
Fan, G. H. (2012)	33	36	23	36	9.5%	1.43 [1.10, 1.87]
Wang, J. (2016)	26	30	23	30	9.5%	1.13 [0.89, 1.44]
Zhu, G. Y. (2014)	13	15	9	15	3.7%	1.44 [0.91, 2.28]
**Subtotal (95% CI)**		**141**		**141**	**42.2%**	**1.26 [1.13, 1.42]**
Total events	129		102			
Heterogeneity: Chi² = 2.34, df = 3 (P = 0.50); I² = 0%						
Test for overall effect: Z = 4.06 (P < 0.0001)						
**30< days ≤45**						
Zhao, M. J. (2011)	17	20	15	18	6.5%	1.02 [0.77, 1.35]
Zhong, Q. Y. (2019)	29	30	25	30	10.3%	1.16 [0.98, 1.38]
**Subtotal (95% CI)**		**50**		**48**	**16.9%**	**1.11 [0.95, 1.28]**
Total events	46		40			
Heterogeneity: Chi² = 0.62, df = 1 (P = 0.43); I² = 0%						
Test for overall effect: Z = 1.32 (P = 0.19)						
**45< days ≤60**						
Han, Y. (2022)	27	32	17	32	7.0%	1.59 [1.11, 2.27]
Qiao, J. F. (2021)	28	30	21	30	8.7%	1.33 [1.04, 1.72]
Zhang, P. (2016)	25	30	17	30	7.0%	1.47 [1.03, 2.09]
**Subtotal (95% CI)**		**92**		**92**	**22.7%**	**1.45 [1.21, 1.75]**
Total events	80		55			
Heterogeneity: Chi² = 0.69, df = 2 (P = 0.71); I² = 0%						
Test for overall effect: Z = 3.99 (P < 0.0001)						
**>60 days**						
Li, J. (2014)	28	30	21	30	8.7%	1.33 [1.04, 1.72]
Yuan, H. W. (2018)	32	34	23	34	9.5%	1.39 [1.09, 1.78]
**Subtotal (95% CI)**		**64**		**64**	**18.2%**	**1.36 [1.14, 1.63]**
Total events	60		44			
Heterogeneity: Chi² = 0.06, df = 1 (P = 0.81); I² = 0%						
Test for overall effect: Z = 3.44 (P = 0.0006)						
**Total (95% CI)**		**347**		**345**	**100.0%**	**1.30 [1.20, 1.40]**
Total events	315		241			
Heterogeneity: Chi² = 9.52, df = 10 (P = 0.48); I² = 0%						
Test for overall effect: Z = 6.72 (P < 0.00001)						
Test for subgroup differences: Chi² = 6.00, df = 3 (P = 0.11), I² = 50.0%						

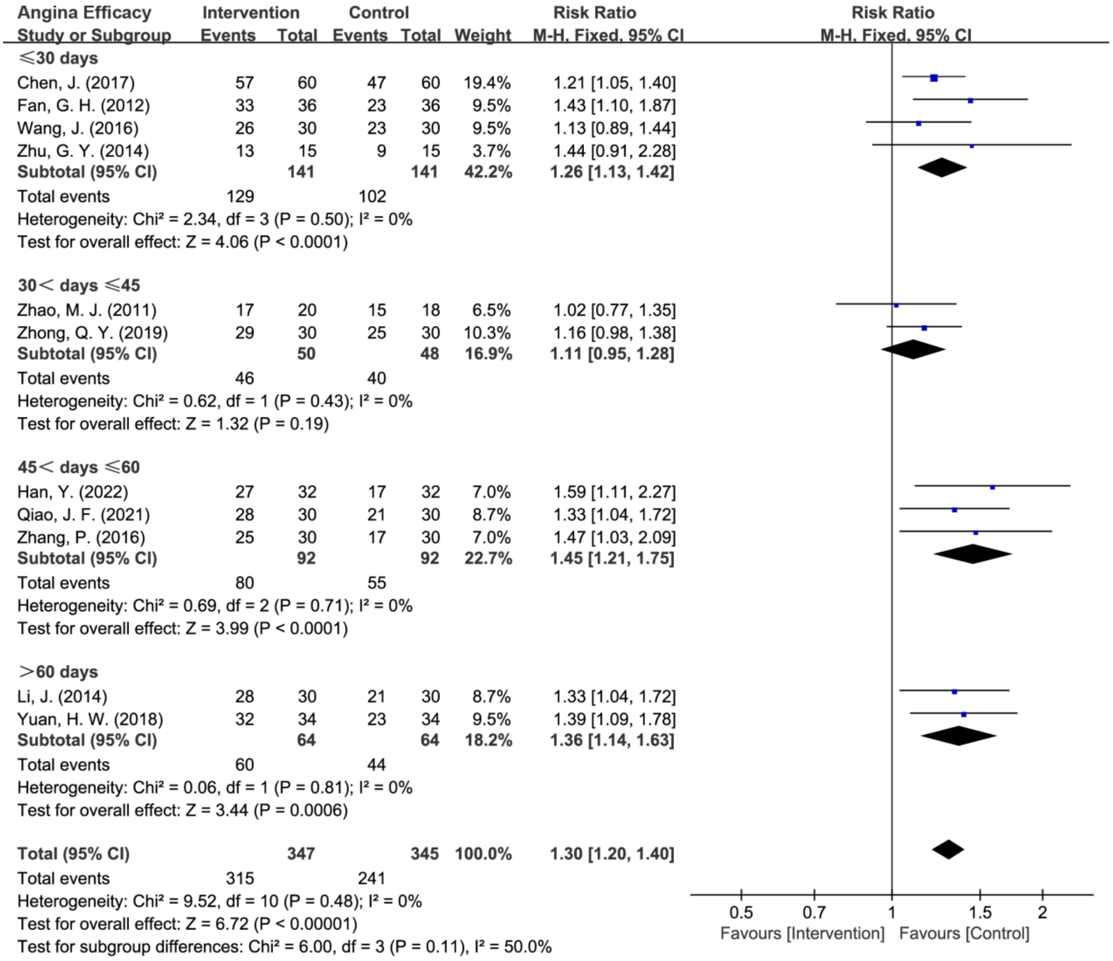

S3. Angina Attack Frequency Efficacy Analysis

S3.1 All 4 RCTs after conversion of units

	Intervention			Control				Mean Difference
Study or Subgroup	Mean	SD	Total	Mean	SD	Total	Weight	IV, Random, 95% CI
Cao, M. (2024)	-3.34	1.26	35	-2.32	1.26	35	25.4%	-1.02 [-1.61, -0.43]
Wang, J. G. (2016)	-0.08	0.03	40	-0.06	0.01	40	31.3%	-0.02 [-0.03, -0.01]
Yin, X. S. (2021)	-2.57	1.16	35	-1.61	1.06	35	26.5%	-0.96 [-1.48, -0.44]
Zheng, Y. H. (2012)	-1.6	2.62	40	-0.9	2.52	40	16.9%	-0.70 [-1.83, 0.43]
**Total (95% CI)**			**150**			**150**	**100.0%**	**-0.64 [-1.32, 0.05]**

Heterogeneity: Tau² = 0.39; Chi² = 24.93, df = 3 (P < 0.0001); I² = 88%
Test for overall effect: Z = 1.83 (P = 0.07)

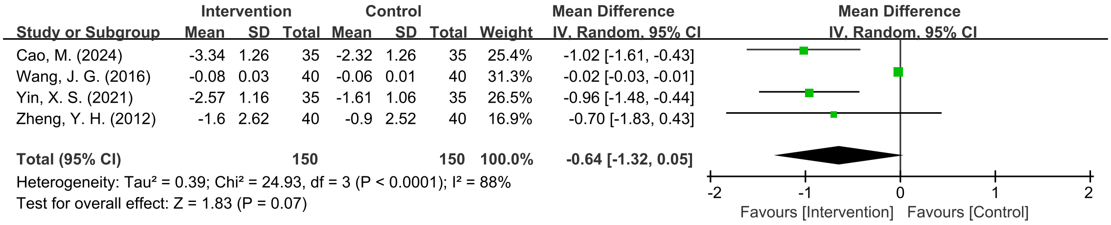

S3.2 Sensitivity Analysis of Heterogeneous Sources

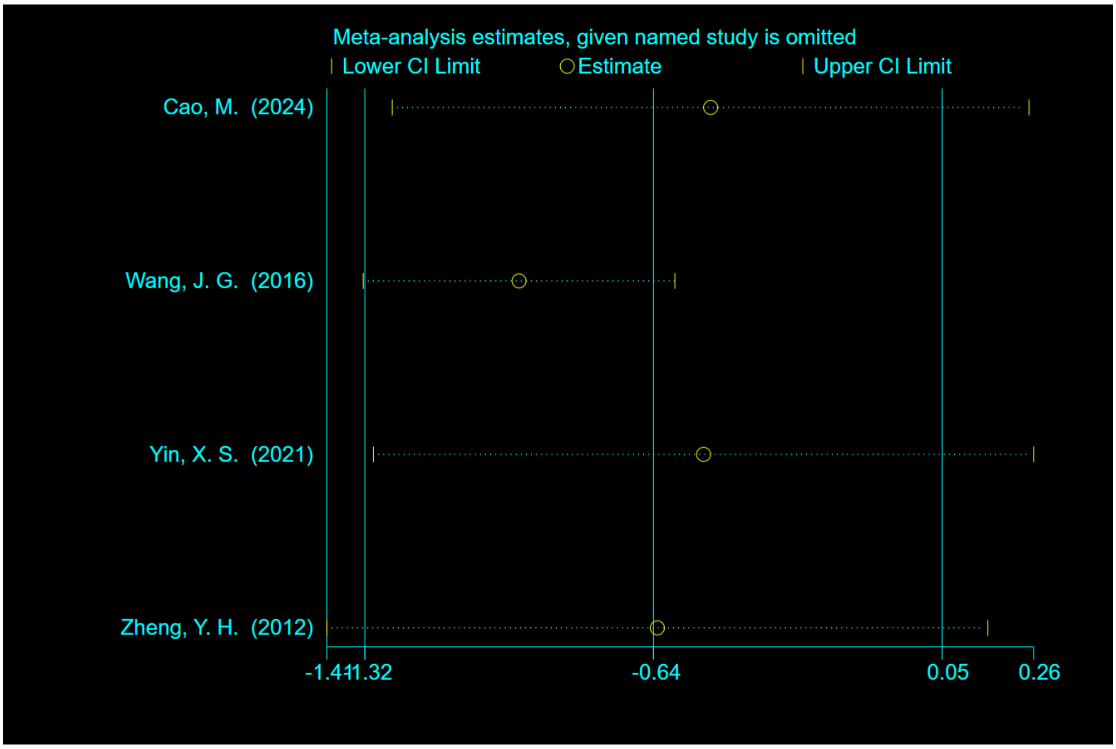

Study omitted	Estimate	[95% Conf.	Interval]
Cao, M. (2024)	-.50197375	-1.2558076	.25186017
Wang, J. G. (2016)	-.95554918	-1.3244717	-.58662665
Yin, X. S. (2021)	-.51869774	-1.3002707	.26287517
Zheng, Y. H. (2012)	-.62814158	-1.4104522	.15416907
Combined	-.63731184	-1.3206452	.04602149

S3.3 Sensitivity analysis for Deleting Zheng, Y. H. (2012)' High-risk Articles

Angina Frequency	Intervention			Control				Mean Difference
Study or Subgroup	Mean	SD	Total	Mean	SD	Total	Weight	IV, Random, 95% CI
Cao, M. (2024)	-3.34	1.26	35	-2.32	1.26	35	30.7%	-1.02 [-1.61, -0.43]
Wang, J. G. (2016)	-0.08	0.03	40	-0.06	0.01	40	37.3%	-0.02 [-0.03, -0.01]
Yin, X. S. (2021)	-2.57	1.16	35	-1.61	1.06	35	32.0%	-0.96 [-1.48, -0.44]
**Total (95% CI)**			**110**			**110**	**100.0%**	**-0.63 [-1.41, 0.15]**

Heterogeneity: Tau² = 0.43; Chi² = 23.53, df = 2 (P < 0.00001); I² = 92%
Test for overall effect: Z = 1.57 (P = 0.12)

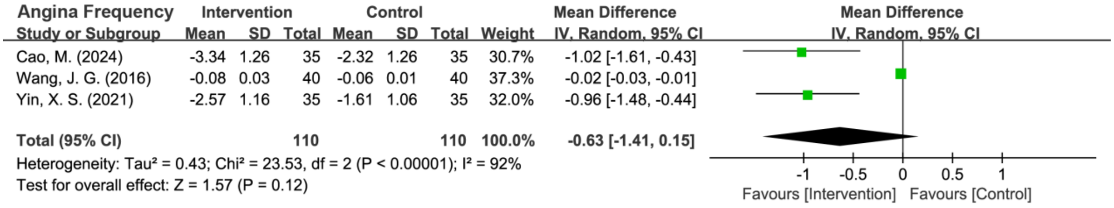

S3.4 Sensitivity analysis for Deleting Zheng, Y. H. (2012)' High-risk Articles and Heterogeneous Sources by Wang, J. G. (2016)

Angina Frequency	Intervention			Control				Mean Difference
Study or Subgroup	Mean	SD	Total	Mean	SD	Total	Weight	IV, Fixed, 95% CI
Cao, M. (2024)	-3.34	1.26	35	-2.32	1.26	35	43.7%	-1.02 [-1.61, -0.43]
Yin, X. S. (2021)	-2.57	1.16	35	-1.61	1.06	35	56.3%	-0.96 [-1.48, -0.44]
**Total (95% CI)**			**70**			**70**	**100.0%**	**-0.99 [-1.38, -0.60]**

Heterogeneity: Chi² = 0.02, df = 1 (P = 0.88); I² = 0%
Test for overall effect: Z = 4.95 (P < 0.00001)

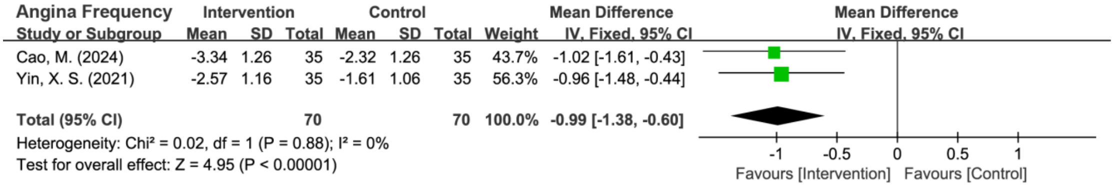

S4. ECG Efficacy Analysis

S4.1 Sensitivity Analysis of Heterogeneous Sources in the TCM-alone Group

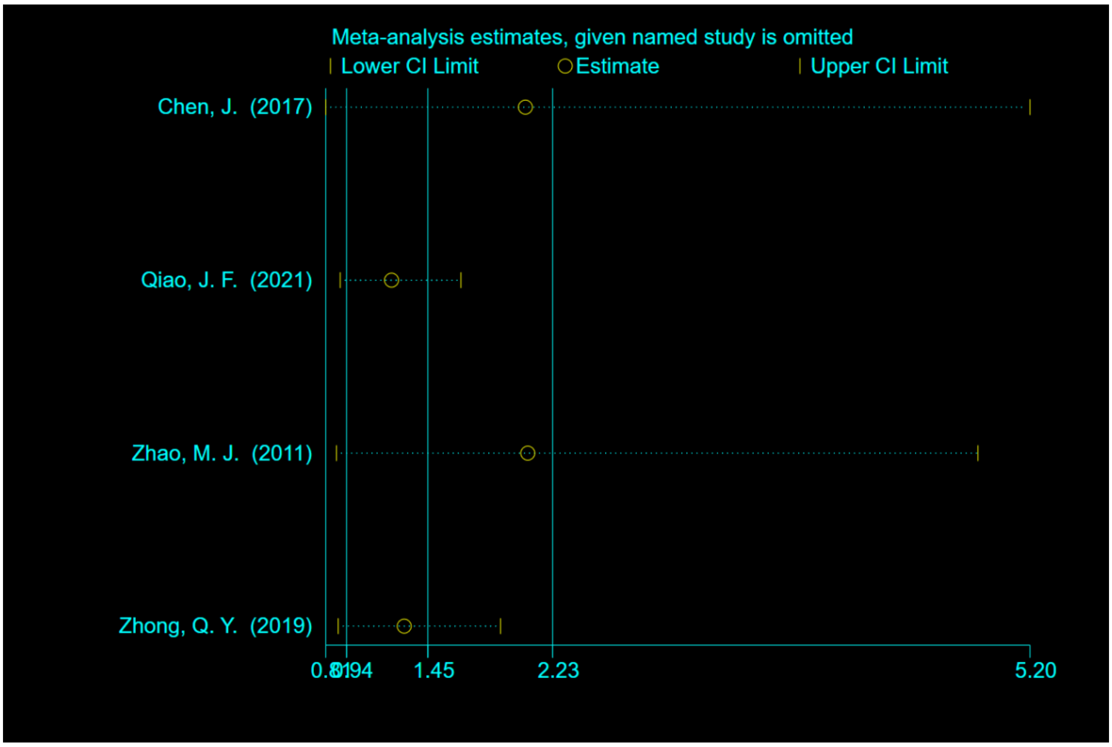

Study omitted	Estimate	[95% Conf.	Interval]
Chen, J. (2017)	2.0566592	.81372988	5.1980977
Qiao, J. F. (2021)	1.2232947	.90390247	1.6555438
Zhao, M. J. (2011)	2.0716915	.88059127	4.8738909
Zhong, Q. Y. (2019)	1.3021995	.8914479	1.9022126
Combined	1.4492641	.94355793	2.2260069

S4.2 Remaining 2 RCTs in the TCM-alone group (Exclude Qiao and Zhong)

Study or Subgroup	Intervention Events	Intervention Total	Control Events	Control Total	Weight	Risk Ratio M-H, Fixed, 95% CI
**TCM+BM**						
Cao, M. (2024)	34	35	28	35	11.1%	1.21 [1.02, 1.45]
Fan, G. H. (2012)	33	36	23	36	9.1%	1.43 [1.10, 1.87]
Li, J. (2014)	22	30	19	30	7.5%	1.16 [0.82, 1.64]
Wang, J. (2016)	22	30	18	30	7.1%	1.22 [0.85, 1.76]
Yin, X. S. (2021)	33	35	26	35	10.3%	1.27 [1.03, 1.57]
Yuan, H. W. (2018)	30	34	20	34	7.9%	1.50 [1.10, 2.04]
Zhang, J. (2023)	41	46	35	46	13.9%	1.17 [0.97, 1.42]
Zhang, P. (2016)	25	30	17	30	6.7%	1.47 [1.03, 2.09]
Zhu, G. Y. (2014)	12	15	11	15	4.4%	1.09 [0.73, 1.62]
**Subtotal (95% CI)**		**291**		**291**	**78.0%**	**1.28 [1.17, 1.40]**
Total events	252		197			
Heterogeneity: Chi² = 4.52, df = 8 (P = 0.81); I² = 0%						
Test for overall effect: Z = 5.34 (P < 0.00001)						
**TCM-alone**						
Chen, J. (2017)	53	60	44	60	17.4%	1.20 [1.01, 1.44]
Zhao, M. J. (2011)	13	20	11	18	4.6%	1.06 [0.65, 1.73]
**Subtotal (95% CI)**		**80**		**78**	**22.0%**	**1.18 [0.99, 1.40]**
Total events	66		55			
Heterogeneity: Chi² = 0.23, df = 1 (P = 0.63); I² = 0%						
Test for overall effect: Z = 1.83 (P = 0.07)						
**Total (95% CI)**		**371**		**369**	**100.0%**	**1.26 [1.16, 1.36]**
Total events	318		252			
Heterogeneity: Chi² = 5.07, df = 10 (P = 0.89); I² = 0%						
Test for overall effect: Z = 5.59 (P < 0.00001)						
Test for subgroup differences: Chi² = 0.73, df = 1 (P = 0.39), I² = 0%						

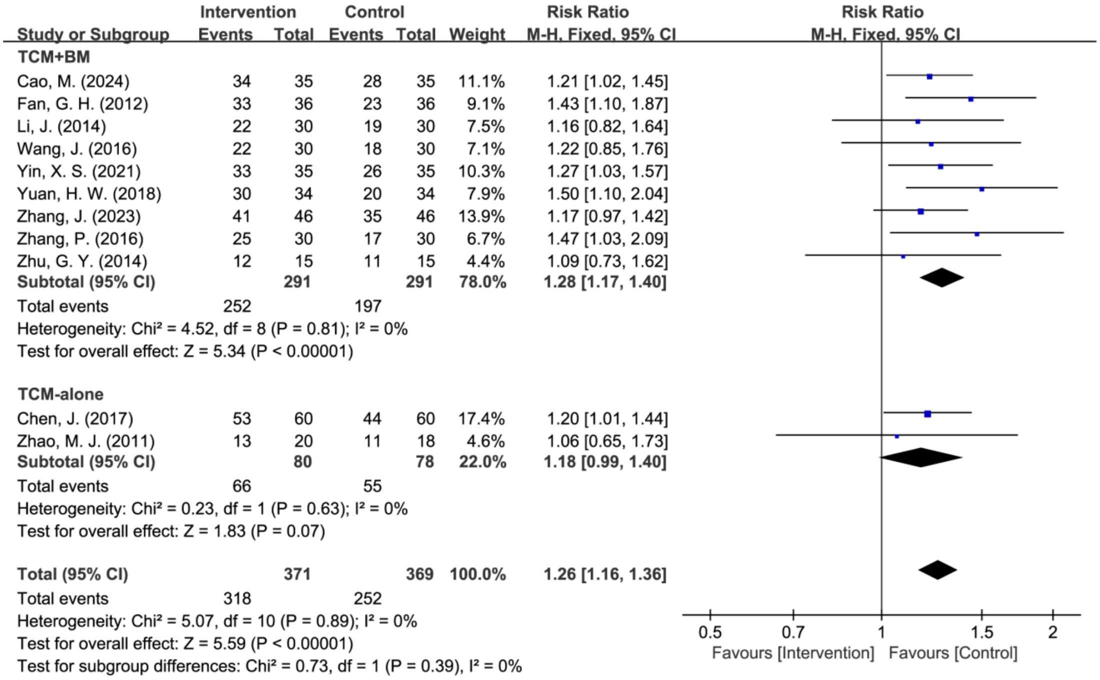

S4.3 Remaining 2 RCTs in the TCM-alone group (Exclude Chen and Zhao)

Study or Subgroup	Intervention Events	Intervention Total	Control Events	Control Total	Weight	Risk Ratio M-H, Fixed, 95% CI
**TCM+BM**						
Cao, M. (2024)	34	35	28	35	13.8%	1.21 [1.02, 1.45]
Fan, G. H. (2012)	33	36	23	36	11.3%	1.43 [1.10, 1.87]
Li, J. (2014)	22	30	19	30	9.4%	1.16 [0.82, 1.64]
Wang, J. (2016)	22	30	18	30	8.9%	1.22 [0.85, 1.76]
Yin, X. S. (2021)	33	35	26	35	12.8%	1.27 [1.03, 1.57]
Yuan, H. W. (2018)	30	34	20	34	9.9%	1.50 [1.10, 2.04]
Zhang, J. (2023)	41	46	35	46	17.2%	1.17 [0.97, 1.42]
Zhang, P. (2016)	25	30	17	30	8.4%	1.47 [1.03, 2.09]
Zhu, G. Y. (2014)	12	15	11	15	5.4%	1.09 [0.73, 1.62]
**Subtotal (95% CI)**		**291**		**291**	**97.0%**	**1.28 [1.17, 1.40]**
Total events	252		197			
Heterogeneity: Chi² = 4.52, df = 8 (P = 0.81); I² = 0%						
Test for overall effect: Z = 5.34 (P < 0.00001)						
**TCM-alone**						
Qiao, J. F. (2021)	13	30	4	30	2.0%	3.25 [1.20, 8.83]
Zhong, Q. Y. (2019)	8	30	2	30	1.0%	4.00 [0.92, 17.30]
**Subtotal (95% CI)**		**60**		**60**	**3.0%**	**3.50 [1.53, 8.01]**
Total events	21		6			
Heterogeneity: Chi² = 0.05, df = 1 (P = 0.82); I² = 0%						
Test for overall effect: Z = 2.97 (P = 0.003)						
**Total (95% CI)**		**351**		**351**	**100.0%**	**1.34 [1.22, 1.48]**
Total events	273		203			
Heterogeneity: Chi² = 11.74, df = 10 (P = 0.30); I² = 15%						
Test for overall effect: Z = 6.16 (P < 0.00001)						
Test for subgroup differences: Chi² = 5.61, df = 1 (P = 0.02), I² = 82.2%						

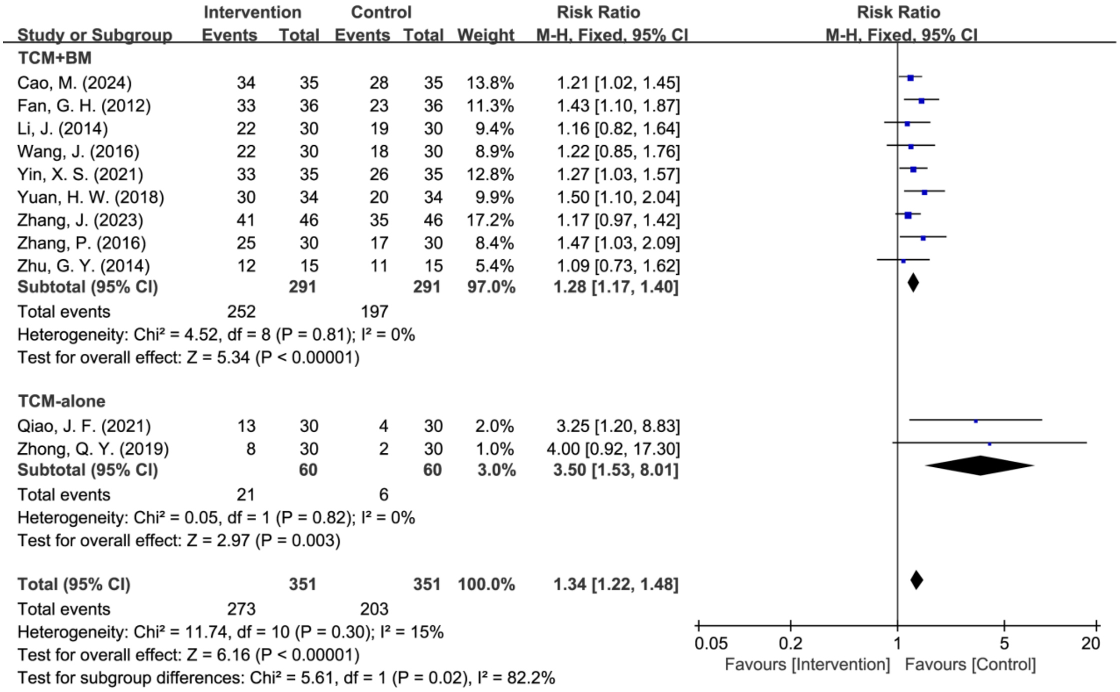

S5 ECG Efficacy Analysis based on Subgroups of Treatment Duration

S5.1 30 days

Study or Subgroup	Intervention Events	Intervention Total	Control Events	Control Total	Weight	Risk Ratio M-H, Fixed, 95% CI
**≤30 days**						
Chen, J. (2017)	53	60	44	60	17.0%	1.20 [1.01, 1.44]
Fan, G. H. (2012)	33	36	23	36	8.9%	1.43 [1.10, 1.87]
Wang, J. (2016)	22	30	18	30	7.0%	1.22 [0.85, 1.76]
Zhu, G. Y. (2014)	12	15	11	15	4.3%	1.09 [0.73, 1.62]
**Subtotal (95% CI)**		**141**		**141**	**37.1%**	**1.25 [1.10, 1.43]**
Total events	120		96			
Heterogeneity: Chi² = 1.68, df = 3 (P = 0.64); I² = 0%						
Test for overall effect: Z = 3.32 (P = 0.0009)						
**>30 days**						
Cao, M. (2024)	34	35	28	35	10.8%	1.21 [1.02, 1.45]
Li, J. (2014)	22	30	19	30	7.3%	1.16 [0.82, 1.64]
Qiao, J. F. (2021)	13	30	4	30	1.5%	3.25 [1.20, 8.83]
Yin, X. S. (2021)	33	35	26	35	10.1%	1.27 [1.03, 1.57]
Yuan, H. W. (2018)	30	34	20	34	7.7%	1.50 [1.10, 2.04]
Zhang, J. (2023)	41	46	35	46	13.5%	1.17 [0.97, 1.42]
Zhang, P. (2016)	25	30	17	30	6.6%	1.47 [1.03, 2.09]
Zhao, M. J. (2011)	13	20	11	18	4.5%	1.06 [0.65, 1.73]
Zhong, Q. Y. (2019)	8	30	2	30	0.8%	4.00 [0.92, 17.30]
**Subtotal (95% CI)**		**290**		**288**	**62.9%**	**1.34 [1.21, 1.49]**
Total events	219		162			
Heterogeneity: Chi² = 10.97, df = 8 (P = 0.20); I² = 27%						
Test for overall effect: Z = 5.44 (P < 0.00001)						
**Total (95% CI)**		**431**		**429**	**100.0%**	**1.31 [1.20, 1.42]**
Total events	339		258			
Heterogeneity: Chi² = 12.07, df = 12 (P = 0.44); I² = 1%						
Test for overall effect: Z = 6.36 (P < 0.00001)						
Test for subgroup differences: Chi² = 0.69, df = 1 (P = 0.41), I² = 0%						

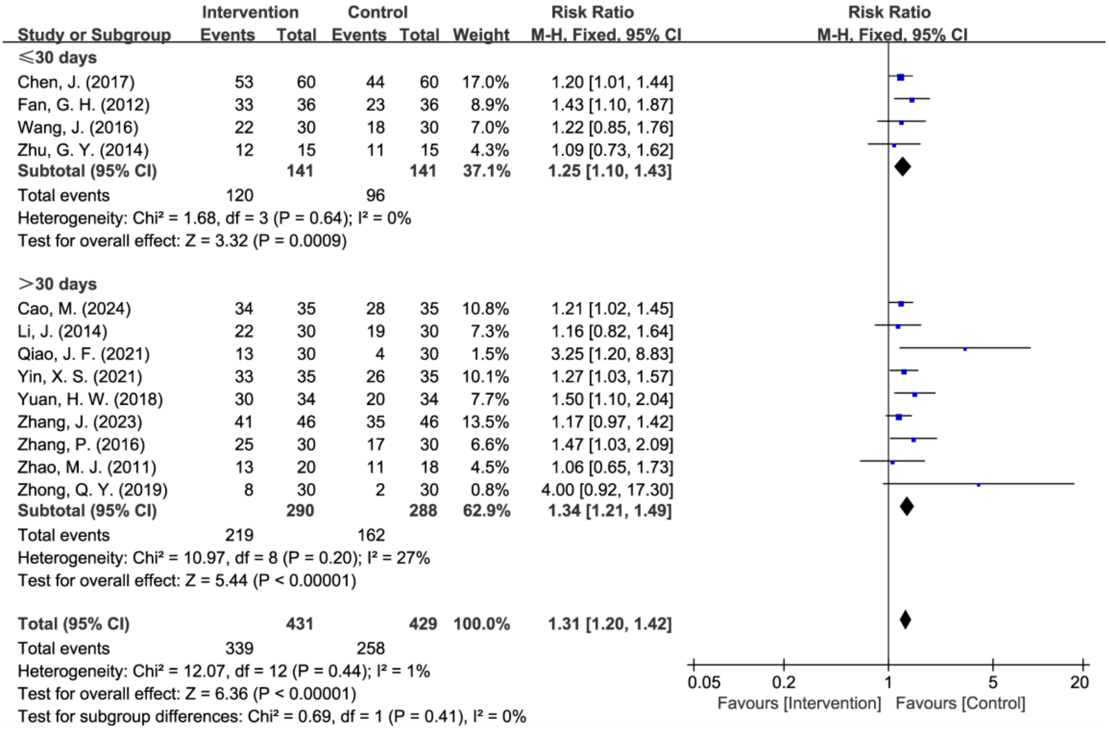

S5.2 45 days

Study or Subgroup	Intervention Events	Intervention Total	Control Events	Control Total	Weight	Risk Ratio M-H, Fixed, 95% CI
**≤45 days**						
Chen, J. (2017)	53	60	44	60	17.0%	1.20 [1.01, 1.44]
Fan, G. H. (2012)	33	36	23	36	8.9%	1.43 [1.10, 1.87]
Wang, J. (2016)	22	30	18	30	7.0%	1.22 [0.85, 1.76]
Zhao, M. J. (2011)	13	20	11	18	4.5%	1.06 [0.65, 1.73]
Zhong, Q. Y. (2019)	8	30	2	30	0.8%	4.00 [0.92, 17.30]
Zhu, G. Y. (2014)	12	15	11	15	4.3%	1.09 [0.73, 1.62]
**Subtotal (95% CI)**		**191**		**189**	**42.4%**	**1.28 [1.12, 1.46]**
Total events	141		109			
Heterogeneity: Chi² = 4.73, df = 5 (P = 0.45); I² = 0%						
Test for overall effect: Z = 3.67 (P = 0.0002)						
**>45 days**						
Cao, M. (2024)	34	35	28	35	10.8%	1.21 [1.02, 1.45]
Li, J. (2014)	22	30	19	30	7.3%	1.16 [0.82, 1.64]
Qiao, J. F. (2021)	13	30	4	30	1.5%	3.25 [1.20, 8.83]
Yin, X. S. (2021)	33	35	26	35	10.1%	1.27 [1.03, 1.57]
Yuan, H. W. (2018)	30	34	20	34	7.7%	1.50 [1.10, 2.04]
Zhang, J. (2023)	41	46	35	46	13.5%	1.17 [0.97, 1.42]
Zhang, P. (2016)	25	30	17	30	6.6%	1.47 [1.03, 2.09]
**Subtotal (95% CI)**		**240**		**240**	**57.6%**	**1.33 [1.20, 1.48]**
Total events	198		149			
Heterogeneity: Chi² = 7.47, df = 6 (P = 0.28); I² = 20%						
Test for overall effect: Z = 5.26 (P < 0.00001)						
**Total (95% CI)**		**431**		**429**	**100.0%**	**1.31 [1.20, 1.42]**
Total events	339		258			
Heterogeneity: Chi² = 12.07, df = 12 (P = 0.44); I² = 1%						
Test for overall effect: Z = 6.36 (P < 0.00001)						
Test for subgroup differences: Chi² = 0.18, df = 1 (P = 0.67), I² = 0%						

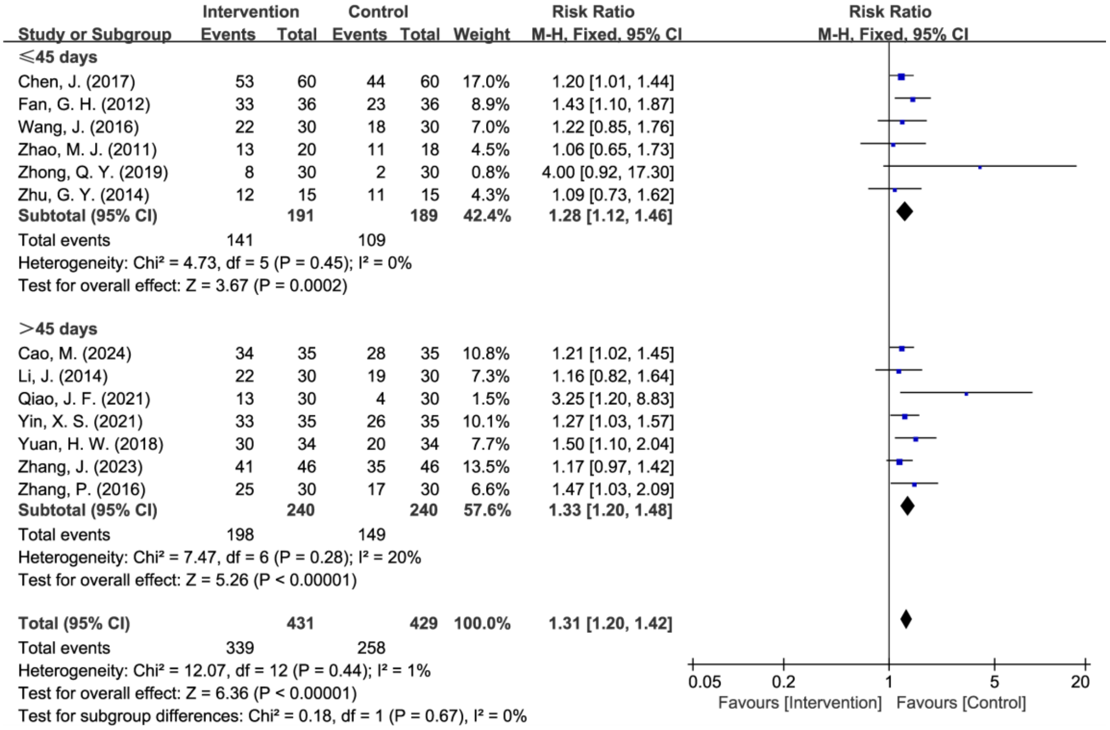

S5.3 60 days

Study or Subgroup	Intervention Events	Intervention Total	Control Events	Control Total	Weight	Risk Ratio M-H, Fixed, 95% CI
**≤ 60 days**						
Cao, M. (2024)	34	35	28	35	10.8%	1.21 [1.02, 1.45]
Chen, J. (2017)	53	60	44	60	17.0%	1.20 [1.01, 1.44]
Fan, G. H. (2012)	33	36	23	36	8.9%	1.43 [1.10, 1.87]
Qiao, J. F. (2021)	13	30	4	30	1.5%	3.25 [1.20, 8.83]
Wang, J. (2016)	22	30	18	30	7.0%	1.22 [0.85, 1.76]
Zhang, J. (2023)	41	46	35	46	13.5%	1.17 [0.97, 1.42]
Zhang, P. (2016)	25	30	17	30	6.6%	1.47 [1.03, 2.09]
Zhao, M. J. (2011)	13	20	11	18	4.5%	1.06 [0.65, 1.73]
Zhong, Q. Y. (2019)	8	30	2	30	0.8%	4.00 [0.92, 17.30]
Zhu, G. Y. (2014)	12	15	11	15	4.3%	1.09 [0.73, 1.62]
**Subtotal (95% CI)**		**332**		**330**	**74.9%**	**1.31 [1.19, 1.44]**
Total events	254		193			
Heterogeneity: Chi² = 10.76, df = 9 (P = 0.29); I² = 16%						
Test for overall effect: Z = 5.48 (P < 0.00001)						
**>60 days**						
Li, J. (2014)	22	30	19	30	7.3%	1.16 [0.82, 1.64]
Yin, X. S. (2021)	33	35	26	35	10.1%	1.27 [1.03, 1.57]
Yuan, H. W. (2018)	30	34	20	34	7.7%	1.50 [1.10, 2.04]
**Subtotal (95% CI)**		**99**		**99**	**25.1%**	**1.31 [1.11, 1.54]**
Total events	85		65			
Heterogeneity: Chi² = 1.32, df = 2 (P = 0.52); I² = 0%						
Test for overall effect: Z = 3.24 (P = 0.001)						
**Total (95% CI)**		**431**		**429**	**100.0%**	**1.31 [1.20, 1.42]**
Total events	339		258			
Heterogeneity: Chi² = 12.07, df = 12 (P = 0.44); I² = 1%						
Test for overall effect: Z = 6.36 (P < 0.00001)						
Test for subgroup differences: Chi² = 0.00, df = 1 (P = 0.99), I² = 0%						

Risk Ratio M-H, Fixed, 95% CI

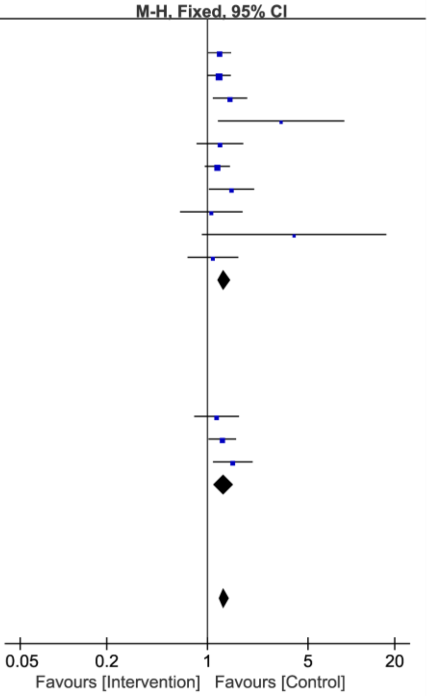

S5.4 30, 30-60, 60 days

Study or Subgroup	Intervention Events	Intervention Total	Control Events	Control Total	Weight	Risk Ratio M-H, Random, 95% CI
**≤30 days**						
Chen, J. (2017)	53	60	44	60	17.9%	1.20 [1.01, 1.44]
Fan, G. H. (2012)	33	36	23	36	8.2%	1.43 [1.10, 1.87]
Wang, J. (2016)	22	30	18	30	4.4%	1.22 [0.85, 1.76]
Zhu, G. Y. (2014)	12	15	11	15	3.7%	1.09 [0.73, 1.62]
**Subtotal (95% CI)**		**141**		**141**	**34.2%**	**1.25 [1.09, 1.42]**
Total events	120		96			
Heterogeneity: Tau² = 0.00; Chi² = 1.68, df = 3 (P = 0.64); I² = 0%						
Test for overall effect: Z = 3.32 (P = 0.0009)						
**30< days ≤60**						
Cao, M. (2024)	34	35	28	35	18.6%	1.21 [1.02, 1.45]
Qiao, J. F. (2021)	13	30	4	30	0.6%	3.25 [1.20, 8.83]
Zhang, J. (2023)	41	46	35	46	15.7%	1.17 [0.97, 1.42]
Zhang, P. (2016)	25	30	17	30	4.7%	1.47 [1.03, 2.09]
Zhao, M. J. (2011)	13	20	11	18	2.4%	1.06 [0.65, 1.73]
Zhong, Q. Y. (2019)	8	30	2	30	0.3%	4.00 [0.92, 17.30]
**Subtotal (95% CI)**		**191**		**189**	**42.1%**	**1.31 [1.06, 1.61]**
Total events	134		97			
Heterogeneity: Tau² = 0.03; Chi² = 10.36, df = 5 (P = 0.07); I² = 52%						
Test for overall effect: Z = 2.46 (P = 0.01)						
**>60 days**						
Li, J. (2014)	22	30	19	30	4.8%	1.16 [0.82, 1.64]
Yin, X. S. (2021)	33	35	26	35	12.8%	1.27 [1.03, 1.57]
Yuan, H. W. (2018)	30	34	20	34	6.1%	1.50 [1.10, 2.04]
**Subtotal (95% CI)**		**99**		**99**	**23.7%**	**1.30 [1.11, 1.52]**
Total events	85		65			
Heterogeneity: Tau² = 0.00; Chi² = 1.32, df = 2 (P = 0.52); I² = 0%						
Test for overall effect: Z = 3.31 (P = 0.0009)						
**Total (95% CI)**		**431**		**429**	**100.0%**	**1.26 [1.16, 1.36]**
Total events	339		258			
Heterogeneity: Tau² = 0.00; Chi² = 12.07, df = 12 (P = 0.44); I² = 1%						
Test for overall effect: Z = 5.88 (P < 0.00001)						
Test for subgroup differences: Chi² = 0.24, df = 2 (P = 0.89), I² = 0%						

Risk Ratio M-H, Random, 95% CI

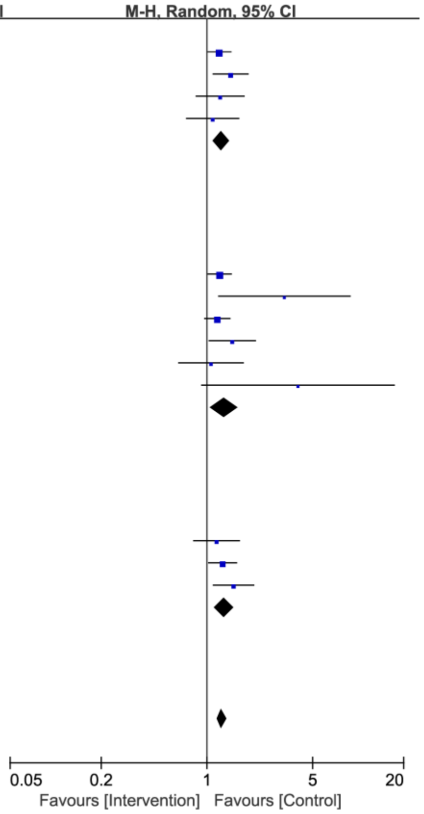

S5.5 30, 30-45, 45-60, 60 days

Study or Subgroup	Intervention Events	Intervention Total	Control Events	Control Total	Weight	Risk Ratio M-H, Random, 95% CI
**≤30 days**						
Chen, J. (2017)	53	60	44	60	17.9%	1.20 [1.01, 1.44]
Fan, G. H. (2012)	33	36	23	36	8.2%	1.43 [1.10, 1.87]
Wang, J. (2016)	22	30	18	30	4.4%	1.22 [0.85, 1.76]
Zhu, G. Y. (2014)	12	15	11	15	3.7%	1.09 [0.73, 1.62]
**Subtotal (95% CI)**		**141**		**141**	**34.2%**	**1.25 [1.09, 1.42]**
Total events	120		96			
Heterogeneity: Tau² = 0.00; Chi² = 1.68, df = 3 (P = 0.64); I² = 0%						
Test for overall effect: Z = 3.32 (P = 0.0009)						
**30＜ days ≤45**						
Zhao, M. J. (2011)	13	20	11	18	2.4%	1.06 [0.65, 1.73]
Zhong, Q. Y. (2019)	8	30	2	30	0.3%	4.00 [0.92, 17.30]
**Subtotal (95% CI)**		**50**		**48**	**2.7%**	**1.78 [0.43, 7.43]**
Total events	21		13			
Heterogeneity: Tau² = 0.81; Chi² = 3.60, df = 1 (P = 0.06); I² = 72%						
Test for overall effect: Z = 0.79 (P = 0.43)						
**45＜ days ≤60**						
Cao, M. (2024)	34	35	28	35	18.6%	1.21 [1.02, 1.45]
Qiao, J. F. (2021)	13	30	4	30	0.6%	3.25 [1.20, 8.83]
Zhang, J. (2023)	41	46	35	46	15.7%	1.17 [0.97, 1.42]
Zhang, P. (2016)	25	30	17	30	4.7%	1.47 [1.03, 2.09]
**Subtotal (95% CI)**		**141**		**141**	**39.5%**	**1.30 [1.05, 1.60]**
Total events	113		84			
Heterogeneity: Tau² = 0.02; Chi² = 6.57, df = 3 (P = 0.09); I² = 54%						
Test for overall effect: Z = 2.44 (P = 0.01)						
**＞60 days**						
Li, J. (2014)	22	30	19	30	4.8%	1.16 [0.82, 1.64]
Yin, X. S. (2021)	33	35	26	35	12.8%	1.27 [1.03, 1.57]
Yuan, H. W. (2018)	30	34	20	34	6.1%	1.50 [1.10, 2.04]
**Subtotal (95% CI)**		**99**		**99**	**23.7%**	**1.30 [1.11, 1.52]**
Total events	85		65			
Heterogeneity: Tau² = 0.00; Chi² = 1.32, df = 2 (P = 0.52); I² = 0%						
Test for overall effect: Z = 3.31 (P = 0.0009)						
**Total (95% CI)**		**431**		**429**	**100.0%**	**1.26 [1.16, 1.36]**
Total events	339		258			
Heterogeneity: Tau² = 0.00; Chi² = 12.07, df = 12 (P = 0.44); I² = 1%						
Test for overall effect: Z = 5.88 (P < 0.00001)						
Test for subgroup differences: Chi² = 0.43, df = 3 (P = 0.93), I² = 0%						

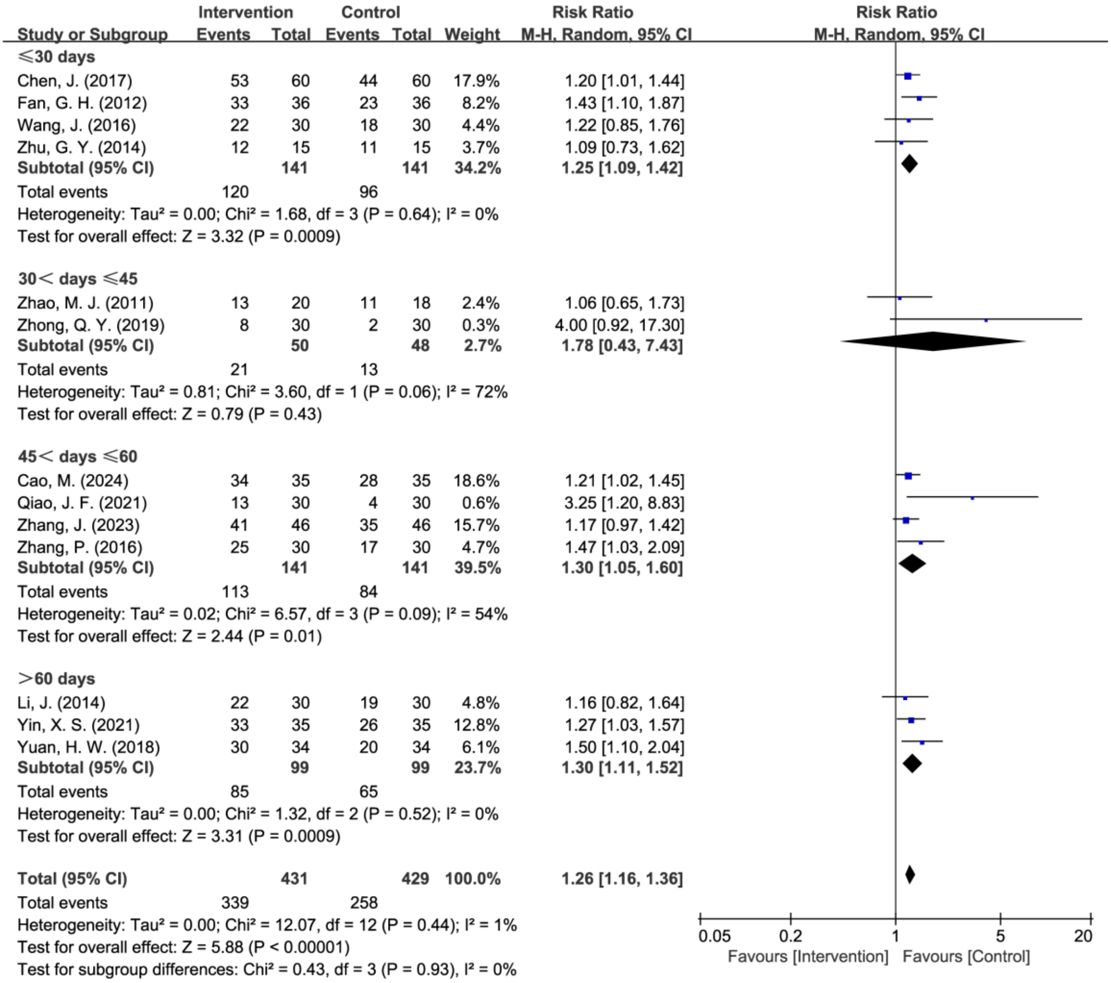

S6. TCM Syndrome Score Efficacy Analysis

S6.1 Sensitivity Analysis of Heterogeneous Sources

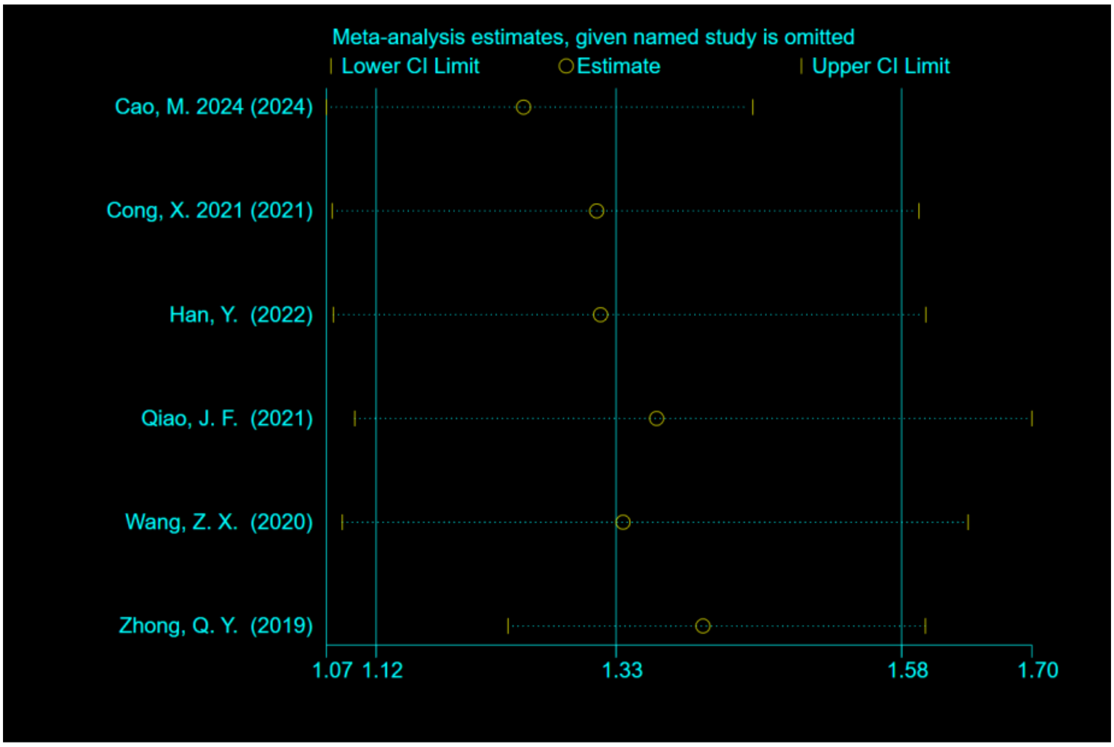

Study omitted	Estimate	[95% Conf.	Interval]
Cao, M. 2024 (2024)	1.2477095	1.072945	1.4509403
Cong, X. 2021 (2021)	1.3125085	1.0780392	1.5979741
Han, Y. (2022)	1.3158751	1.0793407	1.6042452
Qiao, J. F. (2021)	1.36559	1.0982281	1.6980406
Wang, Z. X. (2020)	1.3358605	1.0870944	1.6415529
Zhong, Q. Y. (2019)	1.4066585	1.2338338	1.6036912
Combined	1.3297006	1.1171459	1.5826972

S6.2 Remaining 5 RCTs (Exclude Zhong)

Study or Subgroup	Intervention Events	Intervention Total	Control Events	Control Total	Weight	Risk Ratio M-H, Random, 95% CI
Cao, M. (2024)	34	35	18	35	14.2%	1.89 [1.36, 2.62]
Cong, X. (2021)	39	45	27	45	16.0%	1.44 [1.11, 1.88]
Han, Y. (2022)	30	32	21	32	16.0%	1.43 [1.09, 1.86]
Qiao, J. F. (2021)	28	30	23	30	17.4%	1.22 [0.98, 1.52]
Wang, Z. X. (2020)	24	25	18	25	16.3%	1.33 [1.03, 1.72]
Zhong, Q. Y. (2019)	29	30	28	30	20.1%	1.04 [0.92, 1.16]
**Total (95% CI)**		**197**		**197**	**100.0%**	**1.34 [1.09, 1.66]**
Total events	184		135			

Heterogeneity: Tau² = 0.05; Chi² = 26.53, df = 5 (P < 0.0001); I² = 81%

Test for overall effect: Z = 2.72 (P = 0.006)

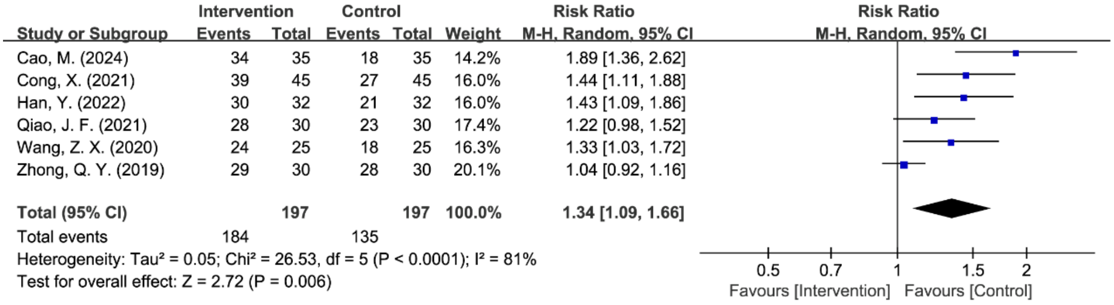

S6.3 Remaining 5 RCTs (Exclude Zhong)

Study or Subgroup	Intervention Events	Intervention Total	Control Events	Control Total	Weight	Risk Ratio M-H, Fixed, 95% CI
Cao, M. (2024)	34	35	18	35	16.8%	1.89 [1.36, 2.62]
Cong, X. (2021)	39	45	27	45	25.2%	1.44 [1.11, 1.88]
Han, Y. (2022)	30	32	21	32	19.6%	1.43 [1.09, 1.86]
Qiao, J. F. (2021)	28	30	23	30	21.5%	1.22 [0.98, 1.52]
Wang, Z. X. (2020)	24	25	18	25	16.8%	1.33 [1.03, 1.72]
**Total (95% CI)**		**167**		**167**	**100.0%**	**1.45 [1.28, 1.64]**
Total events	155		107			

Heterogeneity: Chi² = 5.36, df = 4 (P = 0.25); I² = 25%

Test for overall effect: Z = 6.00 (P < 0.00001)

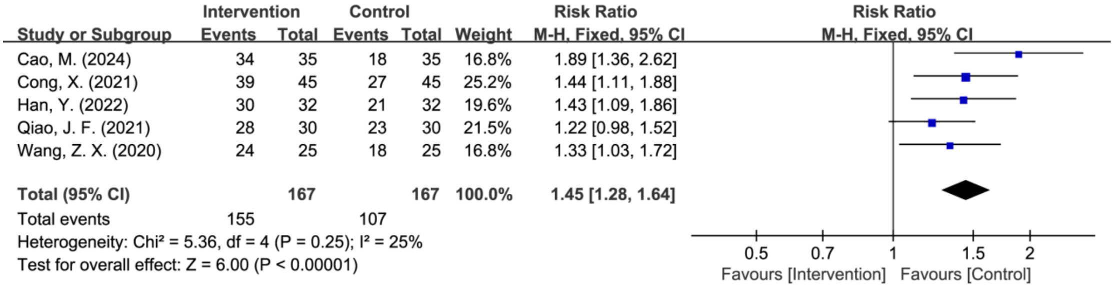

S6.4 Remaining 4 RCTs (Exclude Cao)

Study or Subgroup	Intervention Events	Intervention Total	Control Events	Control Total	Weight	Risk Ratio M-H, Random, 95% CI
Cong, X. (2021)	39	45	27	45	17.9%	1.44 [1.11, 1.88]
Han, Y. (2022)	30	32	21	32	17.8%	1.43 [1.09, 1.86]
Qiao, J. F. (2021)	28	30	23	30	20.3%	1.22 [0.98, 1.52]
Wang, Z. X. (2020)	24	25	18	25	18.3%	1.33 [1.03, 1.72]
Zhong, Q. Y. (2019)	29	30	28	30	25.7%	1.04 [0.92, 1.16]
**Total (95% CI)**		**162**		**162**	**100.0%**	**1.26 [1.05, 1.51]**
Total events	150		117			

Heterogeneity: Tau² = 0.03; Chi² = 14.61, df = 4 (P = 0.006); I² = 73%

Test for overall effect: Z = 2.48 (P = 0.01)

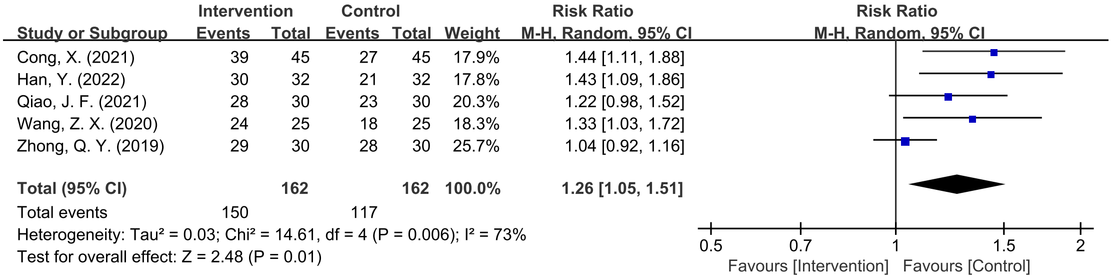

S6.5 Remaining 4 RCTs (Exclude Cao and Zhong)

Study or Subgroup	Intervention Events	Intervention Total	Control Events	Control Total	Weight	Risk Ratio M-H, Fixed, 95% CI
Cong, X. (2021)	39	45	27	45	30.3%	1.44 [1.11, 1.88]
Han, Y. (2022)	30	32	21	32	23.6%	1.43 [1.09, 1.86]
Qiao, J. F. (2021)	28	30	23	30	25.8%	1.22 [0.98, 1.52]
Wang, Z. X. (2020)	24	25	18	25	20.2%	1.33 [1.03, 1.72]
**Total (95% CI)**		**132**		**132**	**100.0%**	**1.36 [1.20, 1.55]**
Total events	121		89			

Heterogeneity: Chi² = 1.33, df = 3 (P = 0.72); I² = 0%

Test for overall effect: Z = 4.68 (P < 0.00001)

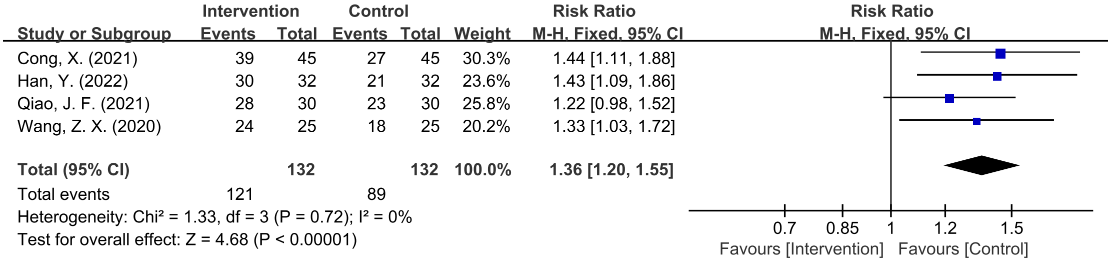

S6.6 Remaining 4 RCTs (Exclude Qiao and Zhong)

Study or Subgroup	Intervention Events	Intervention Total	Control Events	Control Total	Weight	Risk Ratio M-H, Fixed, 95% CI
Cao, M. (2024)	34	35	18	35	21.4%	1.89 [1.36, 2.62]
Cong, X. (2021)	39	45	27	45	32.1%	1.44 [1.11, 1.88]
Han, Y. (2022)	30	32	21	32	25.0%	1.43 [1.09, 1.86]
Wang, Z. X. (2020)	24	25	18	25	21.4%	1.33 [1.03, 1.72]
**Total (95% CI)**		**137**		**137**	**100.0%**	**1.51 [1.31, 1.74]**
Total events	127		84			

Heterogeneity: Chi² = 2.99, df = 3 (P = 0.39); I² = 0%

Test for overall effect: Z = 5.74 (P < 0.00001)

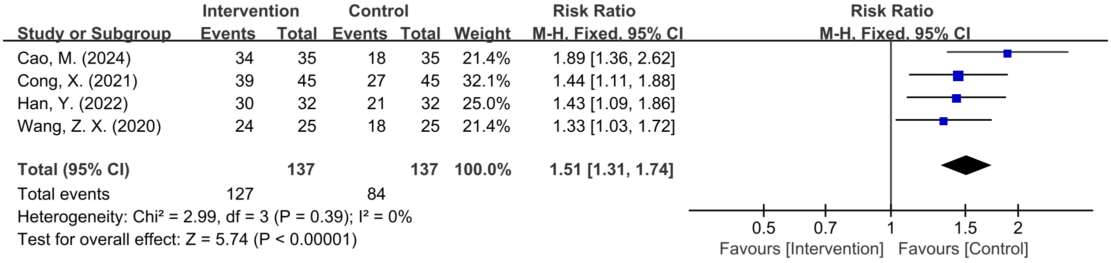

S7 TCM Syndrome Score Efficacy Analysis based on Subgroups of Treatment Duration

S7.1 30 days

Study or Subgroup	Intervention Events	Intervention Total	Control Events	Control Total	Weight	Risk Ratio M-H, Random, 95% CI
**≤30 days**						
Wang, Z. X. (2020)	24	25	18	25	16.3%	1.33 [1.03, 1.72]
**Subtotal (95% CI)**		**25**		**25**	**16.3%**	**1.33 [1.03, 1.72]**
Total events	24		18			
Heterogeneity: Not applicable						
Test for overall effect: Z = 2.19 (P = 0.03)						
**>30 days**						
Cao, M. (2024)	34	35	18	35	14.2%	1.89 [1.36, 2.62]
Cong, X. (2021)	39	45	27	45	16.0%	1.44 [1.11, 1.88]
Han, Y. (2022)	30	32	21	32	16.0%	1.43 [1.09, 1.86]
Qiao, J. F. (2021)	28	30	23	30	17.4%	1.22 [0.98, 1.52]
Zhong, Q. Y. (2019)	29	30	28	30	20.1%	1.04 [0.92, 1.16]
**Subtotal (95% CI)**		**172**		**172**	**83.7%**	**1.35 [1.04, 1.75]**
Total events	160		117			
Heterogeneity: $Tau^2 = 0.07$; $Chi^2 = 26.97$, df = 4 (P < 0.0001); $I^2 = 85\%$						
Test for overall effect: Z = 2.28 (P = 0.02)						
**Total (95% CI)**		**197**		**197**	**100.0%**	**1.34 [1.09, 1.66]**
Total events	184		135			
Heterogeneity: $Tau^2 = 0.05$; $Chi^2 = 26.53$, df = 5 (P < 0.0001); $I^2 = 81\%$						
Test for overall effect: Z = 2.72 (P = 0.006)						
Test for subgroup differences: $Chi^2 = 0.00$, df = 1 (P = 0.95), $I^2 = 0\%$						

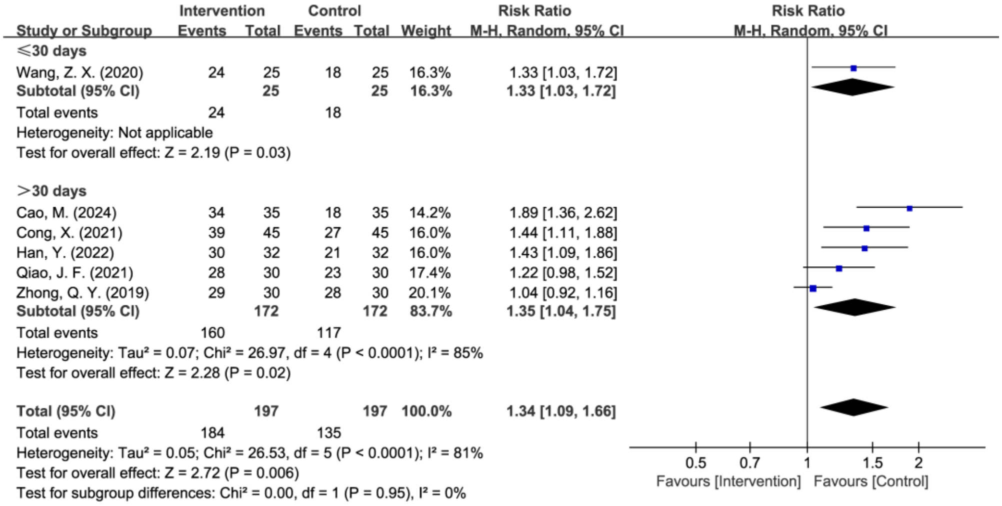

S7.2 45 days

Study or Subgroup	Intervention Events	Intervention Total	Control Events	Control Total	Weight	Risk Ratio M-H, Random, 95% CI
**≤45 days**						
Wang, Z. X. (2020)	24	25	18	25	16.3%	1.33 [1.03, 1.72]
Zhong, Q. Y. (2019)	29	30	28	30	20.1%	1.04 [0.92, 1.16]
**Subtotal (95% CI)**		**55**		**55**	**36.4%**	**1.15 [0.86, 1.55]**
Total events	53		46			
Heterogeneity: $Tau^2 = 0.04$; $Chi^2 = 4.45$, df = 1 (P = 0.03); $I^2 = 78\%$						
Test for overall effect: Z = 0.95 (P = 0.34)						
**>45 days**						
Cao, M. (2024)	34	35	18	35	14.2%	1.89 [1.36, 2.62]
Cong, X. (2021)	39	45	27	45	16.0%	1.44 [1.11, 1.88]
Han, Y. (2022)	30	32	21	32	16.0%	1.43 [1.09, 1.86]
Qiao, J. F. (2021)	28	30	23	30	17.4%	1.22 [0.98, 1.52]
**Subtotal (95% CI)**		**142**		**142**	**63.6%**	**1.44 [1.21, 1.71]**
Total events	131		89			
Heterogeneity: $Tau^2 = 0.01$; $Chi^2 = 5.18$, df = 3 (P = 0.16); $I^2 = 42\%$						
Test for overall effect: Z = 4.09 (P < 0.0001)						
**Total (95% CI)**		**197**		**197**	**100.0%**	**1.34 [1.09, 1.66]**
Total events	184		135			
Heterogeneity: $Tau^2 = 0.05$; $Chi^2 = 26.53$, df = 5 (P < 0.0001); $I^2 = 81\%$						
Test for overall effect: Z = 2.72 (P = 0.006)						
Test for subgroup differences: $Chi^2 = 1.61$, df = 1 (P = 0.20), $I^2 = 37.8\%$						

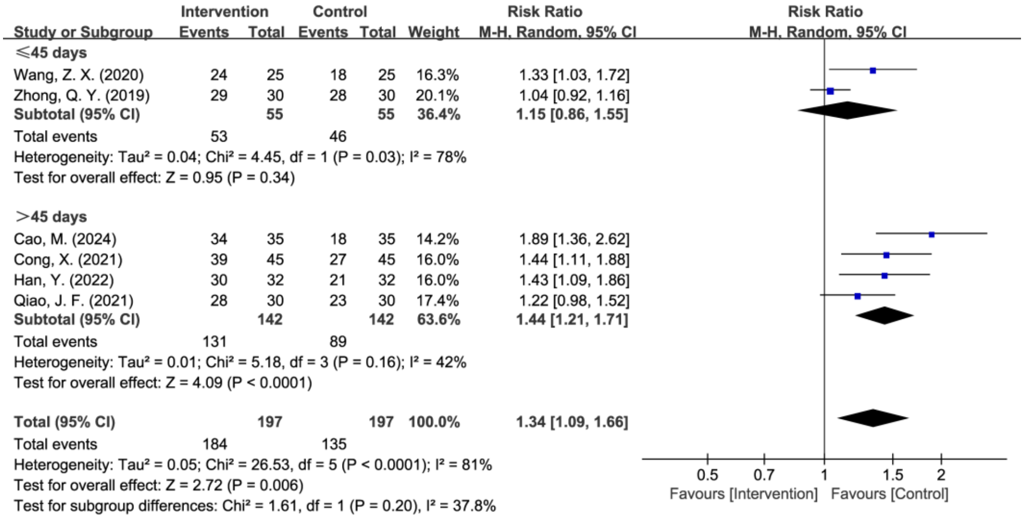

S7.3 60 days

Study or Subgroup	Intervention Events	Intervention Total	Control Events	Control Total	Weight	Risk Ratio M-H, Random, 95% CI
**≤60 days**						
Cao, M. (2024)	34	35	18	35	14.2%	1.89 [1.36, 2.62]
Han, Y. (2022)	30	32	21	32	16.0%	1.43 [1.09, 1.86]
Qiao, J. F. (2021)	28	30	23	30	17.4%	1.22 [0.98, 1.52]
Wang, Z. X. (2020)	24	25	18	25	16.3%	1.33 [1.03, 1.72]
Zhong, Q. Y. (2019)	29	30	28	30	20.1%	1.04 [0.92, 1.16]
**Subtotal (95% CI)**		**152**		**152**	**84.0%**	**1.33 [1.04, 1.69]**
Total events	145		108			
Heterogeneity: $Tau^2 = 0.06$; $Chi^2 = 24.24$, df = 4 (P < 0.0001); $I^2 = 83\%$						
Test for overall effect: Z = 2.28 (P = 0.02)						
**>60 days**						
Cong, X. (2021)	39	45	27	45	16.0%	1.44 [1.11, 1.88]
**Subtotal (95% CI)**		**45**		**45**	**16.0%**	**1.44 [1.11, 1.88]**
Total events	39		27			
Heterogeneity: Not applicable						
Test for overall effect: Z = 2.72 (P = 0.006)						
**Total (95% CI)**		**197**		**197**	**100.0%**	**1.34 [1.09, 1.66]**
Total events	184		135			
Heterogeneity: $Tau^2 = 0.05$; $Chi^2 = 26.53$, df = 5 (P < 0.0001); $I^2 = 81\%$						
Test for overall effect: Z = 2.72 (P = 0.006)						
Test for subgroup differences: $Chi^2 = 0.22$, df = 1 (P = 0.64), $I^2 = 0\%$						

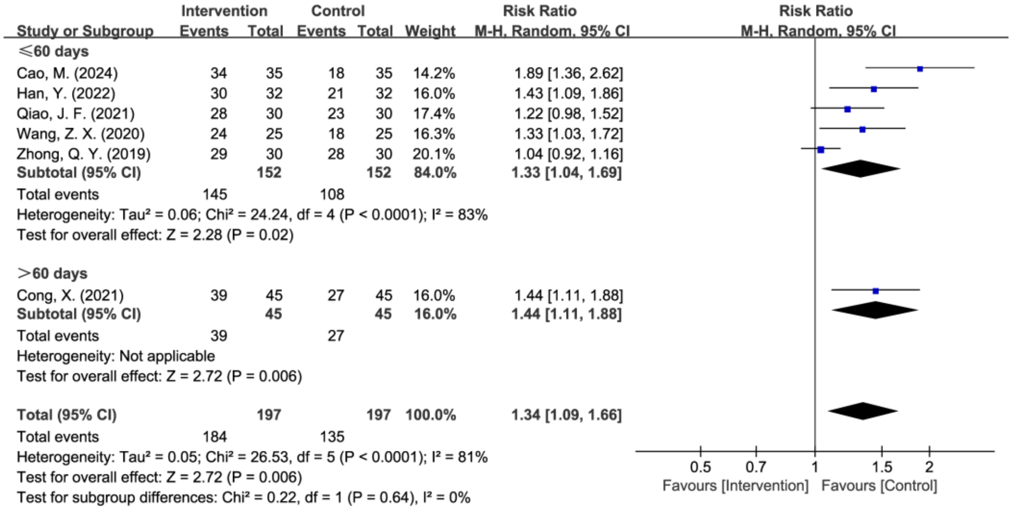

S7.4 30, 30-60, 60 days

Study or Subgroup	Intervention Events	Intervention Total	Control Events	Control Total	Weight	Risk Ratio M-H, Random, 95% CI
**≤30 days**						
Wang, Z. X. (2020)	24	25	18	25	16.3%	1.33 [1.03, 1.72]
**Subtotal (95% CI)**		**25**		**25**	**16.3%**	**1.33 [1.03, 1.72]**
Total events	24		18			
Heterogeneity: Not applicable						
Test for overall effect: Z = 2.19 (P = 0.03)						
**30< days ≤60**						
Cao, M. (2024)	34	35	18	35	14.2%	1.89 [1.36, 2.62]
Han, Y. (2022)	30	32	21	32	16.0%	1.43 [1.09, 1.86]
Qiao, J. F. (2021)	28	30	23	30	17.4%	1.22 [0.98, 1.52]
Zhong, Q. Y. (2019)	29	30	28	30	20.1%	1.04 [0.92, 1.16]
**Subtotal (95% CI)**		**127**		**127**	**67.7%**	**1.33 [0.98, 1.81]**
Total events	121		90			
Heterogeneity: Tau² = 0.08; Chi² = 24.42, df = 3 (P < 0.0001); I² = 88%						
Test for overall effect: Z = 1.82 (P = 0.07)						
**>60 days**						
Cong, X. (2021)	39	45	27	45	16.0%	1.44 [1.11, 1.88]
**Subtotal (95% CI)**		**45**		**45**	**16.0%**	**1.44 [1.11, 1.88]**
Total events	39		27			
Heterogeneity: Not applicable						
Test for overall effect: Z = 2.72 (P = 0.006)						
**Total (95% CI)**		**197**		**197**	**100.0%**	**1.34 [1.09, 1.66]**
Total events	184		135			
Heterogeneity: Tau² = 0.05; Chi² = 26.53, df = 5 (P < 0.0001); I² = 81%						
Test for overall effect: Z = 2.72 (P = 0.006)						
Test for subgroup differences: Chi² = 0.23, df = 2 (P = 0.89), I² = 0%						

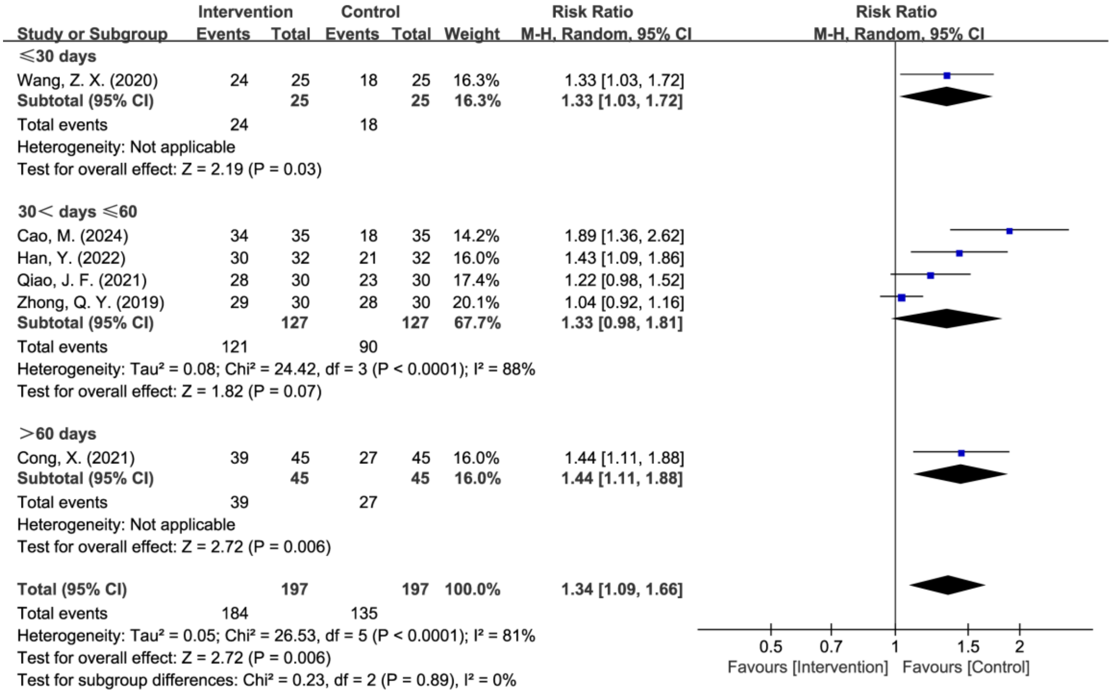

S7.5 30, 30-45, 45-60, 60 days

Study or Subgroup	Intervention Events	Intervention Total	Control Events	Control Total	Weight	Risk Ratio M-H, Fixed, 95% CI
**≤30 days**						
Wang, Z. X. (2020)	24	25	18	25	13.3%	1.33 [1.03, 1.72]
**Subtotal (95% CI)**		**25**		**25**	**13.3%**	**1.33 [1.03, 1.72]**
Total events	24		18			
Heterogeneity: Not applicable						
Test for overall effect: Z = 2.19 (P = 0.03)						
**30< days ≤45**						
Zhong, Q. Y. (2019)	29	30	28	30	20.7%	1.04 [0.92, 1.16]
**Subtotal (95% CI)**		**30**		**30**	**20.7%**	**1.04 [0.92, 1.16]**
Total events	29		28			
Heterogeneity: Not applicable						
Test for overall effect: Z = 0.59 (P = 0.55)						
**45< days ≤60**						
Cao, M. (2024)	34	35	18	35	13.3%	1.89 [1.36, 2.62]
Han, Y. (2022)	30	32	21	32	15.6%	1.43 [1.09, 1.86]
Qiao, J. F. (2021)	28	30	23	30	17.0%	1.22 [0.98, 1.52]
**Subtotal (95% CI)**		**97**		**97**	**45.9%**	**1.48 [1.27, 1.74]**
Total events	92		62			
Heterogeneity: Chi² = 5.30, df = 2 (P = 0.07); I² = 62%						
Test for overall effect: Z = 4.92 (P < 0.00001)						
**>60 days**						
Cong, X. (2021)	39	45	27	45	20.0%	1.44 [1.11, 1.88]
**Subtotal (95% CI)**		**45**		**45**	**20.0%**	**1.44 [1.11, 1.88]**
Total events	39		27			
Heterogeneity: Not applicable						
Test for overall effect: Z = 2.72 (P = 0.006)						
**Total (95% CI)**		**197**		**197**	**100.0%**	**1.36 [1.23, 1.51]**
Total events	184		135			
Heterogeneity: Chi² = 26.53, df = 5 (P < 0.0001); I² = 81%						
Test for overall effect: Z = 6.00 (P < 0.00001)						
Test for subgroup differences: Chi² = 15.57, df = 3 (P = 0.001), I² = 80.7%						

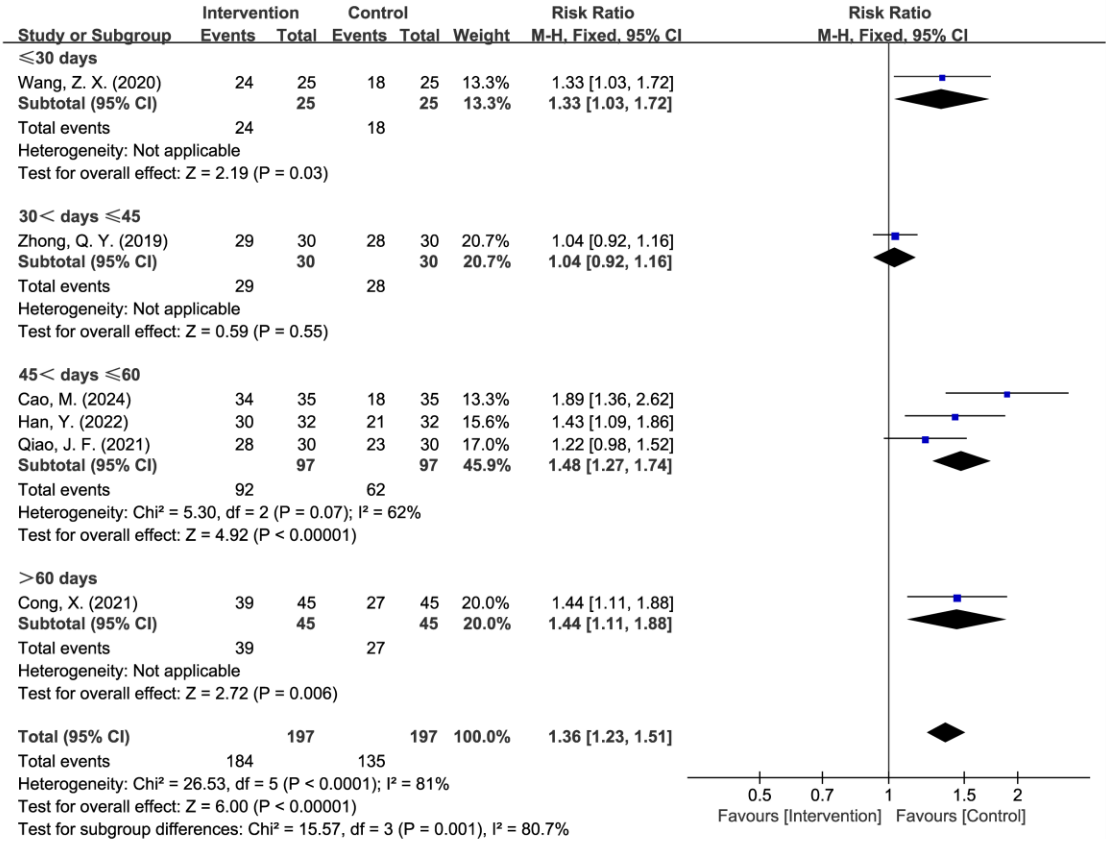

S8. TCM Syndrome Score Efficacy Analysis for Deleting Wang, Z. X. (2020)' High-risk Articles

S8.1 Sensitivity Analysis for Deleting Wang, Z. X. (2020)' High-risk Articles

TCM Syndrome Score Efficacy<br>Study or Subgroup	Intervention<br>Events	 Total	Control<br>Events	 Total	 Weight	Risk Ratio<br>M-H, Random, 95% CI
Cao, M. (2024)	34	35	18	35	17.5%	1.89 [1.36, 2.62]
Cong, X. (2021)	39	45	27	45	19.4%	1.44 [1.11, 1.88]
Han, Y. (2022)	30	32	21	32	19.3%	1.43 [1.09, 1.86]
Qiao, J. F. (2021)	28	30	23	30	20.7%	1.22 [0.98, 1.52]
Zhong, Q. Y. (2019)	29	30	28	30	23.2%	1.04 [0.92, 1.16]
**Total (95% CI)**		**172**		**172**	**100.0%**	**1.35 [1.04, 1.75]**
Total events	160		117			
Heterogeneity: $Tau^2 = 0.07$; $Chi^2 = 26.97$, df = 4 (P < 0.0001); $I^2 = 85\%$						
Test for overall effect: Z = 2.28 (P = 0.02)						

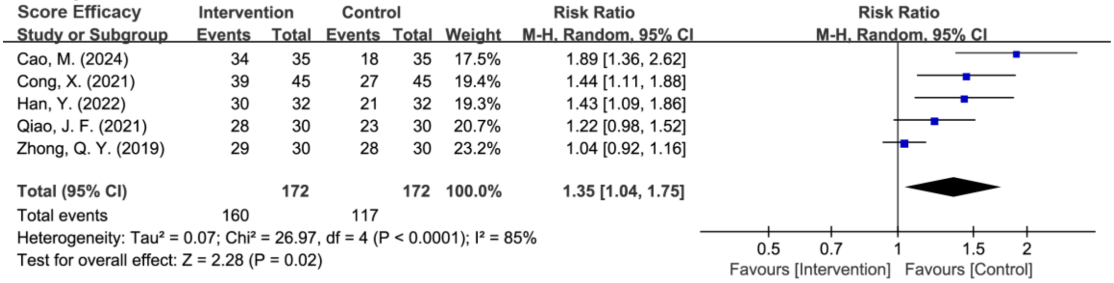

S8.2 Sensitivity Analysis for based on Subgroup Analysis of Intervention Strategies

TCM Syndrome Score Efficacy<br>Study or Subgroup	Intervention<br>Events	 Total	Control<br>Events	 Total	 Weight	Risk Ratio<br>M-H, Random, 95% CI
**TCM+BM**						
Cao, M. (2024)	34	35	18	35	17.5%	1.89 [1.36, 2.62]
Cong, X. (2021)	39	45	27	45	19.4%	1.44 [1.11, 1.88]
Han, Y. (2022)	30	32	21	32	19.3%	1.43 [1.09, 1.86]
**Subtotal (95% CI)**		**112**		**112**	**56.1%**	**1.54 [1.30, 1.81]**
Total events	103		66			
Heterogeneity: $Tau^2 = 0.00$; $Chi^2 = 2.06$, df = 2 (P = 0.36); $I^2 = 3\%$						
Test for overall effect: Z = 5.10 (P < 0.00001)						
**TCM-alone**						
Qiao, J. F. (2021)	28	30	23	30	20.7%	1.22 [0.98, 1.52]
Zhong, Q. Y. (2019)	29	30	28	30	23.2%	1.04 [0.92, 1.16]
**Subtotal (95% CI)**		**60**		**60**	**43.9%**	**1.10 [0.92, 1.32]**
Total events	57		51			
Heterogeneity: $Tau^2 = 0.01$; $Chi^2 = 2.23$, df = 1 (P = 0.14); $I^2 = 55\%$						
Test for overall effect: Z = 1.04 (P = 0.30)						
**Total (95% CI)**		**172**		**172**	**100.0%**	**1.35 [1.04, 1.75]**
Total events	160		117			
Heterogeneity: $Tau^2 = 0.07$; $Chi^2 = 26.97$, df = 4 (P < 0.0001); $I^2 = 85\%$						
Test for overall effect: Z = 2.28 (P = 0.02)						
Test for subgroup differences: $Chi^2 = 7.25$, df = 1 (P = 0.007), $I^2 = 86.2\%$						

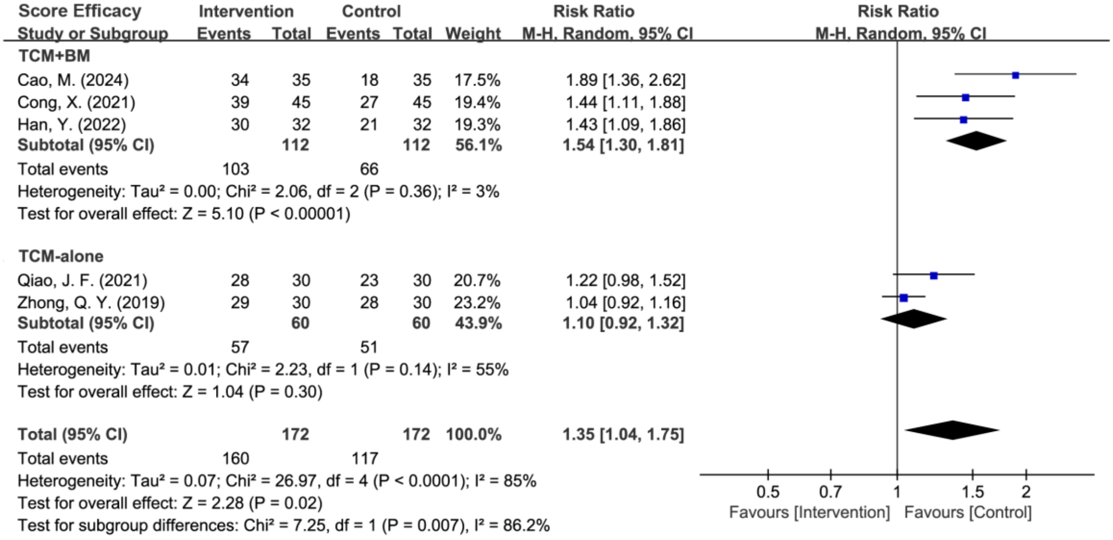

S8.3 45 days

TCM Syndrome Score Efficacy<br>Study or Subgroup	Intervention<br>Events	 Total	Control<br>Events	 Total	 Weight	Risk Ratio<br>M-H, Random, 95% CI
**≤45 days**						
Zhong, Q. Y. (2019)	29	30	28	30	23.2%	1.04 [0.92, 1.16]
**Subtotal (95% CI)**		**30**		**30**	**23.2%**	**1.04 [0.92, 1.16]**
Total events	29		28			
Heterogeneity: Not applicable						
Test for overall effect: Z = 0.59 (P = 0.55)						
**>45 days**						
Cao, M. (2024)	34	35	18	35	17.5%	1.89 [1.36, 2.62]
Cong, X. (2021)	39	45	27	45	19.4%	1.44 [1.11, 1.88]
Han, Y. (2022)	30	32	21	32	19.3%	1.43 [1.09, 1.86]
Qiao, J. F. (2021)	28	30	23	30	20.7%	1.22 [0.98, 1.52]
**Subtotal (95% CI)**		**142**		**142**	**76.8%**	**1.44 [1.21, 1.71]**
Total events	131		89			
Heterogeneity: $Tau^2 = 0.01$; $Chi^2 = 5.18$, df = 3 (P = 0.16); $I^2 = 42\%$						
Test for overall effect: Z = 4.09 (P < 0.0001)						
**Total (95% CI)**		**172**		**172**	**100.0%**	**1.35 [1.04, 1.75]**
Total events	160		117			
Heterogeneity: $Tau^2 = 0.07$; $Chi^2 = 26.97$, df = 4 (P < 0.0001); $I^2 = 85\%$						
Test for overall effect: Z = 2.28 (P = 0.02)						
Test for subgroup differences: $Chi^2 = 9.46$, df = 1 (P = 0.002), $I^2 = 89.4\%$						

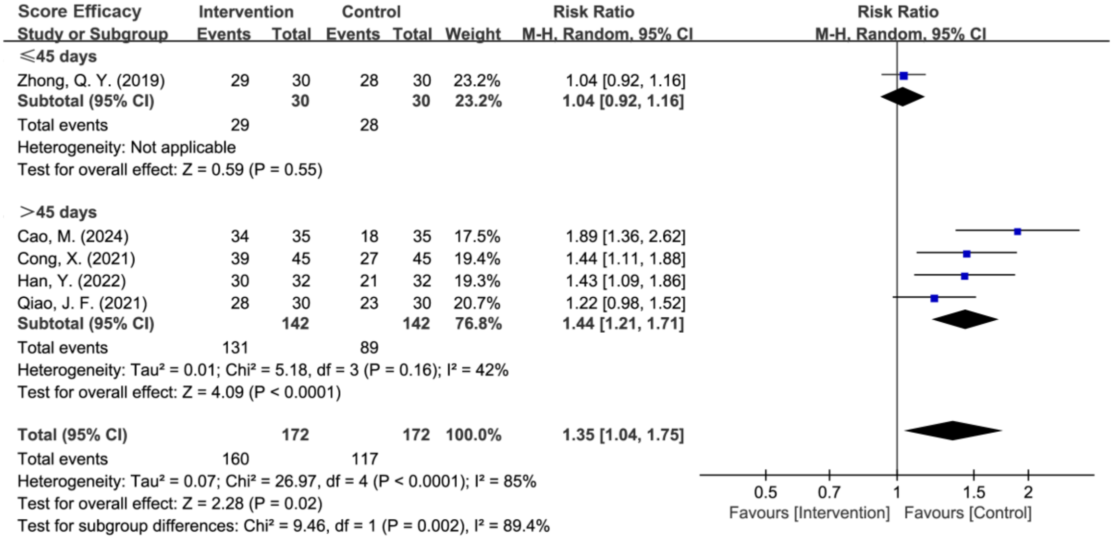

S8.4 60 days

TCM Syndrome Score Efficacy<br>Study or Subgroup	Intervention<br>Events	Intervention<br>Total	Control<br>Events	Control<br>Total	Weight	Risk Ratio<br>M-H, Random, 95% CI
**≤60 days**						
Cao, M. (2024)	34	35	18	35	17.5%	1.89 [1.36, 2.62]
Han, Y. (2022)	30	32	21	32	19.3%	1.43 [1.09, 1.86]
Qiao, J. F. (2021)	28	30	23	30	20.7%	1.22 [0.98, 1.52]
Zhong, Q. Y. (2019)	29	30	28	30	23.2%	1.04 [0.92, 1.16]
**Subtotal (95% CI)**		**127**		**127**	**80.6%**	**1.33 [0.98, 1.81]**
Total events	121		90			
Heterogeneity: Tau² = 0.08; Chi² = 24.42, df = 3 (P < 0.0001); I² = 88%						
Test for overall effect: Z = 1.82 (P = 0.07)						
**>60 days**						
Cong, X. (2021)	39	45	27	45	19.4%	1.44 [1.11, 1.88]
**Subtotal (95% CI)**		**45**		**45**	**19.4%**	**1.44 [1.11, 1.88]**
Total events	39		27			
Heterogeneity: Not applicable						
Test for overall effect: Z = 2.72 (P = 0.006)						
**Total (95% CI)**		**172**		**172**	**100.0%**	**1.35 [1.04, 1.75]**
Total events	160		117			
Heterogeneity: Tau² = 0.07; Chi² = 26.97, df = 4 (P < 0.0001); I² = 85%						
Test for overall effect: Z = 2.28 (P = 0.02)						
Test for subgroup differences: Chi² = 0.15, df = 1 (P = 0.70), I² = 0%						

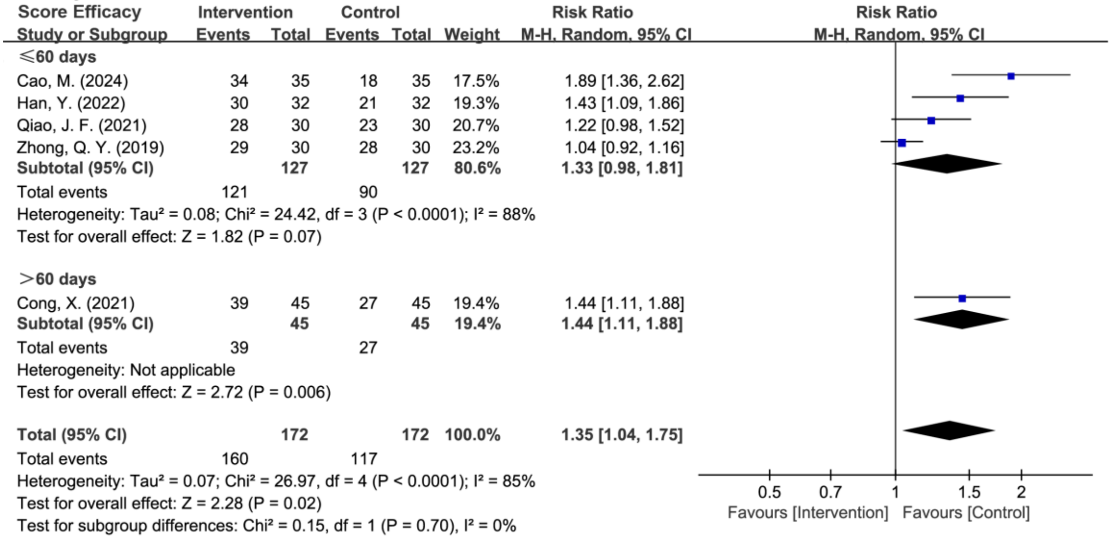

S8.5 30-45, 45-60, 60 days

TCM Syndrome Score Efficacy<br>Study or Subgroup	Intervention<br>Events	Intervention<br>Total	Control<br>Events	Control<br>Total	Weight	Risk Ratio<br>M-H, Random, 95% CI
**30< days ≤45**						
Zhong, Q. Y. (2019)	29	30	28	30	23.2%	1.04 [0.92, 1.16]
**Subtotal (95% CI)**		**30**		**30**	**23.2%**	**1.04 [0.92, 1.16]**
Total events	29		28			
Heterogeneity: Not applicable						
Test for overall effect: Z = 0.59 (P = 0.55)						
**45< days ≤60**						
Cao, M. (2024)	34	35	18	35	17.5%	1.89 [1.36, 2.62]
Han, Y. (2022)	30	32	21	32	19.3%	1.43 [1.09, 1.86]
Qiao, J. F. (2021)	28	30	23	30	20.7%	1.22 [0.98, 1.52]
**Subtotal (95% CI)**		**97**		**97**	**57.5%**	**1.45 [1.13, 1.87]**
Total events	92		62			
Heterogeneity: Tau² = 0.03; Chi² = 5.30, df = 2 (P = 0.07); I² = 62%						
Test for overall effect: Z = 2.91 (P = 0.004)						
**>60 days**						
Cong, X. (2021)	39	45	27	45	19.4%	1.44 [1.11, 1.88]
**Subtotal (95% CI)**		**45**		**45**	**19.4%**	**1.44 [1.11, 1.88]**
Total events	39		27			
Heterogeneity: Not applicable						
Test for overall effect: Z = 2.72 (P = 0.006)						
**Total (95% CI)**		**172**		**172**	**100.0%**	**1.35 [1.04, 1.75]**
Total events	160		117			
Heterogeneity: Tau² = 0.07; Chi² = 26.97, df = 4 (P < 0.0001); I² = 85%						
Test for overall effect: Z = 2.28 (P = 0.02)						
Test for subgroup differences: Chi² = 9.27, df = 2 (P = 0.010), I² = 78.4%						

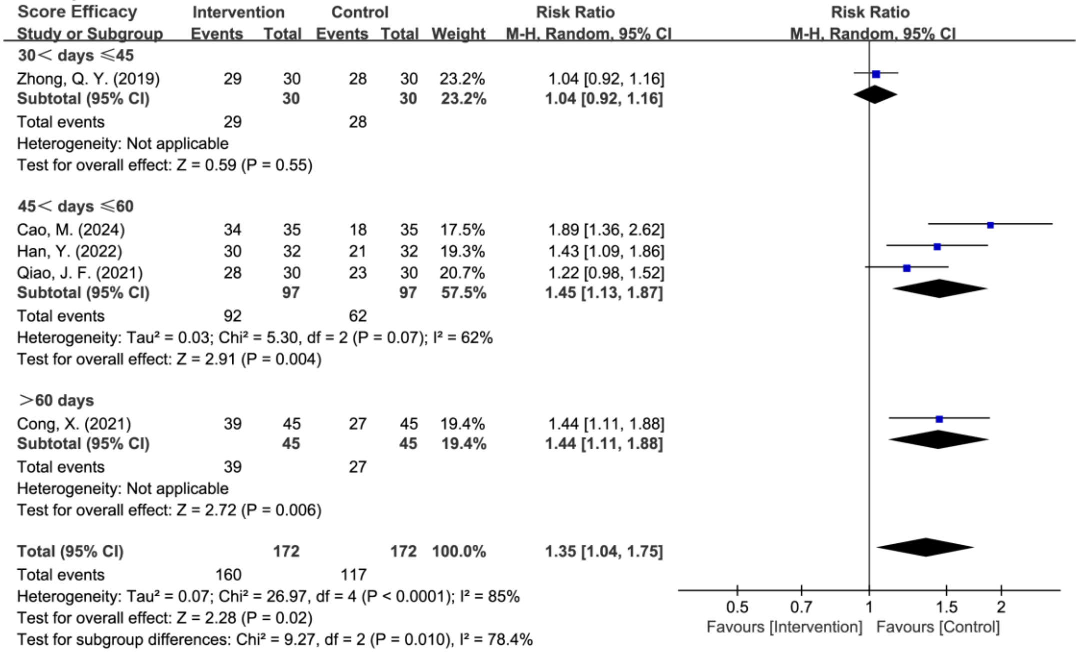

S9. Adverse Reactions Analysis

S9.1 Adverse Reactions Analysis based on Subgroups of Interventions

Study or Subgroup	Intervention Events	Intervention Total	Control Events	Control Total	Weight	Risk Ratio M-H, Fixed, 95% CI
**TCM+BM**						
Cao, M. (2024)	5	35	4	35	13.5%	1.25 [0.37, 4.27]
Cong, X. (2021)	2	45	3	45	10.1%	0.67 [0.12, 3.80]
Li, J. (2014)	4	30	4	30	13.5%	1.00 [0.28, 3.63]
Wang, J. (2016)	0	30	3	30	11.8%	0.14 [0.01, 2.65]
Yin, X. S. (2021)	1	35	1	35	3.4%	1.00 [0.07, 15.36]
Yuan, H. W. (2018)	11	34	10	34	33.7%	1.10 [0.54, 2.24]
**Subtotal (95% CI)**		**209**		**209**	**85.8%**	**0.92 [0.56, 1.53]**
Total events	23		25			
Heterogeneity: Chi² = 2.19, df = 5 (P = 0.82); I² = 0%						
Test for overall effect: Z = 0.32 (P = 0.75)						
**TCM-alone**						
Zhao, M. J. (2011)	1	20	4	18	14.2%	0.23 [0.03, 1.83]
**Subtotal (95% CI)**		**20**		**18**	**14.2%**	**0.23 [0.03, 1.83]**
Total events	1		4			
Heterogeneity: Not applicable						
Test for overall effect: Z = 1.39 (P = 0.16)						
**Total (95% CI)**		**229**		**227**	**100.0%**	**0.82 [0.51, 1.34]**
Total events	24		29			
Heterogeneity: Chi² = 4.10, df = 6 (P = 0.66); I² = 0%						
Test for overall effect: Z = 0.79 (P = 0.43)						
Test for subgroup differences: Chi² = 1.64, df = 1 (P = 0.20), I² = 39.1%						

Risk Ratio
M-H, Fixed, 95% CI

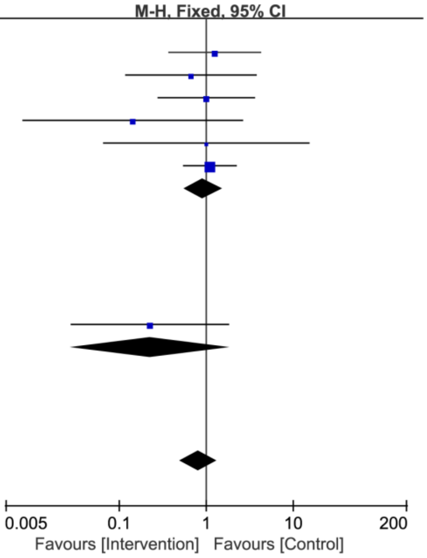

S10 Adverse Reactions Analysis based on Subgroups of Treatment Duration

S10.1 30 days

Study or Subgroup	Intervention Events	Intervention Total	Control Events	Control Total	Weight	Risk Ratio M-H, Fixed, 95% CI
**≤30 days**						
Wang, J. (2016)	0	30	3	30	11.8%	0.14 [0.01, 2.65]
**Subtotal (95% CI)**		**30**		**30**	**11.8%**	**0.14 [0.01, 2.65]**
Total events	0		3			
Heterogeneity: Not applicable						
Test for overall effect: Z = 1.31 (P = 0.19)						
**>30 days**						
Cao, M. (2024)	5	35	4	35	13.5%	1.25 [0.37, 4.27]
Cong, X. (2021)	2	45	3	45	10.1%	0.67 [0.12, 3.80]
Li, J. (2014)	4	30	4	30	13.5%	1.00 [0.28, 3.63]
Yin, X. S. (2021)	1	35	1	35	3.4%	1.00 [0.07, 15.36]
Yuan, H. W. (2018)	11	34	10	34	33.7%	1.10 [0.54, 2.24]
Zhao, M. J. (2011)	1	20	4	18	14.2%	0.23 [0.03, 1.83]
**Subtotal (95% CI)**		**199**		**197**	**88.2%**	**0.91 [0.56, 1.50]**
Total events	24		26			
Heterogeneity: Chi² = 2.38, df = 5 (P = 0.80); I² = 0%						
Test for overall effect: Z = 0.36 (P = 0.72)						
**Total (95% CI)**		**229**		**227**	**100.0%**	**0.82 [0.51, 1.34]**
Total events	24		29			
Heterogeneity: Chi² = 4.10, df = 6 (P = 0.66); I² = 0%						
Test for overall effect: Z = 0.79 (P = 0.43)						
Test for subgroup differences: Chi² = 1.51, df = 1 (P = 0.22), I² = 33.6%						

Risk Ratio M-H, Fixed, 95% CI

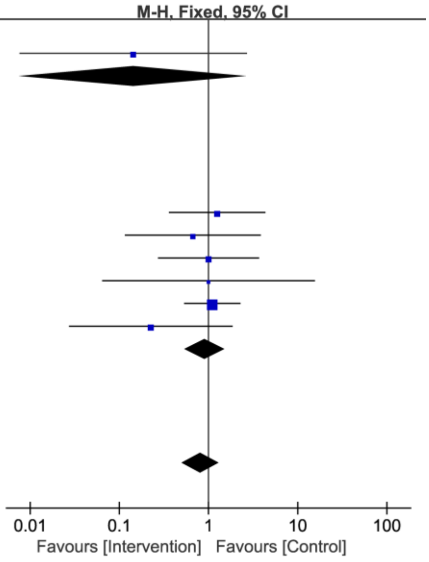

S10.2 45 days

Study or Subgroup	Intervention Events	Intervention Total	Control Events	Control Total	Weight	Risk Ratio M-H, Random, 95% CI
**≤45 days**						
Wang, J. (2016)	0	30	3	30	2.9%	0.14 [0.01, 2.65]
Zhao, M. J. (2011)	1	20	4	18	5.6%	0.23 [0.03, 1.83]
**Subtotal (95% CI)**		**50**		**48**	**8.5%**	**0.19 [0.04, 1.06]**
Total events	1		7			
Heterogeneity: Tau² = 0.00; Chi² = 0.06, df = 1 (P = 0.80); I² = 0%						
Test for overall effect: Z = 1.89 (P = 0.06)						
**>45 days**						
Cao, M. (2024)	5	35	4	35	16.4%	1.25 [0.37, 4.27]
Cong, X. (2021)	2	45	3	45	8.2%	0.67 [0.12, 3.80]
Li, J. (2014)	4	30	4	30	14.9%	1.00 [0.28, 3.63]
Yin, X. S. (2021)	1	35	1	35	3.3%	1.00 [0.07, 15.36]
Yuan, H. W. (2018)	11	34	10	34	48.7%	1.10 [0.54, 2.24]
**Subtotal (95% CI)**		**179**		**179**	**91.5%**	**1.06 [0.63, 1.78]**
Total events	23		22			
Heterogeneity: Tau² = 0.00; Chi² = 0.36, df = 4 (P = 0.99); I² = 0%						
Test for overall effect: Z = 0.21 (P = 0.84)						
**Total (95% CI)**		**229**		**227**	**100.0%**	**0.91 [0.56, 1.50]**
Total events	24		29			
Heterogeneity: Tau² = 0.00; Chi² = 4.10, df = 6 (P = 0.66); I² = 0%						
Test for overall effect: Z = 0.36 (P = 0.72)						
Test for subgroup differences: Chi² = 3.50, df = 1 (P = 0.06), I² = 71.5%						

Risk Ratio M-H, Random, 95% CI

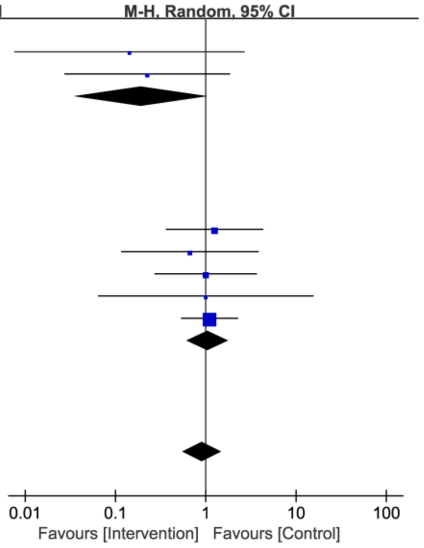

S10.3 60 days (Fixed Model)

Study or Subgroup	Intervention Events	Intervention Total	Control Events	Control Total	Weight	Risk Ratio M-H, Fixed, 95% CI
**≤60 days**						
Cao, M. (2024)	5	35	4	35	13.5%	1.25 [0.37, 4.27]
Wang, J. (2016)	0	30	3	30	11.8%	0.14 [0.01, 2.65]
Zhao, M. J. (2011)	1	20	4	18	14.2%	0.23 [0.03, 1.83]
**Subtotal (95% CI)**		**85**		**83**	**39.4%**	**0.55 [0.22, 1.37]**
Total events	6		11			
Heterogeneity: Chi² = 3.23, df = 2 (P = 0.20); I² = 38%						
Test for overall effect: Z = 1.28 (P = 0.20)						
**>60 days**						
Cong, X. (2021)	2	45	3	45	10.1%	0.67 [0.12, 3.80]
Li, J. (2014)	4	30	4	30	13.5%	1.00 [0.28, 3.63]
Yin, X. S. (2021)	1	35	1	35	3.4%	1.00 [0.07, 15.36]
Yuan, H. W. (2018)	11	34	10	34	33.7%	1.10 [0.54, 2.24]
**Subtotal (95% CI)**		**144**		**144**	**60.6%**	**1.00 [0.56, 1.78]**
Total events	18		18			
Heterogeneity: Chi² = 0.28, df = 3 (P = 0.96); I² = 0%						
Test for overall effect: Z = 0.00 (P = 1.00)						
**Total (95% CI)**		**229**		**227**	**100.0%**	**0.82 [0.51, 1.34]**
Total events	24		29			
Heterogeneity: Chi² = 4.10, df = 6 (P = 0.66); I² = 0%						
Test for overall effect: Z = 0.79 (P = 0.43)						
Test for subgroup differences: Chi² = 1.18, df = 1 (P = 0.28), I² = 15.0%						

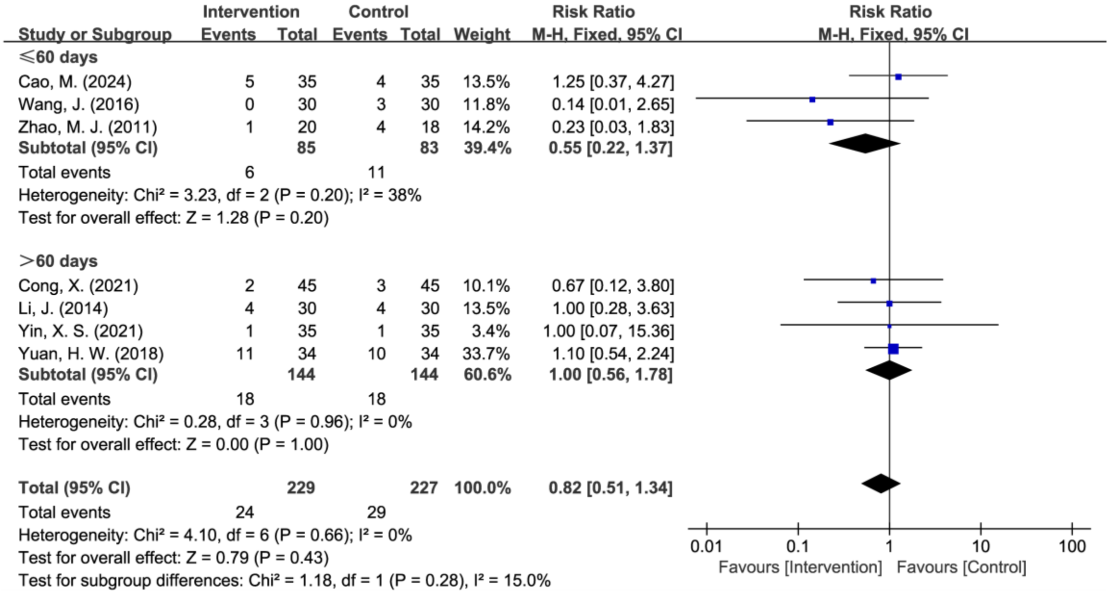

S10.4 60 days (Random Model)

Study or Subgroup	Intervention Events	Intervention Total	Control Events	Control Total	Weight	Risk Ratio M-H, Random, 95% CI
**≤60 days**						
Cao, M. (2024)	5	35	4	35	16.4%	1.25 [0.37, 4.27]
Wang, J. (2016)	0	30	3	30	2.9%	0.14 [0.01, 2.65]
Zhao, M. J. (2011)	1	20	4	18	5.6%	0.23 [0.03, 1.83]
**Subtotal (95% CI)**		**85**		**83**	**24.9%**	**0.50 [0.12, 2.09]**
Total events	6		11			
Heterogeneity: Tau² = 0.63; Chi² = 3.23, df = 2 (P = 0.20); I² = 38%						
Test for overall effect: Z = 0.95 (P = 0.34)						
**>60 days**						
Cong, X. (2021)	2	45	3	45	8.2%	0.67 [0.12, 3.80]
Li, J. (2014)	4	30	4	30	14.9%	1.00 [0.28, 3.63]
Yin, X. S. (2021)	1	35	1	35	3.3%	1.00 [0.07, 15.36]
Yuan, H. W. (2018)	11	34	10	34	48.7%	1.10 [0.54, 2.24]
**Subtotal (95% CI)**		**144**		**144**	**75.1%**	**1.02 [0.57, 1.81]**
Total events	18		18			
Heterogeneity: Tau² = 0.00; Chi² = 0.28, df = 3 (P = 0.96); I² = 0%						
Test for overall effect: Z = 0.06 (P = 0.95)						
**Total (95% CI)**		**229**		**227**	**100.0%**	**0.91 [0.56, 1.50]**
Total events	24		29			
Heterogeneity: Tau² = 0.00; Chi² = 4.10, df = 6 (P = 0.66); I² = 0%						
Test for overall effect: Z = 0.36 (P = 0.72)						
Test for subgroup differences: Chi² = 0.82, df = 1 (P = 0.37), I² = 0%						

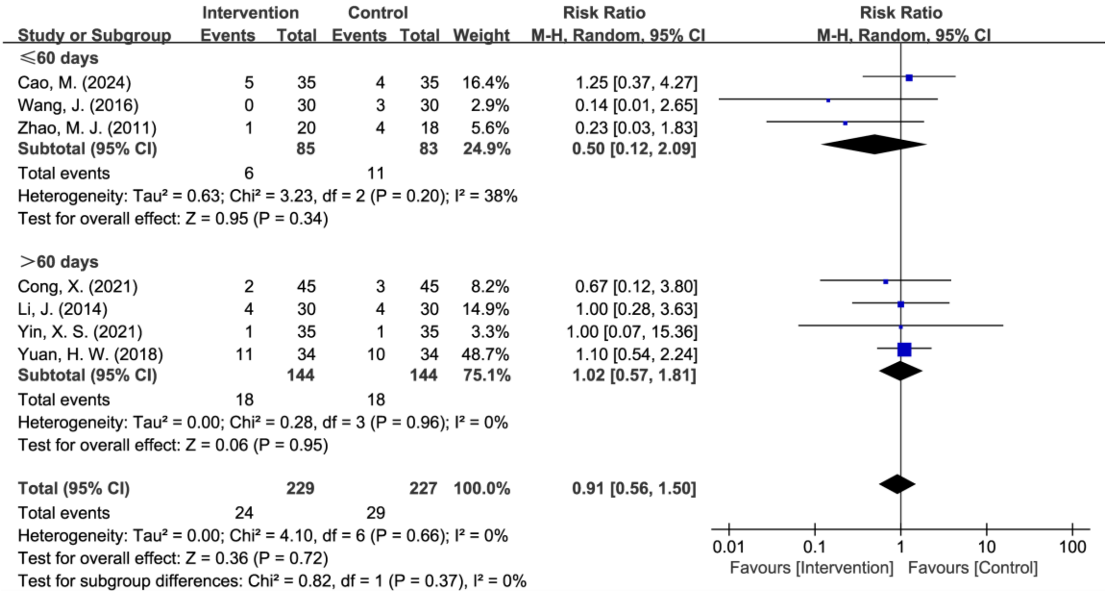

S10.5 Sensitivity Analysis of Heterogeneous Sources in ≤60 days

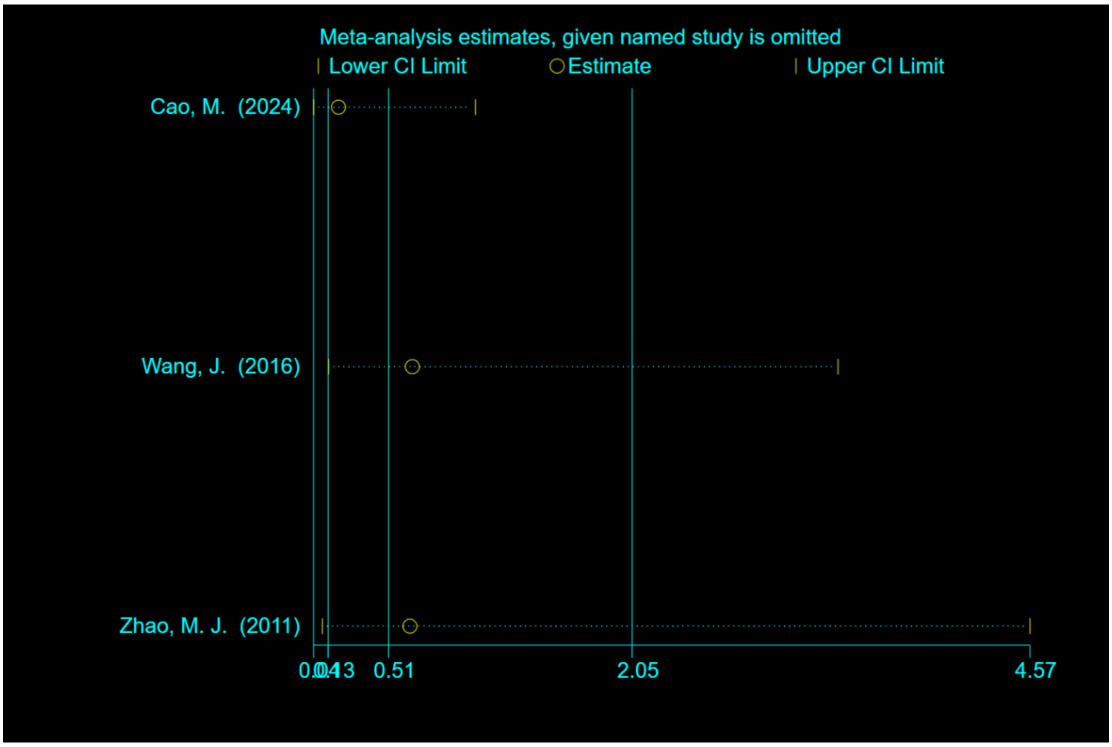

Study omitted	Estimate	[95% Conf.	Interval]
Cao, M. (2024)	.19279677	.03510283	1.0589057
Wang, J. (2016)	.66026878	.13005979	3.3519573
Zhao, M. J. (2011)	.64408618	.09085555	4.5660062
Combined	.50973921	.12675224	2.0499367

S10.6 Remaining 2 RCTs in the ≤60 days group (Exclude Cao)

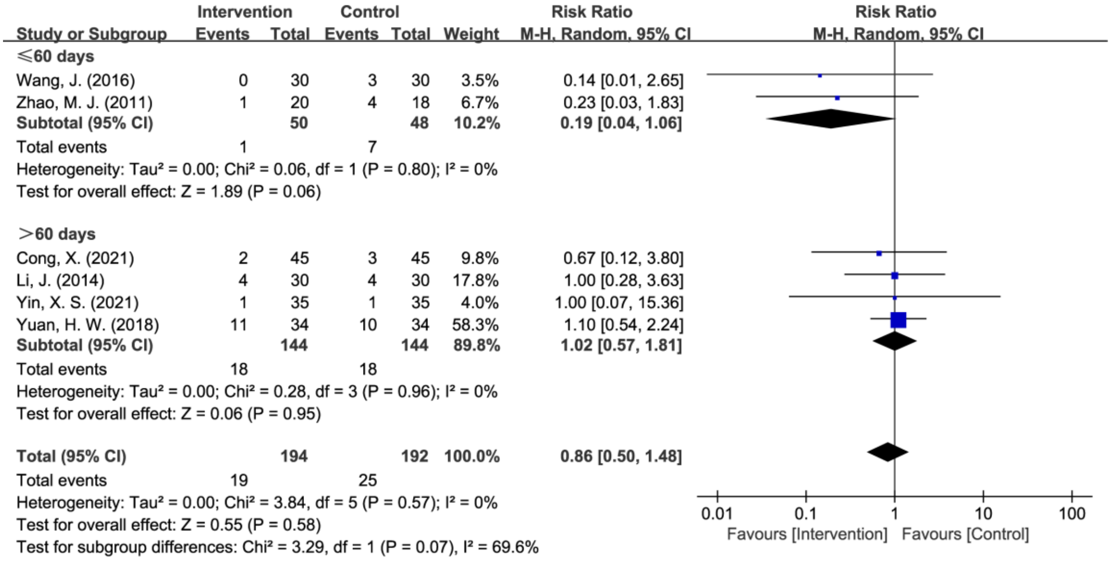

S10.7 30, 30-60, 60 days

Study or Subgroup	Intervention Events	Intervention Total	Control Events	Control Total	Weight	Risk Ratio M-H, Fixed, 95% CI
**≤30 days**						
Wang, J. (2016)	0	30	3	30	11.8%	0.14 [0.01, 2.65]
**Subtotal (95% CI)**		**30**		**30**	**11.8%**	**0.14 [0.01, 2.65]**
Total events	0		3			
Heterogeneity: Not applicable						
Test for overall effect: Z = 1.31 (P = 0.19)						
**30< days ≤60**						
Cao, M. (2024)	5	35	4	35	13.5%	1.25 [0.37, 4.27]
Zhao, M. J. (2011)	1	20	4	18	14.2%	0.23 [0.03, 1.83]
**Subtotal (95% CI)**		**55**		**53**	**27.6%**	**0.72 [0.27, 1.95]**
Total events	6		8			
Heterogeneity: Chi² = 1.95, df = 1 (P = 0.16); I² = 49%						
Test for overall effect: Z = 0.64 (P = 0.52)						
**>60 days**						
Cong, X. (2021)	2	45	3	45	10.1%	0.67 [0.12, 3.80]
Li, J. (2014)	4	30	4	30	13.5%	1.00 [0.28, 3.63]
Yin, X. S. (2021)	1	35	1	35	3.4%	1.00 [0.07, 15.36]
Yuan, H. W. (2018)	11	34	10	34	33.7%	1.10 [0.54, 2.24]
**Subtotal (95% CI)**		**144**		**144**	**60.6%**	**1.00 [0.56, 1.78]**
Total events	18		18			
Heterogeneity: Chi² = 0.28, df = 3 (P = 0.96); I² = 0%						
Test for overall effect: Z = 0.00 (P = 1.00)						
**Total (95% CI)**		**229**		**227**	**100.0%**	**0.82 [0.51, 1.34]**
Total events	24		29			
Heterogeneity: Chi² = 4.10, df = 6 (P = 0.66); I² = 0%						
Test for overall effect: Z = 0.79 (P = 0.43)						
Test for subgroup differences: Chi² = 1.82, df = 2 (P = 0.40), I² = 0%						

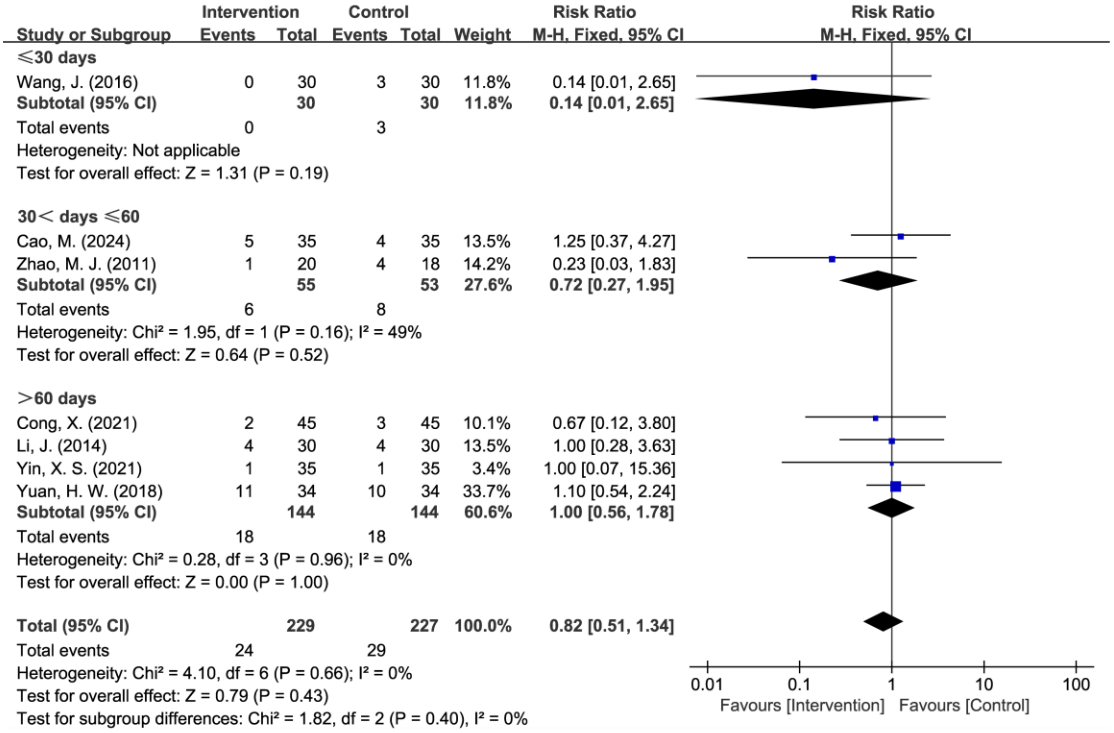

S10.8 30, 30-45, 45-60, 60 days

Study or Subgroup	Intervention Events	Intervention Total	Control Events	Control Total	Weight	Risk Ratio M-H, Fixed, 95% CI
**≤30 days**						
Wang, J. (2016)	0	30	3	30	11.8%	0.14 [0.01, 2.65]
**Subtotal (95% CI)**		**30**		**30**	**11.8%**	**0.14 [0.01, 2.65]**
Total events	0		3			
Heterogeneity: Not applicable						
Test for overall effect: Z = 1.31 (P = 0.19)						
**30< days ≤45**						
Zhao, M. J. (2011)	1	20	4	18	14.2%	0.23 [0.03, 1.83]
**Subtotal (95% CI)**		**20**		**18**	**14.2%**	**0.23 [0.03, 1.83]**
Total events	1		4			
Heterogeneity: Not applicable						
Test for overall effect: Z = 1.39 (P = 0.16)						
**45< days ≤60**						
Cao, M. (2024)	5	35	4	35	13.5%	1.25 [0.37, 4.27]
**Subtotal (95% CI)**		**35**		**35**	**13.5%**	**1.25 [0.37, 4.27]**
Total events	5		4			
Heterogeneity: Not applicable						
Test for overall effect: Z = 0.36 (P = 0.72)						
**>60 days**						
Cong, X. (2021)	2	45	3	45	10.1%	0.67 [0.12, 3.80]
Li, J. (2014)	4	30	4	30	13.5%	1.00 [0.28, 3.63]
Yin, X. S. (2021)	1	35	1	35	3.4%	1.00 [0.07, 15.36]
Yuan, H. W. (2018)	11	34	10	34	33.7%	1.10 [0.54, 2.24]
**Subtotal (95% CI)**		**144**		**144**	**60.6%**	**1.00 [0.56, 1.78]**
Total events	18		18			
Heterogeneity: Chi² = 0.28, df = 3 (P = 0.96); I² = 0%						
Test for overall effect: Z = 0.00 (P = 1.00)						
**Total (95% CI)**		**229**		**227**	**100.0%**	**0.82 [0.51, 1.34]**
Total events	24		29			
Heterogeneity: Chi² = 4.10, df = 6 (P = 0.66); I² = 0%						
Test for overall effect: Z = 0.79 (P = 0.43)						
Test for subgroup differences: Chi² = 3.61, df = 3 (P = 0.31), I² = 16.8%						

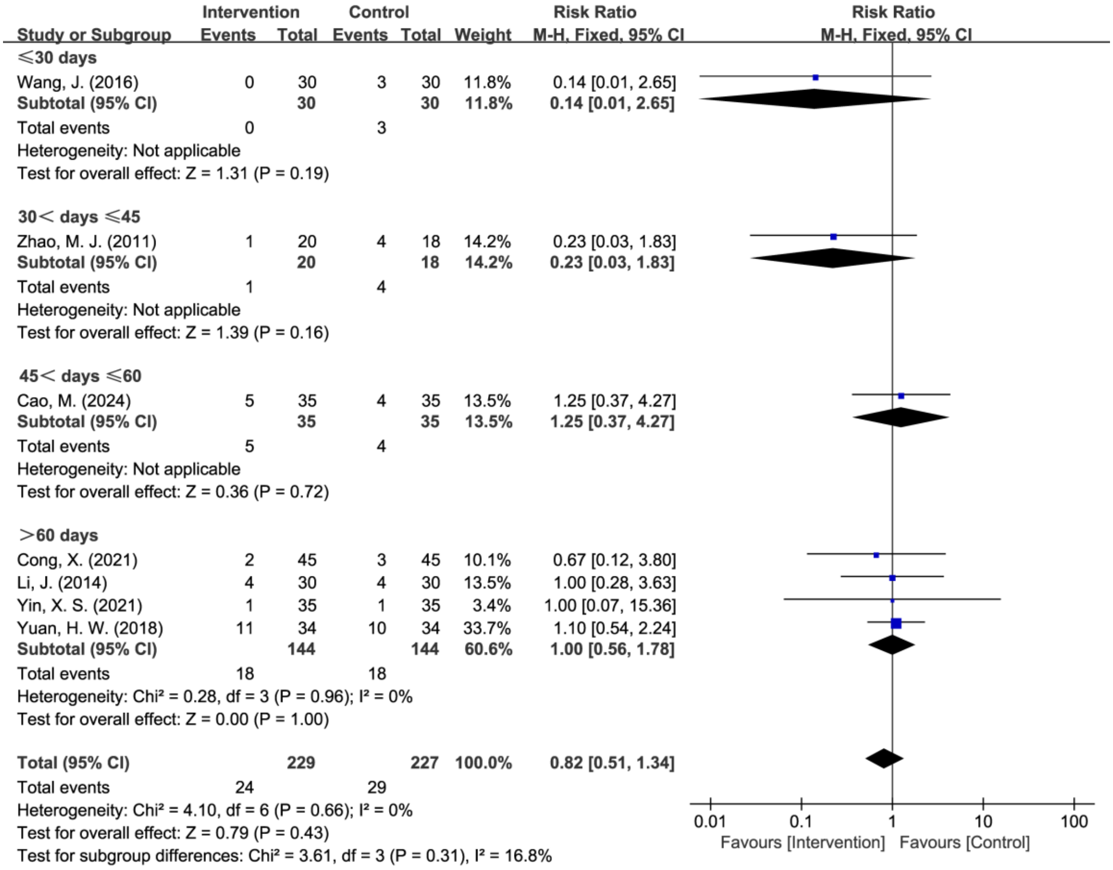

Supplement: Supplementary file 3 [file DataSheet3.pdf]
