# Supplementary material for: Efficacy and safety of Traditional Chinese Medicine in alleviating symptoms associated with myocardial bridge: a systematic review and meta-analysis
Source: Front Pharmacol. 2025 Sep 19;16:1619617. doi: 10.3389/fphar.2025.1619617 (PMC12492955; doi:10.3389/fphar.2025.1619617)
Supplement: Supplementary file 4 [file DataSheet1.pdf]

Detailed Search Strategy in Eight Databases

We searched eight databases [PubMed, Embase, Web of Science, Cochrane Library, China Biology Medicine Database (CBM), Wanfang Database, China Science and Technology Journal Database (VIP) and China National Knowledge Infrastructure (CNKI)] from database inception until April 1, 2025.

Page 1 to 2: Search strategy in PubMed.
Page 3: Search strategy in Embase.
Page 4: Search strategy in Web of Science.
Page 5: Search strategy in Cochrane Library.
Page 6: Search strategy in CBM.
Page 7: Search strategy in Wanfang.
Page 8: Search strategy in VIP.
Page 9 to 10: Search strategy in CNKI.
Page 11 to 13: Included literatures
Page 14 to 15: Excluded literatures

Supplementary Table S1. Search strategy in PubMed.

Search number	Query	Results
#1	"Myocardial Bridging"[Mesh] AND 1800/01/01:2025/04/01[dp]	510
#2	(Myocardial Bridging[Title/Abstract]) OR (Bridging, Myocardial[Title/Abstract]) OR (Bridgings, Myocardial[Title/Abstract]) OR (Myocardial Bridgings[Title/Abstract]) AND 1800/01/01:2025/04/01[dp]	3,585
#3	"Medicine, Chinese Traditional"[Mesh] AND 1800/01/01:2025/04/01[dp]	26,446
#4	(Zhong Yi Xue[Title/Abstract]) OR (Chung I Hsueh[Title/Abstract]) OR (Hsueh, Chung I[Title/Abstract]) OR (Traditional Medicine, Chinese[Title/Abstract]) OR (Chinese Traditional Medicine[Title/Abstract]) OR (Traditional Chinese Medicine[Title/Abstract]) OR (Chinese Medicine, Traditional[Title/Abstract]) OR (Traditional Tongue Diagnosis[Title/Abstract]) OR (Tongue Diagnoses, Traditional[Title/Abstract]) OR (Tongue Diagnosis, Traditional[Title/Abstract]) OR (Traditional Tongue Diagnoses[Title/Abstract]) OR (Traditional Tongue Assessment[Title/Abstract]) OR (Tongue Assessment, Traditional[Title/Abstract]) OR (Traditional Tongue Assessments[Title/Abstract]) AND 1800/01/01:2025/04/01[dp]	41,350
#5	#1 OR #2	3,615
#6	#3 OR #4	55,533
#7	#5 AND #6	3

History and Search Details

Download Delete

Search	Actions	Details	Query	Results	Time
#7	•••	>	Search: **#5 AND #6**	3	22:14:32
#6	•••	>	Search: **#3 OR #4**	55,533	22:14:21
#5	•••	>	Search: **#1 OR #2**	3,615	22:14:10
#4	•••	>	Search: **(Zhong Yi Xue[Title/Abstract]) OR (Chung I Hsueh[Title/Abstract]) OR (Hsueh, Chung I[Title/Abstract]) OR (Traditional Medicine, Chinese[Title/Abstract]) OR (Chinese Traditional Medicine[Title/Abstract]) OR (Traditional Chinese Medicine[Title/Abstract]) OR (Chinese Medicine, Traditional[Title/Abstract]) OR (Traditional Tongue Diagnosis[Title/Abstract]) OR (Tongue Diagnoses, Traditional[Title/Abstract]) OR (Tongue Diagnosis, Traditional[Title/Abstract]) OR (Traditional Tongue Diagnoses[Title/Abstract]) OR (Traditional Tongue Assessment[Title/Abstract]) OR (Tongue Assessment, Traditional[Title/Abstract]) OR (Traditional Tongue Assessments[Title/Abstract]) AND 1800/01/01:2025/04/01[dp]**	41,350	22:12:53
#3	•••	>	Search: **"Medicine, Chinese Traditional"[Mesh] AND 1800/01/01:2025/04/01[dp]**	26,446	22:12:40
#2	•••	>	Search: **(Myocardial Bridging[Title/Abstract]) OR (Bridging, Myocardial[Title/Abstract]) OR (Bridgings, Myocardial[Title/Abstract]) OR (Myocardial Bridgings[Title/Abstract]) AND 1800/01/01:2025/04/01[dp]**	3,586	22:12:31
#1	•••	>	Search: **"Myocardial Bridging"[Mesh] AND 1800/01/01:2025/04/01[dp]**	510	22:12:22

Showing 1 to 7 of 7 entries

Supplementary Table S2. Search strategy in Embase.

No.	Query	Results
#7	#3 AND #6	2
#6	#4 OR #5	110701
#5	'zhong yi xue':ab,ti OR 'chung i hsueh':ab,ti OR 'hsueh, chung i':ab,ti OR 'traditional medicine, chinese':ab,ti OR 'chinese traditional medicine':ab,ti OR 'traditional chinese medicine':ab,ti OR 'chinese medicine, traditional':ab,ti OR 'traditional tongue diagnosis':ab,ti OR 'tongue diagnoses, traditional':ab,ti OR 'tongue diagnosis, traditional':ab,ti OR 'traditional tongue diagnoses':ab,ti OR 'traditional tongue assessment':ab,ti OR 'tongue assessment, traditional':ab,ti OR 'traditional tongue assessments':ab,ti	54067
#4	'chinese medicine'/exp	92621
#3	#1 OR #2	2079
#2	'bridging, myocardial':ab,ti OR 'bridgings, myocardial':ab,ti OR 'myocardial bridgings':ab,ti	11
#1	'myocardial bridging'/exp	2073

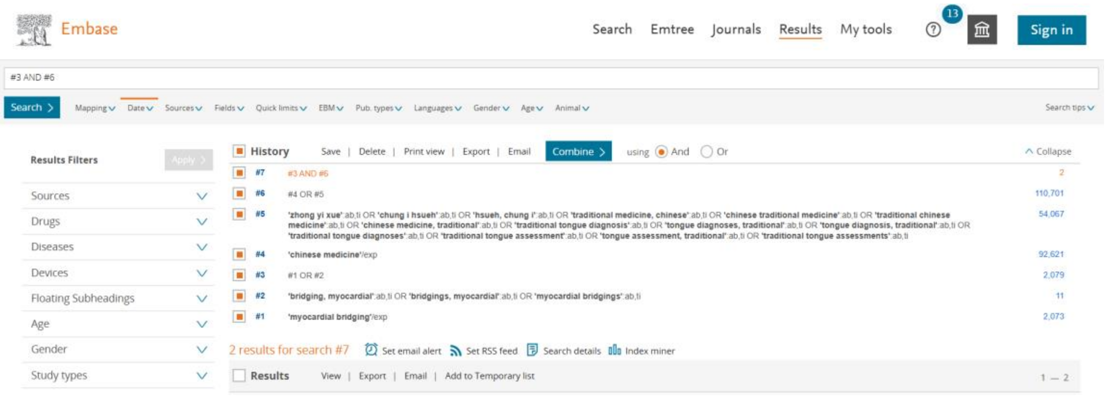

Supplementary Table S3. Search strategy in Web of Science.

#	Search Query	Results
#1	(((TS=("Myocardial Bridging")) OR TS=("Bridging, Myocardial")) OR TS=("Bridgings, Myocardial")) OR TS=("Myocardial Bridgings") and Preprint Citation Index (Exclude – Database)	1443
#2	((((((((((((TS=("Medicine, Chinese Traditional")) OR TS=("Zhong Yi Xue")) OR TS=("Chung I Hsueh")) OR TS=("VHsueh, Chung I")) OR TS=("Traditional Medicine, Chinese")) OR TS=("Chinese Traditional Medicine")) OR TS=("Traditional Chinese Medicine")) OR TS=("Chinese Medicine, Traditional")) OR TS=("Traditional Tongue Diagnosis")) OR TS=("Tongue Diagnoses, Traditional")) OR TS=("Tongue Diagnosis, Traditional")) OR TS=("Traditional Tongue Diagnoses")) OR TS=("Traditional Tongue Assessment")) OR TS=("Tongue Assessment, Traditional")) OR TS=("Traditional Tongue Assessments") and Preprint Citation Index (Exclude – Database)	103393
#3	#1 AND #2 and Preprint Citation Index (Exclude – Database)	2

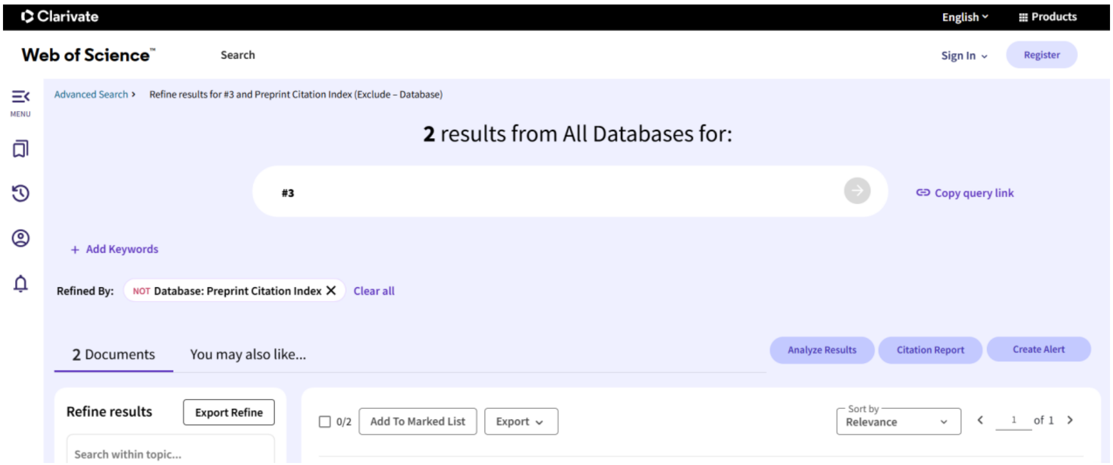

Supplementary Table S4. Search strategy in Cochrane Library.

No.	Query	Results
#1	MeSH descriptor: [Myocardial Bridging] explode all trees	6
#2	((Bridging, Myocardial) OR (Bridgings, Myocardial) OR (Myocardial Bridgings)):ti,ab,kw	58
#3	#1 OR #2	58
#4	MeSH descriptor: [Medicine, Chinese Traditional] explode all trees	1830
#5	(Zhong Yi Xue) OR (Chung I Hsueh) OR (Hsueh, Chung I) OR (Traditional Medicine, Chinese) OR (Chinese Traditional Medicine) OR (Traditional Chinese Medicine) OR (Chinese Medicine, Traditional) OR (Traditional Tongue Diagnosis) OR (Tongue Diagnoses, Traditional) OR (Tongue Diagnosis, Traditional) OR (Traditional Tongue Diagnoses) OR (Traditional Tongue Assessment) OR (Tongue Assessment, Traditional) OR (Traditional Tongue Assessments)	28382
#6	#4 OR #5	28601
#7	#3 AND #6	0

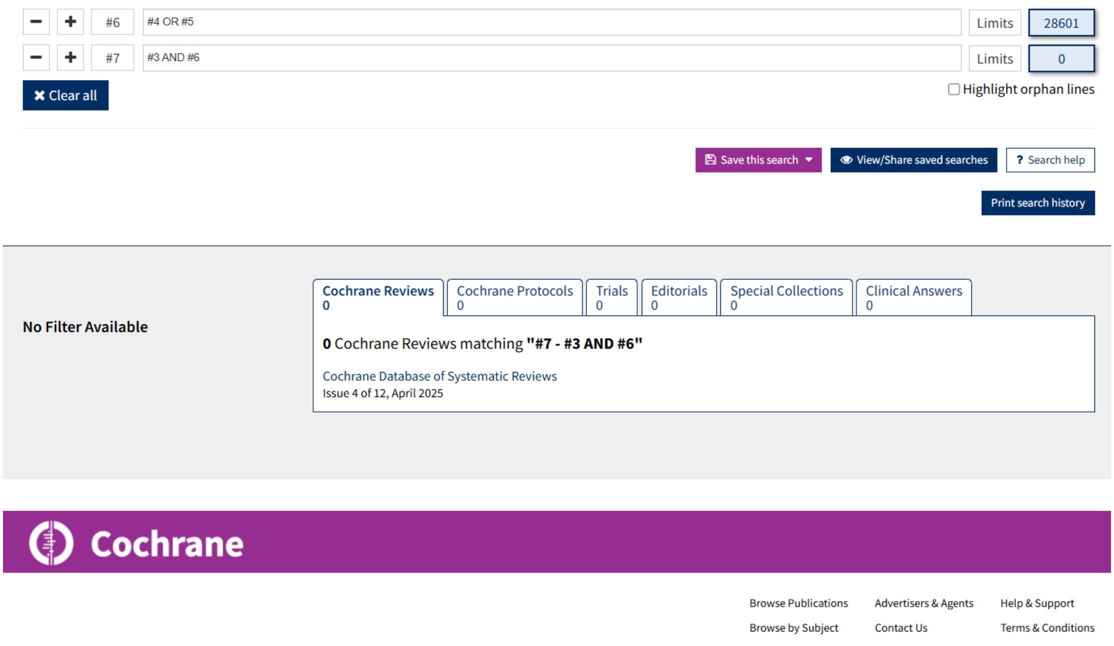

Supplementary Search strategy in CBM (English)

Search formula: ("Traditional Chinese Medicine" [common field:intelligent] OR "Chinese materia medica" [common field:intelligent] OR "Traditional Chinese Medicine and Chinese materia medica" [common field:intelligent] OR "capsule" [common field:intelligent] OR "tablet" [common field:intelligent] OR "pill" [common field:intelligent] OR "powder" [common field:intelligent] OR "Self-formulated" [common field:intelligent] OR "preparation" [common field:intelligent] OR "granule" [common field:intelligent]) AND ("Myocardial bridging" [common field:intelligent] OR "Coronary artery myocardial bridging" [common field:intelligent] OR "Coronary myocardial bridging" [common field:intelligent])

Results = 273

Supplementary Search strategy in CBM (Chinese)

检索式：("中医"[常用字段:智能] OR "中药"[常用字段:智能] OR "中医药"[常用字段:智能] OR "胶囊"[常用字段:智能] OR "片"[常用字段:智能] OR "丸"[常用字段:智能] OR "散"[常用字段:智能] OR "自拟"[常用字段:智能] OR "剂"[常用字段:智能] OR "颗粒"[常用字段:智能]) AND("心肌桥"[常用字段:智能] OR "冠状动脉心肌桥"[常用字段:智能] OR "冠状动脉肌桥"[常用字段:智能])

结果 = 273

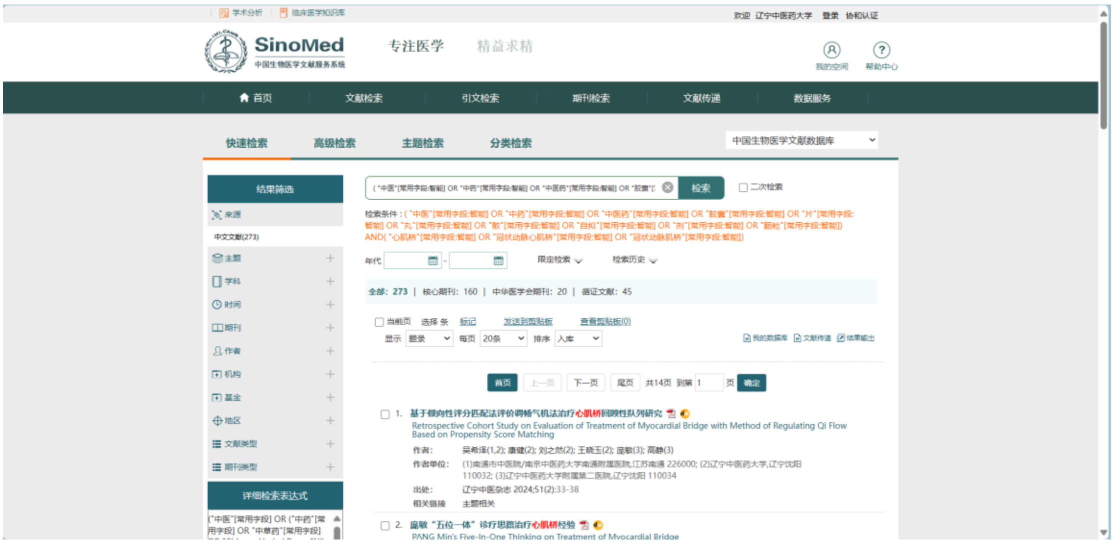

Supplementary Table S2. Search strategy in WanFang (English)

Search formula: ((theme:(Traditional Chinese Medicine OR Chinese materia medica OR Traditional Chinese Medicine and Chinese materia medica OR capsule OR tablet OR pill OR powder OR Self-formulated OR preparation OR granule)) AND (published-time:*-2025)) AND ((theme:(Myocardial bridging OR Coronary artery myocardial bridging OR Coronary myocardial bridging)) AND (published time:*-2025))

Results = 362

Supplementary Table S3. Search strategy in WanFang (Chinese)

检索式：((主题:(中医 OR 中药 OR 中医药 OR 胶囊 OR 片 OR 丸 OR 散 OR 自拟 OR 剂 OR 颗粒)) and 发表时间:*-2025) AND ((主题:(心肌桥 OR 冠状动脉心肌桥 OR 冠状动脉肌桥)) and 发表时间:*-2025)

结果 = 362

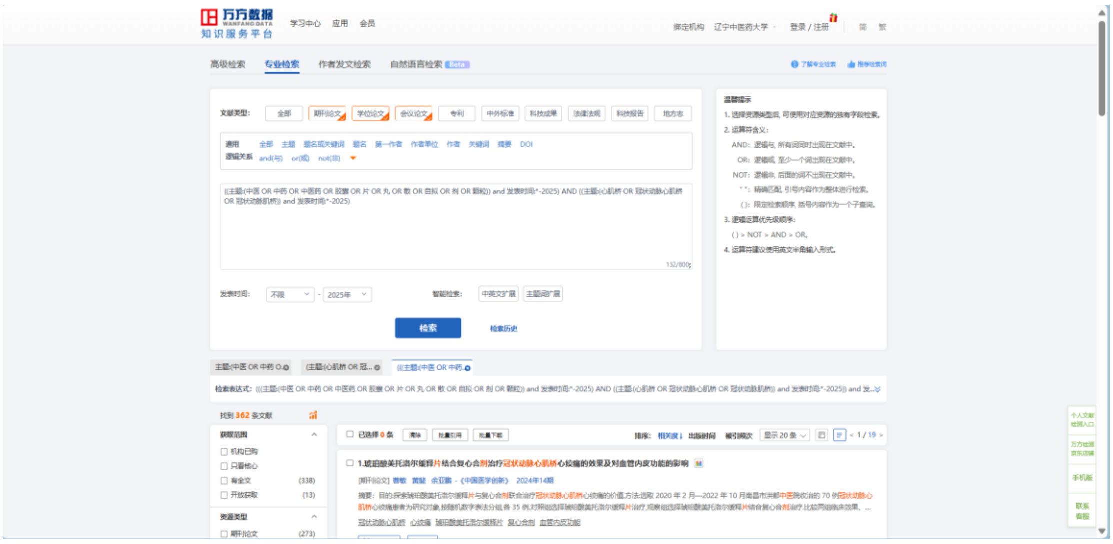

Supplementary Search strategy in VIP (English)

Search formula: (M=(Traditional Chinese Medicine OR Chinese materia medica OR Traditional Chinese Medicine and Chinese materia medica OR capsule OR tablet OR pill OR powder OR Self-formulated OR preparation OR granule) OR R=(Traditional Chinese Medicine OR Chinese materia medica OR Traditional Chinese Medicine and Chinese materia medica OR capsule OR tablet OR pill OR powder OR Self-formulated OR preparation OR granule)) AND (M=(Myocardial bridging OR Coronary artery myocardial bridging OR Coronary myocardial bridging) or R=(Myocardial bridging OR Coronary artery myocardial bridging OR Coronary myocardial bridging))

Results = 193

Supplementary Search strategy in VIP (Chinese)

检索式：(M=(中医 OR 中药 OR 中医药 OR 胶囊 OR 片 OR 丸 OR 散 OR 自拟 OR 剂 OR 颗粒) OR R=(中医 OR 中药 OR 中医药 OR 胶囊 OR 片 OR 丸 OR 散 OR 自拟 OR 剂 OR 颗粒)) AND (M=(心肌桥 OR 冠状动脉心肌桥 OR 冠状动脉肌桥) or R=(心肌桥 OR 冠状动脉心肌桥 OR 冠状动脉肌桥))

结果 = 193

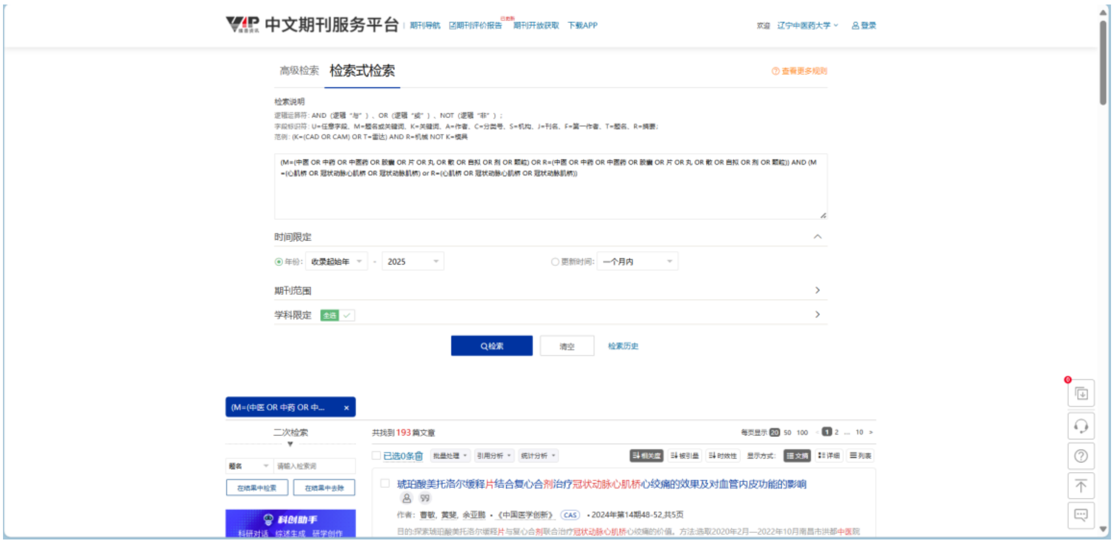

Supplementary Table S5. Search strategy in CNKI (English)

Search number	Query	Results
#1	Traditional Chinese Medicine + Chinese materia medica + Traditional Chinese Medicine and Chinese materia medica + capsule + tablet + pill + powder + Self-formulated + preparation + granule	3,515,514
#2	Myocardial bridging + Coronary artery myocardial bridging + Coronary myocardial bridging	2045
#3	1 * 2	115

Supplementary Table S6. Search strategy in CNKI (Chinese)

Search number	Query	Results
#1	中医 + 中药 + 中医药 + 胶囊 + 片 + 丸 + 散 + 自拟 + 剂 + 颗粒	3,515,514
#2	心肌桥 + 冠状动脉心肌桥 + 冠状动脉肌桥	2045
#3	1 * 2	115

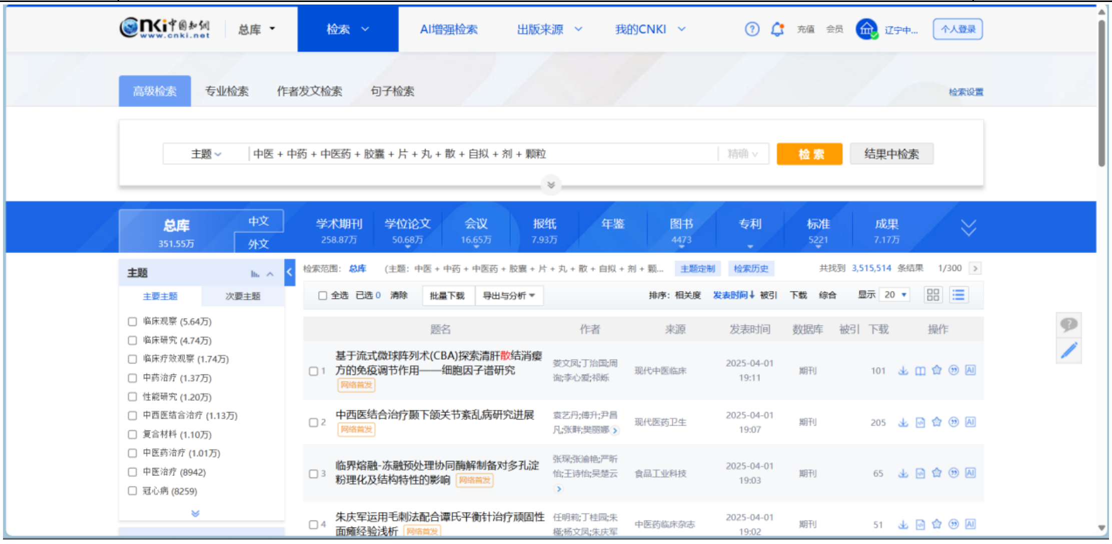

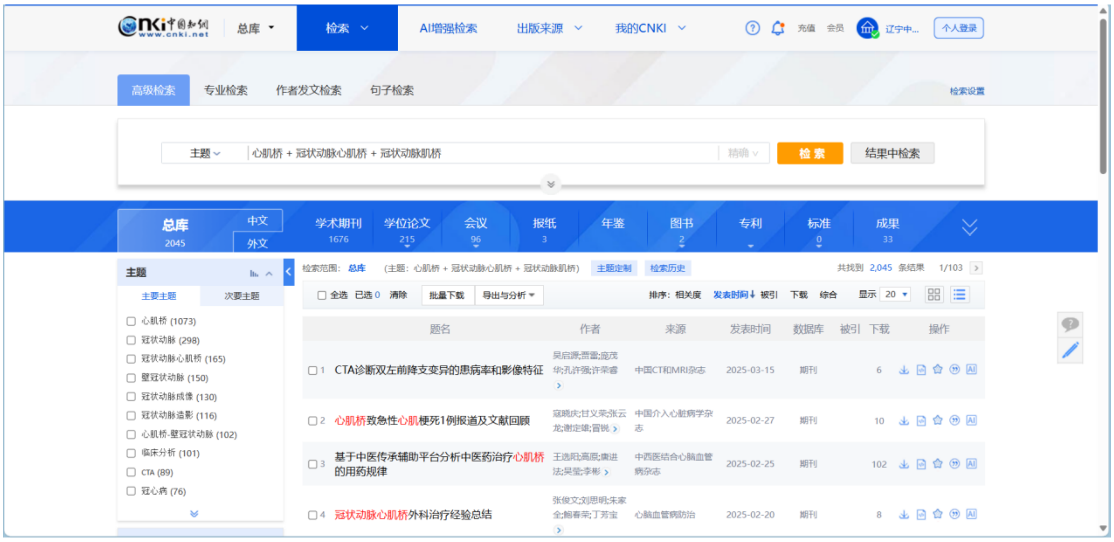

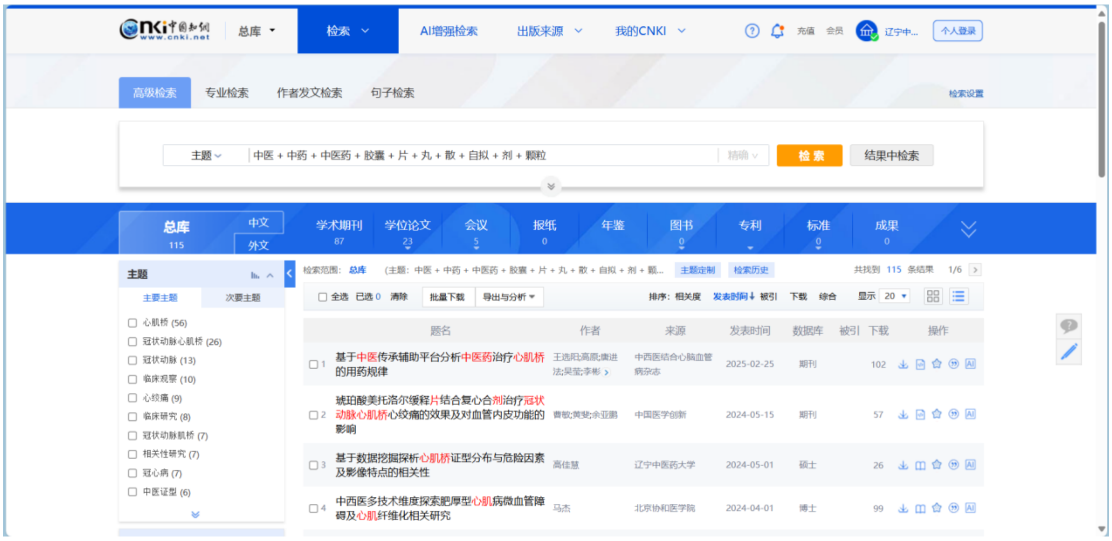
总库
检索
AI增强检索
出版来源
我的CNKI
充值
会员
个人登录
高级检索
专业检索
作者发文检索
句子检索
检索设置
主题
中医 + 中药 + 中医药 + 胶囊 + 片 + 丸 + 散 + 自拟 + 剂 + 颗粒
检 索
结果中检索
总库 115
中文
外文
学术期刊 87
学位论文 23
会议 5
报纸 0
年鉴
图书 0
专利
标准 0
成果 0
主题
主要主题
次要主题
心肌桥 (56)
冠状动脉心肌桥 (26)
冠状动脉 (13)
临床观察 (10)
心绞痛 (9)
临床研究 (8)
冠状动脉肌桥 (7)
相关性研究 (7)
冠心病 (7)
中医证型 (6)
主题定制
检索历史
共找到 115 条结果
1/6
全选
已选 0
清除
批量下载
导出与分析
排序：相关度
发表时间
被引
下载
综合
显示 20
题名
作者
来源
发表时间
数据库
被引
下载
操作
基于中医传承辅助平台分析中医药治疗心肌桥的用药规律
2025-02-25
期刊
102
琥珀酸美托洛尔缓释片结合复心合剂治疗冠状动脉心肌桥心绞痛的效果及对血管内皮功能的影响
中国医学创新
2024-05-15
期刊
57
基于数据挖掘探析心肌桥证型分布与危险因素及影像特点的相关性
高佳慧
辽宁中医药大学
2024-05-01
硕士
26
中西医多技术维度探索肥厚型心肌病微血管障碍及心肌纤维化相关研究
马杰
北京协和医学院
2024-04-01
博士
99

Included literatures

[1]曹敏,黄斐,余亚鹏.琥珀酸美托洛尔缓释片结合复心合剂治疗冠状动脉心肌桥心绞痛的效果及对血管内皮功能的影响[J].中国医学创新,2024,21(14):48-52. DOI:10.3969/j.issn.1674-4985.2024.14.012

[1]Cao M, Huang F, Yu YP. The Effect of Metoprolol Succinate Sustained-release Tablets Combined with Fuxin Mixture in the Treatment of Coronary Artery Myocardial Bridging Angina and Its Impact on Vascular Endothelial Function. *Med Innov China*. 2024;21(14):48-52. DOI:10.3969/j.issn.1674-4985.2024.14.012

[2]陈军,陈联发,许娜.自拟理气活血通络方治疗气滞血瘀型冠状动脉心肌桥的临床研究[J].心血管病防治知识,2017,(8):50-52. DOI:10.3969/j.issn.1672-3015(x).2017.08.019

[2]Chen J, Chen LF, Xu N. Clinical Observation of Self-prepared Liqi Huoxue Tongluo Formula in the Treatment of Qi Stagnation and Blood Stasis Type Coronary Artery Myocardial Bridge. *Prev Treat Cardiovasc Dis*. 2017;(8):50-52. DOI:10.3969/j.issn.1672-3015(x).2017.08.019

[3]丛晓,王莉,粟全球,等. 比索洛尔联合活血安神解郁方对冠状动脉左前降支心肌桥合并焦虑和抑郁患者平板运动试验及不良情绪的影响[J]. 现代中西医结合杂志,2021,30(17):1867-1870+1919. DOI:10.3969/j.issn.1008-8849.2021.17.010.

[3]Cong X, Wang L, Su Q, Q., Mu RN, Li L. Effect of bisoprolol combined with Huoxue Anshen Jieyu Decoction on treadmill exercise test and unhealthy emotion in patients with left anterior descending coronary artery myocardial bridge combined with anxiety and depression. *Mod J Integr Tradit West Med*. 2021;30(17):1867-1870+1919. DOI:10.3969/j.issn.1008-8849.2021.17.010.

[4]樊光辉,何亚雄,邹佳妮,等.麝香保心丸联合美托洛尔治疗冠状动脉心肌桥疗效观察[J].中西医结合心脑血管病杂志,2012,10(1):3-4. DOI:10.3969/j.issn.1672-1349.2012.01.002

[4]Fan GH, He YX, Zou JN, Chen XJ. Clinical Observation of Shexiang Baoxin Pill and Metoprolol for Treatment of Coronary Myocardial Bridge. *Chin J Integr Med Cardio Cerebrovasc Dis*. 2012;10(1):3-4. DOI:10.3969/j.issn.1672-1349.2012.01.002

[5]韩垚,戴梅,刘红旭,等. 益气通脉汤治疗孤立性冠状动脉肌桥心绞痛气虚血瘀证临床研究[J]. 国际中医中药杂志,2022,44(1):22-27. DOI:10.3760/cma.j.cn115398-20210825-00299.

[5]Han Y, Dai M, Liu HX, Zhang DW, Wei ZZ. Clinical study of Yiqi-Tongmai Decoction on qi deficiency and blood stasis syndrome of isolated coronary artery muscle bridge angina pectoris. *Int J Tradit Chin Med*. 2022;44(1):22-27. DOI:10.3760/cma.j.cn115398-20210825-00299

[6]李建.心可舒片联合美托洛尔治疗冠状动脉心肌桥的临床观察[J].中西医结合心脑血管病杂志,2014,12(10):1185-1186. DOI:10.3969/j.issn.16721349.2014.10.010

[6]Li J. Clinical Observation of Xinkeshu Tablets and Metoprolol in the Treatment of Coronary Artery Myocardial Bridge. *Chin J Integr Med Cardio Cerebrovasc Dis*. 2014;12(10):1185-1186. DOI:10.3969/j.issn.16721349.2014.10.010

[7]乔建峰,郑峰,李翠云,等.益气畅脉饮加减治疗气虚血瘀型心肌桥的临床观察[J].心血管病防治知识,2021,11(26):16-18. DOI:10.3969/j.issn.1672-3015(x).2021.26.005

[7]Qiao JF, Zheng F, Li CY, Lin C, Wu X. Clinical Observation of Modified Yiqi Changmai Yin in the Treatment of Deficiency of Qi and Blood Stasis Type Coronary Artery Myocardial Bridge. *Prev Treat Cardiovasc Dis*. 2021;11(26):16-18. DOI:10.3969/j.issn.1672-3015(x).2021.26.005

[8]王建刚,席建堂,赵小奎,等.脑心通胶囊治疗有症状冠状动脉肌桥合并颈动脉硬化的疗效观察及

预后分析[J].临床医药文献电子杂志,2016,3(21):4302-4304. DOI:10.16281/j.cnki.jocml.2016.21.123
[8]Wang JG, Xi JT, Zhao XK, et al. Efficacy Observation and Prognostic Analysis of Naoxintong Capsules in the Treatment of Symptomatic Coronary Artery Myocardial Bridges Combined with Carotid Atherosclerosis. *Electron J Clin Med Lit*. 2016;3(21):4302-4304. DOI:10.16281/j.cnki.jocml.2016.21.123
[9]王娟,薛军民,吕兴旺,等.水蛭、苦参粉治疗冠状动脉肌桥致心绞痛 60 例临床观察[J].中国中医药科技,2016,23(4):492-493.
[9]Wang J, Xue JM, Lv XW, Zhang XL, Wang LR, Chen JS. Clinical Observation of Leech and Kushen Powder Capsules in the Treatment of 60 Cases of Patients with Angina Pectoris Due to Coronary Artery Myocardial Bridge. *Chin J Tradit Med Sci Technol*. 2016;23(4):492-493.
[10]王志新,韩燕. 柴胡疏肝散加减治疗气滞血瘀型冠状动脉心肌桥的效果评价[J]. 健康必读,2020(26):68.
[10]Wang ZX, Han Y. Evaluation of the Effect of Modified Chaihu Shuohe Powder in the Treatment of Qi Stagnation and Blood Stasis Type Coronary Artery Myocardial Bridge. *Healthmust-Read Mag*. 2020;(26):68.
[11]尹晓姝,郑经纬,张永红,等.宽胸气雾剂治疗冠状动脉肌桥患者的临床研究[J].吉林医学,2021,42(7):1569-1571. DOI:10.3969/j.issn.1004-0412.2021.07.009
[11]Yin XS, Zheng JW, Zhang YH, et al. Clinical study of wide chest aerosol in the treatment of patients with coronary artery muscle bridge. *Jilin Med J*. 2021;42(7):1569-1571. DOI:10.3969/j.issn.1004-0412.2021.07.009
[12]袁洪文,王新莉,李朕,等.三参三七蛭琥颗粒联合比索洛尔治疗冠状动脉心肌桥的临床研究[J].中西医结合心脑血管病杂志,2018,16(6):684-686. DOI:10.3969/j.issn.1672-1349.2018.06.003
[12]Yuan HW, Wang XL, Li Z, Yang JY, Yuan JQ. Clinical Observation on Sanshen Sanqi Zhihu Granule and Bisoprolol in the Treatment of Myocardial Bridge. *Chin J Integr Med Cardio Cerebrovasc Dis*. 2018;16(6):684-686. DOI:10.3969/j.issn.1672-1349.2018.06.003
[13]张娟,武佶,魏希进,等.复心合剂联合琥珀酸美托洛尔缓释片治疗冠状动脉肌桥心绞痛患者临床观察[J].疑难病杂志,2023,22(3):253-257+271. DOI:10.3969/j.issn.1671-6450.2023.03.006
[13]Zhang J, Wu J, Wei XJ, Yang SW. Clinical observation of Fuxin Heji Decoction combined with metoprolol succinate sustained-release tablets in the treatment of coronary artery myobridge angina. *Chin J Diffic Complic Cases*. 2023;22(3):253-257+271. DOI:10.3969/j.issn.1671-6450.2023.03.006
[14]张萍,张广金,张虹.芪参益气滴丸治疗心肌桥伴心绞痛患者的有效性研究[J].天津中医药,2016,33(4):208-212. DOI:10.11656/j.issn.1672-1519.2016.04.05
[14]Zhang P, Zhang GJ, Zhang H. Efficacy study for Qishen Yiqi dropping pills in patients with myocardial bridge and angina pectoris. *Tianjin J Tradit Chin Med*. 2016;33(4):208-212. DOI:10.11656/j.issn.1672-1519.2016.04.05
[15]赵明君,闫维力,赵阳.通心络胶囊治疗冠状动脉心肌桥的临床观察[J].陕西中医,2011,32(6):645+658. DOI:10.3969/j.issn.1000-7369.2011.06.002
[15]Zhao MJ, Yan WL, Zhao Y. Coronary Myocardial Bridge Massage Treated by Tongxinluo Capsule. *Shaanxi J Tradit Chin Med*. 2011;32(6):645+658. DOI:10.3969/j.issn.1000-7369.2011.06.002
[16]郑永宏,谢建农,林松,等."顺气通脉胶囊"治疗冠状动脉心肌桥患者心绞痛 40 例临床研究[J].江苏中医药,2012,44(9):13-14. DOI: 10.3969/j.issn.1672-397X.2012.09.008
[16]Zheng YH, Xie JN, Lin S, Zhang W, Chen ZG, Zhou JR. Clinical Observation of "Shunqi

Tongxin Capsule" in the Treatment of 40 Cases of Patients with Angina Pectoris Due to Coronary Artery Myocardial Bridge. *Jiangsu J Tradit Chin Med*. 2012;44(9):13-14. DOI: 10.3969/j.issn.1672-397X.2012.09.008

[17]钟庆扬,郭进建.从肝论治气滞血瘀型心肌桥的临床观察[J].光明中医,2019,34(13):1967-1970. DOI:10.3969/j.issn.1003-8914.2019.13.012

[17]Zhong QY, Guo JJ. Clinical Observation on Treatment of Qi Stagnation and Blood Stasis Type Myocardial Bridge from Liver. *Guangming J Chin Med*. 2019;34(13):1967-1970. DOI:10.3969/j.issn.1003-8914.2019.13.012

[18]朱根源.营心宁胶囊联合美托洛尔治疗冠状动脉心肌桥疗效观察[J].湖北中医杂志,2014,36(5):3-4.

[18]Zhu GY. Clinical Observation of Yingxinning Capsule and Metoprolol in Treatment of Coronary Myocardial Bridge. *Hubei J Tradit Chin Med*. 2014;36(5):3-4.

Excluded literatures

① master's theses (*n*=5)

[1]陈萍萍.柴胡疏肝散加减治疗气滞血瘀型冠状动脉心肌桥的疗效观察[D].福建中医药大学,2016.

[1]Chen PP. Effects of Chai Hu Shu Gan San on Coronary Myocardial Bridge of Qi Stagnation and Blood Stasis Type. Fujian University of Traditional Chinese Medicine, 2016.

[2]陈赛赛.柴胡疏肝散合丹参饮治疗气滞血瘀型冠状动脉心肌桥的临床研究[D].上海中医药大学,2019. DOI:10.27320/d.cnki.gszyu.2019.000425.

[2]Chen, SS. Clinical Study of Chaihu Shugan San and Danshen Yin in the Treatment of Coronary Artery Myocardial Bridge of Qi Stagnation and Blood Stasis Type. Shanghai University of Traditional Chinese Medicine, 2019.

[3]徐亦男.心肌桥患者发生心绞痛与肌桥指数的关系及天香丹的干预研究[D].新疆医科大学,2019.

[3]Xu YN. Relationship between angina pectoris and muscle bridge index in patients with myocardial bridge and intervention study of Tianxiang Dan. Xinjiang Medical University. 2019.

[4]杨佳瑞.益气活血法治疗心肌桥患者的临床疗效观察[D].黑龙江中医药大学,2014.

[4]Yang JR. To observe the clinical therapeutic effect of supplementing qi and activating blood circulation method in treatment of patients with myocardial bridge. Heilongjiang University of Chinese Medicine,2014.

[5]张派.养心氏片治疗冠状动脉肌桥的疗效观察[D].天津中医药大学,2017.

[5]Zhang P. Treatment effects of Yangxin tablet on coronary artery bridge. Tianjin University of Chinese Medicine,2014.

② non-RCTs (*n*=2)

[1]陶学良,裴冰洁,牛天福.牛天福自拟八味通络化瘀汤治疗气虚血瘀型心肌桥探析[J].世界最新医学信息文摘,2018,18(55):200+202. DOI:10.19613/j.cnki.1671-3141.2018.55.101.

[1]Tao XL. Pei BJ. Niu TF. Exploration of Niu Tianfu's Self-prepared Eight-flavored Tongluo Huayu Tang for the treatment of myocardial bridges of qi deficiency and blood stasis type. World Latest Med Inf.2018;18(55):200+202. DOI:10.19613/j.cnki.1671-3141.2018.55.101.

[2]林小端.丹栀逍遥散治疗气滞型心肌桥胸痹 30 例临床观察[J].福建中医药大学学报,2013,23(6):53-55. DOI:10.13261/j.cnki.jfutcm.003060.

[2]Lin XD. Clinical observation on 30 cases of chest paralysis of qi stagnation type myocardial bridge treated by Danzhi Xiaoyao San. Rehabil Med. 2013;23(6):53-55. DOI:10.13261/j.cnki.jfutcm.003060.

③ insufficient sample size (<30 participants, *n*=1)

[1]何晓.通心络胶囊联合倍他乐克治疗冠状动脉心肌桥临床观察[J].临床合理用药杂志,2012,5(20):7-8. DOI:10.15887/j.cnki.13-1389/r.2012.20.065.

[1]He X. The clinical observation of Tongxinluo Capsule combined with metoprolol in the treatment of coronary myocardial bridge. Chin J Clin Rational Drug Use. 2012;5(20):7-8. DOI:10.15887/j.cnki.13-1389/r.2012.20.065.

④ mismatched study content (*n*=1)

[1]杜秀娟,李旭文.比索洛尔与通心络胶囊联合分时段给药治疗单纯不完全心肌桥临床观察[J].临床医学,2014,34(8):4-6.

[1]Du XJ, Li XW. Clinical observation of combined drug treatment of bisoprolol and Tongxinl

uo capsule at different period of time for in-complete myocardial bridge. Clin Med. 2014;34(8): 4-6.

⑤ Other outcome indicators (*n*=1)

[1]王铭,李瑛,蒋嘉辉,等.天王补心丹对心肌桥患者躯体化症状及焦虑、抑郁的影响[J].中国民间疗法,2021,29(11):62-66. DOI:10.19621/j.cnki.11-3555/r.2021.1124.

[1]Wang M, Li Y, Jiang JH, Song F. Effects of Tianwang Buxin Pills on Somatization Symptoms and Anxiety and Depression in Patients with Myocardial Bridges. Chin Naturopathy. 2021;29(11):62-66. DOI:10.19621/j.cnki.11-3555/r.2021.1124
