# Supplementary material for: Efficacy and safety of Traditional Chinese Medicine in alleviating symptoms associated with myocardial bridge: a systematic review and meta-analysis
Source: Front Pharmacol. 2025 Sep 19;16:1619617. doi: 10.3389/fphar.2025.1619617 (PMC12492955; doi:10.3389/fphar.2025.1619617)
Supplement: Supplementary file 5 [file DataSheet5.pdf]

Herbs:

Chinese Name	Pinyin	Latin Name (with Nomenclator)	Family	Standard Format	Part(s) of plant used	Frequency	Toxicity	MPNS Link	POWO Link
丹参	Danshen	*Salvia miltiorrhiza*Bunge	Lamiaceae	*Salvia miltiorrhiza* Bunge [Lamiaceae; *Salviae miltiorrhizae radix et rhizoma*]	rhizome, root	12	Non-toxic	https://mpns.science.kew.org/mpns-portal/plantDetail?plantId=183206&query=Danshen&filter=&fuzzy=false&nameType=all&dbs=wcs	https://powo.science.kew.org/taxon/urn:lsid:ipni.org:names:456707-1
川芎#	Chuanxiong	*Ligusticum chuanxiong* Hort.	Apiaceae	*Ligusticum chuanxiong* Hort. [Apiaceae; *Chuanxiong rhizoma*]	rhizome	11	Non-toxic	-	-
甘草	Gancao	*Glycyrrhiza glabra* L.	Fabaceae	*Glycyrrhiza glabra* L. [Fabaceae; *Glycyrrhizae radix et rhizoma*]	rhizome, root	7	Non-toxic	https://mpns.science.kew.org/mpns-portal/plantDetail?plantId=2827728&query=Gancao&filter=&fuzzy=false&nameType=all&dbs=wcs	https://powo.science.kew.org/taxon/urn:lsid:ipni.org:names:496941-1
当归	Danggui	*Angelica sinensis* (Oliv.) Diels	Apiaceae	*Angelica sinensis* (Oliv.) Diels [Apiaceae; *Angelicae sinensis radix*]	root	7	Non-toxic	https://mpns.science.kew.org/mpns-portal/plantDetail?plantId=2639272&query=Danggui&filter=&fuzzy=false&nameType=all&dbs=wcsCmp	https://powo.science.kew.org/taxon/urn:lsid:ipni.org:names:77065778-1
柴胡	Chaihu	*Bupleurum chinense* DC.	Apiaceae	*Bupleurum chinense* DC. [Apiaceae; *Bupleuri radix*]	root	6	Non-toxic	https://mpns.science.kew.org/mpns-portal/plantDetail?plantId=2686488&query=Chaihu&filter=&fuzzy=false&nameType=all&dbs=wcsCmp	https://powo.science.kew.org/taxon/urn:lsid:ipni.org:names:839099-1

Chinese Name	Pinyin	Latin Name (with Nomenclator)	Family	Standard Format	Part(s) of plant used	Frequency	Toxicity	MPNS Link	POWO Link
黄芪	Huangqi	*Astragalus mongholicus* Bunge	Fabaceae	*Astragalus mongholicus* Bunge [Fabaceae; *Astragali radix*]	root	5	Non-toxic	https://mpns.science.kew.org/mpns-portal/plantDetail?plantId=2661222&query=Huangqi&filter=&fuzzy=false&nameType=all&dbs=wcs	https://powo.science.kew.org/taxon/urn:lsid:ipni.org:names:478720-1
白芍	Baishao	*Paeonia lactiflora* Pall.	Paeoniaceae	*Paeonia lactiflora* Pall. [Paeoniaceae; *Paeoniae radix alba*]	root	5	Non-toxic	https://mpns.science.kew.org/mpns-portal/plantDetail?plantId=519125&query=Baishao&filter=&fuzzy=false&nameType=all&dbs=wcs	https://powo.science.kew.org/taxon/urn:lsid:ipni.org:names:711802-1
红花	Honghua	*Carthamus tinctorius* L.	Asteraceae	*Carthamus tinctorius* L. [Asteraceae; *Carthami flos*]	flower	5	Non-toxic	https://mpns.science.kew.org/mpns-portal/plantDetail?plantId=2900984&query=Honghua&filter=&fuzzy=false&nameType=all&dbs=wcsCmp	https://powo.science.kew.org/taxon/urn:lsid:ipni.org:names:324467-2
檀香	Tanxiang	*Santalum album* L.	Santalaceae	*Santalum album* L. [Santalaceae; *Santali albi lignum*]	wood	4	Non-toxic	https://mpns.science.kew.org/mpns-portal/plantDetail?plantId=2581919&query=Tanxiang&filter=&fuzzy=false&nameType=all&dbs=wcsCmp	https://powo.science.kew.org/taxon/urn:lsid:ipni.org:names:780592-1
三七	Sanqi	*Panax notoginseng* (Burkill) F.H. Chen	Araliaceae	*Panax notoginseng* (Burkill) F.H.Chen [Araliaceae; *Notoginseng radix et rhizoma*]	root	4	Non-toxic	https://mpns.science.kew.org/mpns-portal/plantDetail?plantId=146751&query=Sanqi&filter=&fuzzy=false&nameType=all&dbs=wcs	https://powo.science.kew.org/taxon/urn:lsid:ipni.org:names:91521-1

Chinese Name	Pinyin	Latin Name (with Nomenclator)	Family	Standard Format	Part(s) of plant used	Frequency	Toxicity	MPNS Link	POWO Link
赤芍	Chishao	*Paeonia lactiflora* Pall.	Paeoniaceae	*Paeonia lactiflora* Pall. [Paeoniaceae; *Paeoniae radix rubra*]	root	4	Non-toxic	https://mpns.science.kew.org/mpns-portal/plantDetail?plantId=519125&query=Chishao&filter=&fuzzy=false&nameType=all&dbs=wcs	https://powo.science.kew.org/taxon/urn:lsid:ipni.org:names:711802-1
麦冬	Maidong	*Ophiopogon japonicus* (Thunb.) Ker-Gawl.	Asparagaceae	*Ophiopogon japonicus* (Thunb.) Ker-Gawl. [Asparagaceae; *Ophiopogonis radix*]	root tuber	3	Non-toxic	https://mpns.science.kew.org/mpns-portal/plantDetail?plantId=279475&query=Maidong&filter=&fuzzy=false&nameType=all&dbs=wcs	https://powo.science.kew.org/taxon/urn:lsid:ipni.org:names:429781-1
党参	Dangshen	*Codonopsis pilosula* (Franch.) Nannf.	Campanulaceae	*Codonopsis pilosula* (Franch.) Nannf. [Campanulaceae; *Codonopsis radix*]	root	3	Non-toxic	https://mpns.science.kew.org/mpns-portal/plantDetail?plantId=367867&query=Dangshen&filter=&fuzzy=false&nameType=all&dbs=wcs	https://powo.science.kew.org/taxon/urn:lsid:ipni.org:names:141829-1
白术	Baizhu	*Atractylodes macrocephala* Koidz.	Asteraceae	*Atractylodes macrocephala* Koidz [Asteraceae; *Atractylodis macrocephalae rhizoma*]	rhizome	3	Non-toxic	https://mpns.science.kew.org/mpns-portal/plantDetail?plantId=2872479&query=Baizhu&filter=&fuzzy=false&nameType=all&dbs=wcsCmp	https://powo.science.kew.org/taxon/urn:lsid:ipni.org:names:182978-1
降香	Jiangxiang	*Dalbergia odorifera* T.C.Chen	Fabaceae	*Dalbergia odorifera* T.C.Chen [Fabaceae; *Dalbergiae odoriferae lignum*]	wood	3	Non-toxic	https://mpns.science.kew.org/mpns-portal/plantDetail?plantId=2755780&query=Jiangxiang&filter=&fuzzy=false&nameType=all&dbs=wcs	https://powo.science.kew.org/taxon/urn:lsid:ipni.org:names:490369-1

Chinese Name	Pinyin	Latin Name (with Nomenclator)	Family	Standard Format	Part(s) of plant used	Frequency	Toxicity	MPNS Link	POWO Link
桃仁	Taoren	*Prunus persica* (L.) Batsch	Rosaceae	*Prunus persica* (L.) Batsch [Rosaceae; *Persicae semen*]	seed	3	Non-toxic	https://mpns.science.kew.org/mpns-portal/plantDetail?plantId=2901279&query=Taoren&filter=&fuzzy=false&nameType=all&dbs=wcsCmp	https://powo.science.kew.org/taxon/urn:lsid:ipni.org:names:1212858-2
冰片	Bingpian	*Dryobalanops aromatica* C.F.Gaertn.	Dipterocarpaceae	*Dryobalanops aromatica* C.F.Gaertn. [Dipterocarpaceae; *Borneolum syntheticum*]	crystal	3	Non-toxic	https://mpns.science.kew.org/mpns-portal/plantDetail?plantId=2778746&query=Bingpian&filter=&fuzzy=false&nameType=all&dbs=wcsCmp	https://powo.science.kew.org/taxon/urn:lsid:ipni.org:names:320807-1
木香	Muxiang	*Aucklandia costus* (Falc.) Kasana & A.K.Pandey	Asteraceae	*Aucklandia costus* (Falc.) Kasana & A.K.Pandey [Asteraceae; *Aucklandiae radix*]	root	3	Non-toxic	https://mpns.science.kew.org/mpns-portal/plantDetail?plantId=3243811&query=Muxiang&filter=&fuzzy=false&nameType=all&dbs=wcsCmp	https://powo.science.kew.org/taxon/urn:lsid:ipni.org:names:77210782-1
香附	Xiangfu	*Cyperus rotundus* L.	Cyperaceae	*Cyperus rotundus* L. [Cyperaceae; *Cyperi rhizoma*]	rhizome	3	Non-toxic	https://mpns.science.kew.org/mpns-portal/plantDetail?plantId=238342&query=Xiangfu&filter=&fuzzy=false&nameType=all&dbs=wcs	https://powo.science.kew.org/taxon/urn:lsid:ipni.org:names:305797-1
枳壳	Zhiqiao	*Citrus* × *aurantium* L.	Rutaceae	*Citrus* × *aurantium* L. [Rutaceae; *Aurantii fructus*]	fruit	3	Non-toxic	https://mpns.science.kew.org/mpns-portal/plantDetail?plantId=2723957&query=Zhiqiao&filter=&fuzzy=false&nameType=all&dbs=wcsCmp	https://powo.science.kew.org/taxon/urn:lsid:ipni.org:names:59600-2

Chinese Name	Pinyin	Latin Name (with Nomenclator)	Family	Standard Format	Part(s) of plant used	Frequency	Toxicity	MPNS Link	POWO Link
枳实	Zhishi	*Citrus × aurantium* f. aurantium	Rutaceae	*Citrus × aurantium* f. aurantium [Rutaceae; Aurantii fructus immaturus]	fruit	2	Non-toxic	https://mpns.science.kew.org/mpns-portal/plantDetail?plantId=3287406&query=Zhishi&filter=&fuzzy=false&nameType=all&dbs=wcsCmp	https://powo.science.kew.org/taxon/urn:lsid:ipni.org:names:3287406-4
酸枣仁	Suanzaoren	*Ziziphus jujuba* Mill.	Rhamnaceae	*Ziziphus jujuba* Mill. [Paeoniaceae; *Ziziphi spinosae semen*]	fruit	2	Non-toxic	https://mpns.science.kew.org/mpns-portal/plantDetail?plantId=2470699&query=Suanzaoren&filter=&fuzzy=false&nameType=all&dbs=wcsCmp	https://powo.science.kew.org/taxon/urn:lsid:ipni.org:names:719213-1
熟地黄	Shudihuang	*Rehmannia glutinosa* (Gaertn.) Libosch. ex DC.	Orobanchaceae	*Rehmannia glutinosa* (Gaertn.) Libosch. ex DC. [Orobanchaceae; *Rehmanniae radix praeparata*]	root tuber	2	Non-toxic	https://mpns.science.kew.org/mpns-portal/plantDetail?plantId=2527243&query=Shudihuang&filter=&fuzzy=false&nameType=all&dbs=wcsCmp	https://powo.science.kew.org/taxon/urn:lsid:ipni.org:names:808644-1
附子	Fuzi	*Aconitum carmichaelii* Debeaux	Ranunculaceae	*Aconitum carmichaelii* Debeaux [Ranunculaceae; *Aconiti lateralis radix praeparata*]	root	2	Toxic	https://mpns.science.kew.org/mpns-portal/plantDetail?plantId=2618501&query=Fuzi&filter=&fuzzy=false&nameType=all&dbs=wcsCmp	https://powo.science.kew.org/taxon/urn:lsid:ipni.org:names:707241-1
红参	Hongshen	*Panax ginseng* C.A.Mey.	Araliaceae	*Panax ginseng* C.A.Mey. [Araliaceae; *Ginseng radix et rhizoma rubra*]	root	2	Non-toxic	https://mpns.science.kew.org/mpns-portal/plantDetail?plantId=146697&query=Hongshen&filter=&fuzzy=false&nameType=all&dbs=wcs	https://powo.science.kew.org/taxon/urn:lsid:ipni.org:names:91472-1

Chinese Name	Pinyin	Latin Name (with Nomenclator)	Family	Standard Format	Part(s) of plant used	Frequency	Toxicity	MPNS Link	POWO Link
人参	Renshen	*Panax ginseng* C.A.Mey.	Araliaceae	*Panax ginseng* C.A.Mey. [Araliaceae; *Ginseng radix et rhizoma*]	root	2	Non-toxic	https://mpns.science.kew.org/mpns-portal/plantDetail?plantId=146697&query=Renshen&filter=&fuzzy=false&nameType=all&dbs=wcs	https://powo.science.kew.org/taxon/urn:lsid:ipni.org:names:91472-1
瓜蒌	Gualou	*Trichosanthes kirilowii* Maxim.	Cucurbitaceae	*Trichosanthes kirilowii* Maxim. [Cucurbitaceae; *Trichosanthis fructus*]	seed	2	Non-toxic	https://mpns.science.kew.org/mpns-portal/plantDetail?plantId=2433223&query=Gualou&filter=&fuzzy=false&nameType=all&dbs=wcsCmp	https://powo.science.kew.org/taxon/urn:lsid:ipni.org:names:294222-1
延胡索	Yanhusuo	*Corydalis yanhusuo* (Y.H.Chou & Chun C.Hsu) W.T.Wang ex Z.Y.Su & C.Y.Wu	Papaveraceae	*Corydalis yanhusuo* (Y.H.Chou & Chun C.Hsu) W.T.Wang ex Z.Y.Su & C.Y.Wu [Papaveraceae; *Corydalis rhizoma*]	tuber	2	Non-toxic	https://mpns.science.kew.org/mpns-portal/plantDetail?plantId=2739428&query=Yanhusuo&filter=&fuzzy=false&nameType=all&dbs=wcsCmp	https://powo.science.kew.org/taxon/urn:lsid:ipni.org:names:929107-1
乳香	Ruxiang	*Boswellia sacra* Flück.	Burseraceae	*Boswellia sacra* Flück. [Burseraceae; *Olibanum*]	oleo-gum-resin	2	Non-toxic	https://mpns.science.kew.org/mpns-portal/plantDetail?plantId=2680579&query=Ruxiang&filter=&fuzzy=false&nameType=all&dbs=wcsCmp	https://powo.science.kew.org/taxon/urn:lsid:ipni.org:names:127065-1
升麻	Shengma	*Actaea cimicifuga* L.	Ranunculaceae	*Actaea cimicifuga* L. [Ranunculaceae; *Cimicifugae rhizoma*]	rhizome	2	Non-toxic	https://mpns.science.kew.org/mpns-portal/plantDetail?plantId=2620525&query=Shengma&filter=&fuzzy=false&nameType=all&dbs=wcsCmp	https://powo.science.kew.org/taxon/urn:lsid:ipni.org:names:707994-1

Chinese Name	Pinyin	Latin Name (with Nomenclator)	Family	Standard Format	Part(s) of plant used	Frequency	Toxicity	MPNS Link	POWO Link
泽泻	Zexie	*Alisma plantago-aquatica* subsp. *orientale* (Sam.) Sam.	Alismataceae	*Alisma orientale* (Sam.) Juz. [Alismataceae; *Alismatis rhizoma*]	tuber	2	Non-toxic	https://mpns.science.kew.org/mpns-portal/plantDetail?plantId=294847&query=Zexie&filter=&fuzzy=false&nameType=all&dbs=wcs	https://powo.science.kew.org/taxon/urn:lsid:ipni.org:names:60444562-2
陈皮	Chenpi	*Citrus reticulata* Blanco	Rutaceae	*Citrus reticulata* Blanco [Rutaceae; *Citri reticulatae pericarpium*]	seed	2	Non-toxic	https://mpns.science.kew.org/mpns-portal/plantDetail?plantId=2724336&query=Chenpi&filter=&fuzzy=false&nameType=all&dbs=wcsCmp	https://powo.science.kew.org/taxon/urn:lsid:ipni.org:names:772038-1
佛手*	Foshou	*Citrus medica* L. var. *sarcodactylis* Swingle	Rutaceae	*Citrus medica* L. var. *sarcodactylis* Swingle [Rutaceae; *Citri sarcodactylis fructus*]	fruit	2	Non-toxic	-	-
香橼	Xiangyuan	*Citrus medica* L.	Rutaceae	*Citrus medica* L. [Rutaceae; *Citri fructus*]	fruit	1	Non-toxic	https://mpns.science.kew.org/mpns-portal/plantDetail?plantId=2724208&query=Xiangyuan&filter=&fuzzy=false&nameType=all&dbs=wcsCmp	https://powo.science.kew.org/taxon/urn:lsid:ipni.org:names:59603-2
大枣	Dazao	*Ziziphus jujuba* Mill.	Rhamnaceae	*Ziziphus jujuba* Mill. [Rhamnaceae; *Jujubae fructus*]	fruit	1	Non-toxic	https://mpns.science.kew.org/mpns-portal/plantDetail?plantId=2470699&query=Dazao&filter=&fuzzy=false&nameType=all&dbs=wcsCmp	https://powo.science.kew.org/taxon/urn:lsid:ipni.org:names:719213-1

Chinese Name	Pinyin	Latin Name (with Nomenclator)	Family	Standard Format	Part(s) of plant used	Frequency	Toxicity	MPNS Link	POWO Link
肉桂	Rougui	*Cinnamomum cassia* (L.) Kosterm.	Lauraceae	*Cinnamomum cassia* (L.) Kosterm. [Lauraceae; *Cinnamomi cortex*]	stem bark	1	Non-toxic	https://mpns.science.kew.org/mpns-portal/plantDetail?plantId=2383714&query=Rougui&filter=&fuzzy=false&nameType=all&dbs=wcsCmp	https://powo.science.kew.org/taxon/urn:lsid:ipni.org:names:466940-1
绞股蓝	Jiaogulan	*Gynostemma pentaphyllum* (Thunb.) Makino	Cucurbitaceae	*Gynostemma pentaphyllum* (Thunb.) Makino [Cucurbitaceae; *Herba Gynostemmatis Pentaphylli*]	leaves	1	Non-toxic	https://mpns.science.kew.org/mpns-portal/plantDetail?plantId=2836473&query=Jiaogulan&filter=&fuzzy=false&nameType=all&dbs=wcsCmp	https://powo.science.kew.org/taxon/urn:lsid:ipni.org:names:292905-1
百合	Baihe	*Lilium lancifolium* Thunb.	Liliaceae	*Lilium lancifolium* Thunb. [Liliaceae; *Lilii bulbus*]	leaf	1	Non-toxic	https://mpns.science.kew.org/mpns-portal/plantDetail?plantId=279922&query=Baihe&filter=&fuzzy=false&nameType=all&dbs=wcs	https://powo.science.kew.org/taxon/urn:lsid:ipni.org:names:537628-1
太子参	Taizishen	*Pseudostellaria heterophylla* (Miq.) Pax	Caryophyllaceae	*Pseudostellaria heterophylla* (Miq.) Pax [Caryophyllaceae; *Pseudostellariae radix*]	root tuber	1	Non-toxic	https://mpns.science.kew.org/mpns-portal/plantDetail?plantId=2409729&query=Taizishen&filter=&fuzzy=false&nameType=all&dbs=wcsCmp	https://powo.science.kew.org/taxon/urn:lsid:ipni.org:names:156276-1
广藿香	Guanghuoxiang	*Pogostemon cablin* (Blanco) Benth.	Lamiaceae	*Pogostemon cablin* (Blanco) Benth. [Lamiaceae; *Pogostemonis herba*]	aerial parts	1	Non-toxic	https://mpns.science.kew.org/mpns-portal/plantDetail?plantId=162094&query=Guanghuoxiang&filter=&fuzzy=false&nameType=all&dbs=wcs	https://powo.science.kew.org/taxon/urn:lsid:ipni.org:names:454827-1

Chinese Name	Pinyin	Latin Name (with Nomenclator)	Family	Standard Format	Part(s) of plant used	Frequency	Toxicity	MPNS Link	POWO Link
半夏	Banxia	*Pinellia ternata* (Thunb.) Makino	Araceae	*Pinellia ternata* (Thunb.) Makino [Araceae; *Pinelliae rhizoma*]	tuber	1	Toxic	https://mpns.science.kew.org/mpns-portal/plantDetail?plantId=156583&query=Banxia&filter=&fuzzy=false&nameType=all&dbs=wcs	https://powo.science.kew.org/taxon/urn:lsid:ipni.org:names:972483-1
莪术	Ezhu	*Curcuma phaeocaulis* Valeton	Zingiberaceae	*Curcuma phaeocaulis* Valeton [Zingiberaceae; *Curcumae rhizoma*]	root tuber	1	Non-toxic	https://mpns.science.kew.org/mpns-portal/plantDetail?plantId=235270&query=Ezhu&filter=&fuzzy=false&nameType=all&dbs=wcs	https://powo.science.kew.org/taxon/urn:lsid:ipni.org:names:872383-1
鸡血藤	Jixueteng	*Spatholobus suberectus* Dunn	Leguminosae	*Spatholobus suberectus* Dunn [Leguminosae; *Spatholobi caulis*]	stem	1	Non-toxic	https://mpns.science.kew.org/mpns-portal/plantDetail?plantId=2597597&query=Jixueteng&filter=&fuzzy=false&nameType=all&dbs=wcs	https://powo.science.kew.org/taxon/urn:lsid:ipni.org:names:519185-1
没药	Moyao	*Commiphora myrrha* (T.Nees) Engl.	Burseraceae	*Commiphora myrrha* (Nees) Engl. [Burseraceae; *Myrrha*]	stem, branch	1	Non-toxic	https://mpns.science.kew.org/mpns-portal/plantDetail?plantId=2733595&query=Moyao&filter=&fuzzy=false&nameType=all&dbs=wcsCmp	https://powo.science.kew.org/taxon/urn:lsid:ipni.org:names:127741-1
牛膝	Niuxi	*Achyranthes bidentata* Blume	Amaranthaceae	*Achyranthes bidentata* Blume [Amaranthaceae; *Achyranthis bidentatae radix*]	root	1	Non-toxic	https://mpns.science.kew.org/mpns-portal/plantDetail?plantId=2617516&query=Niuxi&filter=&fuzzy=false&nameType=all&dbs=wcsCmp	https://powo.science.kew.org/taxon/urn:lsid:ipni.org:names:58678-1

Chinese Name	Pinyin	Latin Name (with Nomenclator)	Family	Standard Format	Part(s) of plant used	Frequency	Toxicity	MPNS Link	POWO Link
桂枝	Guizhi	*Neolitsea cassia* (L.) Kosterm.	Lauraceae	*Neolitsea cassia* (L.) Kosterm. [Lauraceae; *Cinnamomi ramulus*]	stem bark	1	Non-toxic	https://mpns.science.kew.org/mpns-portal/plantDetail?plantId=2383714&query=Guizhi&filter=&fuzzy=false&nameType=all&dbs=wcsCmp	https://powo.science.kew.org/taxon/urn:lsid:ipni.org:names:466940-1
细辛	Xixin	*Asarum heterotropoides* F.Schmidt	Aristolochiaceae	*Asarum heterotropoides* F.Schmidt [Aristolochiaceae; *Asari radix et rhizoma*]	rhizome, root	1	Toxic	https://mpns.science.kew.org/mpns-portal/plantDetail?plantId=2654341&query=Xixin&filter=&fuzzy=false&nameType=all&dbs=wcsCmp	https://powo.science.kew.org/taxon/urn:lsid:ipni.org:names:93505-1
葛根	Gegen	*Pueraria montana* var. *lobata* (Willd.) Maesen & S.M.Almeida ex Sanjappa & Predeep	Fabaceae	*Pueraria montana* var. *lobata* (Willd.) Maesen & S.M.Almeida ex Sanjappa & Predeep [Fabaceae; *Puerariae lobatae radix*]	root	1	Non-toxic	https://mpns.science.kew.org/mpns-portal/plantDetail?plantId=2537348&query=Gegen&filter=&fuzzy=false&nameType=all&dbs=wcs	https://powo.science.kew.org/taxon/urn:lsid:ipni.org:names:967441-1
薄荷	Bohe	*Mentha canadensis* L.	Lamiaceae	*Mentha canadensis* L. [Lamiaceae; *Menthae haplocalycis herba*]	aerial parts	1	Non-toxic	https://mpns.science.kew.org/mpns-portal/plantDetail?plantId=124520&query=Bohe&filter=&fuzzy=false&nameType=all&dbs=wcs	https://powo.science.kew.org/taxon/urn:lsid:ipni.org:names:450311-1
苏合香	Suhexiang	*Liquidambar orientalis* Mill.	Altingiaceae	*Liquidambar orientalis* Mill. [Altingiaceae; *Styrax*]	trunk	1	Non-toxic	https://mpns.science.kew.org/mpns-portal/plantDetail?plantId=500214&query=Suhexiang&filter=&fuzzy=false&nameType=all&dbs=wcs	https://powo.science.kew.org/taxon/urn:lsid:ipni.org:names:430714-1

Chinese Name	Pinyin	Latin Name (with Nomenclator)	Family	Standard Format	Part(s) of plant used	Frequency	Toxicity	MPNS Link	POWO Link
玫瑰花	Meiguihua	*Rosa rugosa* Thunb.	Rosaceae	*Rosa rugosa Thunb.* [Rosaceae; *Rosae rugosae flos*]	flower	1	Non-toxic	https://mpns.science.kew.org/mpns-portal/plantDetail?plantId=2902118&query=Meiguihua&filter=&fuzzy=false&nameType=all&dbs=wcsCmp	https://powo.science.kew.org/taxon/urn:lsid:ipni.org:names:927373-1
乌药	Wuyao	*Lindera aggregata* (Sims) Kosterm.	Lauraceae	*Lindera aggregata* (Sims) Kosterm. [Lauraceae; *Linderae radix*]	root tuber	1	Non-toxic	https://mpns.science.kew.org/mpns-portal/plantDetail?plantId=2351909&query=Wuyao&filter=&fuzzy=false&nameType=all&dbs=wcsCmp	https://powo.science.kew.org/taxon/urn:lsid:ipni.org:names:465318-1
薤白	Xiebai	*Allium macrostemon* Bunge	Alliaceae	*Allium macrostemon* Bunge [Alliaceae; *Allii macrostemonis bulbus*]	bulb	1	Non-toxic	https://mpns.science.kew.org/mpns-portal/plantDetail?plantId=295955&query=Xiebai&filter=&fuzzy=false&nameType=all&dbs=wcs	https://powo.science.kew.org/taxon/urn:lsid:ipni.org:names:528368-1
玄参	Xuanshen	*Scrophularia ningpoensis* Hemsl.	Scrophulariaceae	*Scrophularia ningpoensis* Hemsl. [Scrophulariaceae; *Scrophulariae radix*]	root	1	Non-toxic	https://mpns.science.kew.org/mpns-portal/plantDetail?plantId=2587380&query=Xuanshen&filter=&fuzzy=false&nameType=all&dbs=wcsCmp	https://powo.science.kew.org/taxon/urn:lsid:ipni.org:names:809386-1
栀子	Zhizi	*Gardenia jasminoides* J.Ellis	Rubiaceae	*Gardenia jasminoides* J.Ellis [Rubiaceae; *Gardeniae fructus*]	fruit	1	Non-toxic	https://mpns.science.kew.org/mpns-portal/plantDetail?plantId=88270&query=Zhizi&filter=&fuzzy=false&nameType=all&dbs=wcs	https://powo.science.kew.org/taxon/urn:lsid:ipni.org:names:751073-1

Chinese Name	Pinyin	Latin Name (with Nomenclator)	Family	Standard Format	Part(s) of plant used	Frequency	Toxicity	MPNS Link	POWO Link
苦参	Kushen	*Sophora flavescens* Aiton	Fabaceae	*Sophora flavescens* Aiton [Fabaceae; *Sophorae flavescentis radix*]	root	1	Non-toxic	https://mpns.science.kew.org/mpns-portal/plantDetail?plantId=2597260&query=Kushen&filter=&fuzzy=false&nameType=all&dbs=wcs	https://powo.science.kew.org/taxon/urn:lsid:ipni.org:names:518831-1
桑枝	Sangzhi	*Morus alba* L.	Moraceae	*Morus alba* L. [Moraceae; *Mori ramulus*]	branch	1	Non-toxic	https://mpns.science.kew.org/mpns-portal/plantDetail?plantId=2501381&query=Sangzhi&filter=&fuzzy=false&nameType=all&dbs=wcsCmp	https://powo.science.kew.org/taxon/urn:lsid:ipni.org:names:30051955-2
五味子	Wuweizi	*Schisandra chinensis* (Turcz.) Baill.	Schisandraceae	*Schisandra chinensis* (Turcz.) Baill. [Schisandraceae; *Schisandrae chinensis fructus*]	fruit	1	Non-toxic	https://mpns.science.kew.org/mpns-portal/plantDetail?plantId=381262&query=Wuweizi&filter=&fuzzy=false&nameType=all&dbs=wcs	https://powo.science.kew.org/taxon/urn:lsid:ipni.org:names:60456331-2
高良姜	Gaoliangjiang	*Alpinia officinarum* Hance	Zingiberaceae	*Alpinia officinarum* Hance [Zingiberaceae; *Alpiniae officinarum rhizoma*]	rhizome	1	Non-toxic	https://mpns.science.kew.org/mpns-portal/plantDetail?plantId=218941&query=Gaoliangjiang&filter=&fuzzy=false&nameType=all&dbs=wcs	https://powo.science.kew.org/taxon/urn:lsid:ipni.org:names:795352-1
荜茇	Bibo	*Piper longum* L.	Piperaceae	*Piper longum* L. [Piperaceae; *Piperis longi fructus*]	fruit	1	Non-toxic	https://mpns.science.kew.org/mpns-portal/plantDetail?plantId=2568895&query=Bibo&filter=&fuzzy=false&nameType=all&dbs=wcsCmp	https://powo.science.kew.org/taxon/urn:lsid:ipni.org:names:682031-1

Chinese Name	Pinyin	Latin Name (with Nomenclator)	Family	Standard Format	Part(s) of plant used	Frequency	Toxicity	MPNS Link	POWO Link
山楂	Shanzha	*Crataegus pinnatifida* Bunge	Rosaceae	*Crataegus pinnatifida Bunge* [Rosaceae; *Crataegi fructus*]	fruit	1	Non-toxic	https://mpns.science.kew.org/mpns-portal/plantDetail?plantId=3257066&query=Shanzha&filter=&fuzzy=false&nameType=all&dbs=wcsCmp	https://powo.science.kew.org/taxon/urn:lsid:ipni.org:names:724002-1

#This source plant used in the included literatures are *Ligusticum chuanxiong* Hort. (Apiaceae). The MPNS database lists it as *Conioselinum anthriscoides 'Chuanxiong'*，but this name is unplaced in POWO. According to the standard of Pharmacopoeia of the People's Republic of China[1]，*Ligusticum chuanxiong* Hort. [Apiaceae; *Chuanxiong rhizoma*] is the standard name.

**Citrus medica* L. var. *sarcodactylis* Swingle [Rutaceae; *Citri sarcodactylis fructus*] is not listed separately in POWO and MPNS, but according to related studies[2], it is a genetic branch of *Citrus medica* L. [Rutaceae; *Citri fructus*].

[1] Chinese Pharmacopoeia Commission. Pharmacopoeia of the People's Republic of China. Vol. I. Beijing: China Medical Science Press; 2020.
[2] Wu, G., Terol, J., Ibanez, V. et al. Genomics of the origin and evolution of Citrus. Nature 554, 311-316 (2018). https://doi.org/10.1038/nature25447

Other

Chinese Name	Pinyin	Latin Name (with Nomenclator)	English Name	Standard Format	Frequency	Toxicity
水蛭	Shuizhi	*Whitmania pigra*	Medicinal Leech	*Whitmania pigra* (Medicinal Leech; *Shuizhi*)	4	Toxic
全蝎	Quanxie	*Mesobuthus martensii*	Scorpion	*Mesobuthus martensii* (Scorpion; *Quanxie*)	2	Toxic
蟾酥	Chansu	*Venenum Bufonis*	Toad-cake	*Venenum Bufonis* (Toad-cake; *Chansu*)	1	Toxic
土鳖虫	Tubiechong	*Eupolyphaga sinensis*	Ground Beetle	*Eupolyphaga sinensis* (Ground Beetle; *Tubiechong*)	1	Toxic
蝉蜕	Chantui	*Periostracum Cicadae*	Cicada Slough	*Periostracum Cicadae* (Cicada Slough; *Chantui*)	1	Non-toxic
人工麝香	Rengong Shexiang	*Moschus Artificialis*	Synthetic Musk Compound	*Moschus Artificialis* (Synthetic Musk Compound, *Rengong Shexiang*)	1	-
蜈蚣	Wugong	*Scolopendra subspinipes mutilans*	Centipede	*Scolopendra subspinipes mutilans* (Centipede; *Wugong*)	1	Toxic
地龙	Dilong	*Pheretima aspergillum*	Earthworm	*Pheretima aspergillum* (Earthworm; *Dilong*)	1	Non-toxic
人工牛黄	Rengong Niuhuang	*Bovis Calculus Artifactus*	Artificial Bile Extract	*Bovis Calculus Artifactus* (Artificial Bile Extract, *Rengong Shexiang*)	1	-
龙齿*	Longchi	*Dens Draconis*	Fossilized Mammal Tooth	*Dens Draconis* (Fossilized Mammal Tooth, *Longchi*)	1	Non-toxic
琥珀*	Hupo	*Succinum*	Fossilized Resin	*Succinum* (Fossilized Resin, *Hupo*)	1	Non-toxic

The standard formats of non-botanical drugs follow the standard of Pharmacopoeia of the People's Republic of China[1].

*They are not included in the Pharmacopoeia of the People's Republic of China[2], and its medicinal name refers to the Guangdong Provincial Standard for Chinese Materia Medica[3].

All animal-related medicines comply with the standard of Pharmacopoeia of the People's Republic of China[1] and do not involve illegal content such as endangered species.

The medicinal substances species were identified from the National Library of Medicine (https://www.ncbi.nlm.nih.gov/taxonomy).

Toxicity data obtained from the Chinese Pharmacopoeia.

[1] Chinese Pharmacopoeia Commission. Pharmacopoeia of the People's Republic of China. Vol. I. Beijing: China Medical Science Press; 2020.
[2] Wu, G., Terol, J., Ibanez, V. et al. Genomics of the origin and evolution of Citrus. Nature 554, 311-316 (2018). https://doi.org/10.1038/nature25447
[3] Guangdong Food and Drug Administration. (2011). *Guangdong Provincial Standard for Chinese Materia Medica*. Guangdong Science and Technology Press.

龙　齿

Longchi

DENS DRACONIS

本品为古代哺乳动物如三趾马、犀类、鹿类、牛类、象类等的牙齿化石。挖出后，除去泥土，敲去牙床。

【性状】 本品为齿状或破碎成不规则的块状，分犬齿和臼齿。完整者犬齿圆锥形，顶端较细或略弯曲，直径0.8~3.5cm，近尖端处断面常中空。臼齿呈圆柱形或方柱形，一端较细，略弯曲，长2~20cm，直径1~9cm，多有深浅不同的沟棱。表面呈浅蓝灰色或暗棕色者，习称"青龙齿"，呈黄白色者习称"白龙齿"。有的表面可见具光泽的釉质层（珐琅质），质坚硬，断面粗糙，凹凸不平，外层微显纤维状层纹，内面色较深，常具蓝青色或棕色条纹或斑点。吸水性强。气微、味淡。

【鉴别】（1）取本品粉末2g，滴加稀硝酸10ml，即泡沸，发生二氧化碳气体；将此气体导入氢氧化钙试液中，即产生白色沉淀。

（2）取鉴别（1）项下的反应液2ml，加水20ml，滤过，滤液加甲基红1滴，用氨试液中和至中性，取中性溶液5 ml，加硝酸银试液即产生黄色沉淀，分离，沉淀在氨试液或稀硝酸中溶解。

（3）取鉴别（2）项下的中性溶液5 ml，滴加盐酸调至恰呈酸性，再滴加草酸铵试液，即产生白色沉淀；分离，沉淀在醋酸中不溶，在稀盐酸中溶解。

【检查】 **酸不溶性灰分**　不得过8.0%（《中国药典》一部附录Ⅸ K）。

重金属　取本品粉末1g，置坩埚中小火缓缓炽热，至完全炭化，在500~600℃炽灼至完全灰化，放冷，取残渣，加冰醋酸1ml与水24ml，煮沸10分钟，放冷，滤过，滤液置纳氏比色管中，用少量水洗涤残渣及滤纸，洗液并入同一纳氏比色管中，加水稀释成25ml，照重金属检查法（《中国药典》一部附录Ⅸ E第一法）检查；含重金属不得过百万分之三十。

砷盐　取本品粉末0.2g，研细，加盐酸5ml与水23ml，加热使溶解，放冷，依法检查（《中国药典》一部附录Ⅸ F第一法），含砷量不得过百万分之十。

【炮制】 取敲去牙床的龙齿，刷净泥土，打碎，照明煅法（《中国药典》一部附录Ⅱ D）煅至酥脆。取出、放凉、碾碎，以煅龙齿粉末入药。

【性味与归经】 甘、涩，凉。归心、肝经。

【功能与主治】 镇惊安神，清热除烦。用于治惊痫癫狂，心悸怔忡，失眠多梦，身热心烦。

【用法与用量】 10~20g。

【贮藏】 置干燥处，防潮。

·起草说明·

别名： 龙牙、龙齿墩、白条龙齿、青龙齿。

琥　珀

Hupo

SUCCINUM

本品为古代松科植物的树脂埋藏于地下经年久转化而成的树脂化石。全年均可采收。按性状不同，商品分为琥珀（从地下挖出者）和煤珀（从煤中选出者）。除净沙石、泥土及煤屑等杂质。

【性状】 **琥珀**　呈不规则块状、颗粒状或多角形，大小不一，表面黄棕色，血红色或黑棕色，略透明。质硬而脆，断面平滑，有玻璃样光泽。气微，味淡。手捻易成粉末，微有涩感。

煤珀　呈不规则多角形块状或颗粒状，少数呈滴乳状，大小不一。表面淡黄色、红褐色及黑棕色，有光泽。质坚硬，不易碎，断面有玻璃样光泽，无臭，味淡。

【鉴别】 本品粉末淡黄色或淡黄棕色。不规则形碎片无色或微显淡黄色，半透明。大小不等，棱角明显，富有立体感。有的表面可见顺直的或呈波状弯曲的细微纹理，有的一侧或表面可见细密的颗粒状突起。

【检查】 取本品粉末1g，加石油醚（60~90℃）10ml，振摇，滤过，滤液加新制的0.5%醋酸铜溶液10ml，振摇，石油醚层不得显蓝绿色。

【炮制】 除去杂质，用时研成细粉。

【性味与归经】 甘，平。归心、肝、膀胱经。

【功能与主治】 安神镇惊，活血散瘀，利尿通淋。用于心悸失眠，惊风癫痫，血瘀肿痛，经闭痛经，心腹刺痛，癥瘕积聚，热淋，石淋，血淋，癃闭。

【用法与用量】 1~2g，研末吞服或入丸散剂。

【贮藏】 置干燥处。

·起草说明·

别名： 虎珀、虎魄、江珠、琥魄、血琥珀、黑琥珀、血珀、红琥珀。

本品为中成药前列通片中的一味中药材，投料前研成细粉，以粉末投料。

一般从黏土、沙层中挖出的称为琥珀，从煤层内挖出的称为煤珀，总称为琥珀。历代本草所述琥珀的来源、性状、物理性状等均与现代琥珀相同。琥珀载入《中国药典》1963年版及1977年版，之后收载于各版药典的附录中。

【来源】 为古松科松属植物的树脂，埋藏于地下经久凝结而成的碳氢化合物。

主产于云南滕冲，广西贵县，河南安阳；煤珀主产于辽宁抚顺等地。

【性状】 本品性状根据样品描述，见图1。

以色红、明亮、块整齐、易碎者为佳。
